# A Bifunctional Organic Photocatalyst for Efficient Single‐Electron and Energy Transfer Activation

**DOI:** 10.1002/anie.202509770

**Published:** 2025-07-09

**Authors:** Sumitava Mallik, Hailong Wang, Nunzio Matera, Bi‐Xiao Li, Stefano Stagni, Paolo Melchiorre

**Affiliations:** ^1^ Department of Industrial Chemistry ‘Toso Montanari’ University of Bologna via Piero Gobetti, 85 – Bologna 40129 Italy

**Keywords:** Bifunctional photocatalysis, Energy transfer (EnT), Organic photocatalyst, Photochemistry, Single‐electron transfer

## Abstract

Bifunctional photocatalysts capable of mediating both single‐electron transfer (SET) and energy transfer (EnT) processes are rare and typically metal based. Here, we present 3‐thioaryl‐4‐hydroxycoumarins, a new family of cost‐effective organic photocatalysts that leverage a stabilized charge‐transfer (CT) excited state to achieve both strong reducing power and efficient energy transfer. The spatial separation of the HOMO and LUMO stabilizes the CT state, enhancing SET reactivity (E*_red_ = −3.08 V vs. SCE) while maintaining a sufficiently high triplet energy (*E*_T_ = 67 kcal mol^−1^) for EnT‐driven transformations. This dual reactivity enables the activation of redox‐inert substrates (*E*_red_ < −2.8 V vs. SCE) via SET reduction, generating radicals suitable for diverse C─S, C‐─P, C─B, and C─C bond‐forming transformations, alongside EnT‐based processes such as *E*/*Z* olefin isomerization and [2 + 2] photocycloadditions. Mechanistic studies, supported by photophysical and theoretical analyses, confirmed the catalyst's bifunctionality.

## Graphical abstract

Thioaryl hydroxycoumarins are versatile, metal‐free photocatalysts for both single‐electron (SET), and energy transfer (EnT) pathways, enabling radical and triplet energy transfer reactions with a single organic scaffold.

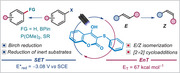

## Introduction

**Figure 1:**
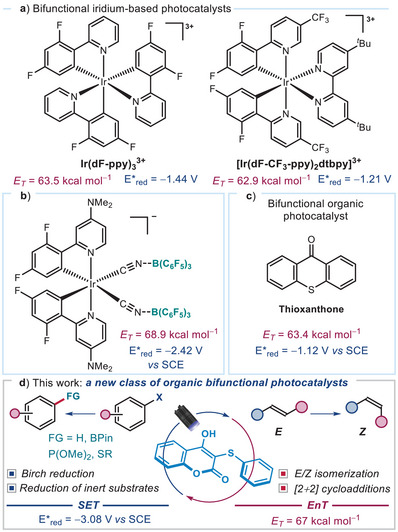
a) Iridium‐based bifunctional photocatalysts with high energy transfer (EnT) activity but moderate redox potential. b) Iridium(III) isocyanoborato complex, a strong EnT and SET photocatalyst. c) Thioxanthone, an example of an organic bifunctional photocatalyst with limited redox power. d) Our newly developed class of bifunctional photocatalysts, 3‐thioaryl‐4‐hydroxycoumarins, exhibiting both versatile SET and EnT catalytic functions. Redox potentials (*E*_red_) are reported vs. SCE in CH_3_CN.

Photocatalysis has advanced modern synthesis significantly,^[^ ^1^ ^]^ enabling highly selective transformations under mild conditions. Photocatalysts typically activate substrates through single‐electron transfer (SET)^[^ ^2^ ^]^ or energy transfer (EnT)^[^ ^3^, ^4^ ^]^ mechanisms. However, achieving both high EnT efficiency and strong SET reducing power within a single photocatalyst remains rare and holds significant potential for streamlining catalyst selection and reaction development. Metal‐based systems, such as cyclometalated Ir(III) complexes, have shown potential as bifunctional photocatalysts (Figure 1a).^[^ ^5^, ^6^ ^]^ These complexes are effective EnT‐based photocatalysts due to their high triplet energy levels (*E*_T_ > 62 kcal mol^−1^) and long lived excited states.^[^ ^7^, ^8^, ^9^ ^]^ However, their relatively weak reduction potentials (*E*_red_ < −1.5 V vs. SCE)^[^ ^10^ ^]^ limit their ability to activate substrates via an SET mechanism. To address this limitation, modified Ir(III) complexes incorporating isocyanoborato ligands were designed (Figure 1b),^[^ ^11^ ^]^ which exhibited improved properties, including a higher triplet‐state energy (*E*_T_ = 68.9 kcal mol^−1^) and a stronger reducing power (*E*_red_ = −2.42 V vs. SCE), enabling effective SET activation of simple organic substrates.^[^ ^12^ ^]^ An alternative strategy for bifunctional photocatalysis involves Coulombic dyads,^[^ ^13^ ^]^ where electrostatic interactions between a cationic ruthenium complex and an anionic pyrene derivative enable efficient energy and electron transfer.

While transition‐metal systems have proven valuable, organic photocatalysts^[^ ^14^, ^15^ ^]^ offer a compelling alternative due to their cost‐effectiveness and accessibility. However, the development of bifunctional organic photocatalysts capable of efficiently combining strong SET and EnT functions has lagged behind. Aromatic ketones, exemplified by thioxanthone^[^ ^16^, ^17^ ^]^ (Figure 1c), are the main class of organic photocatalysts investigated for dual reactivity.^[^ ^18^, ^19^ ^]^ While thioxanthone is effective in EnT activation, with a triplet energy of 63.4 kcal mol^−1^, its limited photoreductive power (E*_red_ = −1.12 V vs. SCE) underscores the challenge of designing organic photocatalysts that can efficiently mediate both SET and EnT processes.

**Figure 2:**
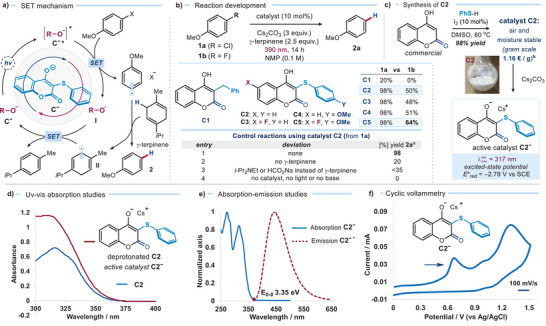
a) Proposed SET‐based mechanism using the 4‐hydroxycoumarin‐derived catalysts **C** for the hydrodefunctionalization of redox‐inert aryl halides **1**; **R‐O^−^** indicates the deprotonated catalyst **C^−^** while **R‐O** ^∙^ the resulting radical. b) Catalyst screening and control experiments; reactions performed on a 0.2 mmol scale using 1 equiv. of chloroanisole **1a** or fluoroanisole **1b** under illumination by a purple LED (*Kessil* lamp) at 390 nm. c) Synthesis of catalyst **C2** and preparation of the active catalytic salt **C2^−^**. d) UV–vis absorption spectra of catalyst **C2** and the corresponding anion **C2^−^** (formed in situ treating **C2** with 3 equiv. of Cs_2_CO_3_) in CH_3_CN (10^−4^ M). e) Emission of the excited anion C2^−*^ in CH_3_CN (formed in situ treating **C2** with Cs_2_CO_3_) upon irradiation at 354 nm and its intercept at 370 nm with the normalized absorption spectrum, with a 0–0 transition energy (E_0,0_) of 3.35 eV. f) Cyclic voltammetry measurements of the deprotonated catalyst anion **C2^−^** (oxidation onset) carried out in CH_3_CN vs. Ag/AgCl at a scan rate of 100 mV s^−1^. Estimated redox potential vs. SCE in CH_3_CN; NMP: *N*‐methyl‐2‐pyrrolidone. ^a^Yields of **2a** measured by ^1^H NMR analysis using dimethylbromide as the internal standard. ^b^Prices of reagents estimated based on the highest quantity and purity available in the Sigma–Aldrich catalogue (February 2025).

Herein, we disclose 3‐thiophenyl‐4‐hydroxycoumarins (Figure 2d), a family of organic bifunctional photocatalysts that, upon excitation, exhibit strong SET reducing power and high energy transfer capabilities. This dual functionality enables a wide range of transformations, including the activation of redox‐inert substrates containing strong carbon‐fluorine and carbon─chlorine bonds, the Birch reductions of arenes, as well as EnT‐mediated processes like *E*/*Z* olefin photoisomerization and [2 + 2] photocycloadditions.

## Results and Discussion

### Background and Photocatalyst Design

Our catalyst design was informed by insights from our recent study on the direct photoexcitation of 4‐hydroxycoumarin derivatives.^[^ ^20^ ^]^ Specifically, we discovered that in situ deprotonated 3‐benzyl‐4‐hydroxycoumarin **C1** (Figure 2b) could be excited by purple light (390 nm) to then serve as SET reductant, enabling activation of alkyl halides to generate carbon radicals. Additionally, sparse literature reports suggest the capacity of excited 4‐hydroxycoumarins to mediate EnT processes and undergo nonradiative phosphorescence decay.^[^ ^21^, ^22^, ^23^, ^24^ ^]^ Building on these findings, we hypothesized that suitably modified 4‐hydroxycoumarins could effectively combine strong SET potential with efficient EnT activity.

We first examined the ability of **C1** and the newly designed hydroxycoumarin‐based catalysts **C2–C5** as strong SET reductants upon excitation. Mechanistically, we hypothesized that the electron‐rich anion **C^−^**, formed in situ by deprotonation of catalyst **C**, would reach a highly reducing excited state (C^−^*) under light irradiation (Figure 2a). SET activation of a difficult‐to‐reduce electron‐rich C(*sp^2^*)—X substrate **1** would generate an aryl radical. γ‐Terpinene, serving as a hydrogen atom donor, was expected to quench this aryl radical via hydrogen‐atom transfer (HAT), yielding the reduced product **2**. The resulting cyclohexadienyl radical **II** would then undergo either SET or HAT^[^ ^25^ ^]^ with the radical intermediate **I**, thereby regenerating the catalyst and completing the catalytic cycle.

### Developing Efficient Single‐Electron Transfer Catalysts

To evaluate the reducing power of our organic catalysts, we tested the hydrodechlorination of 4‐chloroanisole (**1a**, *E*_red_ = −2.9 V vs. SCE) and the more challenging defluorination of 4‐fluoroanisole (**1b**, *E*_red_ < −3.0 V vs. SCE), both requiring highly negative redox potentials. Reactions were conducted with 10 mol% of catalysts **C**, Cs₂CO₃ as the base (3 equiv.), γ‐terpinene, and under irradiation by 390 nm light in *N*‐methyl‐2‐pyrrolidone (NMP, Figure 2b). The 3‐benzyl‐4‐hydroxycoumarin catalyst **C1**, with an excited‐state reduction potential (E*_red_) = −2.85 V vs. SCE,^[^ ^20^ ^]^ yielded 20% of product **2a** from **1a** but was ineffective for reducing **1b**. To enhance the photocatalyst's redox power, we replaced the benzyl fragment on the hydroxycoumarin framework in **C1** with a thioaryl group (**C2–C5**). This modification was designed to leverage the sulfur lone pair to enhance electronic conjugation within the catalyst,^[^ ^26^ ^]^ thus improving electron delocalization and stabilizing a charge‐transfer (CT) state for more efficient redox activity.^[^ ^27^, ^28^, ^29^ ^]^ The resulting 3‐thiophenyl‐4‐hydroxycoumarin catalyst **C2** exhibited remarkable SET activity, affording the target product **2a** in 98% yield from 4‐chloroanisole **1a** and 50% yield from the fluoro substrate **1b**, successfully activating both C(*sp^2^*)─Cl and C(*sp^2^*)─F bonds. Control experiments showed that the reaction performed worse in the absence of γ‐terpinene (Figure 2b, entry 2), while alternative hydrogen donors, such as *i*‐Pr₂NEt and sodium formate, led to moderate results (entry 3). Importantly, the absence of Cs₂CO₃ completely inhibited the reactivity (entry 4). Other carbonate bases, such as Na₂CO₃ and K₂CO₃, remained effective but gave slightly reduced yields (80% and 90%, respectively), whereas organic bases (e.g., pyridine, *N,N*‐diisopropylethylamine, and 1,1,3,3‐tetramethylguanidine) failed to promote the reaction (see Table S1 in the Supporting Information. We also confirmed that the presence of the catalyst and light were essential for the reaction to occur. Notably, catalyst **C2** is an air‐stable solid that can be synthesized on a gram scale in a single step from inexpensive, commercially available 4‐hydroxycoumarin and thiophenol (Figure 2c).

We then explored further catalyst modifications (catalysts **C3**–**C5**) to evaluate whether better results could be obtained in the reduction of fluorine substrate **1b**. Catalyst **C5**, featuring an electron‐donating methoxy group on the thiophenyl scaffold and an electron‐withdrawing fluorine atom on the coumarin core, exhibited enhanced efficiency in the photoreduction of **1b** compared to the simpler catalyst **C2** (Figure 2b). This observation suggests that a push‐pull effect, by stabilizing charge‐separated states, may enhance redox activity of our photocatalysts.^[^ ^30^ ^]^

### Mechanistic Insights Into SET Activity and Charge‐Transfer State Formation

To understand the photocatalytic SET performance of our catalysts, we conducted both experimental and computational studies. Treatment of catalyst **C2** with Cs₂CO₃ (3 equiv.) in CD_3_CN rapidly and quantitatively generated the electron‐rich anion **C2^−^**, as confirmed by ¹H NMR analysis (see Section F4 in the Supporting Information (SI) for details). Absorption spectroscopic analysis (Figure 2d, see also Section F2.1 in the Supporting Information) revealed that catalyst **C2** lacks significant absorption in the visible region. Deprotonation with Cs₂CO₃ to form **C2⁻** induced a minimal bathochromic shift, extending absorption to ∼390 nm (the excitation wavelength used in this study) though the extinction coefficient remained low (*ε*_390_ = 81 M⁻¹ cm⁻¹ at 1 mM). Upon excitation at 354 nm of anion **C2^−^** in CH_3_CN (in situ‐generated by mixing **C2** with Cs₂CO₃), an emission maximum at 445 nm was detected, confirming that **C2^−^** could access an electronically excited state (Figure 2e). From the overlap of normalized UV–visible absorption and emission spectra, the 0−0 transition energy (*E*₀,₀) was determined to be 3.35 eV at 370 nm. Next, electrochemical studies using cyclic voltammetry revealed that anion **C2^−^** exhibited an irreversible oxidation peak at +0.65 V vs. Ag/AgCl in CH₃CN (Figure 2f). Using the Rehm–Weller formalism,^[^ ^31^ ^]^ the redox potential of the excited state of anion **C2^−^** (E(**I**/C2^−^*)) was estimated to be −2.78 V vs. SCE, confirming its strong reducing capability upon excitation. Further experimental evidence from Stern–Volmer quenching experiments (see Section F2.6 and Figure S21 for details) demonstrated that the fluorescence of excited **C2^−^** was quenched by 4‐chloroanisole **1a** (*K_SV_* = 1.5 × 10^−3^ M^−1^; *k_q_* = 5.0 x 10^5^ M^−1^ s^−1^), corroborating its potential for productive SET reactivity. Similar experiments were performed for photocatalysts **C3**–**C5**, and the results are reported in Section F of the Supporting Information.

**Figure 3:**
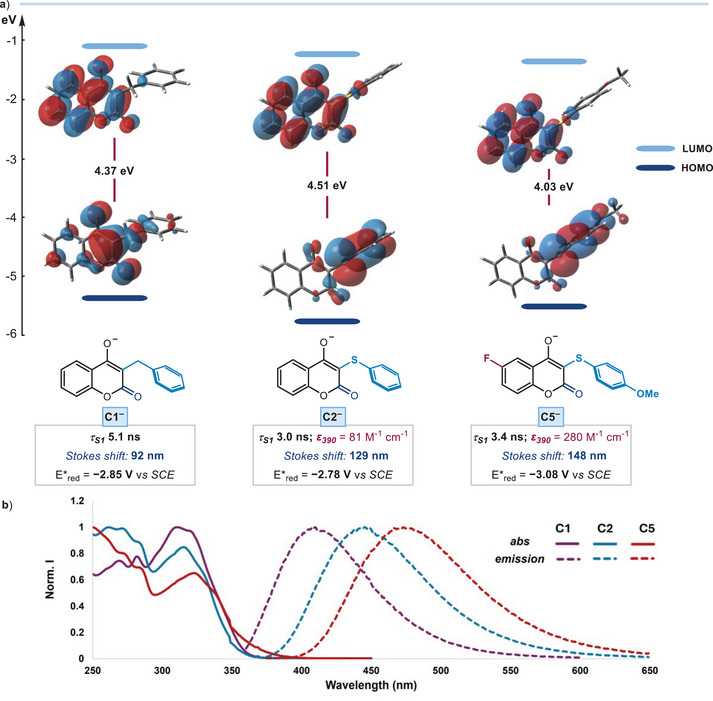
DFT‐calculated HOMO–LUMO orbitals for the deprotonated catalysts **C1**, **C2**, and **C5**, along with their experimentally measured photophysical properties; calculations were carried out at B3LYP/6‐31 + G** level of theory. ***τ*_S1_** denotes the singlet excited‐state lifetime, ***ε*_390_** is the molar extinction coefficient at 390 nm (1 mM concentration), the *Stokes shift* refers to the difference in wavelength between the maxima of absorption and emission spectra of the deprotonated catalysts, and E*_red_ indicates the excited‐state reduction potentials. **b**) Absorption and emission spectra of the deprotonated catalysts **C^−^** upon laser excitation at 354 nm (10^−4^ M solutions in CH_3_CN obtained by mixing **C1, C2**, or **C5** in the presence of 3 equiv. of Cs_2_CO_3_).

Our experiments demonstrated that replacing the benzyl substituent in catalyst **C1** with the thiophenyl fragment in catalysts **C2** and **C5** significantly enhanced photoreductive performance. We therefore used a combination of photophysical data and computational investigations to rationalize the enhanced SET function of **C2** and **C5** (Figure 3). Photophysical characterization revealed that catalysts **C2** and **C5** exhibited similar singlet excited‐state lifetimes (3.0 ns for deprotonated **C2** and 3.4 ns for deprotonated **C5**) as catalyst **C1** (5.1 ns). However, catalysts **C2** and **C5** displayed a more pronounced charge‐transfer (CT) state,^[^ ^30^, ^32^ ^]^ as indicated by the increased Stokes shifts compared to **C1**. Given that the singlet excited‐state lifetimes and redox potentials of the excited states are comparable (Figure 3a, redox potential of the excited anion C5^−^* estimated as −3.08 V vs. SCE), this suggests that the enhanced performance of **C2** and **C5** is not primarily due to thermodynamic differences but rather kinetic factors. The greater stabilization of the CT state in **C2** and **C5** may contribute to a more efficient SET process due to more stable excited‐state dynamics and electron‐transfer kinetics.^[^ ^27^, ^28^, ^29^, ^30^ ^]^

To better understand the influence of the substituents on the photocatalyst scaffold, we performed DFT calculations at the B3LYP/6‐31 + G** level of theory, using the PCM solvent model to account for solvation effects. Time‐dependent DFT (TDDFT) calculations revealed that the primary electronic transition (S₀ → S₁) for all deprotonated catalysts **C1**–**C5** was governed by the HOMO–LUMO transition, with notable differences across the series (see Figure 3a and Section F5 in Supporting Information for details). Computational studies showed that in **C1**, the HOMO was predominantly localized on the carbonyl group of the hydroxycoumarin core, limiting electronic communication within the catalyst. In contrast, the incorporation of a thiophenyl substituent in **C2** shifted the HOMO to the thiophenyl ring, lowering its energy by 0.33 eV and enhancing electronic delocalization. This structural modification facilitated the formation of a more stabilized CT state, as corroborated by the observed Stoke shift differences of **C2** and **C5**. Further, computational studies revealed that the introduction of additional substituents in **C5**, specifically a fluorine electron‐withdrawing group on the hydroxycoumarin core and a methoxy electron‐donating group on the thiophenyl fragment, lowered the HOMO‐LUMO gap by 0.48 eV and further increased the Stokes shift. Overall, these studies, combined with the experimental data, support the conclusion that the enhanced CT state stabilization in **C2** and **C5** is key to their superior SET properties and improved catalytic performance. To further validate our design strategy, we also computationally screened all possible permutations of MeO and F substituents across the X and Y positions, which confirmed that the substitution pattern in **C5** provides the most favorable redshift and push–pull characteristics (see Section F5.1 in the Supporting Information for details).

### Developing Efficient Energy Transfer Catalysts

**Figure 4:**
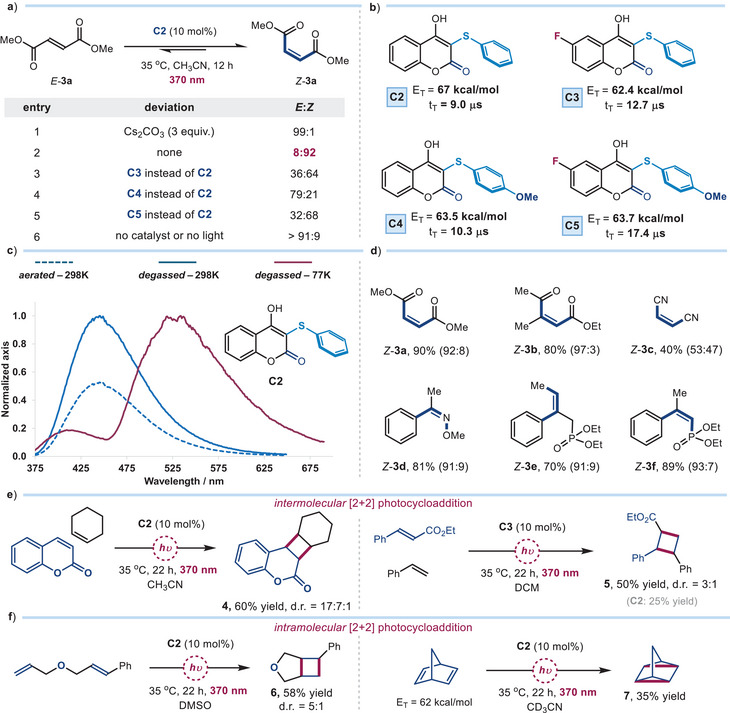
Testing the energy transfer activity. a) Photoisomerization of *E*‐methyl fumarate **3a**; the *E*/*Z* ratio was determined by ^1^H NMR analysis of the crude mixture. b) Relevant photophysical properties of neutral catalysts **C2**–**C5**. c) Normalized emission spectra of catalyst **C2** (10^−4^ M solution in CH₃CN): *blue line* represents the spectrum in degassed, deaerated CH_3_CN at room temperature; *dashed blue line* represents the spectrum in aerated CH_3_CN at room temperature; *red line* represents the spectrum in a 77 K solid matrix of degassed CH_3_CN. d) Exploring the substrate scope of the energy transfer‐mediated *E*/*Z* photoisomerization; reactions performed on a 0.2 mmol scale with 10 mol % of catalyst **C2** in dry, degassed CH_3_CN (1 mL). Yields refer to the isolated *Z* isomers of products **3**, with the *Z/E* ratio indicated in parentheses. e) Intermolecular and f) intramolecular EnT‐mediated [2 + 2] photocycloadditions; reactions performed on a 0.2 mmol scale with 10 mol % of catalyst **C2** or **C3** in dry, degassed solvent (1 mL); yields refer to the isolated products **4**–**6**, while the yield of **7** was measured by ^1^H NMR analysis using dibromomethane as the internal standard.

To evaluate the potential of our newly developed 3‐thiophenyl‐4‐hydroxycoumarin‐based compounds **C2**–**C5** to also serve as effective energy transfer catalysts, we first examined their ability to promote the *E*–*Z* photoisomerization^[^ ^33^ ^]^ of methyl fumarate *E*‐**3a** (*E*_T_ = 66.2 kcal mol^−1^) to methyl maleate *Z*‐**3a** (E_T_ = 71.0 kcal mol^−1^, Figure 4a). The high triplet energy of methyl fumarate *E*‐**3a** was selected to challenge our catalysts, pushing them to operate under conditions where a high triplet energy and long‐lived excited states are crucial for driving the transformation. The reactions were performed in CH₃CN using 10 mol% of the EnT catalyst **C** and under 370 nm light irradiation. Conducting the reaction in the presence of 3 equiv. of Cs₂CO₃, previously used for SET activation, did not provide any *E*/*Z* isomerization (entry 1). In contrast, performing the reaction without any base afforded high selectivity for the desired product *Z*‐**3a**, yielding an *E*:*Z* ratio of 8:92 (entry 2). Given this observation, the neutral forms of other catalysts were tested; however, catalysts **C3**–**C5** proved much less effective (entries 3–5). Finally, control experiments, conducted in the absence of either catalyst or light (entry 6), confirmed the necessity of both. In general, the neutral EnT catalysts performed worse under 390 nm irradiation—used to trigger SET processes— likely due to the poorer absorption at this wavelength (see Scheme S1 in the Supporting Information for details).

To rationalize these observations, we investigated the photophysical properties of catalysts **C2**–**C5** in their neutral forms, conducting studies in the absence of any base (Figure 4b). The triplet‐state lifetimes and triplet energies were determined from emission studies in a 77 K frozen matrix (Figure 4c shows data for catalyst **C2**; see Figure S20 in Supporting Information for other studies). Catalyst **C2** exhibited a triplet energy *E*_T_ of 67.0 kcal mol^−1^ and a triplet‐state lifetime of 9 µs. The presence of a triplet excited state was further confirmed by comparing the emission spectra of **C2** under degassed and aerated conditions at room temperature (Figure 4c, blue vs. dashed blue line), where oxygen quenching indicated the involvement of a long‐lived triplet excited state. As for catalysts **C3**–**C5**, which were not effective in promoting *E*/*Z* isomerization, this was likely due to their lower triplet energies (*E*_T_ ranging from 62.4 to 63.7 kcal mol^−1^), which may be insufficient to effectively activate *E*‐**3a** via EnT sensitization.

To rationalize the difference in EnT activity between the neutral and deprotonated forms of catalyst **C2** in the photoisomerization of dimethyl fumarate *E*‐**3a** (entries 1 & 2 in Figure 4a), we performed Stern–Volmer fluorescence quenching studies using *E*‐**3a** as the quencher (see section F2.6 for details). The deprotonated catalyst **C2⁻** displayed linear quenching behavior (*K_SV_* = 5.3 × 10^−2^ M^−1^), while GC‐MS analysis confirmed the formation of dimethyl succinate, the reduced product derived from **3a** (see Scheme S2 in Supporting Information for details), suggesting that a photoinduced SET mechanism is operative in the presence of a base. This is in line with the reduction potential of *E*‐**3a** (*E*_red_ = −1.55 V vs. Ag/AgCl),^[^ ^34^ ^]^ which renders SET thermodynamically feasible for **C2⁻**. In contrast, the neutral catalyst **C2** exhibited a nonlinear, upward‐curved and complex quenching profile (see Figure S23 in Supporting Information), which can be attributed to triplet–triplet EnT processes.^[^ ^35^ ^]^ These studies therefore suggest that a mechanistic shift from EnT to SET takes place upon deprotonation of the catalyst.

Next, we explored the reactivity of various *E*‐olefins **3** in the *E*/*Z* photoisomerization using the neutral catalyst **C2** (Figure 4d). An unsymmetrical trisubstituted *E*‐keto ester was isomerized to the corresponding *Z*‐**3b** in good yield with high selectivity (97:3 *Z/E* ratio). The low 53:47 *Z*/*E* ratio observed for fumaronitrile **3c** is consistent with previous literature reports.^[^ ^36^ ^]^ This result can be explained by the fact that both *E* and *Z* isomers of **3c** have a similar triplet energy of ≈60 kcal mol^−1^, thus resulting in an approximately equilibrated ratio of isomers at the photostationary state.^[^ ^36^ ^]^ We then evaluated the EnT‐based isomerization of the oxime derivative **3d**.^[^ ^37^ ^]^ The recovered high selectivity (91:9 *Z*/*E* ratio) is congruent with the large triplet energy difference between the *E*‐**3d** (59 kcal mol^−1^) and *Z*‐**3d** (72 kcal mol^−1^)^[^ ^38^ ^]^ isomers, which aligns well with the catalyst's triplet energies, enabling selective isomerization. To further extend the scope of photoisomerization, we investigated styrene derivatives. While *trans*‐β‐methylstyrene did not undergo efficient *E*/*Z* isomerization (see Figure S1 in Supporting Information for a full list of unreactive or moderately reactive substrates), the introduction of a phosphonate group at the β‐position (substrate **3e**) resulted in a *Z*/*E* ratio of 91:9 for *Z*‐**3e**. This outcome is consistent with the reported strategy of leveraging destabilizing A_1,3_‐strain to deconjugate the *Z*‐alkene chromophore, thereby increasing its triplet energy.^[^ ^33^, ^39^, ^40^, ^41^, ^42^, ^43^ ^]^ Also diethyl (*E*)‐(2‐phenylprop‐1‐en‐1‐yl)phosphonate was efficiently isomerized to *Z*‐**3f** in a 93:7 *Z/E* ratio.^[^ ^44^ ^]^

Beyond *E→Z* isomerization, our new organic photocatalysts were suitable for mediating another hallmark transformation of energy transfer catalysis: [2 + 2] photocycloadditions.^[^ ^45^ ^]^ Specifically, we focused on reactions previously reported in the literature and known to proceed via triplet‐sensitization. In the absence of base, catalyst **C2** efficiently catalyzed the EnT‐mediated *intermolecular* [2 + 2] cycloaddition of coumarin (*E*_T_ = 63.2 kcal mol^−1^) and cyclohexene, yielding cyclobutene product **4** (Figure 4e, left panel).^[^ ^46^ ^]^ In this reaction, we also detected a minor amount (∼15%) of coumarin homodimerization, which was the only case where such byproducts were observed in our study. Interestingly, as depicted in Figure 4e, right panel, catalyst **C3** outperformed **C2** in the EnT‐driven [2 + 2] reaction between styrene and ethyl cinnamate (E_T_ ≈ 49 kcal mo^−1^l for methyl cinnamate),^[^ ^47^ ^]^ delivering product **5** in 50% yield (**C2** offered 25% yield).^[^ ^48^ ^]^ These results demonstrate how structurally related catalyst variants can offer a versatile platform to fine‐tune reactivity across different substrates and reaction types. Additionally, **C2** smoothly promoted the *intramolecular* [2 + 2] photocycloaddition of a styrene derivative (Figure 4f, left panel),^[^ ^49^ ^]^ delivering product **6** in 58% yield. **C2** proved also effective in the *intramolecular* [2 + 2] cycloaddition of norbornadiene (*E*_T_ = 62.3 kcal mol^−1^)^[^ ^11^ ^]^ to quadricyclane **7**, affording the product in moderate yield (Figure 4f, right panel). Overall, these results highlight that our catalyst platform is useful for promoting diverse classical triplet‐sensitized photochemical transformations.

### Further Synthetic Applications via SET Activation

**Figure 5:**
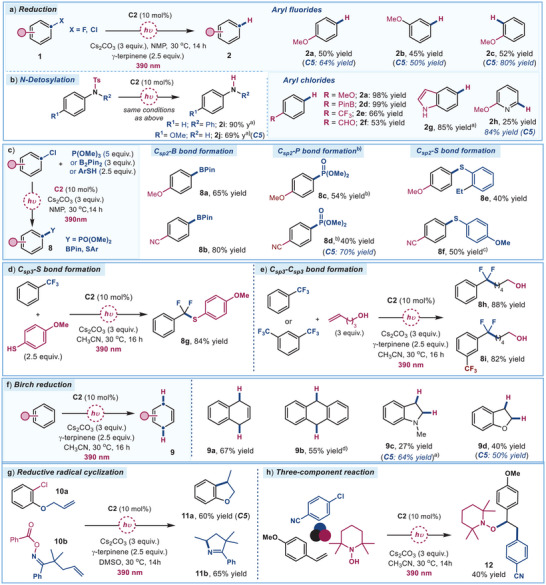
Synthetic Applications via SET Activation. a) Reductive dehalogenation of aryl fluorides and chlorides. b) *N*‐Detosylation of selected amine substrates. c) Photocatalytic borylation, phosphorylation, and thiolation of aryl chlorides. d) Selective mono‐defluorination of trifluorotoluene, followed by thiophenol trapping, yielding CF_2_─S bond formation. e) Radical‐mediated C─C bond formation via aryl‐CF_2_· addition to unactivated olefins. f) Light‐induced Birch reductions of unactivated arenes. g) Radical cyclizations. h) Three‐component radical difunctionalization. Reactions performed on a 0.2 mmol scale using photocatalyst **C2** or **C5** (10 mol%) in a PhotoRedOx Box TC™ reactor under illumination by a Kessil lamp (max 52 W, λ_max_ = 390 nm). Yields of products **2** and **9** were determined by ¹H NMR analysis using dimethylbromide as an internal standard, unless otherwise noted. Yields of products **8**, **11**, and **12** refer to isolated materials after purification. ^a)^ Yield of the isolated purified product. ^b)^ Performed using HCO_2_Na (1 equiv.) instead of γ‐terpinene. ^c)^ *t*BuONa used instead of C_2_CO_3_. ^d)^ Anthracene‐9‐carbonitrile was used. NMP: *N*‐methyl‐2‐pyrrolidone.

To fully explore the applicability of our organophotocatalytic system, we focused on the SET activation of redox‐inert aryl halides characterized by highly negative reduction potentials (*E*_red_ < −3.0 V vs. SCE) and high bond dissociation energies (∼125 ± 2 kcal mol^−1^). To enable the SET reducing power of our system, we used catalyst **C2** in the presence of Cs_2_CO_3_ (3 equiv.). **C2** was chosen due to its low cost and easy accessibility; however, when its performance was insufficient, we evaluated catalyst **C5** as an alternative. We first examined a series of fluoroanisole derivatives, which underwent efficient reductive dehalogenation to afford the corresponding anisoles **2a–c** in good yields (Figure 5a). To further assess the catalytic efficiency of **C2**, we extended the study to both electron‐rich and electron‐deficient chlorides. These substrates, known for their resistance to SET reduction,^[^ ^50^ ^]^ were successfully converted into the desired hydrochlorination products **2a, 2d–h**, achieving high yields. In cases where lower yields were observed, a simple switch to catalyst **C5** led to significantly improved outcomes (e.g., **2c** and **2h**). Importantly, the high yield of products **2g** and **2h** demonstrates the compatibility of our catalyst with heteroaromatic chlorides. The versatility of our catalytic system was further demonstrated by the successful reductive detosylation of *N*,*N*‐diphenyl‐*p*‐toluenesulfonamide and *N*‐(4‐methoxy‐phenyl)‐4‐methyl‐benzenesulfonamide, affording products **2i** and **2j** in high yield (Figure 5b).^[^ ^51^ ^]^ The synthetic utility of our platform was further demonstrated by intercepting the aryl radicals generated upon catalyst‐mediated reductive cleavage of aryl chlorides (Figure 5c). Specifically, the use of bis(pinacolato)diboron (B₂pin₂) and trimethyl phosphite (P(OMe)₃) enabled efficient formation of C(*sp^2^*)─B and C(*sp^2^*)─P bonds within products **8a–b** ^[^ ^52^ ^]^ and **8c–d**,^[^ ^53^ ^]^ respectively. C(*sp^2^*)─S bond formation was also achieved by reacting aryl chlorides and aryl thiols, which afforded the target products **8e–f**. The experiments in Figure 5c were performed using electron‐rich (*para*‐MeO) and electron‐poor (*para*‐CN) aryl chlorides, chosen as model substrates to demonstrate the feasibility of the process regardless of the electronic nature of the radical precursors. In another application, catalyst **C2** was effective in the selective mono‐defluorination of trifluorotoluene, achieved via SET reduction (Figure 5d).^[^ ^54^ ^]^ The resulting aryl‐CF₂· radical was efficiently trapped with thiophenol, providing a streamlined approach to CF₂─S bond formation (product **8g**). Our catalyst's ability to efficiently reduce PhCF₃ (*E*_red_ = −3.04 V vs. SCE) was also applied in radical‐mediated C─C bond formation via addition to unactivated olefins (Figure 5e). In contrast to previous methods that required elevated temperatures,^[^ ^55^ ^]^ our system operates smoothly at room temperature, delivering product **8h**. Also 1,3‐bis(trifluoromethyl)benzene (*E*_red _= −2.07 V vs. SCE) could be readily activated via SET to yield product **8i**.

We also evaluated the capability of catalyst **C2** to drive light‐induced Birch reductions of unactivated arenes (Figure 5f).^[^ ^50^, ^56^ ^]^ This transformation is particularly difficult due to the absence of leaving groups, which often leads to unproductive back‐electron transfer. Our organic catalyst enabled the activation of naphthalene, affording the corresponding dihydro product in good yield (**9a**). In the case of anthracene‐9‐carbonitrile, the reaction proceeded via decyanation followed by Birch reduction (**9b**). As for more challenging substrates, such as *N*‐methyl indole and benzofurane, the use of catalyst **C5** proved effective to achieve appreciable yields (adducts **9c–d**). We then showcased the versatility of our catalyst in driving radical cyclizations (Figure 5g). The chloro‐substrate **10a** was successfully activated to afford 2,3‐dehydrogenated benzofuran **11a**,^[^ ^57^ ^]^ while the reductive imino cyclization from **10b** smoothly led to product **11b**. Finally, we used catalyst **C2** to promote a three‐component difunctionalization of *para*‐methoxy styrene with 4‐cyano‐chlorobenzene and 2,2,6,6‐tetramethylpiperidin‐1‐ol (TEMPO‐H), delivering product **12** (Figure 5h).^[^ ^58^ ^]^ Overall, these results highlight the potential of our catalytic system for promoting diverse and complex SET‐mediated radical transformations.

## Conclusions

In summary, we have introduced 3‐thioaryl‐4‐hydroxycoumarins as a new class of inexpensive, organic bifunctional photocatalysts capable of mediating both SET and EnT processes. These metal‐free catalysts combine strong reducing power—enabling the activation of redox‐inert substrates—with high triplet energies, allowing for efficient energy transfer. Their ability to promote a wide array of C─C, C─S, C─P, and C─B bond‐forming reactions, as well as photochemical isomerizations and cycloadditions, highlights their synthetic utility. Comprehensive mechanistic investigations, supported by photophysical and theoretical analyses, shed light on the basis of this dual activity, which is uncommon among small organic scaffolds. These mechanistic insights may provide a foundation for expanding the scope of organic photocatalysis as a sustainable alternative to transition‐metal‐based systems, while the concept of engineering charge‐transfer excited states to unify SET and EnT pathways may prove useful in guiding future advances in photocatalyst design.

## Supporting Information

Details of experimental procedures and full characterization data and copies of NMR spectra (PDF).

## Conflict of Interests

The authors declare no conflict of interest.

## Supporting information



Supporting Information

## Data Availability

The data that support the findings of this study are available in the Supporting Information of this article.
