# Supplementary material for: A Bifunctional Organic Photocatalyst for Efficient Single‐Electron and Energy Transfer Activation
Source: Angew Chem Int Ed Engl. 2025 Jul 9;64(35):e202509770. doi: 10.1002/anie.202509770 (PMC12377429; doi:10.1002/anie.202509770)

Supplementary Information for

A Bifunctional Organic Photocatalyst for Efficient Single-Electron and Energy Transfer Activation

Sumitava Mallik,† Hailong Wang,† Nunzio Matera,‡ Bi-Xiao Li,‡ Stefano Stagni, and Paolo Melchiorre*

University of Bologna, Department of Industrial Chemistry '*Toso Montanari*'

via Piero Gobetti 85, 40129 Bologna, Italy

*Correspondence to: p.melchiorre@unibo.it

Table of Contents

A. General Information 3

B. Unreactive and Poorly Reactive Substrates 4

C. Synthesis of Substrates and Catalysts 4

D. Optimization Studies 9

E. Experimental Procedures 11

- **E1.** Experimental Setup 11
- **E2.** General Procedure for Defluorination and Dechlorination 12
- **E3.** General Procedure for *N*-Detosylation 13
- **E4.** Borylation of Aryl Chlorides 14
- **E5.** General Procedure for the Phosphorylation of Aryl Chlorides 15
- **E6.** Radical Trapping by Thiophenols 16
- **E7.** Mono-Defluorinative Radical Addition of Trifluorotoluenes to Olefins 17
- **E8.** Birch Reduction 19
- **E9.** Reductive Radical Cyclization 20
- **E10.** Difunctionalization of Alkenes 21
- **E11.** Energy Transfer Catalysis 22
  - **E11.1** General Procedure for the *E*/*Z* Photoisomerization 22
  - **E11.2** General Procedure for [2+2] Photocycloadditions 24

F. Mechanistic Studies 26

- **F1.** Quantum Yield Determination 26
- **F2.** Photophysical studies 27
- **F3.** Electrochemical studies 37
- **F4.** NMR studies of deprotonated species 41
- **F5.** Computational studies 42

G. References 55

H. NMR Spectra 59

A. General Information

The NMR spectra were recorded at 400 MHz and 600 MHz for ^{1}H, 101 or 150 MHz for ^{13}C and 376 MHz for ^{19}F{^{1}H}. The chemical shift (δ) for ^{1}H and ^{13}C are given in ppm relative to residual signals of the solvents ($CHCl_3$ @ 7.26 ppm ^{1}H NMR and 77.16 ppm ^{13}C NMR). Coupling constants are given in Hertz. The following abbreviations are used to indicate the multiplicity: s, singlet; d, doublet; q, quartet; m, multiplet; br, broad signal.

High-resolution mass spectra (HRMS) were obtained from the Mass Facility unit on a Waters Xevo Q Tof spectrometer with electrospray ionization (ESI). UV-vis measurements were carried out on a Cary 3500 Multicell UV-vis spectrophotometer. Cyclic voltammetry studies were carried out on a PGSTAT204 potentiostat, offering compliance voltage up to ± 20 V (available at the counter electrode), ± 10 V scan range and ±0.4 A current range. Fluorescence measurements were carried out on an Edinburgh FLSP920 spectrometer equipped with a 450 W xenon arc lamp, double excitation and single emission monochromators, and a Peltier-cooled Hamamatsu R928P photomultiplier tube (185−850 nm).

Yields refer to isolated materials of >95% purity as determined by ^{1}H NMR analysis. When specified, yields were determined by ^{1}H NMR analysis of the crude mixture using an internal standard, typically in cases where the product was too volatile for isolation or difficult to separate from the substrate.

General Procedures. All reactions were set up under an argon atmosphere in oven-dried glassware. Synthesis grade and anhydrous solvents were used as purchased from commercial sources. Chromatographic purification of products was accomplished using forced-flow chromatography (FC) on silica gel (230-400 mesh). For thin layer chromatography (TLC) analysis throughout this work, Merck pre-coated TLC plates (silica gel 60 GF_{254}, 0.25 mm) were employed, using UV light as the visualizing agent and an acidic mixture of vanillin or basic aqueous potassium permanganate ($KMnO_4$) stain solutions, and heat as developing agents. Organic solutions were concentrated under reduced pressure on a Büchi rotatory evaporator.

Materials. Commercial grade reagents and solvents were purchased at the highest quality from commercial suppliers and used as received, unless otherwise stated.

B. Unreactive and Poorly Reactive Substrates

(a)

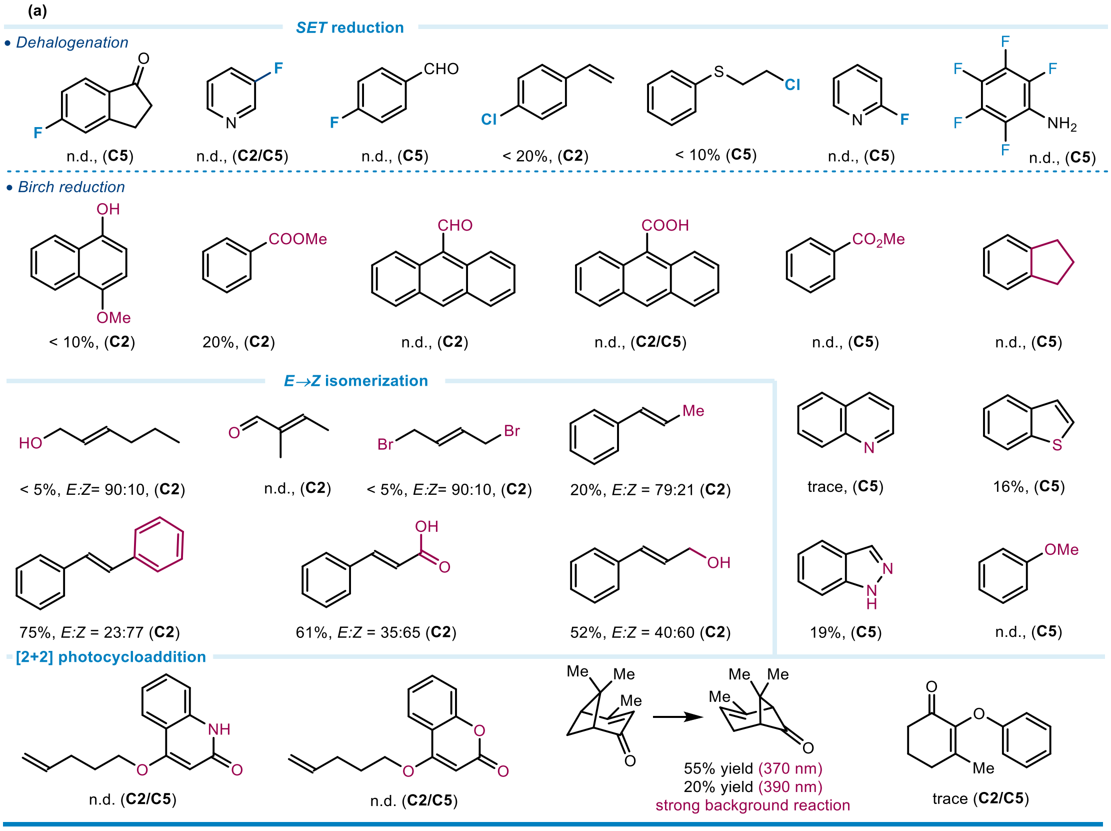

(b)

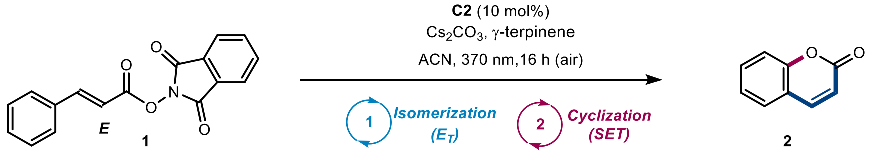

entry	base (1equiv.)	γ-terpinene	C2	Air	[%] yield **2**
1	Cs_2CO_3	2.5 equiv.	10%	yes	17
2	Cs_2CO_3	2.5 equiv.	10%	no	12
3	Cs_2CO_3	2.5 equiv.	--	yes	0
4	Cs_2CO_3	--	10%	yes	4
5	--	2.5 equiv.	10%	yes	6

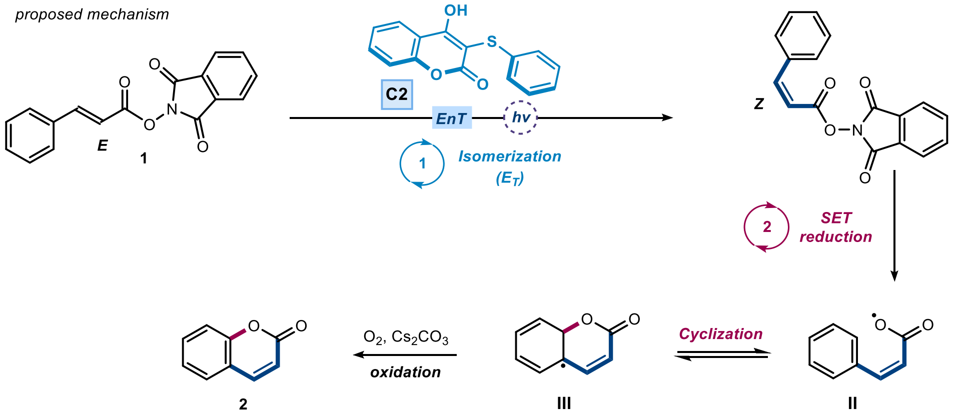

Figure S1: Unsuccessful and moderately reactive substrates. Both SET reductions and *E*/*Z* photoisomerization reactions were carried out on a 0.2 mmol scale under the optimized conditions reported in the main manuscript, using either catalyst **C2** or **C5** as specified. Results are shown below each entry. *n.d.* = *product not detected.* **(b) Attempted tandem reaction** combining EnT-mediated E/Z isomerization and SET-driven radical cyclization along with some optimization results.

C. Synthesis of Substrates and Catalysts

C1. Substrate Synthesis

The following substrates (Figure S2) were synthesized according to reported procedures.[1]

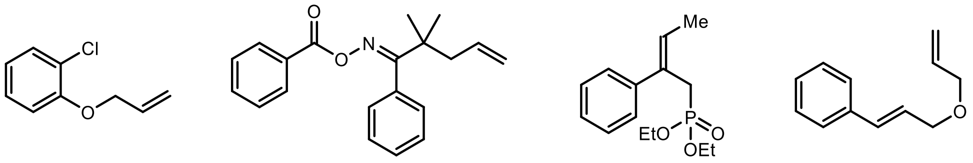

Figure S2: Starting materials synthesized according to known procedures.

General Procedure for the Synthesis of *N*-Ts Amine Derivatives

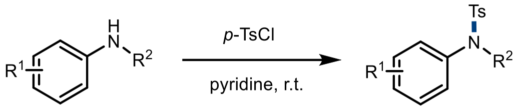

To a solution of the appropriate aniline (5 mmol, 1 equiv.) in pyridine (10 mL, 0.5 M) was added *p*-toluenesulfonyl chloride (5.5 mmol, 1.1 equiv.) at 0 °C. After the solution was stirred at ambient temperature for 2-3 hours, pyridine was removed by rotary evaporator, and the reaction mixture was poured into water. The product was extracted with CH_2Cl_2 (three times), the organic layers dried over Na_2SO_4 and concentrated in vacuo. The residue was purified by column chromatography on silica gel to give the corresponding product.

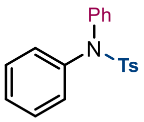

1i

***N,N*-diphenyl-p-toluenesulfonamide (1i):** Synthesized according to the General Procedure using diphenylamine (0.846g, 5 mmol, 1.0 equiv.). The crude mixture was purified by flash column chromatography on silica gel (20% ethyl acetate in hexanes as eluent) to afford **1i** (1.18g, 73% yield) as a white solid. **^{1}H NMR (400 MHz, $CDCl_3$)** δ 7.58 (d, *J* = 8.4 Hz, 2H), 7.33 – 7.25 (m, 12H), 2.43 (s, 3H). Matching reported literature data.[2]

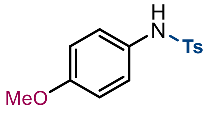

1j

***N*-Ts-4-Methoxyaniline (1j):** Synthesized according to the General Procedure using 4-methoxy-aniline (0.615g, 5 mmol, 1.0 equiv.). The crude mixture was purified by flash column chromatography on silica gel (10% ethyl acetate in hexanes as eluent) to afford **1j** (0.91g, 65% yield) as a white solid. **^{1}H NMR (400 MHz, $CDCl_3$)** δ 7.57 (d, *J* = 8.4 Hz, 2H), 7.21 (d, *J* = 8.6 Hz, 2H), 6.96 (d, *J* = 9.0 Hz, 2H), 6.76 (d, *J* = 9.1 Hz, 2H), 3.76 (s, 3H), 2.38 (s, 3H). Matching reported literature data.[2]

Synthesis of Ethyl (*E*)-3-methyl-4-oxohex-2-enoate (*E*-3b):

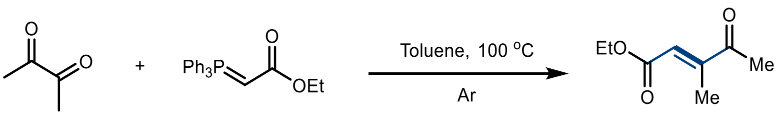

E-**3b**

To an oven-dried Schlenk tube, (carbethoxymethylene)triphenylphosphorane (1.75 g, 5 mmol, 1 equiv.) and dry toluene (5 mL) were added under an argon atmosphere. To the formed suspension, butane-2,3-dione (0.43g, 5 mmol, 1 equiv.) was added in one portion. The reaction mixture was stirred for 3 hours at 100 °C. Afterward, the mixture was allowed to cool to ambient

temperature and was diluted with Et_2O (20 mL), which caused triphenylphosphine oxide to precipitate out. The precipitate was filtered off, the solid further washed with Et_2O and the collected organic layer was concentrated with the use of a rotary evaporator. The crude material was purified by flash column chromatography on silica gel (10% Et_2O in hexanes as eluent) to afford substrate *E*-**3b** (0.32 g, 41% yield) as a clear oil.

^{1}H NMR (400 MHz, $CDCl_3$) δ 6.57 (q, *J* = 1.5 Hz, 1H), 4.24 (q, *J* = 7.1 Hz, 2H), 2.38 (s, 3H), 2.20 (d, *J* = 1.5 Hz, 3H), 1.32 (t, *J* = 7.1 Hz, 3H). Matching reported literature data.[3]

Synthesis of (*E*)-acetophenone *O*-methyloxime (*E*-3d)

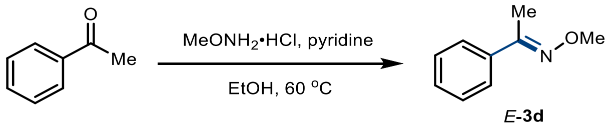

To a solution of acetophenone (2.64 g, 22.0 mmol) and pyridine (5.0 mL, 61.8 mmol) in EtOH (10 mL) was added $MeONH_2$•HCl (2.80 g, 33.0 mmol) in one portion, and the reaction mixture was stirred at 60 °C for 2 h. The reaction was quenched by adding water and extracted twice with ethyl acetate. The combined organic layers were washed with 1N aqueous HCl and brine, dried over $MgSO_4$, and the solvent was removed in a rotary evaporator. The crude material was purified by flash column chromatography on silica gel (10% ethyl acetate in hexanes as eluent) to afford *E*-**3d** (2.90 g, 88% yield) as a clear oil. **^{1}H NMR (400 MHz, $CDCl_3$)** δ 7.72 – 7.60 (m, 2H), 7.37 (dd, J = 5.3, 2.1 Hz, 3H), 4.02 (s, 3H), 2.24 (s, 3H). Matching reported literature data.[4]

Synthesis of diethyl (*E*)-(2-phenylprop-1-en-1-yl)phosphonate (*E*-3e)

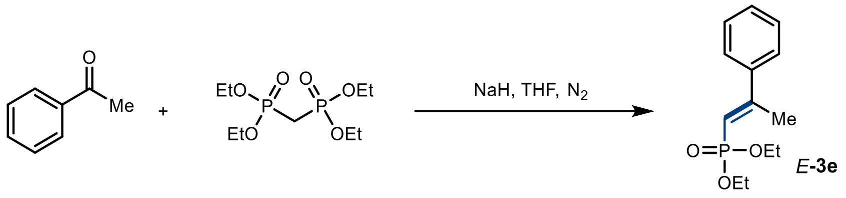

In an oven-dried Schlenk tube under argon atmosphere, sodium hydride (60% in mineral oil, 0.50g) was dissolved in dry tetrahydrofuran (10 mL) at 0 °C. Tetraethyl methylenediphosphonate (2.70 mL, 10.9 mmol, 1.27 equiv.) was added dropwise and the solution was stirred under an argon atmosphere at 0 °C for 1 hour. Acetophenone (1 mL, 8.57 mmol, 1.00 equiv.) was added via syringe and the solution was heated at 65 °C and stirred for three days. After the mixture was cooled to room temperature, water (30 mL) and ethyl acetate (40 mL) were added. The layers were separated and the aqueous layer was extracted with ethyl acetate (3 x 20 mL). The combined organic layers were dried over Na_2SO_4 and the solvent was removed in vacuo. The crude material was purified by flash column chromatography on silica gel (40% ethyl acetate in hexanes as eluent) to afford *E*-**3e** (0.81g, 37% yield) as a yellow oil.

^{1}H NMR (400 MHz, $CDCl_3$) δ 7.46 (dt, *J* = 7.2, 1.8 Hz, 2H), 7.39 – 7.32 (m, 3H), 5.90 (dt, *J* = 16.6, 1.2 Hz, 1H), 4.16 – 4.09 (m, 4H), 2.50 (dd, *J* = 3.2, 1.5 Hz, 3H), 1.35 (td, *J* = 7.1, 1.5 Hz, 6H). Matching reported literature data.[5]

C2. Catalyst Synthesis

Catalyst **C1** was synthesized following a previously reported procedure.[6]

Catalyst **C2** and the analogous 3-thiophenyl 4-hydroxycoumarin catalysts (**C3-C5**) were synthesized according to previously reported procedures,[7] with slight modifications as outlined below.

C2.1. General Procedure A

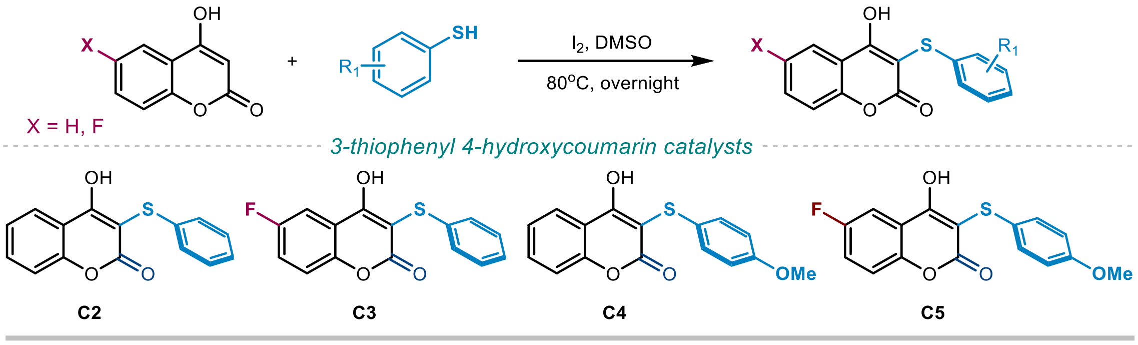

Figure S3: Synthesis of catalysts **C2-C5** according to procedure **A**.

An oven-dried Schlenk flask was charged with 4-hydroxycoumarin (1.0 equiv.), aryl thiol (1.0 equiv.), and iodine (10 mol%), which were dissolved in 0.2 M dimethyl sulfoxide. The reaction mixture was heated in a pre-heated oil bath at 80 °C overnight. The reaction was monitored by TLC analysis, then the reaction was diluted with ethyl acetate and quenched with a saturated aqueous solution of sodium thiosulfate.

*For the isolation of **C2**:* A crystalline solid precipitated in the ethyl acetate layer and was collected by filtration through a Büchner funnel. The solid was washed three times with water and ethyl acetate to afford the pure compound.

*For the isolation of **C3–C5:*** After quenching the reaction with a saturated aqueous solution of sodium thiosulfate, the organic layer was extracted with ethyl acetate, washed twice with brine, and dried over anhydrous Na_2SO_4. After filtration and concentration to dryness, the crude residue was washed with hexane, affording the corresponding pure products.

Characterization of Catalysts C

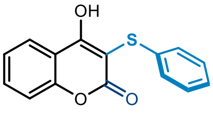

C2

4-Hydroxy-3-(phenylthio)-2*H*-chromen-2-one (C2): Synthesized according to the general procedure **A** using 4-hydroxycoumarin (1.62 g, 10.00 mmol, 1.0 equiv.), thiophenol (1.10 g, 10.00 mmol, 1.0 equiv.) and iodine (0.126 g, 1.00 mmol, 10 mol%.) in DMSO. The crystalline compound was isolated by vacuum filtration to afford **C2** (2.75 g, 98% yield) as a white solid.

^{1}H NMR (600 MHz, DMSO) δ 7.95 (dd, J = 7.9, 1.5 Hz, 1H), 7.71 (ddd, J = 8.6, 7.3, 1.6 Hz, 1H), 7.43 – 7.38 (m, 2H), 7.30 (d, J = 8.6 Hz, 2H), 7.12 (d, J = 8.6 Hz, 2H), 1.23 (s, 9H).

^{13}C NMR (151 MHz, DMSO) δ 168.7, 161.4, 153.4, 148.7, 134.0, 133.0, 126.8, 126.4, 124.8, 124.7, 117.0, 116.3, 95.1, 34.6, 31.5.

HRMS: calculated for $C_{15}H_{10}O_3S$ (M+Na$^+$): 293.0243, found 293.0244.

Matching reported literature data.[7]

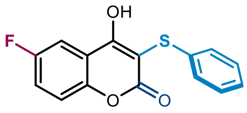

C3

6-Fluoro-4-hydroxy-3-(phenylthio)-2*H*-chromen-2-one (C3): Synthesized according to the general procedure **A** using 6-fluoro-4-hydroxycoumarin (0.214 g, 1.19 mmol, 1.0 equiv.), thiophenol (0.196 g, 1.78 mmol, 1.5 equiv.) and iodine (30.2 mg, 0.119 mmol, 10 mol%.) in DMSO. The crystalline compound was isolated by vacuum filtration to afford **C3** (0.298 g, 87% yield) as a yellow solid.

^{1}H NMR (600 MHz, DMSO) δ 7.67 (d, *J* = 8.7 Hz, 1H), 7.57 (t, *J* = 8.6 Hz, 1H), 7.47 (dd, *J* = 8.9, 4.3 Hz, 1H), 7.26 (t, *J* = 7.7 Hz, 2H), 7.15 (dd, *J* = 14.3, 7.8 Hz, 3H).
^{13}C NMR (151 MHz, DMSO) δ 167.7 (d, *J* = 22.9 Hz), 160.7 (d, *J* = 12.6 Hz), 158.0 (d, *J* = 241.2 Hz), 149.4 (d, *J* = 4.0 Hz), 135.8 (d, *J* = 17.1 Hz), 129.1, 126.3 (d, *J* = 9.9 Hz), 125.6 (d, *J* = 7.7 Hz), 120.9 (d, *J* = 24.6 Hz), 118.7 (d, *J* = 8.6 Hz), 118.2 – 116.6 (m), 109.8 (dd, *J* = 25.2, 3.8 Hz), 95.1 (d, *J* = 42.9 Hz).
^{19}F NMR (376 MHz, DMSO) δ -117.6.
HRMS: calculated for $C_{15}H_9FO_3S$ ($M+H^+$): 289.0329, found 289.0335.

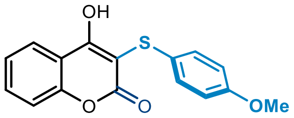

C4

4-Hydroxy-3-((4-methoxyphenyl)thio)-2*H*-chromen-2-one (C4): Synthesized according to the general procedure **A** using 4-hydroxycoumarin (1.00 g, 6.17 mmol, 1.0 equiv.), 4-methoxythiophenol (1.30 g, 9.25 mmol, 1.5 equiv.) and iodine (0.157 g, 0.617 mmol, 10 mol%.) in DMSO. The crystalline compound was isolated by vacuum filtration to afford **C4** (1.66 g, 90% yield) as a white solid.

^{1}H NMR (600 MHz, DMSO) δ 7.94 (dd, *J* = 7.9, 1.4 Hz, 1H), 7.67 (ddd, *J* = 8.4, 7.3, 1.6 Hz, 1H), 7.40 – 7.34 (m, 2H), 7.27 (d, *J* = 8.9 Hz, 2H), 6.88 (d, *J* = 8.9 Hz, 2H), 3.70 (s, 3H).
^{13}C NMR (151 MHz, DMSO) δ167.6, 161.2, 158.5, 153.1, 133.8, 130.3, 126.1, 124.6, 124.5, 116.7, 115.9, 115.1, 97.0, 55.5.
HRMS: calculated for $C_{16}H_{12}O_4S$ ($M+Na^+$): 323.0349, found 323.0352.

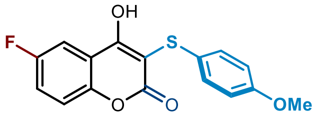

C5

6-Fluoro-4-hydroxy-3-((4-methoxyphenyl)thio)-2H-chromen-2-one (C5): Synthesized according to the general procedure **A** using 6-fluoro-4-hydroxycoumarin (0.22 g, 1.22 mmol, 1.0 equiv.), 4-methoxy thiophenol (0.171 g, 10.00 mmol, 1.0 equiv.) and iodine (31.0 mg, 0.122 mmol, 10 mol%.) in DMSO. The crystalline compound was isolated by vacuum filtration to afford **C5** (0.292 g, 75% yield) as a white solid.
^{1}H NMR (600 MHz, DMSO) δ 7.66 (dd, *J* = 8.7, 3.1 Hz, 1H), 7.55 (td, *J* = 8.4, 3.1 Hz, 1H), 7.45 (dd, *J* = 9.1, 4.4 Hz, 1H), 7.24 (d, *J* = 8.9 Hz, 2H), 6.87 (d, *J* = 8.9 Hz, 2H), 3.71 (s, 3H).
^{13}C NMR (151 MHz, DMSO) δ 166.7 (d, *J* = 18.7 Hz), 160.7 (d, *J* = 9.2 Hz), 158.3 (d, *J* = 5.5 Hz), 158.0 (d, *J* = 241.0 Hz), 149.3, 130.1 (d, *J* = 11.0 Hz), 125.7 (d, *J* = 17.3 Hz), 120.8 (dd, *J* = 24.5, 3.2 Hz), 118.6 (d, *J* = 8.5 Hz), 116.99 (m), 114.79 (s), 109.7 (d, *J* = 25.4 Hz), 97.4 (d, *J* = 34.0 Hz), 55.22 (s).
^{19}F NMR (376 MHz, DMSO) δ -117.9.
HRMS: calculated for $C_{16}H_{11}FO_4S$ ($M+Na^+$): 341.0254, found 341.0262.

D. Optimization Studies

Table S1. Optimization of the SET-based model reaction[a]

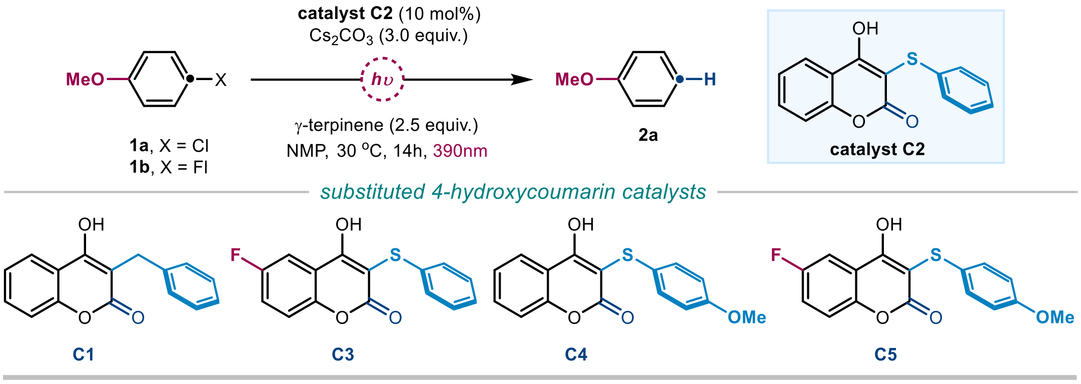

Entry	Substrate	Deviation	Yield % **2a**
1	**1a**	DMSO instead of NMP	0
2	**1a**	CH_3CN instead of NMP	16
3	**1a**	Na_2CO_3 instead of Cs_2CO_3	82
4	**1a**	K_2CO_3 instead of Cs_2CO_3	93
5	**1a**	TMG instead of Cs_2CO_3	28
6	**1a**	DIPEA instead of Cs_2CO_3	trace
7	**1a**	Pyridine instead of Cs_2CO_3	trace
8	**1a**	Under air instead of N_2	0
9	**1a**	Irradiation @ 427 nm, using **C5**	70
10	**1b**	Irradiation @ 427 nm, using **C5**	19
11	**1a**	6 h instead of 14 h, using **C5**	60
12	**1b**	6 h instead of 14 h, using **C5**	49

[a] Reactions performed over 14 hours in 0.4 mL of NMP using 0.2 mmol of **1a**, 0.5 mmol of γ-terpinene, and base (3 equiv.) under irradiation by a Kessil lamp (λ_{max} = 390 nm, irradiance = 100 mW/cm^2). Yield of **2a** determined by ^{1}H NMR analysis of the crude mixture using dibromomethane as the internal standard.

General comment: Catalyst **C5**, which exhibits better absorption in the visible region compared to **C2**, was found to effectively promote the dechlorination of **1a** under 427 nm irradiation. However, when applied to the fluoro derivative **1b**, **C5** demonstrated poor reactivity at the same wavelength. To ensure consistent results, we opted to perform all subsequent SET-based

reactions at 390 nm, even when using **C5**, as this wavelength provided more reliable and reproducible outcomes. Note that for *EnT*-based processes, irradiation at 370 nm was used instead, as it offered improved performance (see Scheme S1 in the following page).

Table S2. Optimization of the EnT-based [2+2] photocycloaddition[a]

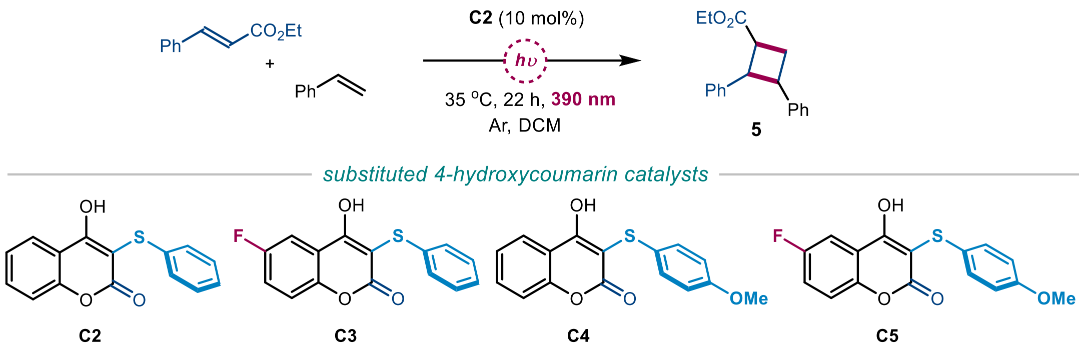

Entry	Deviation	Yield
1	none	20%
2	no catalyst or no light	0
3	CH_3CN instead of DCM	20%
4	370 nm instead of 390 nm	25%
5	**C3** instead of **C2** (370 nm)	50%
6	**C4** instead of **C2** (370 nm)	28%
7	**C5** instead of **C2** (370 nm)	30%

[a] Reactions performed over 22 hours in 1 mL of DCM using 0.2 mmol of ethyl cinnamate and styrene (1 mmol) under irradiation by a Kessil lamp (λ_{max} = 390 nm or 370 nm, irradiance = 100 mW/cm^2). Reaction yield determined by ^{1}H NMR analysis of the crude mixture using dibromomethane as the internal standard.

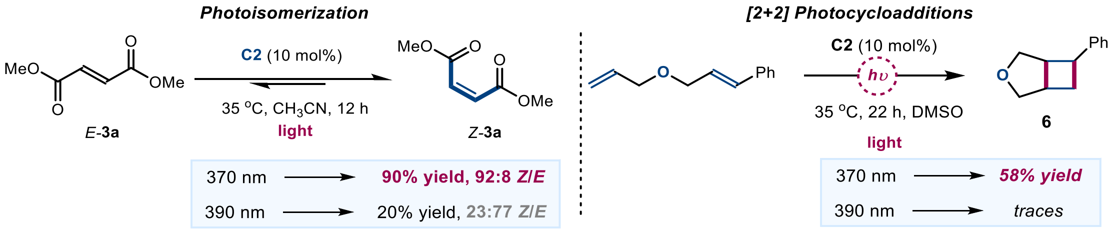

Scheme S1. Effect of light irradiation (370 nm vs 390 nm) on the [2+2] photocycloaddition reaction

E. Experimental Procedures

E1. Experimental Setup

Photochemical Set-up 1 *390 nm Kessil lamp* with **PhotoRedOx Box TC™** (Figure S4) The reactions were performed using a PhotoRedOx Box TC™ reactor under the illumination of a *Kessil* lamp (max 52 W, λ_{max} = 390 nm, positioned 2-3 cm away from the vial), with a fan to control the temperature. Under these conditions, the reaction temperature inside the vessel was measured using a thermometer and found to range between 30-32 ºC.

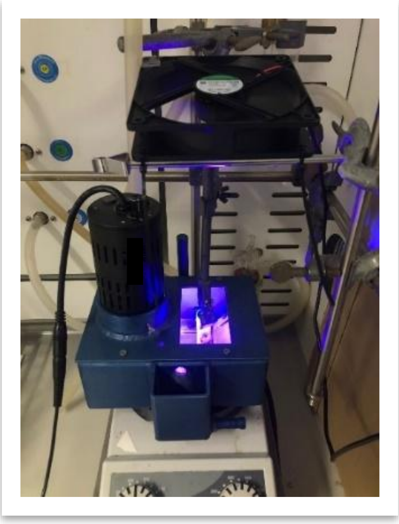

Figure S4: Reaction setup using PhotoRedOx Box TC™ reactor with 390 nm *Kessil lamp*.

Photochemical Set-up 2 *390 nm Kessil lamp* with **3D-printed photoreactor** (Figure S5) The Birch reduction reactions were performed using a 3D-printed photoreactor reported in the literature[8] under the illumination of a Kessil lamp (max 52 W, λ_{max} = 390 nm, positioned 2-3 cm away from the vial). Under these conditions, the reaction temperature in the reactor was maintained at 30 °C using a circulating water cooling system.

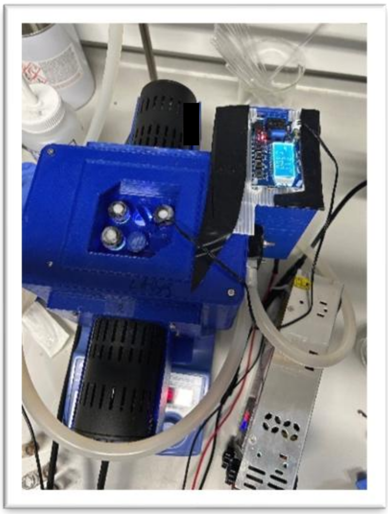

Figure S5: Reaction setup using 3D-printed photoreactor[8] with 390 nm *Kessil lamp*.

Photochemical Set-up 3 *370 nm Kessil lamp* with a Fan (Figure S6)

The EnT reactions were performed under the illumination of a Kessil lamp (max 52 W, λ_{max} = 370 nm, positioned 3-5 cm away from the Schlenk tube), with a fan to control the temperature. Under these conditions, the reaction temperature inside the vessel was measured using a thermometer and found to range between 34-36 ºC.

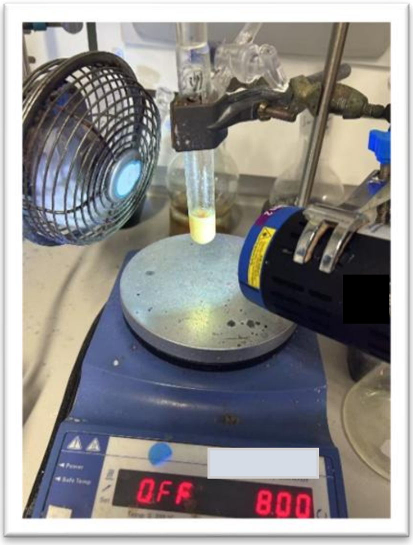

Figure S6: Reaction setup using a Fan with 370 nm or 390nm *Kessil lamp*.

E2. General Procedure for the Reductive Dehalogenation of Aryl Fluorides and Chlorides

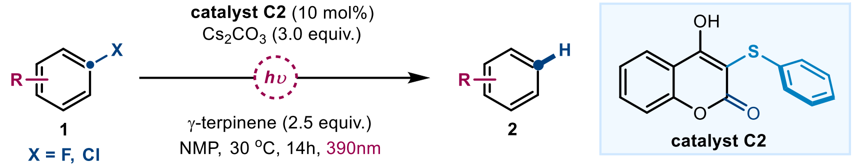

To a 4 mL glass vial, catalyst **C2** (5.4 mg, 0.02 mmol, 0.1 equiv.) or **C5** (6.4 mg, 0,02 mmol, 0.1 equiv. where specified), Cs_2CO_3 (0.6 mmol, 3 equiv.), and aryl halide **1** (*if solid*, 0.2 mmol, 1 equiv.) were sequentially added. The vial was sealed with a screw-top cap with a septum, evacuated, and backfilled with nitrogen three times. Then, aryl halide **1** (*if liquid*, 0.2 mmol, 1 equiv.), γ-terpinene (80 µL, 0.5 mmol, 2.5 equiv.), and argon-sparged NMP (0.5 M, 0.4 mL) were added via syringe. The vial was sealed with Parafilm and stirred under 390 nm Kessil lamp irradiation in a PhotoRedOx Box TC™ reactor for 14 hours using ***Set-up 1*** detailed in Figure S4.

NMR analyses (products **2a-2f**, **2h**): After irradiation at 390 nm for 14 hours, dibromomethane (34.8 mg, 0.2 mmol, 1 equiv.) was added to the crude reaction mixture as an internal standard, followed by 2 mL of H_2O and 1 mL of brine. The mixture was then extracted with 0.7 mL of $CDCl_3$, and the organic layer was analyzed by ^1H NMR spectroscopy.

Isolation of products **2g**: After completion of the reaction, the mixture was transferred to a separatory funnel, and 10 mL of H_2O and 2 mL of brine were added. The organic layer was extracted with EtOAc, washed twice with brine, and then dried over anhydrous Na_2SO_4. After

filtration and concentration to dryness, the crude residue was purified by column chromatography, affording the corresponding products with the reported yields (>95% purity according to ^{1}H NMR analysis).

Characterization of Products

The ^{1}H NMR traces of products **2a-2f, 2h**, which are reported in the NMR Section I of the Supporting Information, matched the reported literature data.[2, 9-11]

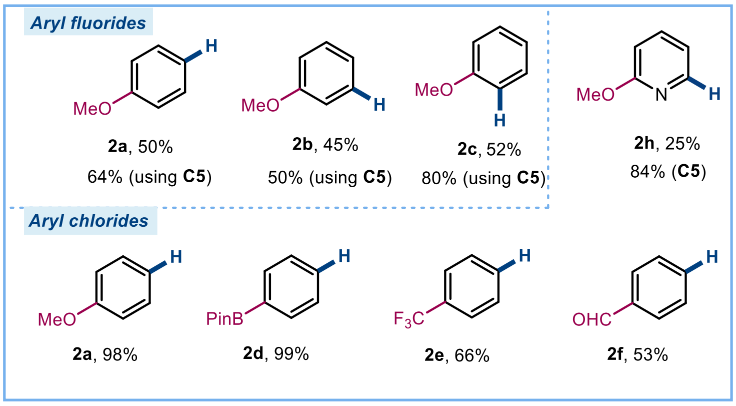

Figure S7: Compounds for which yields have been determined by ^{1}H NMR analysis using an internal standard.

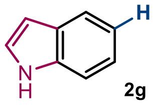

2g

1*H*-indole (2g): Synthesized according to the General Procedure using 5-chloro-1*H*-indole (30.2 mg, 0.2 mmol, 1.0 equiv.) and catalyst **C2**. The crude mixture was purified by flash column chromatography on silica gel (10% ethyl acetate in hexanes as eluent) to afford **2g** (19.8 mg, 85% yield) as a white solid.

^{1}H NMR (600 MHz, CDCl$_3$) δ 8.11 (s, 1H), 7.67 (d, J = 7.5 Hz, 1H), 7.41 (dq, J = 8.2, 0.9 Hz, 1H), 7.24 – 7.20 (m, 2H), 7.16 – 7.13 (m, 1H), 6.58 (ddd, J = 3.1, 2.0, 1.0 Hz, 1H).
^{13}C NMR (151 MHz, CDCl$_3$) δ 135.7, 127.8, 124.1, 121.9, 120.7, 119.8, 111.0, 102.6.

Matching reported literature data.[12]

E.3. General Procedure for *N*-Desotylation

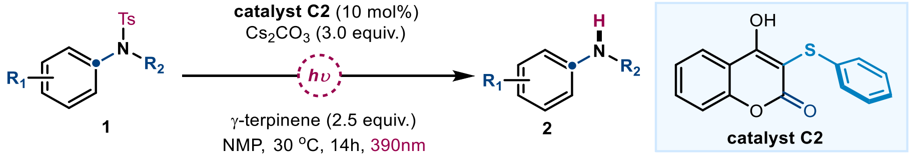

To a 4 mL glass vial, catalyst **C2** (5.4 mg, 0.02 mmol, 0.1 equiv.) or **C5** (6.4 mg, 0,02 mmol, 0.1 equiv. where specified), and Cs_2CO_3 (0.6 mmol, 3 equiv.) were sequentially added. The vial was sealed with a screw-top cap with a septum, evacuated, and backfilled with nitrogen three times. Then, *N*-Ts-amine **1** (0.2 mmol, 1 equiv.), γ-terpinene (80 µL, 0.5 mmol, 2.5 equiv.), and argon-sparged NMP (0.5 M, 0.4 mL) were added via syringe. The vial was sealed with Parafilm and stirred under 390 nm Kessil lamp irradiation in a PhotoRedOx Box TC™ reactor for 14 hours using ***Set-up 1*** detailed in Figure S4.

After irradiation at 390 nm for 14 hours, the reaction mixture was transferred to an extraction funnel. Then, 10 mL of water and 2 mL of brine were added, and the organic layer was extracted

with ethyl acetate (EtOAc x 3). The organic layer was washed twice with brine. The combined organic layers were dried over anhydrous Na_2SO_4, filtered, and concentrated to dryness. The crude residue was purified by column chromatography to afford the corresponding product **2**.

Characterization of Products

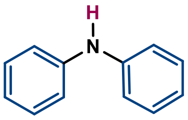

2i

Diphenylamine (2i): Synthesized according to the General Procedure using catalyst **C2** and *N*,*N*-diphenyl-p-toluenesulfonamide (64.7 mg, 0.2 mmol, 1.0 equiv.). The crude mixture was purified by flash column chromatography on silica gel (20% ethyl acetate in hexanes as eluent) to afford **2j** (30.5 mg, 90% yield) as a white solid.

^{1}H NMR (600 MHz, $CDCl_3$) δ 7.34 (q, *J* = 6.4 Hz, 4H), 7.15 (d, *J* = 5.1 Hz, 4H), 7.02 (t, *J* = 7.4 Hz, 2H), 5.73 (s, 1H). **^{13}C NMR (151 MHz, $CDCl_3$)** δ 143.2, 129.4, 121.1, 117.9.
Matching reported literature data.[2]

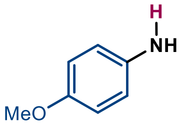

2j

4-Methoxy-aniline (2j): Synthesized according to the General Procedure using catalyst **C5** and *N*-(4-Methoxy-phenyl)-4-methyl-benzenesulfonamide (55.5 mg, 0.2 mmol, 1.0 equiv.). The crude mixture was purified by flash column chromatography on silica gel (5% ethyl acetate in hexanes as eluent) to afford **2k** (17.0 mg, 69% yield) as a brown solid.

^{1}H NMR (600 MHz, $CDCl_3$) δ 6.76 (d, *J* = 9.6 Hz, 2H), 6.65 (d, *J* = 7.5 Hz, 2H), 3.75 (s, 3H), 3.43 (s, 2H). **^{13}C NMR (151 MHz, $CDCl_3$)** δ 152.8, 140.1, 116.5, 114.9, 55.8.
Matching reported literature data.[13]

E.4. Borylation of Aryl Chlorides

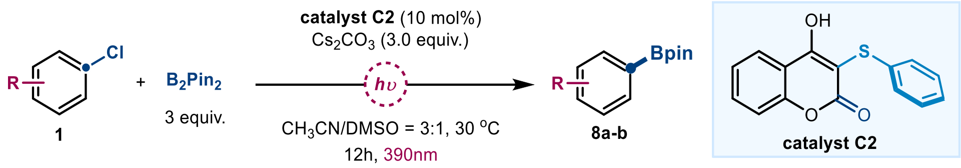

To a 4 mL glass vial, catalyst **C2** (5.4 mg, 0.02 mmol, 0.1 equiv.), cesium carbonate (195 mg, 0.6 mmol, 3 equiv.), B_2Pin_2 (152 mg, 0.6 mmol, 3 equiv.) and aryl chlorides **1** (*if solid*, 0.2 mmol, 1 equiv.) were added as solids. The vial was then evacuated and backfilled with argon three times. Next, aryl chlorides **1** (*if liquid*, 0.2 mmol, 1 equiv.) were added, followed by argon-sparged solvent (CH_3CN:DMSO = 3:1, 0.2 M) via syringe. The vial was sealed with Parafilm and stirred under irradiation from a 390 nm Kessil lamp using the PhotoRedOx Box TC™ reactor for 12 hours (***Set-up 1***, detailed in Figure S4**)**.
After irradiation at 390 nm for 12 hours, the reaction mixture was transferred to an extraction funnel. Then, 10 mL of water and 2 mL of brine were added, and the organic layer was extracted with ethyl acetate (EtOAc). The organic layer was washed twice with brine. The combined organic layers were dried over anhydrous Na_2SO_4, filtered, and concentrated to dryness. The crude residue was purified by column chromatography to afford the corresponding product **8**.

Characterization of Products

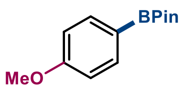

8a

2-(4-methoxyphenyl)-4,4,5,5-tetramethyl-1,3,2-dioxaborolane (8a): Synthesized according to the General Procedure using 1-chloro-4 methoxybenzene (28.5 mg, 0.2 mmol, 1.0 equiv.) and 4,4,4',4',5,5,5',5' octamethyl-2,2'-bi(1,3,2-dioxaborolane) (152.4 mg, 0.6 mmol, 3.0 equiv.). The crude mixture was purified by flash column chromatography on silica gel (3% ethyl acetate in hexanes as eluent) to afford **8a** (30.4 mg, 65% yield) as a white solid.

^{1}H NMR (600 MHz, CDCl$_3$) δ 7.75 (d, J = 8.7 Hz, 2H), 6.89 (d, J = 10.1 Hz, 2H), 3.83 (s, 3H), 1.33 (s, 12H). **^{13}C NMR (151 MHz, CDCl$_3$)** δ 162.1, 136.4, 113.2, 83.4, 55.0, 24.8. Matching reported literature data.[14]

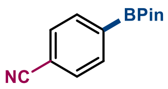

8b

2-(4-cyanophenyl)-4,4,5,5-tetramethyl-1,3,2-dioxaborolane (8b): Synthesized according to the General Procedure using 1-chloro-4-cyanobenzene (27.5 mg, 0.2 mmol, 1.0 equiv.) and 4,4,4',4',5,5,5',5' octamethyl-2,2'-bi(1,3,2-dioxaborolane) (152.4 mg, 0.6 mmol, 3.0 equiv.). The crude mixture was purified by flash column chromatography on silica gel (5% ethyl acetate in hexanes as eluent) to afford **8b** (36.6 mg, 80% yield) as a white solid.

^{1}H NMR (600 MHz, CDCl$_3$) δ 7.86 (d, J = 9.4 Hz, 2H), 7.62 (d, J = 8.4 Hz, 2H), 1.33 (s, 12H).. **^{13}C NMR (151 MHz, CDCl$_3$)** δ 135.2, 131.2, 118.9, 114.6, 84.6, 24.9.

Matching reported literature data.[15]

E.5. General Procedure for the Phosphorylation of Aryl Chlorides

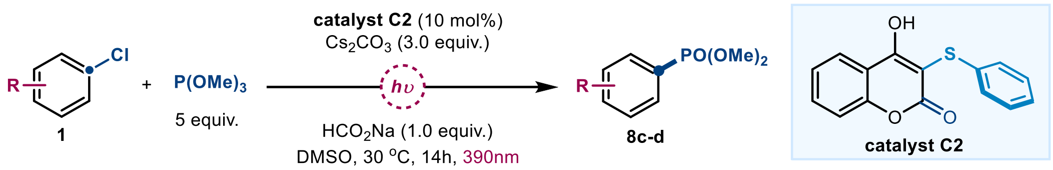

To a 4 mL glass vial, catalyst **C2** (5.4 mg, 0.02 mmol, 0.1 equiv.), cesium carbonate (163 mg, 0.4 mmol, 2 equiv.), sodium formate (13.6 mg, 0.2 mmol, 1 equiv.), and aryl chlorides **1** (*if solid*, 0.2 mmol, 1 equiv.) were added as solids. The vial was then evacuated and backfilled with argon three times. Next, aryl chlorides **1** (*if liquid*, 0.2 mmol, 1 equiv.) and trimethyl phosphite (118 µL, 1.0 mmol, 5.0 equiv.) were added, followed by argon-sparged DMSO (0.5 M, 0.4 mL) via syringe. The vial was sealed with Parafilm and stirred under irradiation from a 390 nm Kessil lamp using the PhotoRedOx Box TC™ reactor for 14 hours (***Set-up 1***, detailed in Figure S4).

After irradiation under 390 nm for 14 hours, the reaction mixture was transferred to an extraction funnel. Then, 10 mL of water and 2 mL of brine were added, and the organic layer was extracted with ethyl acetate (EtOAc). The organic layer was washed twice with brine. The combined organic layers were dried over anhydrous Na_2SO_4, filtered, and concentrated to dryness. The crude residue was purified by column chromatography to afford the corresponding product **8**.

Characterization of Products

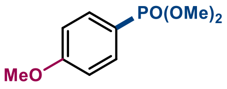

8c

Dimethyl (4-methoxyphenyl)phosphonate (8c): Synthesized according to the General Procedure using 1-chloro-4-methoxybenzene (28.5 mg, 0.2 mmol, 1.0 equiv.) and trimethyl phosphite (124.0 mg, 1.0 mmol, 5.0 equiv.). The crude mixture was purified by flash column chromatography on silica gel (40% acetone in hexanes as eluent) to afford **8c** (23.3 mg, 54% yield) as a yellow liquid.

^{1}H NMR (400 MHz, $CDCl_3$) δ 7.73 (dd, J = 12.7, 8.9 Hz, 2H), 6.97 (dd, J = 8.9, 3.4 Hz, 2H), 3.85 (s, 3H), 3.74 (s, 3H), 3.72 (s, 3H).
^{13}C NMR (151 MHz, $CDCl_3$) δ 163.1, 134.0, 133.9, 118.6, 117.3, 114.2, 114.1, 55.4, 52.6.
^{31}P NMR (243 MHz, $CDCl_3$) δ = 22.77
Matching reported literature data.[11]

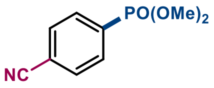

8d

Dimethyl (4-cyanophenyl)phosphonate (8d): Synthesized according to the General Procedure using 1-chloro-4-cyanobenzene (27.5 mg, 0.2 mmol, 1.0 equiv.) and trimethyl phosphite (124.0 mg, 1.0 mmol, 5.0 equiv.). The crude mixture was purified by flash column chromatography on silica gel (40% acetone in hexanes as eluent) to afford **8d** (16.9 mg, 40% yield) as a colorless oil.

^{1}H NMR (400 MHz, $CDCl_3$) δ 7.96 – 7.85 (m, 2H), 7.81 – 7.71 (m, 2H), 3.79 (s, 3H), 3.78 (s, 3H).
^{13}C NMR (151 MHz, $CDCl_3$) δ 133.4, 132.8, 132.7, 132.5, 132.4, 132.2, 118.1, 116.6, 116.6, 53.4, 53.4.
^{31}P NMR (243 MHz, $CDCl_3$) δ = 15.98
Matching reported literature data.[16]

E.6. Radical Trapping by Thiophenols

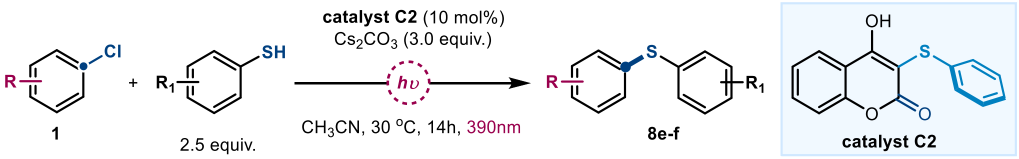

To a 4 mL glass vial, catalyst **C2** (5.4 mg, 0.02 mmol, 0.1 equiv.), cesium carbonate or sodium tert-butoxide (0.6 mmol, 3 equiv.) and aryl chlorides **1** (*if solid*, 0.2 mmol, 1 equiv.) were added as solids. The vial was then evacuated and backfilled with argon three times. Next, aryl chlorides **1** (*if liquid*, 0.2 mmol, 1 equiv.) and thiophenols (0.5 mmol, 2.5 equiv.) were added, followed by argon-sparged solvent (CH_3CN, 0.2 M) via syringe. The vial was sealed with Parafilm and stirred under irradiation from a 390 nm Kessil lamp using the PhotoRedOx Box TC™ reactor (***Set-up 1***, detailed in Figure S4).
After irradiation under 390 nm for 14 hours, the reaction mixture was transferred to an extraction funnel. Then, 10 mL of water and 2 mL of brine were added, and the organic layer was extracted with ethyl acetate (EtOAc). The organic layer was washed twice with brine. The combined organic layers were dried over anhydrous Na_2SO_4, filtered, and concentrated to

dryness. The crude residue was purified by column chromatography to afford the corresponding product.

Characterization of Products

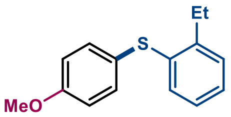

8e

(2-Ethylphenyl)(4-methoxyphenyl)sulfane (8e): Synthesized according to the General Procedure using 1-chloro-4 methoxybenzene (28.5 mg, 0.2 mmol, 1.0 equiv.) and 2-ethylbenzenethiol (54 uL, 0.4 mmol, 2.0 equiv.). The crude mixture was purified by flash column chromatography on silica gel (1% ethyl acetate in hexanes as eluent) to afford **8e** (19.5 mg, 40% yield) as a colorless oil.

^{1}H NMR (600 MHz, CDCl$_3$) δ 7.33 (d, J = 8.8 Hz, 2H), 7.21 (dd, J = 7.6, 1.5 Hz, 1H), 7.14 (td, J = 7.4, 1.4 Hz, 1H), 7.06 (td, J = 7.5, 1.6 Hz, 1H), 7.00 (dd, J = 7.8, 1.4 Hz, 1H), 6.88 (d, J = 8.8 Hz, 2H), 3.82 (s, 3H), 2.80 (q, J = 7.5 Hz, 2H), 1.25 (t, J = 7.5 Hz, 3H).
^{13}C NMR (151 MHz, CDCl$_3$) δ 159.6, 143.2, 136.6, 134.6, 129.8, 128.6, 126.6, 126.5, 125.2, 115.1, 55.5, 27.0, 14.6. Matching reported literature data.[17]

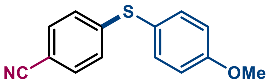

8f

4-((4-methoxyphenyl)thio)benzonitrile (8f): Synthesized according to the General Procedure using 1-chloro-4-cyanobenzene (27.5 mg, 0.2 mmol, 1.0 equiv.) and 4-methoxybenzenethiol (62 uL, 0.5 mmol, 2.5 equiv.). The crude mixture was purified by flash column chromatography on silica gel (1% ethyl acetate in hexanes as eluent) to afford **8f** (24.0 mg, 50% yield) as a colorless oil.

^{1}H NMR (600 MHz, CDCl$_3$) δ 7.45 (dd, J = 15.4, 8.8 Hz, 4H), 7.09 – 7.04 (m, 2H), 6.97 (d, J = 8.8 Hz, 2H), 3.86 (s, 3H).
^{13}C NMR (151 MHz, CDCl$_3$) δ 161.1, 147.5, 137.2, 132.4, 126.2, 120.5, 119.1, 115.7, 108.2, 55.6. Matching reported literature data.[18]

E.7. Mono-Defluorinative Radical Addition of Trifluorotoluenes to Olefins

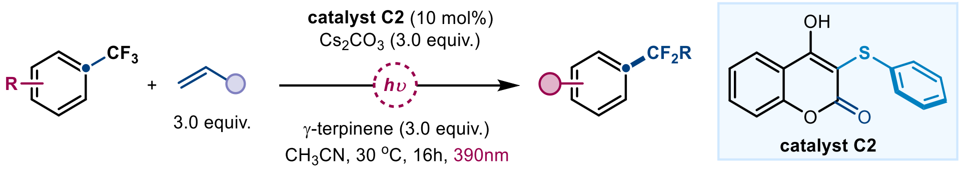

To a 4 mL glass vial, catalyst **C2** (5.4 mg, 0.02 mmol, 0.1 equiv.) and cesium carbonate (163 mg, 0.4 mmol, 2 equiv.) were added as solids. The vial was then evacuated and backfilled with argon three times. Next, 1,3-bis(trifluoromethyl)benzene or *α,α,α*-trifluorotoluene (0.2 mmol, 1 equiv.), *γ*-terpinene (80 μL, 0.5 mmol, 2.5 equiv.) and unactivated alkenes (0.6 mmol, 3 equiv.) were added, followed by argon-sparged CH_3CN (0.1 M) via syringe. The vial was sealed with Parafilm and stirred under irradiation from a 390 nm Kessil lamp using the PhotoRedOx Box TC™ reactor for 16 hours (***Set-up 1***, detailed in Figure S4).
After irradiation under 390 nm for 16 hours, the reaction mixture was transferred to an extraction funnel. Then, 10 mL of water and 2 mL of brine were added, and the organic layer was extracted with ethyl acetate (EtOAc). The organic layer was washed twice with brine. The

combined organic layers were dried over anhydrous Na_2SO_4, filtered, and concentrated to dryness. The crude residue was purified by column chromatography to afford the corresponding product.

Characterization of Products

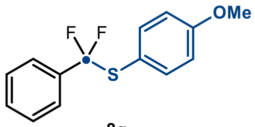

8g

(Difluoro(phenyl)methyl)(4-methoxyphenyl)sulfane (8g): Synthesized according to the General Procedure using α,α,α-trifluorotoluene (29.2 mg, 0.2 mmol, 1.0 equiv.) and 4-methoxybenzenethiol (62 uL, 0.5 mmol, 2.5 equiv.). The crude mixture was purified by flash column chromatography on silica gel (2% ethyl acetate in hexanes as eluent) to afford **8g** (44.7 mg, 84% yield) as a white solid.

^{1}H NMR (600 MHz, $CDCl_3$) δ 7.58 – 7.50 (m, 4H), 7.47 – 7.36 (m, 3H), 6.92 – 6.87 (m, 2H), 3.83 (s, 3H). **^{19}F NMR (376 MHz, $CDCl_3$)** δ -72.8.
Matching reported literature data.[19]

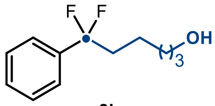

8h

6,6-difluoro-6-phenylhexan-1-ol (8h): Synthesized according to the General Procedure using α,α,α-trifluorotoluene (29.2 mg, 0.2 mmol, 1.0 equiv.) and 4-Penten-1-ol (51.7 mg, 0.6 mmol, 2.0 equiv.). The crude mixture was purified by flash column chromatography on silica gel (20% ethyl acetate in hexanes as eluent) to afford **8h** (37.7 mg, 88% yield) as a colorless oil.

^{1}H NMR (400 MHz, $CDCl_3$) δ 7.51 – 7.36 (m, 5H), 3.61 (t, J = 6.5 Hz, 2H), 2.21 – 2.05 (m, 2H), 1.60 – 1.51 (m, 2H), 1.47 – 1.38 (m, 4H). **^{19}F NMR (376 MHz, $CDCl_3$)** δ -95.5 (t, J = 16.3 Hz). Matching reported literature data.[20]

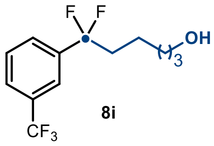

8i

6,6-difluoro-6-phenylhexan-1-ol (8i): Synthesized according to the General Procedure using 1,3-bis(trifluoromethyl)benzene (42.8 mg, 0.2 mmol, 1.0 equiv.) and 4-Penten-1-ol (51.7 mg, 0.6 mmol, 2.0 equiv.). The crude mixture was purified by flash column chromatography on silica gel (20% ethyl acetate in hexanes as eluent) to afford **8i** (46.2 mg, 82% yield) as a colorless oil.

^{1}H NMR (400 MHz, $CDCl_3$) δ 7.80 – 7.71 (m, 1H), 7.71 – 7.62 (m, 2H), 7.56 (t, J = 7.8 Hz, 1H), 3.62 (t, J = 6.5 Hz, 2H), 2.22 – 2.06 (m, 2H), 1.66 – 1.52 (m, 2H), 1.49 – 1.33 (m, 4H). **^{19}F NMR (376 MHz, $CDCl_3$)** δ -62.8, -96.1 (t, J = 16.5 Hz). Matching reported literature data.[21]

E.8. Birch Reduction

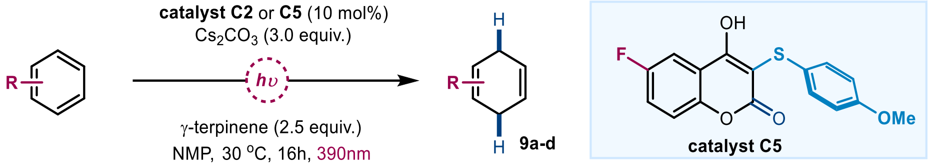

To a 4 mL glass vial, catalyst **C2** (5.4 mg, 0.02 mmol, 0.1 equiv.) or **C5** (6.4 mg, 0,02 mmol, 0.1 equiv. where specified), Cs_2CO_3 (0.4 mmol, 2 equiv.) and arenes (*if solid*, 0.2 mmol, 1 equiv.) were sequentially added. The vial was sealed with a screw-top cap with the septum and then evacuated and backfilled with argon three times. Next, arenes **4** (*if liquid*, 0.2 mmol, 1 equiv.) and γ-terpinene (80 µL, 0.5 mmol, 2.5 equiv.) were added, followed by argon-sparged NMP (0.5 M, 0.4 mL) via syringe. The vial was sealed with Parafilm and stirred under the irradiation of a 390 nm Kessil lamp in the PhotoRedOx Box TC™ reactor for 16 hours using ***Set-up 2***, as detailed in Figure S5.

NMR analyses (products **9a, 9b, 9d**): dibromomethane was added as the internal standard (34.8 mg, 0.2 mmol, 1 equiv.) to the crude reaction mixture, followed by 2 mL H_2O and 1 mL of brine. Then, the solution was extracted with 0.7 mL of $CDCl_3$ and the reactions were analyzed via ^1H NMR spectroscopy of the $CDCl_3$ layer.

For the isolation of product **9c**: After irradiation under 390 nm for 16 hours, the reaction mixture was transferred to an extraction funnel, 10 mL of H_2O and 2 mL of brine were added and the organic layer was extracted with EtOAc. The organic layer was washed twice with brine, the combined organic layers were dried over anhydrous Na_2SO_4, filtered, and concentrated to dryness. The crude residue was purified by column chromatography to afford the corresponding products with the reported yields (>95% purity according to ^1H NMR analysis).

The crude ^1H NMR of products **9a, 9b,** and **9d**, which are reported in the NMR Section I of the Supporting Information, matched the reported literature data.[11, 22, 23]

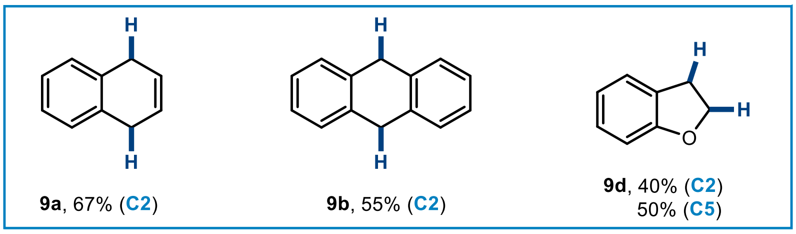

Figure S8: Compounds for which yields have been determined by ^1H NMR analysis using an internal standard.

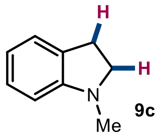

1-Methylindoline (9c): Synthesized according to the General Procedure using 1-methyl-1H-indole (26.2 mg, 0.2 mmol, 1.0 equiv.) and **C5** (6.4 mg, 0.02 mmol, 10 mol%). The crude mixture was purified by flash column chromatography on silica gel (4% ethyl acetate in hexanes as eluent) to afford **9c** (17.0 mg, 64% yield) as an orange oil. **^1H NMR (400 MHz, $CDCl_3$)** δ 7.16 – 7.00 (m, 2H), 6.75 – 6.61 (m, 1H), 6.49 (d, J = 7.0 Hz, 1H), 3.29 (d, J = 15.9 Hz, 2H), 2.95 (t, J = 8.5 Hz, 2H), 2.76 (s, 3H). **^{13}C NMR (151 MHz, $CDCl_3$)** δ 153.7, 130.7, 127.7, 124.6, 118.1, 107.6, 56.5, 36.6, 29.1. Matching reported literature data.[24]

E.9. Reductive Radical Cyclization

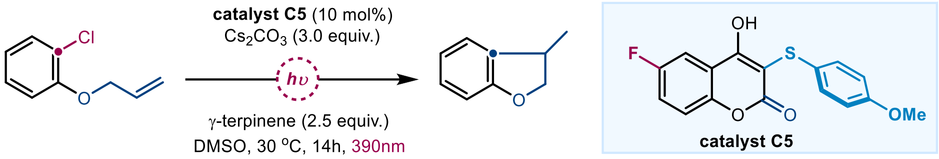

To a 4 mL glass vial, catalyst **C5** (6.4 mg, 0.02 mmol, 0.1 equiv.) and caesium carbonate (130.4 mg, 0.4 mmol, 2 equiv.) were added as solids. The vial was then evacuated and backfilled with argon three times. Next, allyl 2-chlorophenyl ether (33.8 mg, 0.2 mmol, 1 equiv.) and *γ*-terpinene (64.2 µL, 0.4 mmol, 2.0 equiv.) were added, followed by argon-sparged DMSO (1 mL) via syringe. The vial was sealed with Parafilm and then stirred under the irradiation of 390 nm *Kessil* lamp with PhotoRedOx Box TC™ reactor for 14 hours using ***Set-up 1*** detailed in Figure S4.

After irradiation under 390 nm for 14 hours, the reaction mixture was transferred to an extraction funnel. Then, 10 mL of water and 2 mL of brine were added, and the organic layer was extracted with ethyl acetate (EtOAc). The organic layer was washed twice with brine. The combined organic layers were dried over anhydrous Na_2SO_4, filtered, and concentrated to dryness). The crude mixture was purified by flash column chromatography on silica gel (1% ethyl acetate in hexanes as eluent) to afford **11a** (16.1 mg, 60% yield) as a colorless oil.

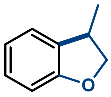

11a

3-methyl-2,3-dihydrobenzofuran (11a)

^{1}H NMR (600 MHz, $CDCl_3$) δ 7.16 (d, *J* = 8.7 Hz, 1H), 7.12 (t, *J* = 7.3 Hz, 1H), 6.87 (t, *J* = 7.3 Hz, 1H), 6.79 (d, *J* = 8.1 Hz, 1H), 4.68 (t, *J* = 8.7 Hz, 1H), 4.12 – 4.01 (m, 1H), 3.55 (h, *J* = 6.8 Hz, 1H), 1.33 (d, *J* = 6.9 Hz, 3H).

Matching reported literature data.[25]

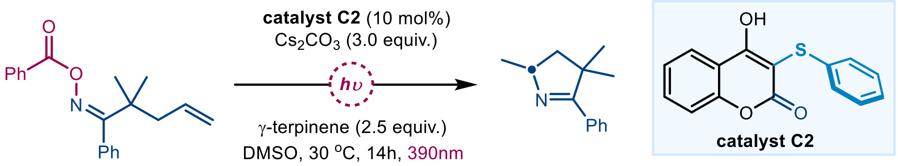

To a 4 mL glass vial, catalyst **C2** (2.7 mg, 0.01 mmol, 0.1 equiv.), γ,δ-unsaturated *O*-benzoyl ketoxime (30.7 mg, 0.1 mmol, 1.0 equiv.), and cesium carbonate (32.6 mg, 0.1 mmol, 1.0 equiv.) were added as solids. The vial was then evacuated and backfilled with argon three times. Next, γ-terpinene (32.1 µL, 0.2 mmol, 2.0 equiv.) was added, followed by argon-sparged DMSO (0.1 M, 0.4 mL) via syringe. The vial was sealed with Parafilm and stirred under the irradiation of a 390 nm Kessil lamp in the PhotoRedOx Box TC™ reactor for 14 hours using ***Set-up 1***, as detailed in Figure S4.

After irradiation under 390 nm for 14 hours, the reaction mixture was transferred to an extraction funnel. Then, 10 mL of water and 2 mL of brine were added, and the organic layer was extracted with ethyl acetate (EtOAc). The organic layer was washed twice with brine. The combined organic layers were dried over anhydrous Na_2SO_4, filtered, and concentrated to dryness. The crude mixture was purified by flash column chromatography on silica gel (20% ethyl acetate in hexanes as eluent) to afford **11b** (12.2 mg, 65% yield) as a yellow oil.

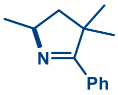

11b

2,4,4-trimethyl-5-phenyl-3,4-dihydro-2H-pyrrole (11b)

^{1}H NMR (600 MHz, $CDCl_3$) δ 7.73 – 7.63 (m, 2H), 7.37 (dd, J = 5.2, 2.0 Hz, 3H), 4.17 – 4.01 (m, 1H), 2.11 (dd, J = 12.4, 6.7 Hz, 1H), 1.50 (dd, J = 12.4, 8.6 Hz, 1H), 1.38 (d, J = 6.8 Hz, 3H), 1.35 (s, 3H), 1.33 (s, 3H).

Matching reported literature data.[26]

E.10. Difunctionalization of Alkenes

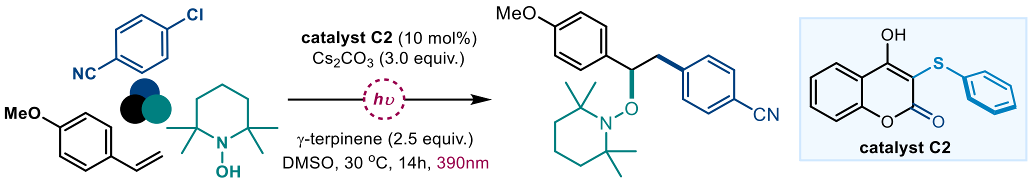

To a 4 mL glass vial, catalyst **C2** (5.5 mg, 0.02 mmol, 0.1 equiv.), TEMPO-H (38 mg, 0.24 mmol, 1.2 equiv.) and caesium carbonate (163 mg, 0.6 mmol, 3 equiv.) were added as solids. The vial was then evacuated and backfilled with argon three times. Next, 4-chlorobenzonitrile (27.5 mg, 0.2 mmol, 1 equiv.) and 4-methoxystyrene (40 μL, 0.3 mmol, 1.5 equiv.) were added, followed by argon-sparged DMSO (1 mL) via syringe. The vial was sealed with Parafilm and stirred under the irradiation of a 390 nm Kessil lamp in the PhotoRedOx Box TC™ reactor for 14 hours using ***Set-up 1***, as detailed in Figure S6.

After irradiation under 390 nm for 14 hours, the reaction mixture was transferred to an extraction funnel. Then, 10 mL of water and 2 mL of brine were added, and the organic layer was extracted with ethyl acetate (EtOAc). The organic layer was washed twice with brine. The combined organic layers were dried over anhydrous Na_2SO_4, filtered, and concentrated to dryness. The crude residue was purified by column chromatography to afford the corresponding product **12** (31 mg, 40% yield) as a white solid.

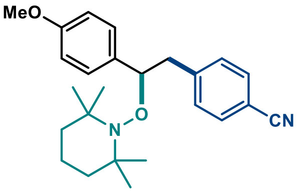

12

4-(2-(4-Methoxyphenyl)-2-((2,2,6,6-tetramethylpiperidin-1-yl)oxy)ethyl)benzonitrile (12)

^{1}H NMR (600 MHz, $CDCl_3$) δ 7.41 (d, *J* = 8.3 Hz, 2H), 6.98 (dd, *J* = 8.4, 3.7 Hz, 4H), 6.74 (d, *J* = 8.6 Hz, 2H), 4.74 (dd, *J* = 9.8, 4.7 Hz, 1H), 3.77 (s, 3H), 3.63 (dd, *J* = 12.7, 4.7 Hz, 1H), 2.95 (dd, *J* = 12.7, 9.8 Hz, 1H), 1.54 – 1.25 (m, 12H), 1.19 – 1.00 (m, 6H). **^{13}C NMR (151 MHz, $CDCl_3$)** δ 159.0, 144.7, 134.1, 131.8, 130.6, 129.1, 119.3, 113.2, 109.8, 87.4, 60.2, 59.9, 55.2, 43.2, 40.5, 29.8, 20.5, 17.3.

Matching reported literature data.[27]

E.11 Energy Transfer Catalysis

E11.1 General Procedure for the *E*/*Z* Photoisomerization

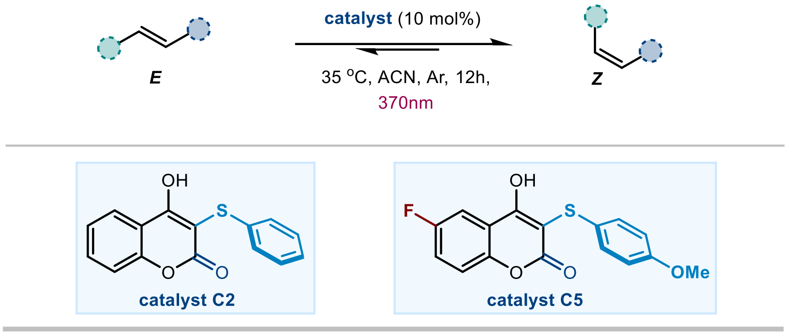

To a 10 mL Schlenk tube, catalyst (0.02 mmol, 0.1 equiv.) and *E* alkenes (0.2 mmol, 1 equiv.) were sequentially added, followed by argon-sparged CH_3CN (0.2 M, 1 mL) via syringe. The Schlenk tube was sealed, frozen in liquid nitrogen, and evacuated. This process was repeated three times, followed by three cycles of freezing, evacuating, and backfilling with argon. The reaction mixture was then stirred under 370 nm Kessil lamp irradiation for 12 hours, using ***Set-up 3***, as detailed in Figure S6.

After irradiation under 370 nm for 12 hours, the reaction mixture was concentrated to dryness. The crude residue was purified by column chromatography to afford the corresponding product *Z*-**3**.

Characterization of Products

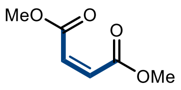

Z-3a

Dimethyl maleate (*Z*-3a): Synthesized according to the General Procedure C using dimethyl fumarate (28.8 mg, 0.2 mmol, 1.0 equiv.) and **C2** (5.4 mg, 0.02 mmol, 10 mol%.). The crude mixture was purified by flash column chromatography on silica gel (20% diethyl ether in hexanes as eluent, $\mathbf{R_f}$ = 0.4) to afford *Z*-**3a** (26.0 mg, 90% yield) as a colorless oil. The ratio of isomers in the crude mixture was determined via ^{1}H NMR analysis - *Z*:*E* = 92:8.

^{1}H NMR (600 MHz, $CDCl_3$) δ 6.29 – 6.20 (m, 2H), 3.84 – 3.70 (m, 6H).
^{13}C NMR (151 MHz, $CDCl_3$) δ 166.0, 130.1, 52.5. Matching reported literature data.[28]

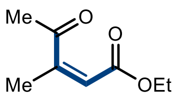

Z-**3b**

Ethyl (*Z*)-3-methyl-4-oxopent-2-enoate (*Z*-3b): Synthesized according to the General Procedure C using Ethyl (*E*)-3-methyl-4-oxohex-2-enoate (31.2 mg, 0.2 mmol, 1.0 equiv.), **C2** (5.4 mg, 0.02 mmol, 10 mol%.). The crude mixture was purified by flash column chromatography on silica gel (10% diethyl ether in hexanes as eluent, $\mathbf{R_f}$ = 0.24) to afford *Z*-**3b** (25.0 mg, 80% yield) as a colorless oil. The ratio of isomers in the crude mixture was determined via ^{1}H NMR - *Z*:*E* = 97:3.

^{1}H NMR (600 MHz, $CDCl_3$) δ 5.68 (q, *J* = 1.6 Hz, 1H), 4.16 (q, *J* = 7.1 Hz, 2H), 2.36 (s, 3H), 1.99 (d, *J* = 1.6 Hz, 3H), 1.26 (t, *J* = 7.2 Hz, 4H).
^{13}C NMR (151 MHz, $CDCl_3$) δ 206.6, 165.5, 157.5, 117.4, 61.1, 29.0, 20.6, 14.4.

Matching reported literature data.[28]

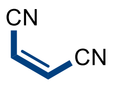

Z-**3c**

Maleonitrile (*Z*-3c): Synthesized according to the General Procedure C using 1,2-Dicyanoethylene (15.6 mg, 0.2 mmol, 1.0 equiv.), **C2** (5.4 mg, 0.02 mmol, 10 mol%). After completion of the reaction, dibromomethane was added as the internal standard (34.8 mg, 0.2 mmol, 1 equiv.) to the crude reaction mixture, the ratio of isomers in the crude mixture was determined via ^{1}H NMR - *Z:E* = 53:47 with a 40% yield. Matching reported literature data.[29]

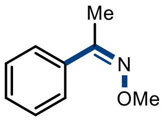

Z-**3d**

(*Z*)-1-phenylethan-1-one *O*-methyl oxime (*Z*-3d): Synthesized according to the General Procedure C using (*E*)-1-phenylethan-1-one O-methyl oxime (30.0 mg, 0.2 mmol, 1.0 equiv.), **C2** (5.4 mg, 0.02 mmol, 10 mol%.), which were stirred under the irradiation of a 390 nm Kessil lamp in the PhotoRedOx Box TC™ reactor for 24 hours using *Set-up 3*, as detailed in Figure S6. After completion, the crude mixture was purified by flash column chromatography on silica gel (10% diethyl ether in hexanes as eluent, $\mathbf{R_f}$ = 0.6) to afford *Z*-**3d** (24.3 mg, 81% yield) as a colorless oil. The ratio of isomers in the crude mixture was determined via ^{1}H NMR - *Z:E* = 91:9.

^{1}H NMR (600 MHz, CDCl$_3$) δ 7.52 – 7.44 (m, 2H), 7.44 – 7.31 (m, 3H), 3.85 (s, 3H), 2.21 (s, 3H). **^{13}C NMR (151 MHz, CDCl$_3$)** δ 154.2, 134.8, 129.3, 128.5, 128.2, 62.1, 22.0.
Matching reported literature data.[30]

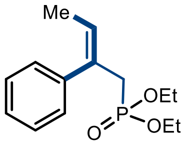

Z-**3e**

Diethyl (*Z*)-(2-phenylbut-1-en-1-yl)phosphonate (*Z*-3e): Synthesized according to the General Procedure C using Diethyl (*E*)-(2-phenylbut-1-en-1-yl)phosphonate (26.8 mg, 0.1 mmol, 1.0 equiv.), **C5** (3.2 mg, 0.01 mmol, 10 mol%.) and Cs_2CO_3 (5.0 mg, 0.015 mmol, 15 mol%.), which were stirred under the irradiation of a 390 nm Kessil lamp in the PhotoRedOx Box TC™ reactor for 24 hours using *Set-up 3*, as detailed in Figure S6. After completion, the crude mixture was purified by flash column chromatography on silica gel (ethyl acetate as eluent, $\mathbf{R_f}$ = 0.26) to afford *Z*-**3e** (18.9 mg, 70% yield) as a colorless oil. The ratio of isomers in the crude mixture was determined via ^{1}H NMR - *Z:E* = 91:9.

^{1}H NMR (600 MHz, CDCl$_3$) δ 7.37 – 7.29 (m, 2H), 7.26 – 7.21 (m, 3H), 5.81 (s, 1H), 4.02 – 3.83 (m, 4H), 2.91 (d, J = 21.4 Hz, 2H), 1.64 (t, J = 6.5 Hz, 3H), 1.17 (t, J = 7.1 Hz, 6H).
^{13}C NMR (151 MHz, CDCl$_3$) δ 140.4 (d, *J* = 3.8 Hz), 131.5 (d, *J* = 10.6 Hz), 129.1, 128.4, 127.3 (d, *J* = 12.0 Hz), 127.2, 62.0 (d, *J* = 6.8 Hz), 36.3 (d, *J* = 138.3 Hz), 16.6 (d, *J* = 6.2 Hz), 15.5 (d, *J* = 3.0 Hz).
^{31}P NMR (243 MHz, CDCl$_3$) δ 27.33 (ddp, *J* = 21.4, 14.6, 6.9 Hz).
HRMS: calculated for $C_{14}H_{22}O_3P$ (M+H$^+$): 269.1301, found 269.1308.

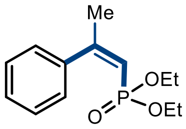

Z-**3f**

Diethyl (*Z*)-(2-phenylprop-1-en-1-yl)phosphonate (*Z*-3f): Synthesized according to the General Procedure C using diethyl (*E*)-(2-phenylprop-1-en-1-yl)phosphonate (50.9 mg, 0.2 mmol, 1.0 equiv.), **C5** (6.4 mg, 0.02 mmol, 10 mol%), which were stirred under the irradiation of a 370 nm Kessil lamp with a fan for 22 hours using *Set-up 3*, as detailed in Figure S6. After

completion, the crude mixture was purified by flash column chromatography on silica gel (30% ethyl acetate in hexanes as eluent, **R$_f$** = 0.3) to afford *Z*-**3f** (45.3 mg, 89% yield) as a colorless oil. The ratio of isomers in the crude mixture was determined via ^{1}H NMR - *Z:E* = 93:7.

^{1}H NMR (600 MHz, CDCl$_3$) δ 7.41 – 7.37 (m, 2H), 7.37 – 7.29 (m, 3H), 5.73 (dd, *J* = 17.3, 1.5 Hz, 1H), 3.94 – 3.66 (m, 4H), 2.23 (s, 3H), 1.07 (t, *J* = 7.0 Hz, 6H).
^{13}C NMR (151 MHz, CDCl$_3$) δ 159.3, 159.3, 140.9, 140.8, 128.6, 128.3, 127.7, 127.6, 115.8, 114.5, 61.7, 61.6, 28.8, 28.7, 16.4.
^{31}P NMR (243 MHz, CDCl$_3$) δ = 16.20 (dp, *J* = 15.8, 7.7 Hz).
HRMS: calculated for $C_{13}H_{19}NaO_3P$ (M+Na$^+$): 277.0964, found 277.0966.
Matching reported literature data.[4]

E.11.2 General Procedure D for the Intermolecular [2+2] Photocycloadditions

To a 10 mL Schlenk tube, catalyst **C** (0.02 mmol, 0.1 equiv.), the EnT-responsive substrate (0.2 mmol, 1 equiv.), and an alkene were sequentially added, followed by argon-sparged solvent (0.2 M, 1 mL) via syringe. The Schlenk tube was sealed, frozen in liquid nitrogen, and evacuated. This process was repeated three times, followed by three cycles of freezing, evacuating, and backfilling with argon. The reaction mixture was then stirred under irradiation of 370 nm *Kessil* lamp with a fan for 22 hours using ***Set-up 3*** detailed in Figure S6.
After the reaction was completed, the reaction mixture was transferred to an extraction funnel, 10 mL of H_2O and 2 mL of brine were added and the organic layer was extracted with EtOAc. The organic layer was washed twice with brine, the combined organic layers were dried over anhydrous Na_2SO_4, filtered, and concentrated to dryness. The crude residue was purified by column chromatography to afford the corresponding products with the reported yields.

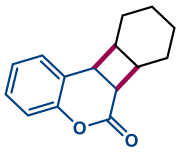

4

6a,6b,7,8,9,10,10a,10b-Octahydro-benzo(3,4)cyclobuta[1,2-c]chromen-6-on (4): Synthesized according to the General Procedure D using coumarin (30.0 mg, 0.2 mmol, 1.0 equiv.), cyclohexene (49.3 mg, 0.6 mmol, 3 equiv.), and catalyst **C2** (5.4 mg, 0.02 mmol, 10 mol%.), dry CH_3CN (1 mL). The crude mixture was purified by flash column chromatography on silica gel (10% diethyl ether in hexanes as eluent) to afford **4** (27.5 mg, 60% yield) as a colorless oil. The ratio of isomers in the crude mixture was determined via ^{1}H NMR analysis to be d.r. = 17:7:1 (see section I for details).

^{1}H NMR (600 MHz, CDCl$_3$) δ 7.18 – 7.14 (m, 1H), 7.03 – 6.99 (m, 2H), 6.97 – 6.94 (m, 1H), 3.77 (t, *J* = 8.9 Hz, 1H), 3.51 – 3.43 (m, 1H), 3.04 – 2.92 (m, 1H), 2.80 – 2.73 (m, 1H), 1.67 – 1.54 (m, 2H), 1.38 – 0.98 (m, 6H).
^{13}C NMR (151 MHz, CDCl$_3$) δ 168.4, 152.4, 128.9, 128.5, 124.7, 121.4, 117.5, 38.1, 38.0, 36.8, 36.6, 24.0, 23.9, 22.2, 21.9.

Matching reported literature data.[31]

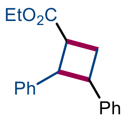

5

Ethyl 2,3-diphenylcyclobutane-1-carboxylate (5): Synthesized according to the General Procedure D using ethyl cinnamate (32.4 mg, 0.2 mmol, 1.0 equiv.), styrene (104.1mg, 1 mmol, 5 equiv.) and catalyst **C3** (5.8 mg, 0.02 mmol, 10 mol%.), dry DCM (1 mL). The crude mixture was purified by flash column

chromatography on silica gel (4% ethyl acetate in hexanes as eluent) to afford **5** (27.8 mg, 50% yield) as a colorless oil. The ratio of isomers in the crude mixture was determined via ^{1}H NMR analysis to be d.r. = 3:1.

^{1}H NMR (600 MHz, $CDCl_3$) δ 7.26 – 7.19 (m, 8H), 7.15 (t, *J* = 7.1 Hz, 2H), 4.15 – 4.07 (m, 2H), 3.78 (t, *J* = 9.8 Hz, 1H), 3.51 – 3.41 (m, 1H), 3.15 – 3.04 (m, 1H), 2.60 – 2.48 (m, 1H), 2.33 (q, *J* = 10.4 Hz, 1H), 1.21 (t, *J* = 7.1 Hz, 4H).
^{13}C NMR (151 MHz, $CDCl_3$) δ 174.5, 143.6, 142.6, 128.8, 127.2, 127.0, 127.0, 126.9, 60.9, 51.0, 43.6, 42.1, 30.1, 29.9, 14.6. Matching reported literature data.[32]

General Procedure E for the Intramolecular [2+2] Photocycloadditions
To a 10 mL Schlenk tube, catalyst **C2** (5.4 mg, 0.02 mmol, 10 mol%.) and the substrate (0.2 mmol, 1 equiv.) were sequentially added, followed by argon-sparged solvent (0.2 M, 1 mL) via syringe. The Schlenk tube was sealed, frozen in liquid nitrogen, and evacuated. This process was repeated three times, followed by three cycles of freezing, evacuating, and backfilling with argon. The reaction mixture was then stirred under irradiation of 370 nm *Kessil* lamp with a fan for 22 hours using ***Set-up 3*** detailed in Figure S6.
After the reaction was completed, the reaction mixture was transferred to an extraction funnel, 10 mL of H_2O and 2 mL of brine were added, and the organic layer was extracted with EtOAc. The organic layer was washed twice with brine, the combined organic layers were dried over anhydrous Na_2SO_4, filtered, and concentrated to dryness. The crude residue was purified by column chromatography to afford the corresponding products with the reported yields.

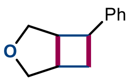

6

6-Phenyl-3-oxa-bicyclo[3.2.0]heptane (6): Synthesized according to the General Procedure E using (*E*)-(3-(allyloxy)prop-1-en-1-yl)benzene (34.8 mg, 0.2 mmol, 1.0 equiv.) and catalyst **C2** (5.4 mg, 0.02 mmol, 10 mol%.), dry DMSO (1mL). The mixture was purified by flash column chromatography on silica gel (10% diethyl ether in hexanes as eluent) to afford **6** (20.2 mg, 58% yield) as a colorless oil. The ratio of isomers in the crude mixture was determined via ^{1}H NMR analysis to be d.r. = 5:1.

^{1}H NMR (600 MHz, $CDCl_3$) δ 7.35 – 7.28 (m, 2H), 7.25 (d, *J* = 6.0 Hz, 2H), 7.22 – 7.16 (m, 1H), 4.03 – 3.94 (m, 2H), 3.61 (dd, *J* = 8.9, 6.0 Hz, 1H), 3.51 (dd, *J* = 9.3, 4.1 Hz, 1H), 3.23 (dd, *J* = 12.4, 9.6 Hz, 1H), 3.05 – 2.90 (m, 2H), 2.35 – 2.24 (m, 1H), 2.21 – 2.12 (m, 1H).
^{13}C NMR (151 MHz, $CDCl_3$) δ 146.2, 128.6, 126.5, 126.1, 74.6, 74.1, 47.3, 42.1, 35.4, 31.9. Matching reported literature data.[33]

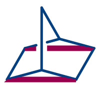
7

Quadricyclo[2.2.1.0.0]heptane (7): Synthesized according to the General Procedure E using bicyclo[2.2.1]hepta-2,5-diene (18.4 mg, 0.2 mmol, 1.0 equiv.) and catalyst **C2** (5.4 mg, 0.02 mmol, 10 mol%.) using CD_3CN as solvent. After completion of the reaction, dibromomethane was added as the internal standard (34.8 mg, 0.2 mmol, 1 equiv.) to the crude reaction mixture. The yield of **7** (35% yield) was determined via ^{1}H NMR analysis of the crude mixture. Characterization data matching reported literature data.[34]

F. Mechanistic Studies

F1. Quantum Yield Determination

-Experimental Setup

The experiments for the quantum yield determination were conducted under illumination by a 390 Kessil lamp (setup depicted in Figure S9), using a 3D printed photoreactor. The intensity was fixed to the maximum value.

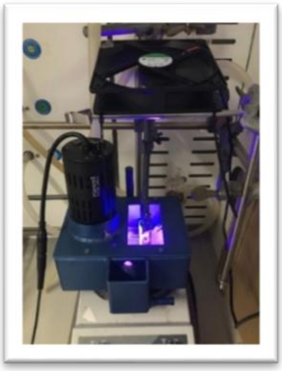

Figure S9: Kessil lamp set-up for the quantum yield determination

General Procedure for photon Flux (F) determination:[35]

$$(1) \qquad \mathbf{\Phi = M / (F \bullet t \bullet f)}$$

$$(2) \qquad \mathbf{f = 1\text{-}10^{-A}}$$

M is the moles of product formed (mol), **F** is the number of photons emitted per second (einstein s−1), **t** is the time (s) and **f** is fraction of light absorbed which can be calculated using eq. 2, where **A** is the measured absorbance at 390 nm.

The number of photons emitted per second was determined using azobenzene as actinometer.[36,39] Using the reaction setup depicted in Figure S9, a glass vial was filled with a solution of *Z*-azobenzene (0.1 mmol) in CD_3OD (0.1M) and irradiated at 390 nm. The *trans-cis* isomerization was monitored by ^{1}H NMR analysis using dibromomethane as internal standard (Figure S10).

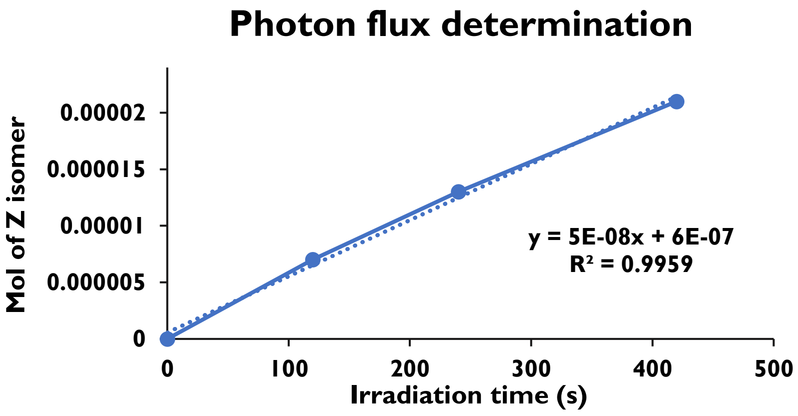

Figure S10. Plot of moles of *cis*-azobenzene formed vs irradiation time (s)

The actinometer solution was irradiated for 0 min, 2 min, 4 min, and 7 min. According to the equations (**1**) and (**2**), and an estimated quantum yield of *trans-cis* isomerization process at 390 nm (Φ = 0.262), the number of photons emitted per time unit (**F**) was determined (2.08 x 10^{-8} mol s^{-1}).

Quantum Yield Determination:
Following the general procedure, three model dechlorination reactions using 4-chlorophenylboronic acid pinacol ester **1d**, catalyst **C2**, γ-terpinene and Cs_2CO_3, leading to product **2d**, were performed separately. Each reaction mixture was irradiated for 0 h, 2 h, 4 h, 6 h, and 8 h. After irradiation, the reactions were worked up and the amount of product **2f** formed was determined by ^{1}H NMR analysis of the crude using mesitylene as the internal standard. Fraction of light absorbed *f* was recognized as 1. The moles of the formed product **2f** are plotted against the number of incident photons (Figure S11). The quantum yield was calculated to be Φ = 0.13 based on the slope and eq. (**1**), (**2**).

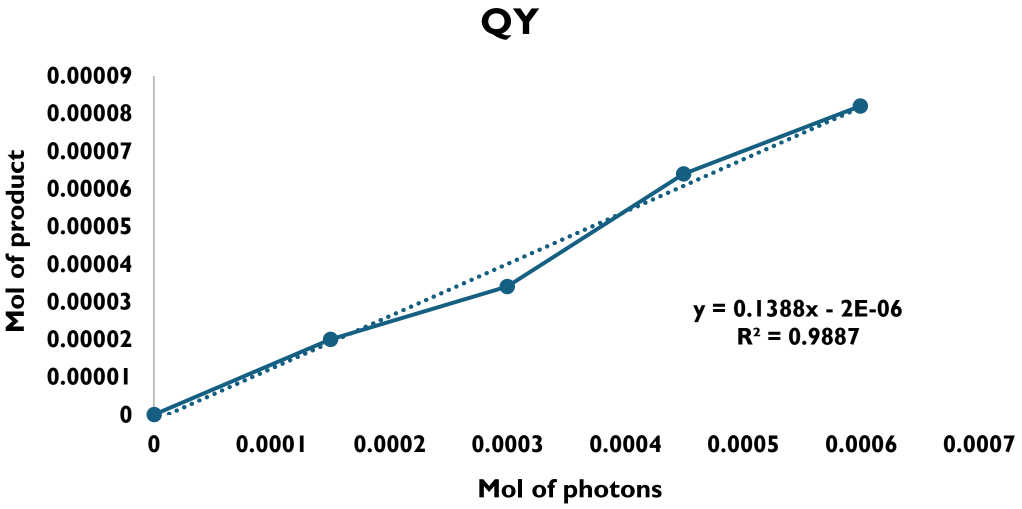

Figure S11: Plot of moles of incident photons vs moles of product **2f** formed.

F2 Photophysical Characterization of the Photocatalysts C

Sample Preparation: Eight 25 mL glass vials, each containing either catalyst **C2**, **C3**, **C4**, or **C5**, or their mixture with Cs_2CO_3 (3 equiv.) as the base, were sealed with septa and degassed. Then, dry, degassed CH_3CN (20 mL) was added to each vial to prepare 0.1 M solutions of the catalysts. From these stock solutions, **C2** to **C5** were prepared at concentrations of 10^{-3} M and 10^{-4} M, and similarly, **C2** to **C5** with Cs_2CO_3 (3 equivalents) were prepared at the same concentrations. Then, 2.5 mL of each solution was transferred into an argon-filled quartz cuvette (10 x 10 mm light path) equipped with a septum.

F2.1 UV-Vis Absorption Studies

UV-Vis measurements were carried out on an Cary 3500 Multicell UV-Vis spectrophotometer equipped with two silicon diode detectors, double beam optics and Xenon pulse light (Figure S12-S14).

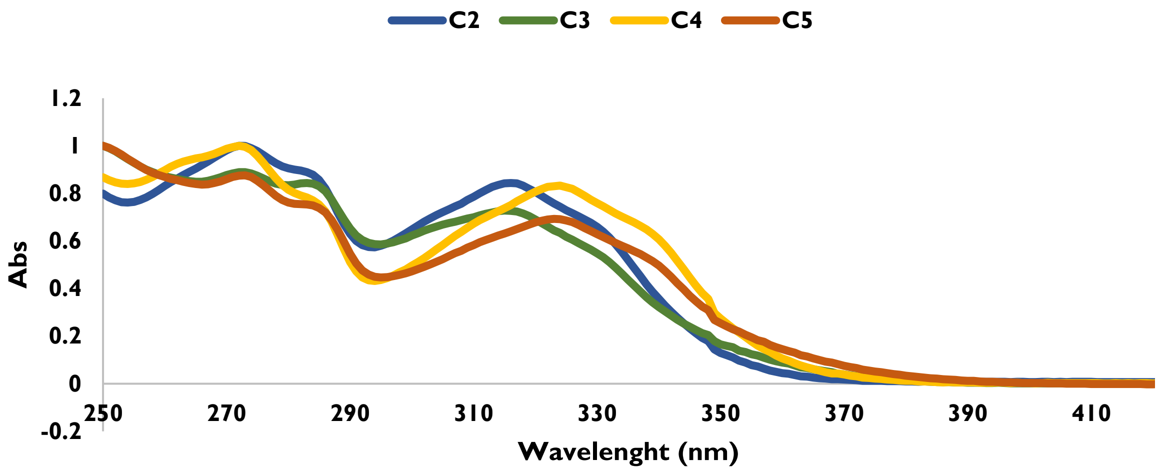

Figure S12 – Absorption profiles of 10^{-4} M solutions of catalysts **C2-C5** in dry, degassed acetonitrile at 298K.

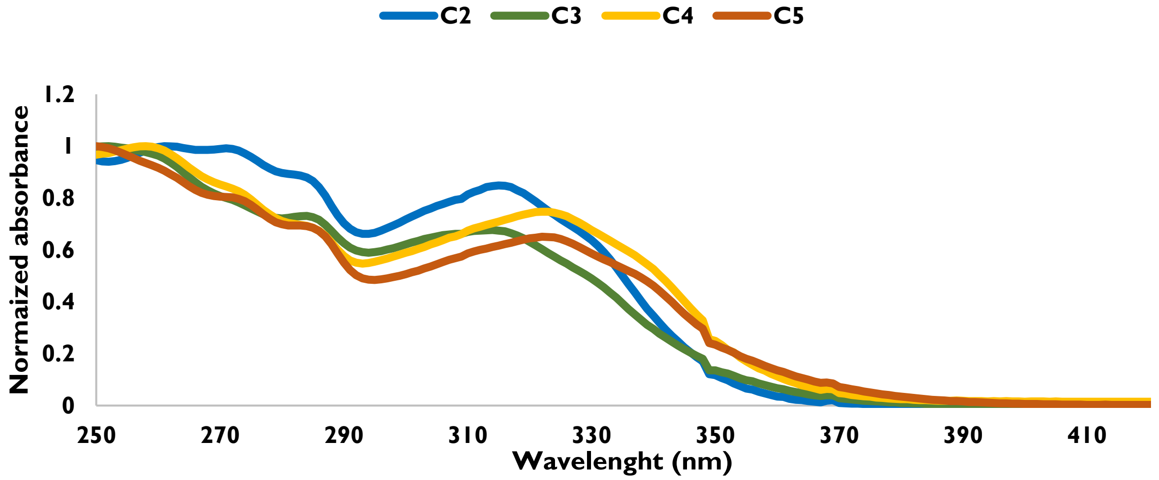

Figure S13 – Absorption profiles of 10^{-4}M solutions of catalysts **C2-C5** in dry, degassed, acetonitrile after the *addition of 3 equiv. of Cs_2CO_3* at 298K.

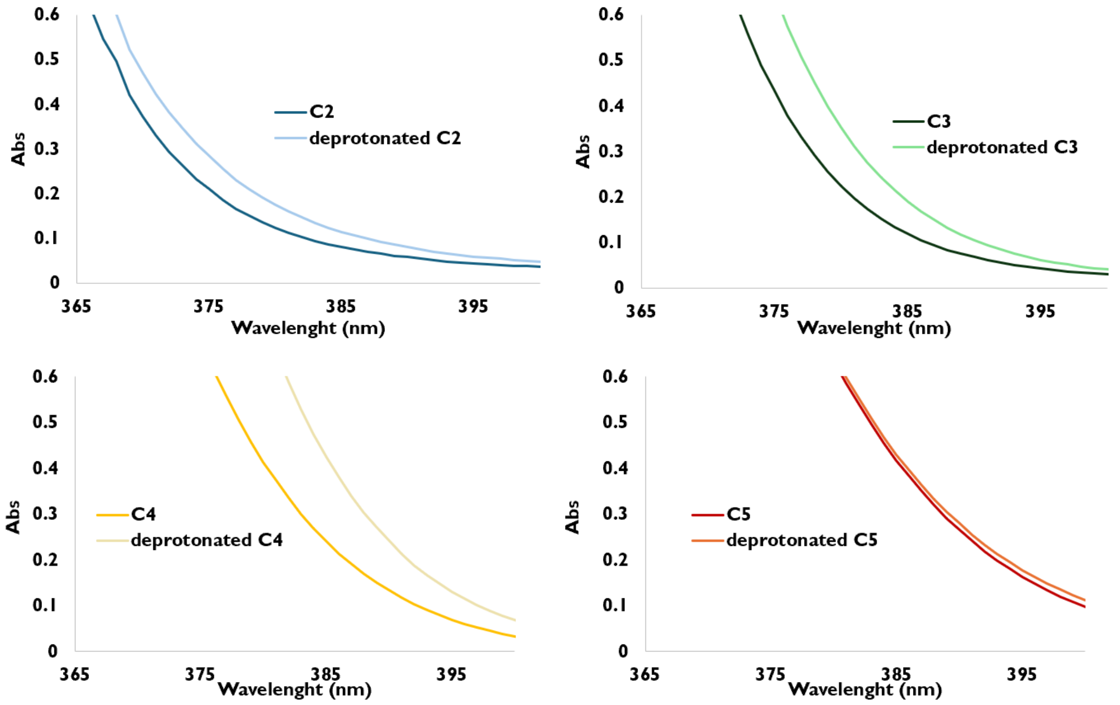

Figure S14 – Absorption profiles of 10^{-3}M solutions of catalysts **C2-C5** in dry, degassed, acetonitrile before and after the addition of 3 equiv. of Cs_2CO_3 at 298 K (only tails of absorption are shown for simplicity).

F2.2 Photophysical Characterization of Catalysts C2-C5: Summary of Relevant Data

Table S3. Representative parameters for catalysts **C2-C5** ***at 298K*** 10^{-4}M CH_3CN solutions

Catalyst	Excitation 298 K[a]	Emission 298 K[a]			Δ exc-em
	λ_{exc}(nm)	λ_{em}(nm)	τ (ns) S^1	τ (μs) T^1	Δ(nm)
**C2**	357	445	2.8	9	88
**C3**	352	449	2.9	12.7	97
**C4**	363	459	4.6	10.3	96
**C5**	374	472	3	17.4	98

[a] Degassed samples

Table S4. Representative parameters for catalysts **C2-C5** ***at 77K*** of 10^{-4}M CH_3CN solutions

Catalyst	Excitation 77 K[a]	Emission 77 K[a]			Δ exc-em	Triplet energy
	λ_{exc}(nm)	λ_{em}(nm)	τ_{ox} (ns) S^1	τ_{ox} (μs) T^1	Δ(nm)	Kcal/mol
**C2**	347	513	1.2	0.9 (53%), 7.3 (47%)	166	67.0
**C3**	366	540	1.2	1.1 (62%), 8.8 (38%)	174	62.4
**C4**	354	540	1.2 (20%) 10.3 (80%)	0.6 (18%), 5.0 (82%)	186	63.5
**C5**	356	547	1.2	0.7 (20%), 6.0 (79%)	191	63.7

[a] In a glassy matrix of CH_3CN

Table S5. Representative parameters for the ***deprotonated*** catalysts **C2-C5** at 298K of 10^{-4}M CH_3CN solutions;

Catalyst	Excitation 298 K[a,b]	Emission 298 K[a,b]			Δ exc-em
	λ(nm)	λ_{em}(nm)	τ (ns) S^1	τ (μs) T^1	Δ(nm)
**C2**	354	445	3.0	13.4	91
**C3**	360	449	3.1	18.0	89
**C4**	359	466	5.0	21.1	107
**C5**	367	473	3.4	15.2	106

[a] After stirring with 3 equiv. of Cs_2CO_3 and filtrating the excess of base. [b] Degassed samples.

F2.3 Photophysical Characterization of Catalysts C2-C5

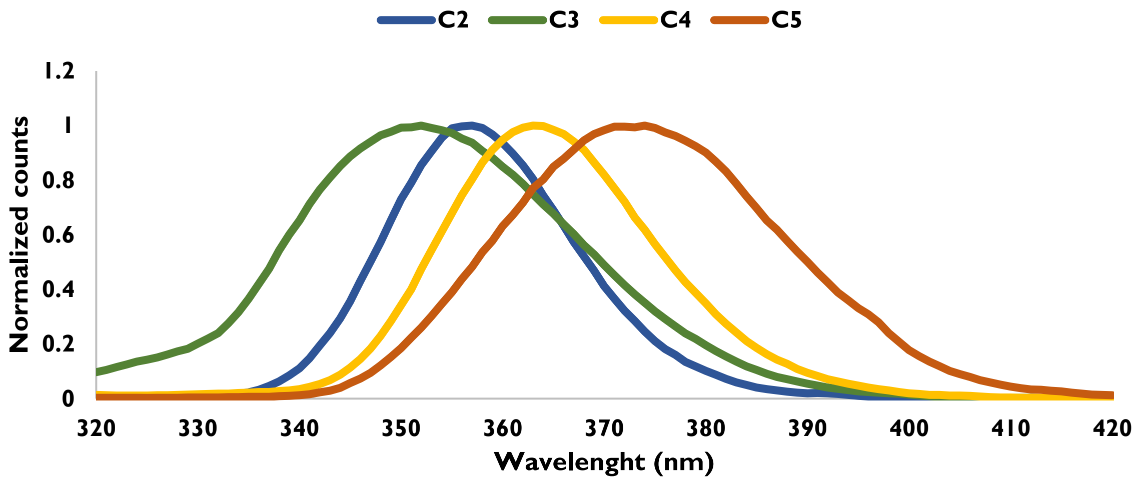

***Figure* S15**. Excitation profiles of 10^{-4}M CH_3CN solutions of catalysts **C2-C5** *at 298K*

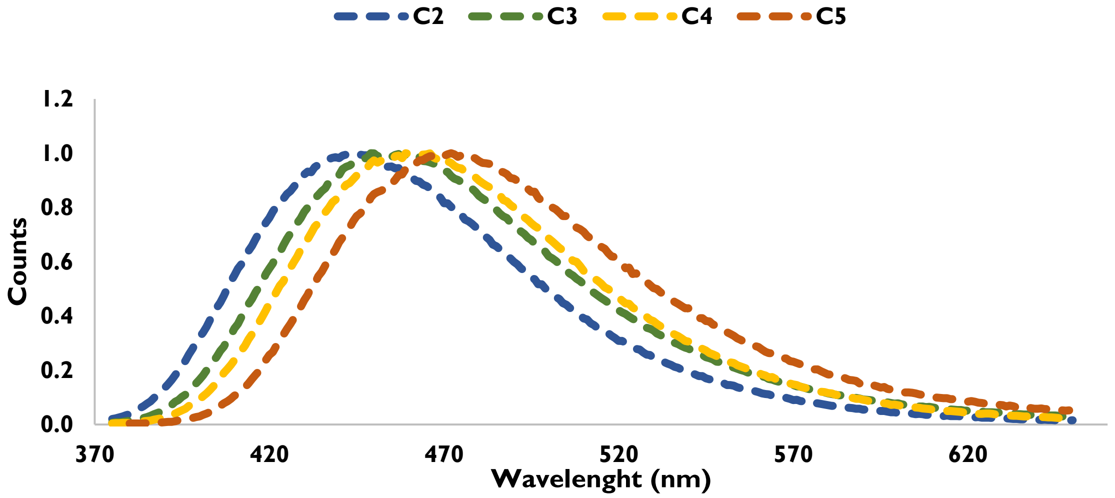

Figure S16. Emission profiles of 10^{-4}M CH_3CN solutions of catalysts **C2-C5** *at 298K*

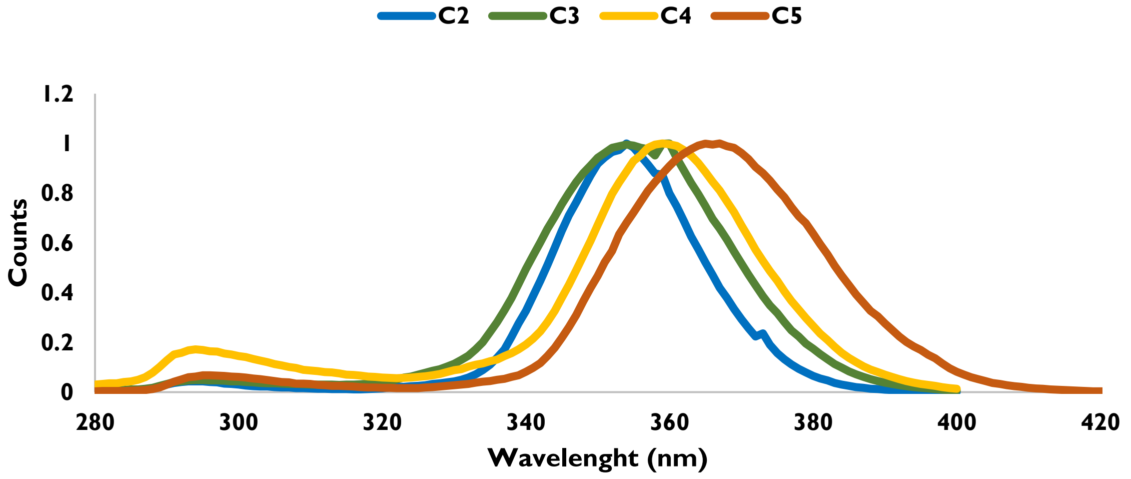

Figure S17. Excitation profiles of 10^{-4}M CH_3CN solutions of *deprotonated* catalysts **C2-C5** in the presence of 3 equiv. of Cs_2CO_3 at 298K.

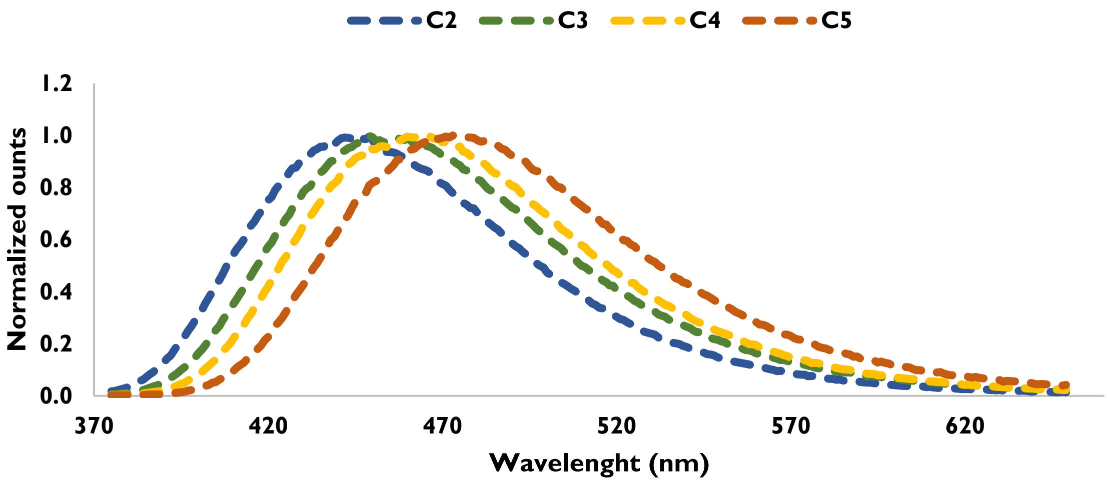

Figure S18. Emission profiles of 10^{-4}M CH_3CN solutions of *deprotonated* **C2-C5** in the presence of 3 equivalents of Cs_2CO_3

F2.4 E(0,0) Determination of the Deprotonated Catalysts C2-C5

Fluorescence measurements were carried out on an Edinburgh FLSP920 spectrometer equipped with a 450 W xenon arc lamp, double excitation and single emission monochromators, and a Peltier-cooled Hamamatsu R928P photomultiplier tube (185−850 nm).

The emission spectrum of the deprotonated catalysts **C2-C5** (formed *in situ* from **C2-C5** upon addition of 3 equivalents of Cs_2CO_3 in dry, degassed CH_3CN) was superimposed with the absorption spectra (Figure S19).

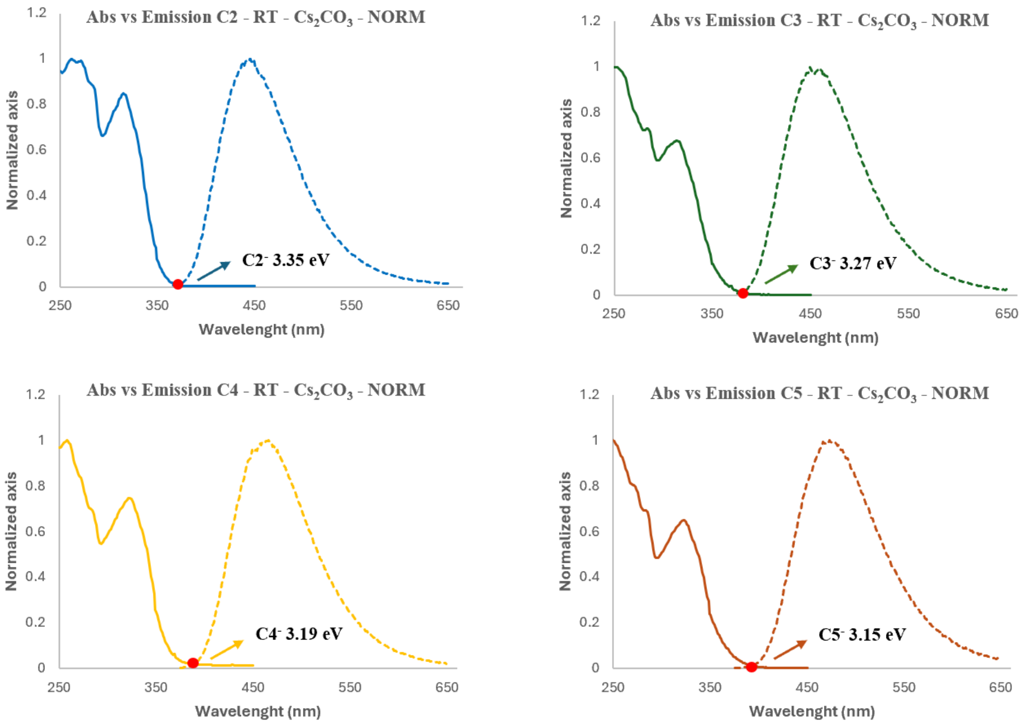

Figure S19. Absorption-emission overlap of 10^{-4}M solutions of catalysts **C2-C5** in dry, degassed, acetonitrile after the addition of 3 equiv. of Cs_2CO_3 at 298K.

F2.5 Triplet Energy Measurement of the Neutral Catalysts C2-C5

Triplet measurements of neutral catalysts **C2-C5** were obtained from low temperature (77 K) emission measurement. The low temperature emission spectrum was measured in FLS900 Fluorescence spectrometer (*Edinburgh Instrument*).

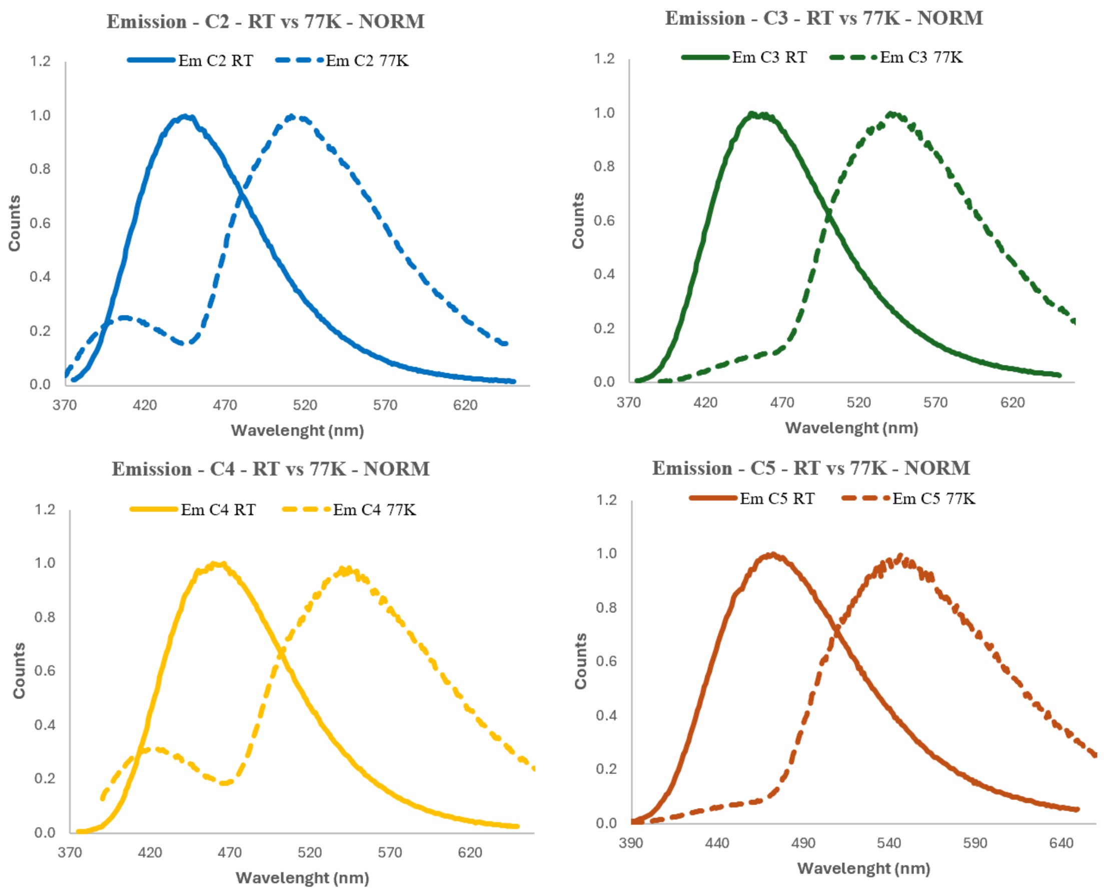

Figure S20. Comparison between emission of **C2-C5** solutions 10^{-4}M in CH_3CN to emission profile of glassy matrix at 77K

In Figure S20, the room temperature emission and glass temperature emission spectra are presented for catalysts **C2-C5**. The red-shifted emission observed at glass temperature is safely assigned to the emission from the triplet state. Since assignment of vibronic peaks was not possible, the crossing point of the tangent for the onset of emission was used for its determination.

F2.6 Stern-Volmer Quenching Studies

Fluorescence measurements were carried out on an Edinburgh FLSP920 spectrometer equipped with a 450 W xenon arc lamp, double excitation and single emission monochromators. The excitation wavelength was fixed at 350 nm for all the experiments.

F2.6.1 Stern-Volmer Quenching Studies with 4-Chloroanisole 1a

A 1.0 M solution of the quencher substrate (4-chloroanisole **1a**) in dry, degassed, CH_3CN (HPLC grade) was prepared and 40 µL of this stock solution were added to the solution (10^{-4}M HPLC grade CH_3CN) of the *deprotonated* catalyst **C2**$^-$, prepared according to the procedure detailed at the beginning of section F.2. The addition of the substrate solution **1a** was repeated four times. After each addition, the solution was mixed, and the emission spectra of the excited catalyst was acquired from 320 nm to 600 nm (the excitation wavelength was fixed at 350 nm). A solvent blank was subtracted from all the measurements.

The results shown in Figure S21 indicate that substrate **1a** quenched the excited-state emission of the deprotonated **C2**. The Stern-Volmer plot shows a linear correlation between the amounts of substrates and the ratio I_0/I, following the relationship: $\mathbf{I_0/I = 1 + K_{SV}[Q]}$ **(Q = Quencher)** (Figure S21).

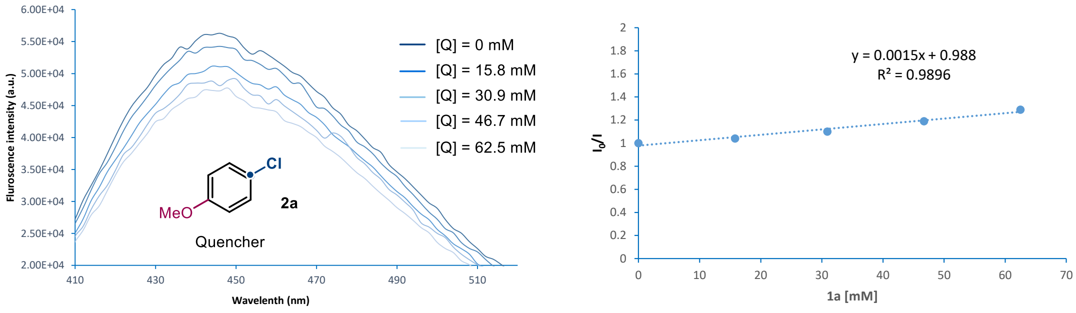

Figure S21. Stern-Volmer quenching studies of the excited deprotonated catalyst **C2**$^-$ with substrate **1a**

F2.6.2 Stern-Volmer Quenching Studies with Methyl Fumarate *E*-3a

A 1.0 M solution of the quencher substrate (methyl fumarate *E*-**3a**) in dry, degassed, CH_3CN (HPLC grade) was prepared, and 40 µL of this stock solution were added to a 10^{-4} M solution (HPLC grade CH_3CN) of either the deprotonated catalyst **C2**$^-$ or the neutral catalyst **C2**. The solutions of the catalyst were prepared according to the procedure detailed at the beginning of section F.2. The addition of the substrate solution **3a** was repeated five times. After each addition, the solution was mixed, and the emission spectra of the excited catalyst was acquired from 380 nm to 690 nm (the excitation wavelength was fixed at 350 nm). A solvent blank was subtracted from all the measurements.

The results shown in Figures S22 (quenching studies on *deprotonated* catalyst **C2**$^-$) and S23 (quenching studies on *neutral* catalyst **C2**) indicate that substrate **3a** quenched the excited-state emission of both the deprotonated **C2**$^-$ or neutral **C2** with a different behavior.

Stern Volmer quenching studies with 3a and deprotonated C2⁻

The Stern-Volmer plot for the quenching studies performed on *deprotonated* **C2⁻** (Figure S22 – right) with **3a** (dimethyl fumarate) shows a linear correlation between the amounts of substrates and the ratio **I_0/I**, following the relationship: **$I_0/I = 1 + K_{SV}[Q]$ (Q = Quencher)**.

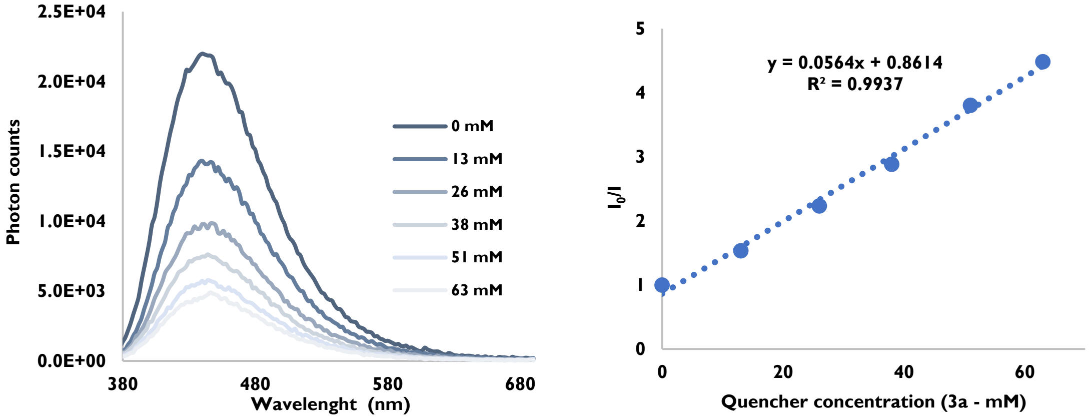

Figure S22 – Stern-Volmer fluorescence quenching studies of deprotonated **C2⁻** with **3a.**

Stern Volmer quenching studies with 3a and neutral C2

The Stern-Volmer plot for quenching studies of *neutral* **C2** (Figure S23 – right) with **3a** (dimethyl fumarate) reveals a departure from the expected linear relationship between substrate concentration and the fluorescence intensity ratio I_0/I. Instead, the data fits an exponential curve with an upward deviation from linearity. This behavior could stem from several factors, such as the simultaneous presence of dynamic and static quenching or the influence of more intricate quenching mechanisms like Förster resonance energy transfer (FRET) or Dexter energy transfer.[37] As a result, a Stern-Volmer quenching constant was not determined for this system.

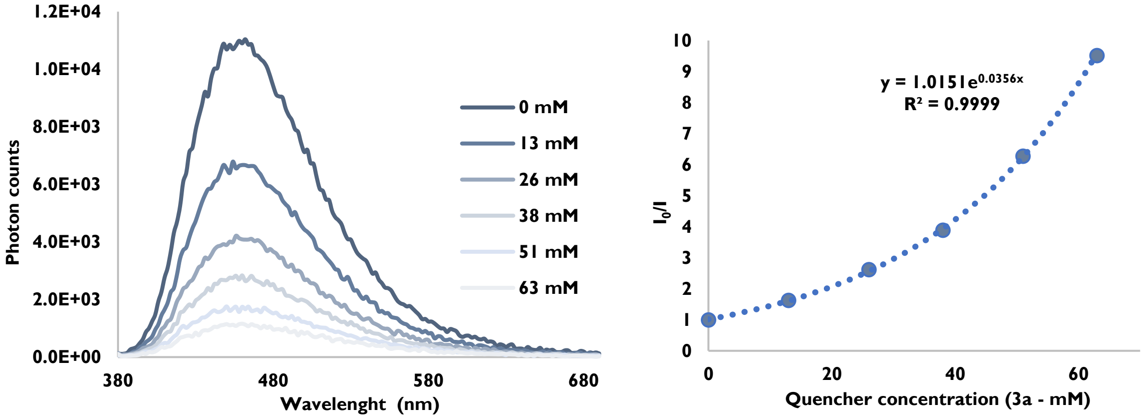

Figure S23 – Stern-Volmer fluorescence quenching studies of **C2** with **3a.**

Based on the experimental results discussed in the main text (e.g., the inability of the deprotonated catalyst to drive the *E/Z* photoisomerization of **3a**, Figure 4a), the different behaviors observed for deprotonated **C2⁻** and neutral **C2** catalysts during the quenching studies can be explained by distinct quenching mechanisms. *When the catalyst is deprotonated, single-electron reduction is likely at play, whereas energy transfer is the dominant process when the catalyst is in its neutral form*. This mechanistic distinction accounts for the differences in the

Stern-Volmer plots and the observed deviation from linearity in the case of the triplet energy transfer with catalyst **C2**.

Further Studies on the Behavior of Deprotonated Catalyst C2⁻ in the Activation of Fumarate 3a

When the *E/Z* photoisomerization of dimethyl fumarate (*E*-**3a**) was carried out using catalyst **C2** in the presence of Cs_2CO_3, no isomerization was observed (see Figure 4a, entry 1 in the main manuscript). This suggests that the deprotonated form of catalyst **C2⁻** is inactive in this transformation, likely due to its incompatibility with an EnT mechanism. In contrast, the neutral catalyst **C2** efficiently promoted the isomerization, confirming its effectiveness as an EnT photocatalyst (Figure 4a, entry 2 in the main manuscript).

To further investigate the reactivity of the deprotonated species, the same model reaction was performed in the presence of γ-terpinene as a hydrogen donor (see below).

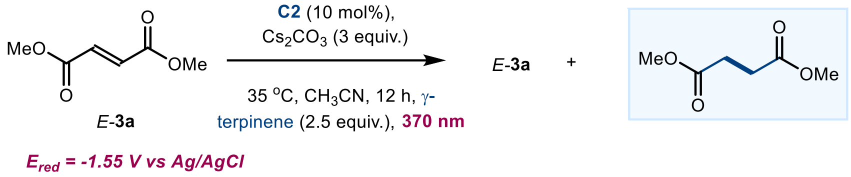

Under these conditions, and using the deprotonated catalyst **C2⁻**, GC-MS analysis after 12 hours revealed the formation of dimethyl succinate, the reduced product from **3a** (see Scheme S2). This observation provides evidence that **C2⁻** engages in SET with *E*-**3a** (E_{red} = -1.55V vs Ag/AgCl),[38] consistent with a mechanistic shift from EnT to SET upon deprotonation of the catalyst.

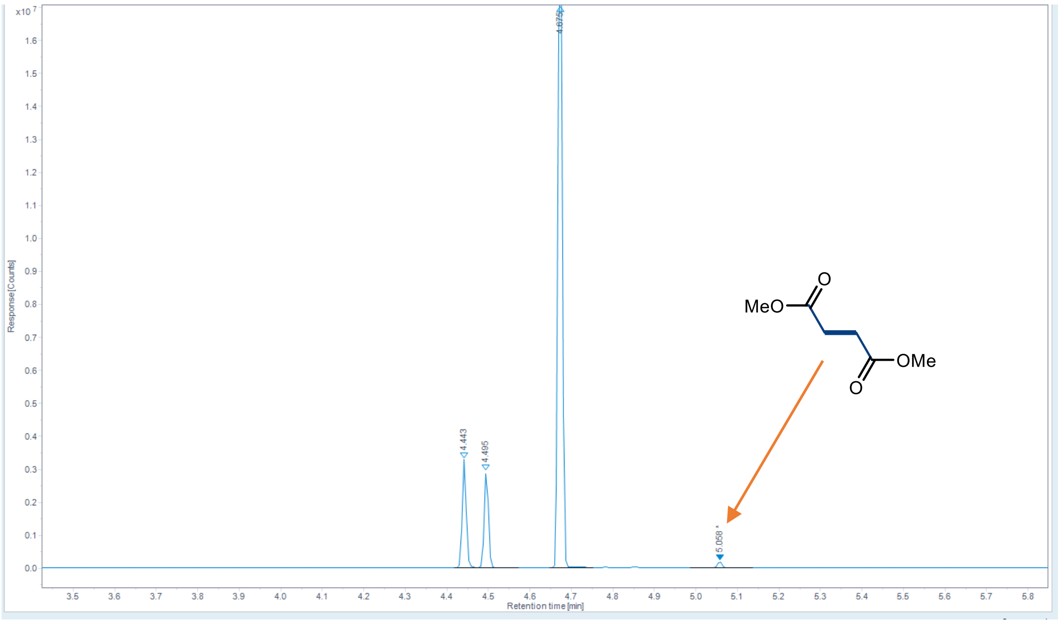

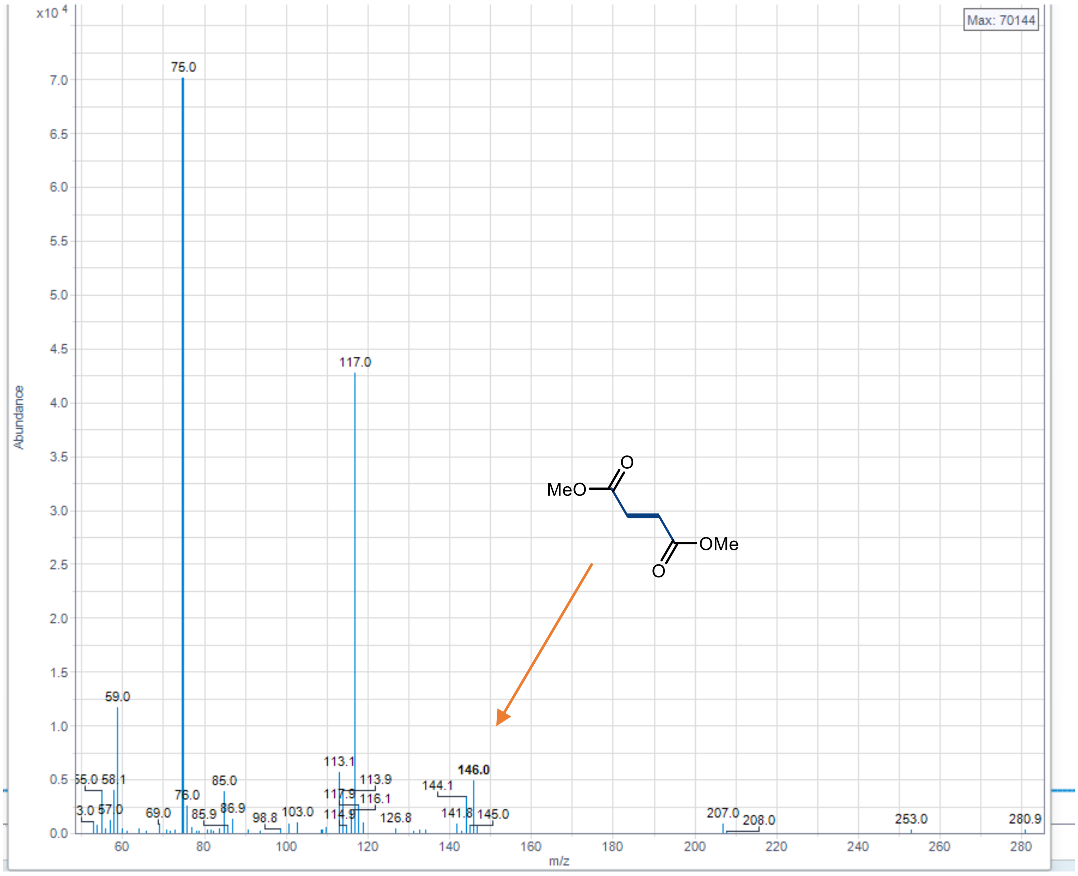

Scheme S2. GC-MS analysis of the reaction between *E*-**3a** catalyzed by the deprotonated **C2** in the presence of γ-terpinene.

F3. Electrochemical Studies

Cyclic voltammetry studies were carried out on a PGSTAT204 potentiostat, offering compliance voltage up to ± 20 V (available at the counter electrode), ± 10 V scan range and ±0.4 A current range with a glassy carbon disk electrode (diameter: 3 mm) as working electrode. A silver wire coated with AgCl immersed in a 3.0 M aqueous solution of KCl and separated from the analyte by a fritted glass disk was employed as the reference electrode and a Pt wire counter-electrode completed the electrochemical setup. The scan rate was 100 mV/s unless otherwise stated. The substrates were measured at concentration of 2 mM in CH_3CN after the addition and filtration of 3 equivalents of Cs_2CO_3, with $TBAPF_6$ (0.1 M) as electrolyte.

F3.1 Oxidation Peaks for the Deprotonated Catalysts C2-C5

Potentials are quoted with the following notation: E_p^A (E_{Ox}) refers to the anodic peak potential while E_p^C (E_{Red}) refers to the cathodic peak potential (Figure S24 to S27).

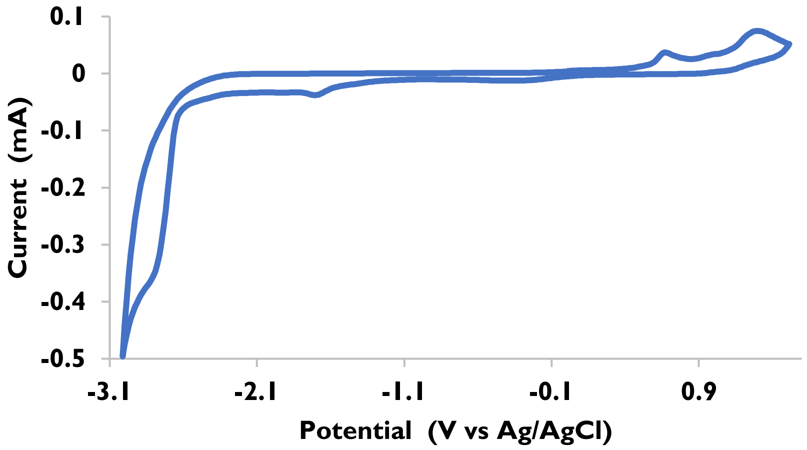

Figure S24. CV of the deprotonated catalyst **C2⁻** in CH_3CN starting with the oxidation, irreversible oxidation and reduction, Ep^A = 0.65 V with a sweep rate of 100 mV/s.

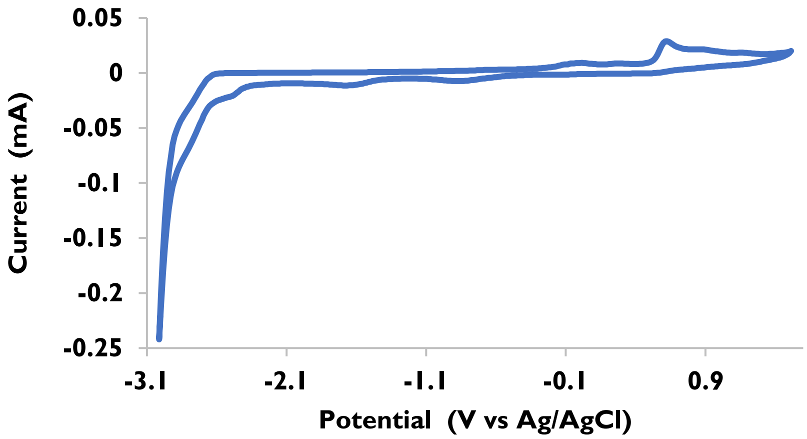

Figure S25: CV of **C3⁻** in CH_3CN starting with the oxidation, irreversible oxidation and reduction, Ep^A = 0.63 V with a sweep rate of 100 mV/s.

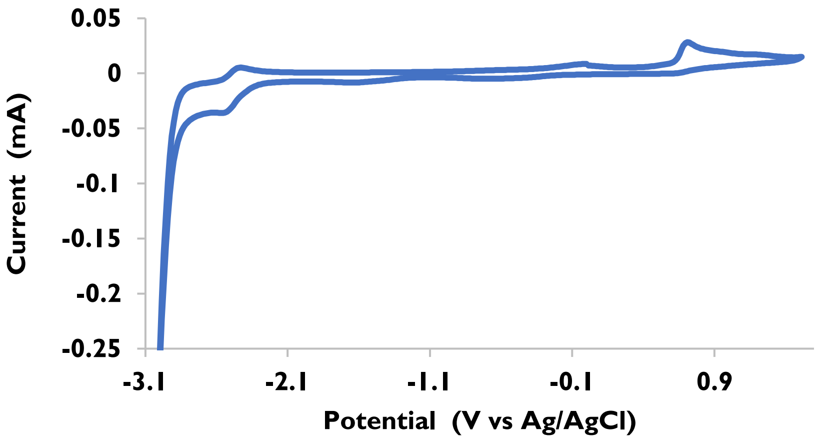

Figure S26. CV of **C4⁻** in CH_3CN starting with the oxidation, irreversible oxidation and reduction, Ep^A = 0.71 V with a sweep rate of 100 mV/s.

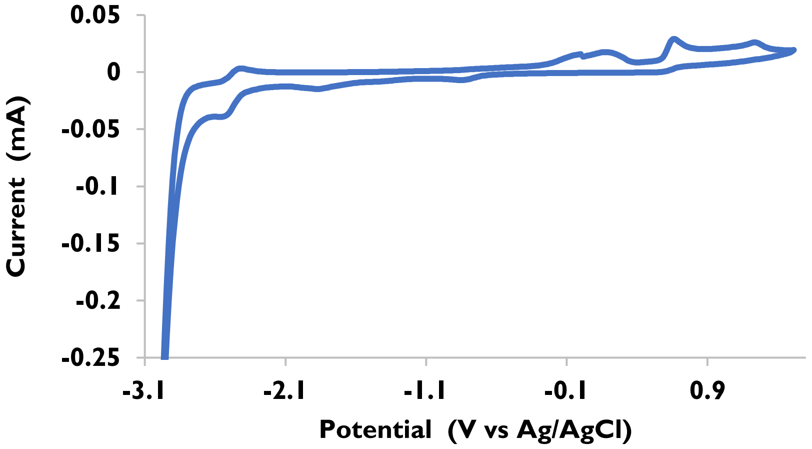

Figure S27: CV of **C5**$^-$ in CH_3CN starting with the oxidation, irreversible oxidation and reduction, Ep^A = 0.15 V with a sweep rate of 100 mV/s.

F3.2 Conversion of the Potential from Ag/AgCl to SCE for Catalysts C2-C5

The conversion of the redox potential from Ag/AgCl to SCE was done according to the literature by measuring the redox potential of ferrocene as reference in CH_3CN (Figure S28).[40]

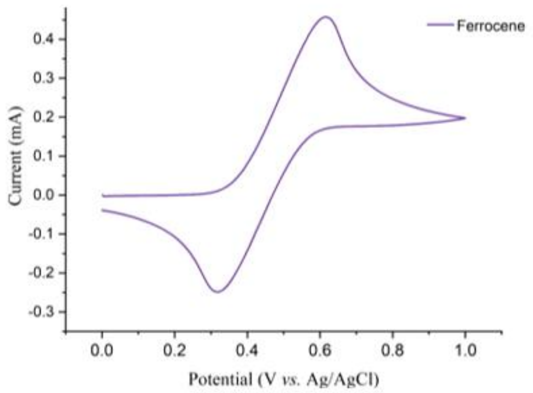

Figure S28. CV of ferrocene in CH_3CN, reversible reduction and oxidation $E_{1/2}$ = 0.46 V.

With the reference CV, the redox potential vs. SCE for the deprotonated catalyst **C2-C5** in CH_3CN was calculated using the following equations:

C2$^-$ E_p^A (E_{Ox}) (**Ag/AgCl to Fc/Fc**$^+$) = 0.65 - 0.46 = 0.19 V vs. Fc/Fc$^+$

C2$^-$ E_p^A (E_{Ox}) **Fc**$^+$ **to SCE**) = 0.19 + 0.38= 0.57 V vs. SCE

C3$^-$ E_p^A (E_{Ox}) (**Ag/AgCl to Fc/Fc**$^+$) = 0.63 - 0.46 = 0.17 V vs. Fc/Fc$^+$

C3$^-$ E_p^A (E_{Ox}) **Fc**$^+$ **to SCE**) = 0.17 + 0.38= 0.55 V vs. SCE

C4$^-$ E_p^A (E_{Ox}) (**Ag/AgCl to Fc/Fc**$^+$) = 0.71 - 0.46 = 0.25 V vs. Fc/Fc$^+$

C4$^-$ E_p^A (E_{Ox}) **Fc**$^+$ **to SCE**) = 0.25 + 0.38= 0.63 V vs. SCE

C5$^-$ E_p^A (E_{Ox}) (**Ag/AgCl to Fc/Fc**$^+$) = 0.15 - 0.46 = -0.31 V vs. Fc/Fc$^+$

C5$^-$ E_p^A (E_{Ox}) **Fc**$^+$ **to SCE**) = -0.31 + 0.38= 0.07 V vs. SCE

F3.3. Evaluation of the Excited-state Potential of the Deprotonated Catalysts C2-C5

Using the data collected from the CV studies (Figure S24-S27) and from the absorption and emission spectra overlap (Figure S19) of deprotonated catalysts **C2-C5**, we could estimate the redox potential of the excited state with the following equation:

$$E(\mathbf{C}^{\bullet+}/\ \mathbf{C}^{*}) = E(\mathbf{C}^{\bullet+}/\ \mathbf{C}) - E_{0\text{-}0}(\mathbf{C^{*}})/(\mathbf{C})$$

Since the electrochemical oxidation of deprotonated catalysts **C2-C5** is irreversible (Figure S20-S23), the irreversible oxidation peak potential anode $\mathbf{Ep^{A}}$ was used for E($\mathbf{Pc^{\bullet+}/Pc}$).

$E_{0\text{-}0}$(**C***)/(**C**) was approximately determined spectroscopically from the overlap of the tail of absorption spectrum with the emission spectrum for every catalyst (Figure S19).

The redox potential of the excited $\mathbf{C2^{-}*}$:

E($\mathbf{C^{\bullet+}}$/ $\mathbf{C^{*}}$) = 0.65 – 3.35 = **-2.7 V** vs. Ag/AgCl

E($\mathbf{C^{\bullet+}}$/ $\mathbf{C^{*}}$) = 0.57 – 3.35 = **-2.78 V** vs. SCE

The redox potential of the excited $\mathbf{C3^{-}*}$:

E($\mathbf{C^{\bullet+}}$/ $\mathbf{C^{*}}$) = 0.63 – 3.27 = **-2.64 V** vs. Ag/AgCl

E($\mathbf{C^{\bullet+}}$/ $\mathbf{C^{*}}$) = 0.55 – 3.27 = **-2.72 V** vs. SCE

The redox potential of the excited $\mathbf{C4^{-}*}$:

E($\mathbf{C^{\bullet+}}$/ $\mathbf{C^{*}}$) = 0.71 – 3.19 = **-2.48 V** vs. Ag/AgCl

E($\mathbf{C^{\bullet+}}$/ $\mathbf{C^{*}}$) = 0.63 – 3.19 = **-2.56 V** vs. SCE

The redox potential of the excited $\mathbf{C5^{-}*}$:

E($\mathbf{C^{\bullet+}}$/ $\mathbf{C^{*}}$) = 0.15 – 3.15 = **-3.00 V** vs. Ag/AgCl

E($\mathbf{C^{\bullet+}}$/ $\mathbf{C^{*}}$) = 0.07 – 3.15 = **-3.08 V** vs. SCE

F4. NMR Studies of the Deprotonated Catalysts

To assess the extent of catalyst deprotonation under the reaction conditions, NMR studies were conducted in CD_3CN. ^1H NMR spectra were recorded for 100 mM solutions of catalysts **C2**–**C5** in deuterated acetonitrile, both before and after the addition of 3 equivalents of Cs_2CO_3 (Figure S29–S32). A significant shift was observed for all aromatic signals, indicating the formation of an electron-rich phenate ion, which enhances shielding across the conjugated system.

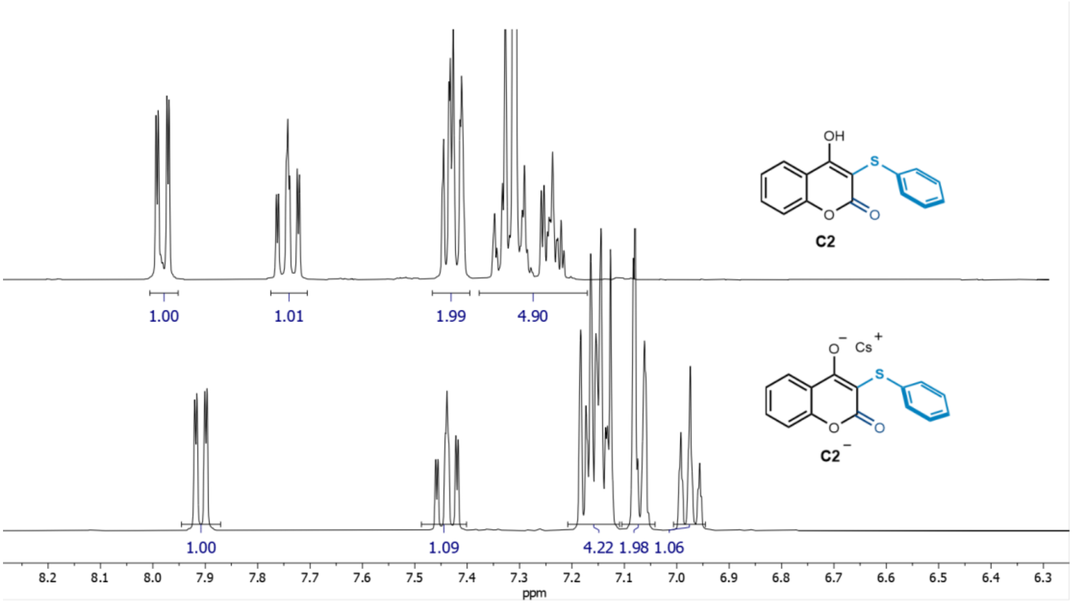

Figure S29. NMR studies of catalyst **C2** (up) and deprotonated **C2** (down) in CD_3CN (100 mM); zoom on the aromatic signals.

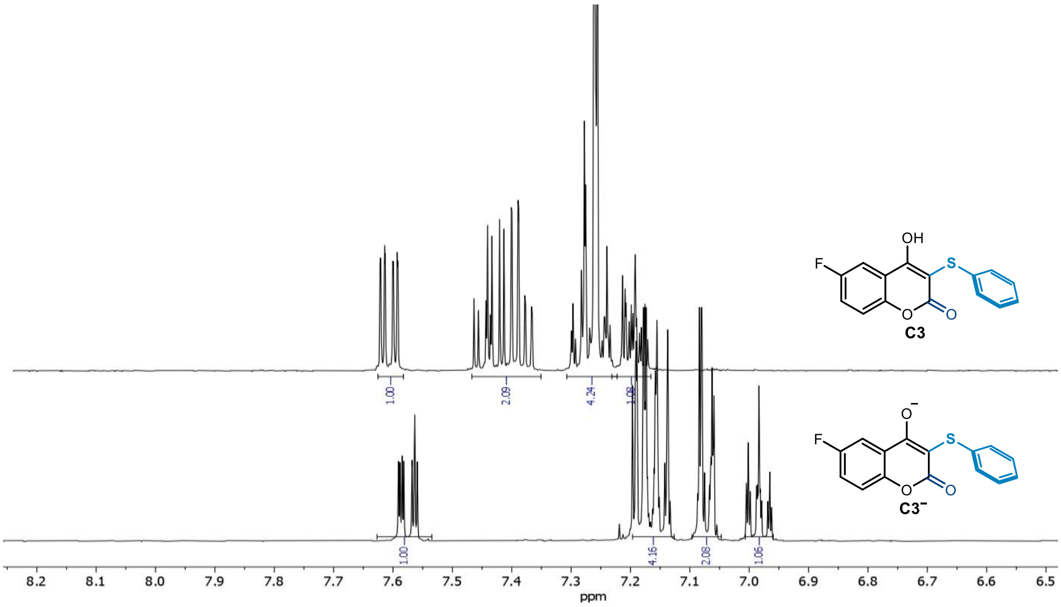

Figure S30 – NMR studies of **C3** (up) and deprotonated **C3** (down) in CD_3CN (100 mM); zoom on the aromatic signals.

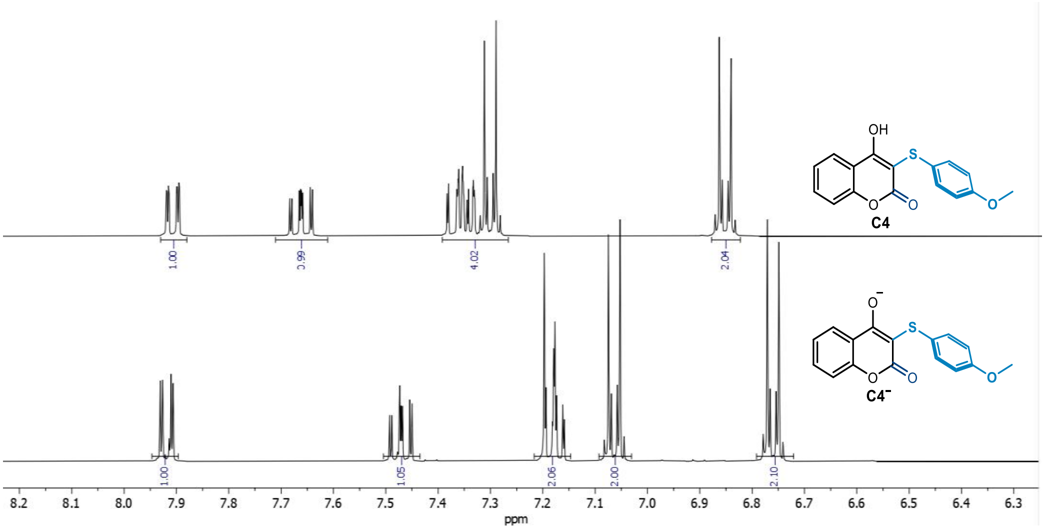

Figure S31 – NMR studies of **C4** (up) and deprotonated **C4** (down) in CD_3CN (100 mM); zoom on the aromatic signals.

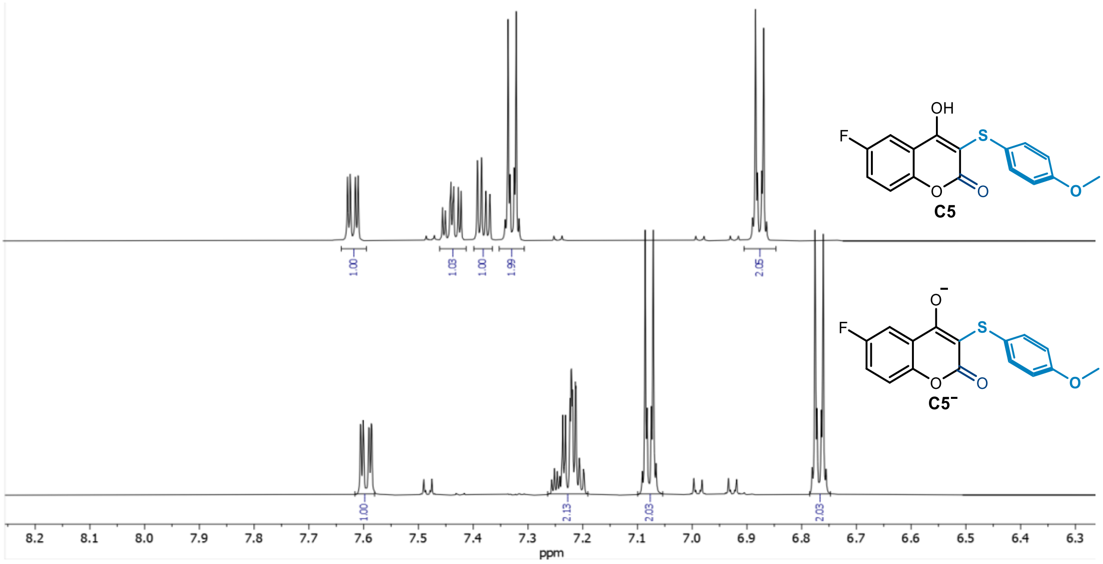

Figure S32 – NMR studies of **C5** (up) and deprotonated **C5** (down) in CD_3CN (100 mM); zoom on the aromatic signals.

F5. Computational Studies

All the calculations were performed with the GAUSSIAN16, Revision A.03 program package.[41] Structure optimizations were carried out at B3LYP/6-31+G** level of theory[42] with the polarizable continuum model (PCM)[43] for the solvation effects of DMSO or ACN. The vibrational frequencies were computed at the same level to confirm each optimized structure as stationary points. NBO analysis was performed after full geometry and energy optimization for MO visualization (for **C1-C5** structures). TD-DFT calculations were conducted with the optimized geometries at the same level of theory with n states =10.

F5.1. Rational Photocatalyst Design via Computed UV-Vis Spectra

To explore the influence of substituents on the photophysical properties of thiophenyl-coumarin catalysts, we screened a selection of compounds having fluorine (-F) or methoxy (-OMe) substituents at X and Y positions of the catalysts scaffold. We analyzed both the catalysts eventually synthesized and tested (**C2**–**C5**), as well as all remaining permutations involving fluorine (-F) and methoxy (-OMe) groups at the X and Y positions (**C6**–**C10**). In total, nine structures (**C2**–**C10**) were examined, whose phenate anion geometries were first optimized and then UV-Vis spectra were obtained through TD-DFT (B3LYP/6-31+G**; SMD=acetonitrile; NStates=10).

The obtained UV-Vis spectra and candidate's structures are shown below (Figure S33):

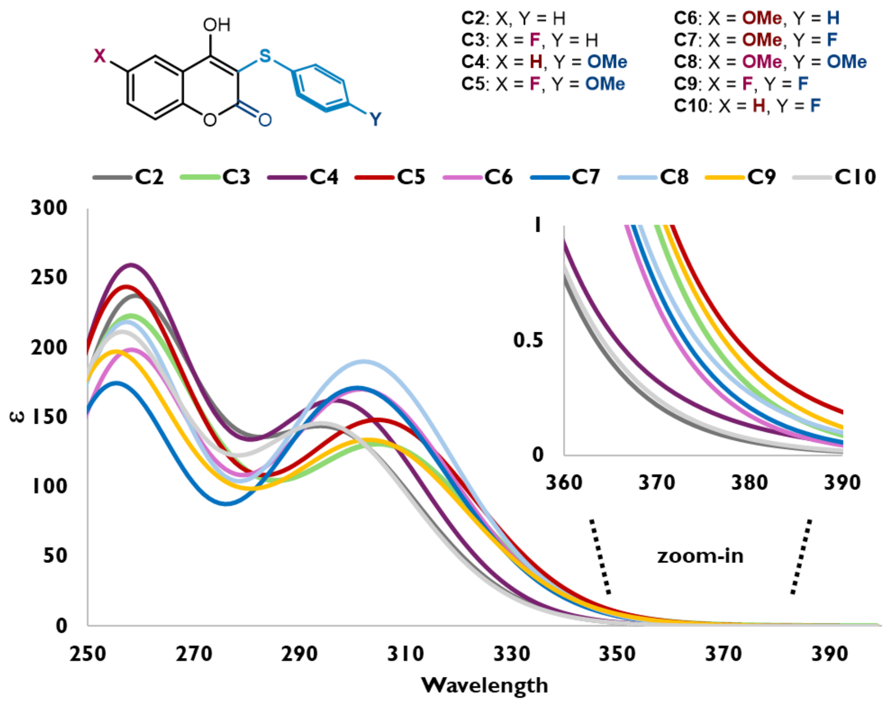

Figure S33 Calculated UV-Vis absorption profiles of family **C2-C10** with TD-DFT (B3LYP/6-31+G**; SMD=acetonitrile; NStates=10).

As shown in Figure S33 (top right corner), fluorine substitution at the X position has the most significant effect in red-shifting the absorption spectrum (notably in **C5**, **C9**, and **C3**). Additionally, catalyst **C5**, featuring a *para*-methoxy group at position Y and a fluorine atom at position X on the hydroxycoumarin core, exemplifies our push-pull design strategy, as supported by the results presented in the manuscript.

Based on these findings and the practical availability of starting materials, we selected the subfamily **C2**–**C5** for further investigation of photoreduction and energy transfer processes.

F5.2. Computational study of excited states for C1-C5

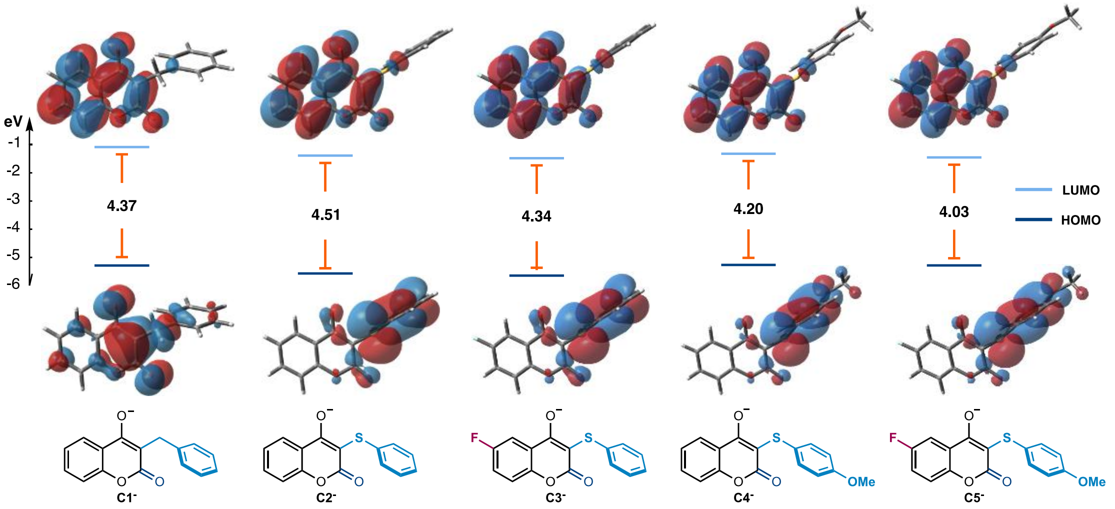

Figure S34. Calculated HOMO-LUMO graph and energy gaps of deprotonated **C1**-**C5**.

Table S6. HOMO and LUMO energies of all the catalysts

**PC**[a]	E_{HOMO}[b]	E_{LUMO}[b]
**C1**⁻	-5.42	-1.05
**C2**⁻	-5.75	-1.24
**C3**⁻	-5.77	-1.43
**C4**⁻	-5.43	-1.23
**C5**⁻	-5.45	-1.42

[a] Deprotonated. [b] Energies are given in eV.

Table S7. Computational spectral data and most relevant transition involved in the formation of the lowest excited state

**PC**[a]	Transition	ΔE(eV)/ λ(nm) (experimental)[b]	Transition Character
**C1**⁻	$S_0 \rightarrow S_1$	3.90/317 (314)	Orbital 66 (HOMO) → Orbital 67 (LUMO) 97%
**C2**⁻	$S_0 \rightarrow S_1$	3.85/322 (315)	Orbital 70 (HOMO) → Orbital 71 (LUMO) 97%
**C3**⁻	$S_0 \rightarrow S_1$	3.69/336 (314)	Orbital 74 (HOMO)→Orbital 75 (LUMO) 98%
**C4**⁻	$S_0 \rightarrow S_1$	3.59/345 (323)	Orbital 78 (HOMO)→Orbital 79 (LUMO) 98%
**C5**⁻	$S_0 \rightarrow S_1$	3.43/361 (322)	Orbital 82 (HOMO)→Orbital 83 (LUMO) 98%

[a] Deprotonated. [b] Experimental absorption peaks are marked in parentheses.

Optimized geometries and Enthalpy and Gibbs free energies (in Hartree):

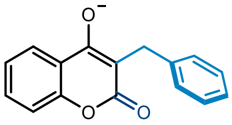

C1

C1⁻

E = -841.983299

G = -842.042480

-1 1

C	-4.71312000	0.02997500	0.91179300
C	-3.73216000	1.01813400	0.88456600
C	-2.48802400	0.73202500	0.30784500
C	-2.20799700	-0.52695400	-0.24166000
C	-3.21434100	-1.50800800	-0.20117800
C	-4.45612200	-1.23950200	0.36736200
H	-5.67951000	0.24711400	1.35744200
H	-3.91027900	2.00518700	1.29971000
C	-0.87280200	-0.78768600	-0.84105400
H	-2.99147100	-2.48106200	-0.62671100
H	-5.22376000	-2.00713700	0.39106500
C	0.04798400	0.29399900	-0.81221900
C	-0.27949800	1.55229800	-0.25026000
C	1.42113400	0.13613700	-1.42424600
H	1.37104000	-0.71743100	-2.11134900
H	1.66655300	1.01987200	-2.02386100
C	2.56623400	-0.10747000	-0.44802800
C	2.42570000	-0.98596300	0.63877100
C	3.81013700	0.51376500	-0.63748200
C	3.49413000	-1.23883700	1.50422600
H	1.46887900	-1.47208500	0.80730200
C	4.88303300	0.26683400	0.22656000
H	3.93932700	1.20338800	-1.46873600
C	4.72986900	-0.61322100	1.30219300
H	3.36161300	-1.92405800	2.33748000
H	5.83460200	0.76511100	0.06091100
H	5.55969900	-0.80661200	1.97613600
O	-0.61890300	-1.92556100	-1.33709600
O	0.44467800	2.55840900	-0.18342100
O	-1.56097900	1.73647100	0.30520100

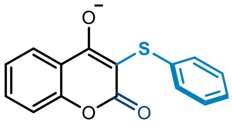

C2

C2⁻

E = -1200.882044

G = -1200.943005

-1 1

C	-4.82166900	-0.45040200	0.85494000
C	-3.95203200	0.62751800	0.99922100
C	-2.67991500	0.56026100	0.41794700
C	-2.26494200	-0.56516200	-0.30389400
C	-3.15937900	-1.64151800	-0.43532700
C	-4.42708000	-1.59101100	0.13590600
H	-5.80955000	-0.40396900	1.30360800
H	-4.23758200	1.51707800	1.55145000
C	-0.90515000	-0.59937000	-0.90789600
H	-2.82974900	-2.51068500	-0.99498200
H	-5.10901300	-2.42873300	0.02734200
C	-0.10996300	0.57371600	-0.69385900
C	-0.56452600	1.69757600	0.06106400
C	2.65023100	0.01872800	-0.28156500
C	2.28300700	-0.47253200	0.97833900
C	4.00335300	-0.00834300	-0.66231200
C	3.25694000	-0.98195800	1.84358000
H	1.24048200	-0.45652500	1.27970400
C	4.96944000	-0.51844900	0.20721600
H	4.30073700	0.36936100	-1.63744600
C	4.60305000	-1.00852600	1.46650500
H	2.95673000	-1.35903700	2.81758400
H	6.01102700	-0.53205600	-0.10190400
H	5.35543800	-1.40452300	2.14189300
O	-0.53445700	-1.61413900	-1.54742600
O	0.04066400	2.74020000	0.32348700
O	-1.86405200	1.64540100	0.59248000
S	1.48100300	0.69963900	-1.46342300

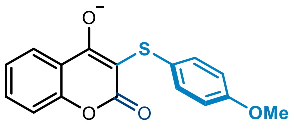

C3

C3⁻

E = -1300.129384

G = -1300.192386

-1 1

C	-4.55365700	0.09853000	0.85167700
C	-3.58931700	1.09335700	0.98619100
C	-2.32513000	0.91150700	0.41159300
C	-2.00722700	-0.25230700	-0.29756600
C	-2.98338000	-1.25410600	-0.43045800
C	-4.22403100	-1.05663600	0.14266700
H	-5.54148900	0.20991400	1.28518100
H	-3.80315100	2.00727800	1.52990600
C	-0.65304900	-0.41365200	-0.89646300
H	-2.74925400	-2.16033000	-0.97720400
C	0.23684200	0.68962200	-0.69544800
C	-0.11910000	1.85620800	0.04971500
C	2.94077100	-0.09315300	-0.28171000
C	2.53814700	-0.53301400	0.98614700
C	4.28402500	-0.24438000	-0.66797200
C	3.46800600	-1.11522900	1.85400500
H	1.50284100	-0.42143000	1.29198900
C	5.20589400	-0.82663500	0.20419300
H	4.60815400	0.09238800	-1.64953900
C	4.80429300	-1.26597500	1.47140400
H	3.14097900	-1.45155800	2.83425300
H	6.24041800	-0.93674100	-0.10924700
H	5.52225900	-1.71855100	2.14869000
O	-0.37441700	-1.46666400	-1.51901300
O	0.57437700	2.84309900	0.30451900
O	-1.41870400	1.92230900	0.57955900
S	1.83005100	0.66969700	-1.47047100
F	-5.17668300	-2.03232200	0.01390500

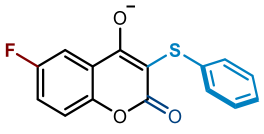

C4

C4⁻

E = -1315.376300

G = -1315.442738

-1 1

C	5.30866300	-1.31332700	-0.73184700
C	4.52593700	-0.33825700	-1.34470900
C	3.33348300	0.06460700	-0.73096100
C	2.91242600	-0.49119900	0.48298300
C	3.71805100	-1.47501500	1.08211300
C	4.90622600	-1.88614000	0.48617600

H	6.23458200	-1.62963800	-1.20301800
H	4.81881900	0.11557500	-2.28603300
C	1.63909700	-0.03601100	1.10459500
H	3.38435100	-1.90244400	2.02190900
C	0.93357600	0.98232700	0.38425000
C	1.38728900	1.51158700	-0.86220000
C	-1.89370700	0.68665900	0.47703900
C	-1.72930200	-0.41651300	-0.37343800
C	-3.18914800	1.03601800	0.87821300
C	-2.83316100	-1.14640200	-0.80987800
H	-0.73440200	-0.70625300	-0.69611000
C	-4.30356900	0.30807000	0.44534800
H	-3.34469000	1.88656600	1.53720300
C	-4.12811200	-0.79115800	-0.40527700
H	-2.70268400	-1.99983100	-1.46871200
H	-5.28938900	0.61053200	0.77794200
O	1.25785100	-0.54682400	2.18625900
O	0.84900900	2.36109400	-1.57708400
O	2.60081000	1.02346300	-1.37657900
S	-0.52959300	1.68918100	1.09053500
O	-5.14746000	-1.57152800	-0.88973900
C	-6.48624000	-1.25022300	-0.50929300
H	-7.12061100	-1.98728400	-1.00211000
H	-6.61877100	-1.32243100	0.57639700
H	-6.76462800	-0.24514300	-0.84683100
H	5.52017900	-2.64632100	0.95927000

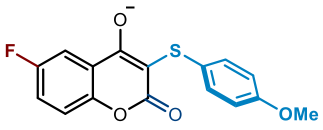

C5

C5$^-$

E = -1414.623656

G = -1414.692540

-1 1

C	5.08811700	-0.64922800	-1.05581700
C	4.20459400	0.33420900	-1.49189800
C	3.00566400	0.54185000	-0.79834800
C	2.67309800	-0.22080400	0.32678700
C	3.56729200	-1.21286900	0.76299100
C	4.74498500	-1.40183400	0.06736800
H	6.02426400	-0.83355600	-1.57139800
H	4.43194000	0.94187600	-2.36118800

C	1.38899700	0.02412700	1.04079800
H	3.32040300	-1.81025900	1.63313700
C	0.58428500	1.07887300	0.50312900
C	0.95086400	1.82594800	-0.65912200
C	-2.21374600	0.57815700	0.59174700
C	-1.99094200	-0.41245900	-0.37585900
C	-3.52720700	0.82170000	1.01174000
C	-3.05617700	-1.13567400	-0.90846900
H	-0.98119800	-0.61842000	-0.71618500
C	-4.60288300	0.09771400	0.48445800
H	-3.72756800	1.58554800	1.75886900
C	-4.36943000	-0.88827600	-0.48296500
H	-2.88088400	-1.90117700	-1.65856000
H	-5.60470300	0.31638800	0.83441500
O	1.09342500	-0.68079300	2.03586400
O	0.32249900	2.72652000	-1.21957100
O	2.17716000	1.52080100	-1.27418100
S	-0.90131200	1.55447800	1.34436700
F	5.61830500	-2.36782900	0.49224100
O	-5.34723800	-1.65346300	-1.06656100
C	-6.70254600	-1.43914200	-0.66946400
H	-7.29779600	-2.14368800	-1.25068000
H	-6.83970500	-1.64017000	0.39933300
H	-7.02506100	-0.41599300	-0.89465100

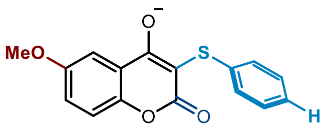

C6

C6^{-}

E = -1315.541079

G = -1315.357709

-1 1

C	-4.20791200	0.43099300	0.75755000
C	-3.18615200	1.36076300	0.97083100
C	-1.91798100	1.15056100	0.42412800
C	-1.65972100	0.00448000	-0.34194300
C	-2.68490300	-0.92227900	-0.55029000
C	-3.95328200	-0.71707100	-0.00839000
H	-3.36268600	2.25478100	1.56096000
C	-0.29764000	-0.21795900	-0.92521400
H	-2.46720700	-1.80424100	-1.14368400
C	0.65262900	0.81787400	-0.63690300
C	0.36120700	1.97650300	0.15815500
C	3.27866100	-0.18980300	-0.26954700
C	2.81966700	-0.74429900	0.93465600
C	4.62747300	-0.37686600	-0.62654400
C	3.68848900	-1.46329600	1.75766100
H	1.78057600	-0.60805700	1.21555800
C	5.49009200	-1.09701000	0.19967300
H	4.99745700	0.04757000	-1.55702700
C	5.02862900	-1.64734300	1.40131500
H	3.31072200	-1.88502000	2.68649400
H	6.52867900	-1.22773500	-0.09684700
O	-0.08022100	-1.24836300	-1.59271200
O	1.09588900	2.90076500	0.47314800
O	-0.96490100	2.09212100	0.66903900
S	2.25248900	0.74225000	-1.39168000
H	5.70020200	-2.20845100	2.04585900
H	-5.18432400	0.61527600	1.19132900
O	-4.89722900	-1.69428000	-0.27501900
C	-6.19540200	-1.52570200	0.25595600
H	-6.77115400	-2.39522800	-0.06932200
H	-6.18642100	-1.49118500	1.35505500
H	-6.67711100	-0.61293200	-0.12370600

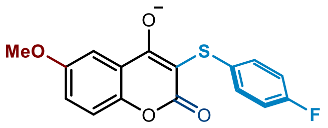

C7

C7^{-}

E = -1414.784038

G = -1414.610408

-1 1

C	4.47771800	0.07679500	-0.91309900
C	3.51091700	1.01746500	-1.27935200
C	2.25985900	1.01873800	-0.65816800
C	1.96376600	0.07556000	0.33667900
C	2.93387600	-0.86365500	0.69726000
C	4.18523400	-0.86879300	0.08192500
H	3.71748400	1.75640100	-2.04740500
C	0.62015100	0.08004500	0.99896200
H	2.68751100	-1.58757000	1.46706200
C	-0.27077200	1.10591600	0.53690900
C	0.05681500	2.05072700	-0.49307600
C	-2.96304700	0.22353300	0.49648300
C	-2.59046800	-0.62206900	-0.55885200
C	-4.30439500	0.21765000	0.92079700
C	-3.52933100	-1.45071000	-1.17897000
H	-1.55792600	-0.62849200	-0.89108700
C	-5.25144300	-0.60698900	0.31038700
H	-4.61141800	0.86693700	1.73679000
C	-4.84292700	-1.42802400	-0.73258500
H	-3.24565400	-2.10749600	-1.99543600
H	-6.28779900	-0.61390300	0.63350600
O	0.36414600	-0.77538200	1.86906800
O	-0.63097500	2.94169500	-0.96869000
O	1.36015900	1.95919600	-1.06091400
S	-1.83055100	1.31021200	1.34774300
F	-5.77037200	-2.24254100	-1.33973100
H	5.44189200	0.09496500	-1.40851500
O	5.07369100	-1.83891100	0.51253600
C	6.35286300	-1.88082600	-0.08580300
H	6.88411600	-2.70667500	0.39270900
H	6.29243600	-2.07075400	-1.16718800
H	6.91246100	-0.94889700	0.08027800

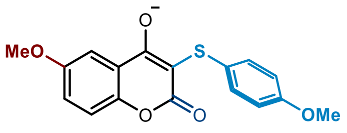

C8

C8⁻

E = -1430.067087

G = -1429.855423

-1 1

C	4.79891100	-0.19578300	-0.98657500
C	3.87872700	0.76735200	-1.41039700
C	2.65013600	0.90164400	-0.75923400
C	2.33002000	0.07049100	0.32417800
C	3.25343800	-0.89177800	0.74214500
C	4.48202700	-1.02938700	0.09703700
H	4.10442800	1.42122600	-2.24719100
C	1.01030600	0.21639300	1.01858800
H	2.98846900	-1.52778700	1.58037700
C	0.16600400	1.24910000	0.48926700
C	0.51765400	2.07852300	-0.62816600
C	-2.58100100	0.54727400	0.57555000
C	-2.29467400	-0.41069600	-0.41074100
C	-3.90729600	0.66661900	1.01107400
C	-3.30037800	-1.21398100	-0.94048100
H	-1.27302400	-0.52136600	-0.75867700
C	-4.92573000	-0.13833800	0.48609100
H	-4.15798400	1.40115400	1.77279100
C	-4.62199500	-1.08514500	-0.49708700
H	-3.07429900	-1.95405000	-1.70277800
H	-5.93842800	-0.00997900	0.85260700
O	0.73507400	-0.54099200	1.97020900
O	-0.12809000	2.96678400	-1.16401000
O	1.79620800	1.85687400	-1.22037700
S	-1.35524200	1.61718100	1.31712300
O	-5.54735200	-1.93051300	-1.08569300
C	-6.89212200	-1.83294800	-0.66249300
H	-7.44725800	-2.57565000	-1.24021900
H	-6.99914500	-2.05567000	0.40884300
H	-7.31029700	-0.83563600	-0.86082100
H	5.74623600	-0.28165600	-1.50691200
O	5.32467400	-2.01131500	0.59001000
C	6.57864900	-2.18577200	-0.03664500
H	7.07510600	-2.99782200	0.49957300
H	6.47158900	-2.46612000	-1.09460800
H	7.19829900	-1.27964800	0.02925300

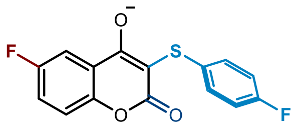

C9

C9⁻

E = -1399.501656
G = -1399.366201
-1 1

C	4.80147500	-0.37102800	-0.94948900
C	3.91128900	0.64364900	-1.29568800
C	2.66215600	0.72258800	-0.66192600
C	2.29587400	-0.20998100	0.31724600
C	3.19422400	-1.23129000	0.66205600
C	4.41966000	-1.28973900	0.02719100
H	4.16465800	1.38084300	-2.05075900
C	0.96038100	-0.11621300	0.98678500
H	2.89898900	-1.94864000	1.42036000
C	0.14794100	0.98215500	0.54898600
C	0.54182000	1.91640300	-0.46602100
C	-2.60445400	0.31177700	0.50707000
C	-2.30464400	-0.53523100	-0.56978400
C	-3.93909400	0.39901800	0.94291900
C	-3.30842900	-1.27557100	-1.19994700
H	-1.27823600	-0.61326100	-0.91165900
C	-4.95051900	-0.33686300	0.32262200
H	-4.18991100	1.05092100	1.77580600
C	-4.61324700	-1.16276200	-0.74180800
H	-3.08157000	-1.93322400	-2.03324200
H	-5.98203100	-0.27212100	0.65452700
O	0.64800500	-0.96673400	1.84296000
O	-0.07325200	2.86498200	-0.92574100
O	1.83737700	1.73283900	-1.04344500
S	-1.38748600	1.29064200	1.37340300
F	-5.60400400	-1.88978100	-1.35849900
H	5.77431800	-0.45610200	-1.42267200
F	5.30510500	-2.28675900	0.36091400

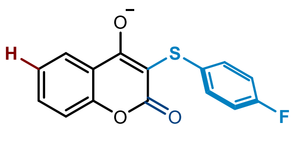

C10

C10⁻
E = -1300.259242
G = -1300.114032

-1 1

C	5.03610800	-1.01032500	-0.75932700
C	4.24565600	0.03426300	-1.23191300
C	2.99923700	0.28263100	-0.63616400
C	2.54117300	-0.50783600	0.42604900
C	3.35222600	-1.55575400	0.88555700
C	4.59180000	-1.81207700	0.30460200
H	4.56695200	0.66726500	-2.05343100
C	1.21036700	-0.23048100	1.05049500
H	2.96850000	-2.15094500	1.70878500
H	5.21128000	-2.62679000	0.67043400
C	0.49926500	0.87995900	0.48189000
C	0.98623500	1.66452900	-0.61473200
C	-2.29861400	0.43262100	0.45362500
C	-2.05507400	-0.55162100	-0.51563200
C	-3.62760000	0.67460400	0.84662900
C	-3.10762400	-1.27550100	-1.08208400
H	-1.03373900	-0.74901600	-0.82299700
C	-4.68764900	-0.04314800	0.28928000
H	-3.83524100	1.43420200	1.59600200
C	-4.40489700	-1.00795400	-0.66882600
H	-2.92421700	-2.03882600	-1.83185000
H	-5.71481400	0.14100200	0.58843400
O	0.80908500	-0.95356000	1.98413000
O	0.46252500	2.60817400	-1.18616000
O	2.26985600	1.31247100	-1.13859500
S	-1.01627600	1.40080200	1.23266200
F	-5.44395400	-1.71864400	-1.22318400
H	6.00191400	-1.20206200	-1.22101700

G. References

1. (a) Folgueiras-Amador, A. A.; Teuten, A. E.; Salam-Perez, M.; Pearce, J. E.; Denuault, G.; Pletcher, D.; Parsons, P. J.; Harrowven, D. C.; Brown, R. C. D. Cathodic Radical Cyclisation of Aryl Halides Using a Strongly-Reducing Catalytic Mediator in Flow. *Angew. Chem., Int. Ed.* **2022**, *61*, e202203694. (b) Su, H.; Li, F.; Xuan, Z.; Yu, W. Copper-Catalyzed Cyclization and Azidation of γ,δ-Unsaturated Ketone *O*-Benzoyl Oximes. *Adv. Synth. Catal*. **2015**, *357*, 64–70. (c) Okamoto, R.; Tanaka, K. Rhodium-Catalyzed Olefin Isomerization/Allyl Claisen Rearrangement/Intramolecular Hydroacylation Cascade. *Org. Lett*. **2013**, *15*, 2112−2115.

2. MacKenzie, I. A.; Wang, L.; Onuska, N. P. R.; Williams, O. F.; Begam, K.; Moran, A. M.; Dunietz, B. M.; Nicewicz, D. Discovery and characterization of an acridine radical photoreductant. *Nature*. **2020**, *580*, 76–80.

3. Neveselý, T.; Molloy, J.J.; McLaughlin, C.; Brüss, L.; Daniliuc, C.G.; Gilmour, R. Leveraging the n→π* Interaction in Alkene Isomerization by Selective Energy Transfer Catalysis. Angew. *Chem., Int. Ed.* **2022**, *61*, e202113600.

4. Too, P. C.; Wang, Y. F.; Chiba, S. Rhodium (III)-catalyzed synthesis of isoquinolines from aryl ketone O-acyloxime derivatives and internal alkynes. *Org. Lett*. **2010**, *12*, 5688−5691.

5. Onneken, C.; Bussmann, K.; Gilmour, R. Inverting External Asymmetric Induction via Selective Energy Transfer Catalysis: A Strategy to β-Chiral Phosphonate Antipodes. *Angew. Chem., Int. Ed*. **2020**, *132*, 338−342.

6. Mallik, S.; Sfreddo, E.; Wang, H.; Melchiorre, P. Radical pathways for 2,4-chromandione synthesis via photoexcitation of 4-hydroxycoumarins. *Chem. Sci*. **2025**, *16*, 124−129.

7. Parumalaa, S. K. R.; Peddinti, R. K. Iodine catalyzed cross-dehydrogenative C–S coupling by C(sp^2)–H bond activation: direct access to aryl sulfides from aryl thiols. *Green Chem*. **2015**, *17*, 4068−4072.

8. Schiel, F.; Peinsipp, C.; Kornigg, S.; Böse, D. A 3D-Printed Open Access Photoreactor Designed for Versatile Applications in Photoredox- and Photoelectrochemical Synthesis. *ChemPhotoChem*. **2021**, *5*, 431–437.

9. Kuhlmann, J. H.; Dickoff, J. H.; Mancheño, O. G. Visible Light Thiyl Radical-Mediated Desilylation of Arylsilanes. *Chem. Eur. J.* **2023**, *29*, e202203347.

10. Yamada, Y. M. A.; Watanabe, T.; Ohno, A.; Uozumi, Y. Development of Polymeric Palladium-Nanoparticle Membrane-Installed Microflow Devices and their Application in Hydrodehalogenation. *ChemSusChem*. **2012**, *5*, 293-299.

11. Wu, S.; Schiel, F.; Melchiorre, P. A General Light-Driven Organocatalytic Platform for the Activation of Inert Substrates. *Angew. Chem., Int. Ed*. **2023**, *62*, e202306364.

12. Kanchupalli, V.; Joseph, D.; Katukojvala, S. Pyridazine *N*-Oxides as Precursors of Metallocarbenes: Rhodium-Catalyzed Transannulation with Pyrroles. *Org. Lett.* **2015**, *17*, 5878−5881.

13. Garcia, N.; Garcia-Garcia, P.; Fernandez-Rodriguez, M. A.; Rubio, R.; Pedrosa, M. R.;

Arnaiz, F. J.; Sanz, R. Pinacol as a New Green Reducing Agent: Molybdenum-Catalyzed Chemoselective Reduction of Sulfoxides and Nitroaromatics. *Adv. Synth. Catal.* **2012**, *354*, 321–327.

14. Zhao, J.-H.; Zhou, Z.-Z.; Zhang, Y.; Su, X.; Chen, X.-M.; Liang, Y.-M. Visible-light-mediated borylation of aryl and alkyl halides with a palladium complex. *Org. Biomol. Chem.* **2020**, *18*, 4390–4394
15. Fan, Y.; Kang, D. W.; Labalme, S.; Li, J.; Lin, W. Enhanced Energy Transfer in A π-Conjugated Covalent Organic Framework Facilitates Excited-State Nickel Catalysis. *Angew. Chem., Int. Ed.* **2023**, *62*, e202218908.
16. Zhao, Y. L.; Wu, G. J.; Li, Y.; Gao, X. L.; Han, F. S. [$NiCl_2$(dppp)]-Catalyzed Cross-Coupling of Aryl Halides with Dialkyl Phosphite, Diphenylphosphine Oxide, and Diphenylphosphine. *Chem. Eur. J.*, **2012**, *18*, 9622– 9627.
17. Liang, G.; Wang, J-H.; Lei, T.; Cheng, Y-Y.; Zhou, C.; Chen, Y-J.; Ye, C.; Chen, B.; Tung, C-H.; Wu, L-Z. Direct C–H Thiolation for Selective Cross-Coupling of Arenes with Thiophenols via Aerobic Visible-Light Catalysis. *Org. Lett.* **2021**, *23*, 8082−8087.
18. Nikitin, M.; Babawale, F.; Tastekin, S.; Antonietti, M.; Ghosh, I.; König, B. C(sp^2)–S cross-coupling reactions with nickel, visible light, and mesoporous graphitic carbon nitride. *Green Chem.* **2024**, *26*, 5845−5851.
19. Brigham, C. E.; Malapit, C. A.; Lalloo, N.; Sanford, M. S. Nickel-Catalyzed Decarbonylative Synthesis of FluoroalkylThioethers. *ACS Catal.* **2020**, *10*, 8315−8320.
20. Li, S-Y.; Yang, X-Y.; Shen, P-H.; Xu, L.; Xu, J.; Zhang, Q.; Xu, H-J. Selective Defluoroalkylation and Hydrodefluorination of Trifluoromethyl Groups Photocatalyzed by Dihydroacridine Derivatives. *J. Org. Chem.* **2023**, *88*, 17284−17296.
21. Wang, H.; Jui, N. T. Catalytic Defluoroalkylation of Trifluoromethylaromatics withUnactivated Alkenes. *J. Am. Chem. Soc.* **2018***, 140,* 163−166.
22. Szostak, M.; Spain, M.; Procter, D. J. Determination of the Effective Redox Potentials of SmI_2, $SmBr_2$, $SmCl_2$, and their Complexes with Water by Reduction of Aromatic Hydrocarbons. Reduction of Anthracene and Stilbene by samarium (II) Iodide−Water Complex. *J. Org. Chem.* **2014**, *79*, 2522−2537,
23. Chatterjee, A.; König, B. Birch-Type Photoreduction of Arenes and Heteroarenes by Sensitized Electron Transfer. *Angew. Chem., Int. Ed.* **2019**, *58*, 14289–14294.
24. Peng, Y.; Wang, G.; Klare, H. F. T. Oestreich, M., Ring Contraction of Saturated Cyclic Amines and Rearrangement of Acyclic Amines Through Their Corresponding Hydroxylamines. *Angew. Chem. Int. Ed.* **2024**, *63*, e202410483.
25. Cowper, N. G. W.; Chernowsky, C. P.; Williams, O. P.; Wickens, Z. K. Potent Reductants via Electron-Primed Photoredox Catalysis: Unlocking Aryl Chlorides for Radical Coupling. *J. Am. Chem. Soc.* **2020**, *142*, 2093−2099.
26. Usami, K.; Yamaguchi, E.; Tada, N.; Itoh, A. Visible-Light-Mediated Iminyl Radical Generation from BenzylOxime Ether: Synthesis of Pyrroline via Hydroimination

Cyclization. *Org. Lett*. **2018**, *20*, 5714−5717.

27. Liang, K.; Liu, Q.; Shen, L.; Li, X.; Wei, D.; Zheng, L.; Xia, C. Intermolecular oxyarylation of olefins with aryl halides and TEMPOH catalyzed by the phenolate anion under visible light. *Chem. Sci*., **2020**,*11*, 6996−7002.

28. Nevesely´, T.; Molloy, J. J.; McLaughlin, C.; Brüss, L.; Daniliuc, C. G.; Gilmour, R. Leveraging the n-p* Interaction in Alkene Isomerization by SelectiveEnergy Transfer Catalysis. *Angew. Chem., Int. Ed*. **2022**, *61*, e202113600.

29. Kaplaneris, N.; Bisticha, A.; Papadopoulos, G. N.; Limnios, D.; Kokotos, C. G. Photoorganocatalytic synthesis of lactones via a selective C–H activation–alkylation of alcohols. *Green Chem*. **2017**,*19*, 4451−4456.

30. Noverges, B.; Mollar, C.; Medio-Simon, M.; Asensio, G. Palladium-Catalyzed Suzuki–Miyaura Cross-Coupling of α-Halomethyl Oxime Ethers and Site-Selective Cross-Coupling of Dihalo Derivatives. *Adv. Synth. Catal*. **2013**, *355*, 2327–2342.

31. Voloshkin, V. A.; Villa, M.; Martynova, E. A.; Beliš, M.; Van Hecke, K.; Ceroni, P.; Nolan, S. P. Synthesis of cyclobutane-fused chromanones via gold-mediated photocatalysis, *Chem. Sci.* **2024**, *15*, 4571−4580.

32. Daub, M. E.; Jung, H.; Lee, B. J.; Won, J.; Baik, M. H.; Yoon, T. P. Enantioselective [2+2] Cycloadditions of Cinnamate Esters: Generalizing Lewis Acid Catalysis of Triplet Energy Transfer, *J. Am. Chem. Soc*. **2019**, *141*, 9543−9547.

33. Lu, Z.; Yoon, T. P. Visible Light Photocatalysis of [2+2] Styrene Cycloadditions by Energy Transfer, *Angew. Chem., Int. Ed*. **2012**, *51*, 10329−10332.

34. Schmid, L.; Glaser, F.; Schaer, R.; Wenger, O. S. High Triplet Energy Iridium(III) Isocyanoborato Complex for Photochemical Upconversion, Photoredox and Energy Transfer Catalysis. *J. Am. Chem. Soc*. **2022**, *144*, 963–976.

35. Cismesia, M. A.; Yoon, T. P. Characterizing chain processes in visible light photoredox catalysis. *Chem. Sci.,* **2015**, *6*, 5426−5434.

36. Leeuwen, T. V.; Buzzetti, L.; Perego, L. A.; Melchiorre, P. A Redox-Active Nickel Complex that Acts as an Electron Mediator in Photochemical Giese Reactions. *Angew. Chem., Int. Ed*. **2019**, *58*, 4953−4957.

37. Gehlen, M. H. The centenary of the Stern–Volmer equation of fluorescence quenching: From the single line plot to the SV quenching map. *J. Photochem. Photobiol.*, C **2020**, *42*, 100338.

38. Doherty, A. P.; Scott, K. The electrochemical reduction of dimethyl maleate and dimethyl fumarate. *J. Electroanal. Chem.* **1998**, *442*, 35–40.

39. Ladányi, V.; Dvořák, P.; Anshori, J. A.; Vetráková, L.; Wirz, J.; Heger, D. Azobenzene photoisomerization quantum yields in methanol redetermined. *Photochem. Photobiol. Sci*., **2017**,*16*, 1757−1761.

40. V. V. Pavlishchuk, A. W. Addison., Conversion constants for redox potentials measured versus different reference electrodes in acetonitrile solutions at 25°C. *Inorganica Chim.*

Acta. **2000**, *298*, 97–102.

41. Frisch, M. J.; Trucks, G. W.; Schlegel, H. B.; Scuseria, G. E.; Robb, M. A.; Cheeseman, J. R.; Scalmani, G.; Barone, V.; Petersson, G. A.; Nakatsuji, H.; Li, X.; Caricato, M.; Marenich, A. V.; Bloino, J.; Janesko, B. G.; Gomperts, R.; Mennucci, B.; Hratchian, H. P.; Ortiz, J. V.; Izmaylov, A. F.; Sonnenberg, J. L.; Williams-Young, D.; Ding, F.; Lipparini, F.; Egidi, F.; Goings, J.; Peng, B.; Petrone, A.; Henderson, T.; Ranasinghe, D.; Zakrzewski, V. G.; Gao, J.; Rega, N.; Zheng, G.; Liang, W.; Hada, M.; Ehara, M.; Toyota, K.; Fukuda, R.; Hasegawa, J.; Ishida, M.; Nakajima, T.; Honda, Y.; Kitao, O.; Nakai, H.; Vreven, T.; Throssell, K.; Montgomery, J. A., Jr.; Peralta, J. E.; Ogliaro, F.; Bearpark, M. J.; Heyd, J. J.; Brothers, E. N.; Kudin, K. N.; Staroverov, V. N.; Keith, T. A.; Kobayashi, R.; Normand, J.; Raghavachari, K.; Rendell, A. P.; Burant, J. C.; Iyengar, S. S.; Tomasi, J.; Cossi, M.; Millam, J. M.; Klene, M.; Adamo, C.; Cammi, R.; Ochterski, J. W.; Martin, R. L.; Morokuma, K.; Farkas, O.; Foresman, J. B.; Fox, D. J. *Gaussian 16*, Revision C.01; *Gaussian, Inc., Wallingford CT*, **2016**.

42. (a) Becke, A. D. A new mixing of Hartree–Fock and local density-functional theories. *J. Chem. Phys*. **1993**, *98*, 1372–1377; (b) Lee, C.; Yang, W.; Parr., R. G. Development of the Colle-Salvetti correlation-energy formula into a functional of the electron density. *Phys. Rev. B*. **1988**, *37*, 785-789; (c) Hariharan, P. C.; Pople, J. A.; Theor The influence of polarization functions on molecular orbital hydrogenation energies. *Chim. Acta*. **1973**, *28*, 213-222; (d) Francl, M. M.; Petro, W. J.; Hehre, W. J.; Binkley, J. S.; Gordon, M. S.; DeFrees, D. J.; Pople, J. A. Self-consistent molecular orbital methods. XXIII. A polarization-type basis set for second-row elements. *J. Chem. Phys*. **1982**, *77*, 3654-3665.

43. (a) (a) Miertuš, S.; Scrocco, E.; Tomasi, J. Electrostatic Interaction of a Solute with a Continuum. A Direct Utilization of ab Initio Molecular Potentials for the Prediction of Solvent Effects. *Chem. Phys.* **1981**, *55*, 117–129. (b) Miertuš, S.; Tomasi, J. Approximate Evaluations of the Electrostatic Free Energy and Internal Energy Changes in Solution Processes. *Chem. Phys.* **1982**, *65*, 239–245. (c) Pascual-Ahuir, J. L.; Silla, E.; Tuñón, I. GEPOL: An Improved Description of Molecular Surfaces. III. A New Algorithm for the Computation of a Solvent-Excluding Surface. *J. Comput. Chem.* **1994**, *15*, 1127–1138.

44. Hendy, C. M.; Smith, G. C.; Xu, Z.; Lian, T.; Jui, N. T. Radical Chain Reduction via Carbon Dioxide Radical Anion ($CO\ 2^{\bullet-}$). *J. Am. Chem. Soc*. **2021**, *143*, 8987−8992.

45. Chmiel, A. F.; Williams, O. P.; Chernowsky, C. P.; Yeung, C. S.; Wickens, Z. L. Alkene Carboxy-Alkylation via $CO2^{\bullet-}$. *J. Am. Chem. Soc.* **2021**, *143*, 10882–10889.

46. Zhang, L.; Jiao, L. Pyridine-Catalyzed Radical Borylation of Aryl Halides. *J. Am. Chem. Soc.* **2019**, *141*, 9124–9128.

47. Xu, J.; Cao, J.; Wu, X.; Wang, H.; Yang, X.; Tang, X.; Toh, R. W.; Zhou, R.; Yeow, E. K. L.; Wu, J. Unveiling Extreme Photoreduction Potentials of Donor-Acceptor Cyanoarenes to Access Aryl Radicals from Aryl Chlorides. *J. Am. Chem. Soc*. **2021**, *143*, 13266–13273.

H. NMR Spectra

Substrates

^{1}H NMR (400 MHz, $CDCl_3$)

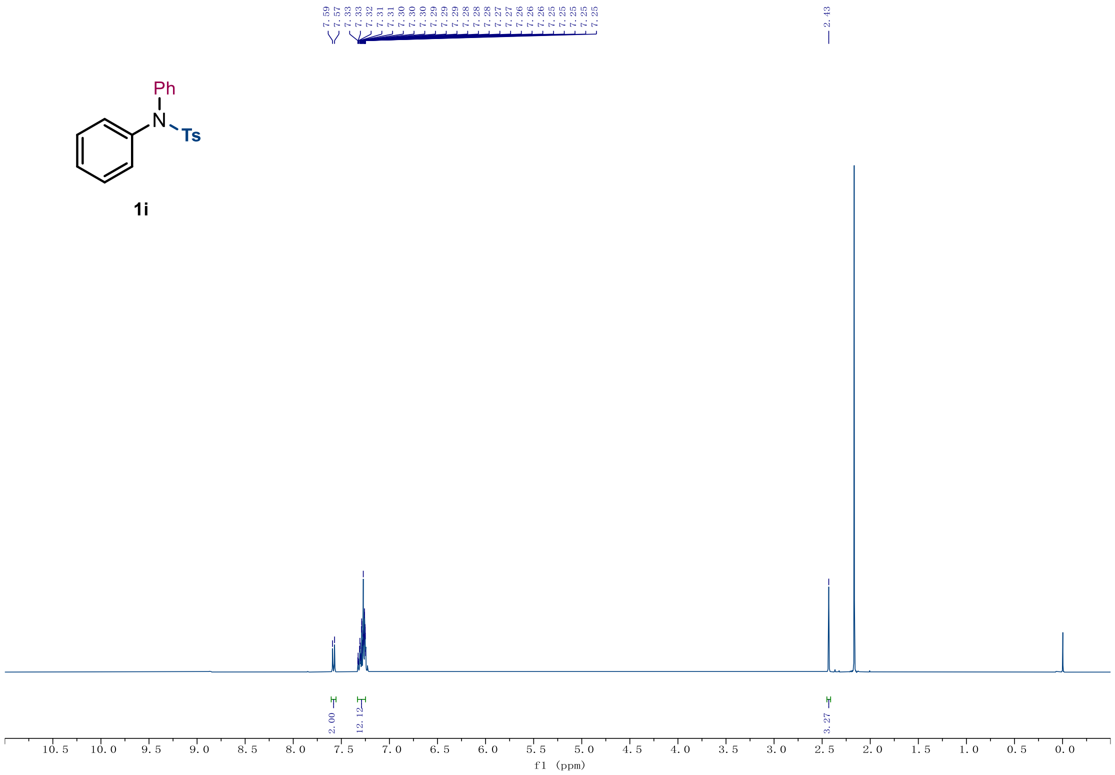

1i

^{1}H NMR (400 MHz, $CDCl_3$)

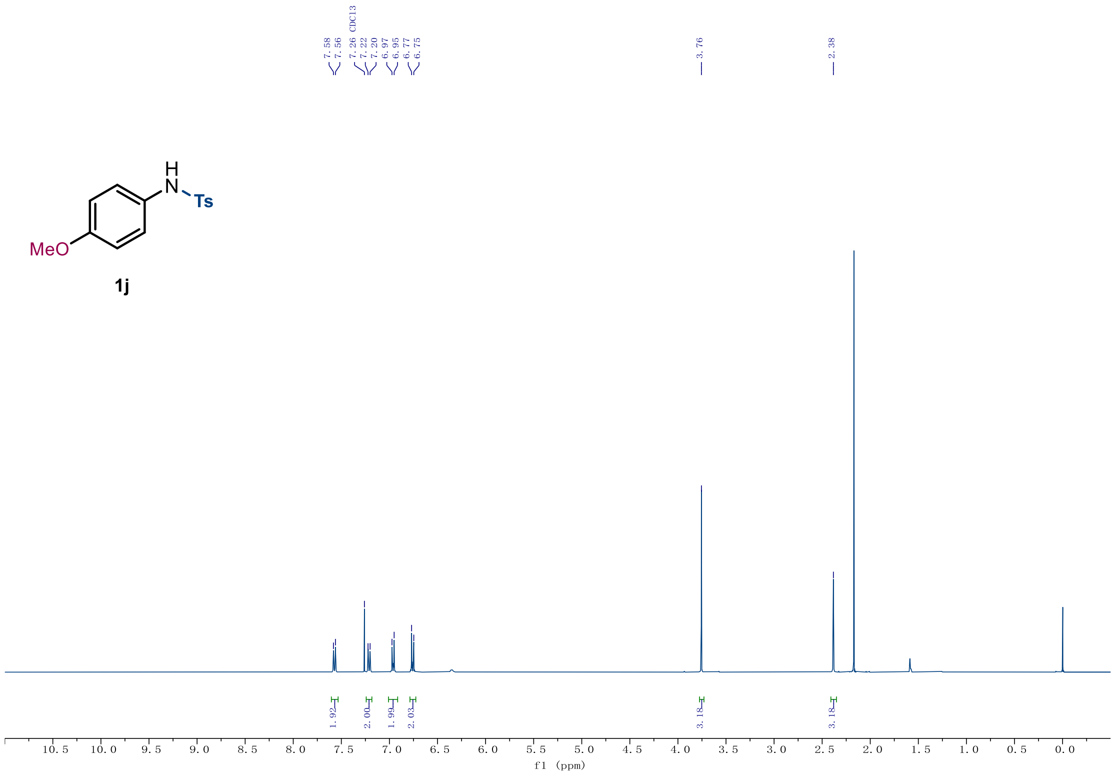

1j

Catalysts C

^{1}H NMR (600 MHz, DMSO)

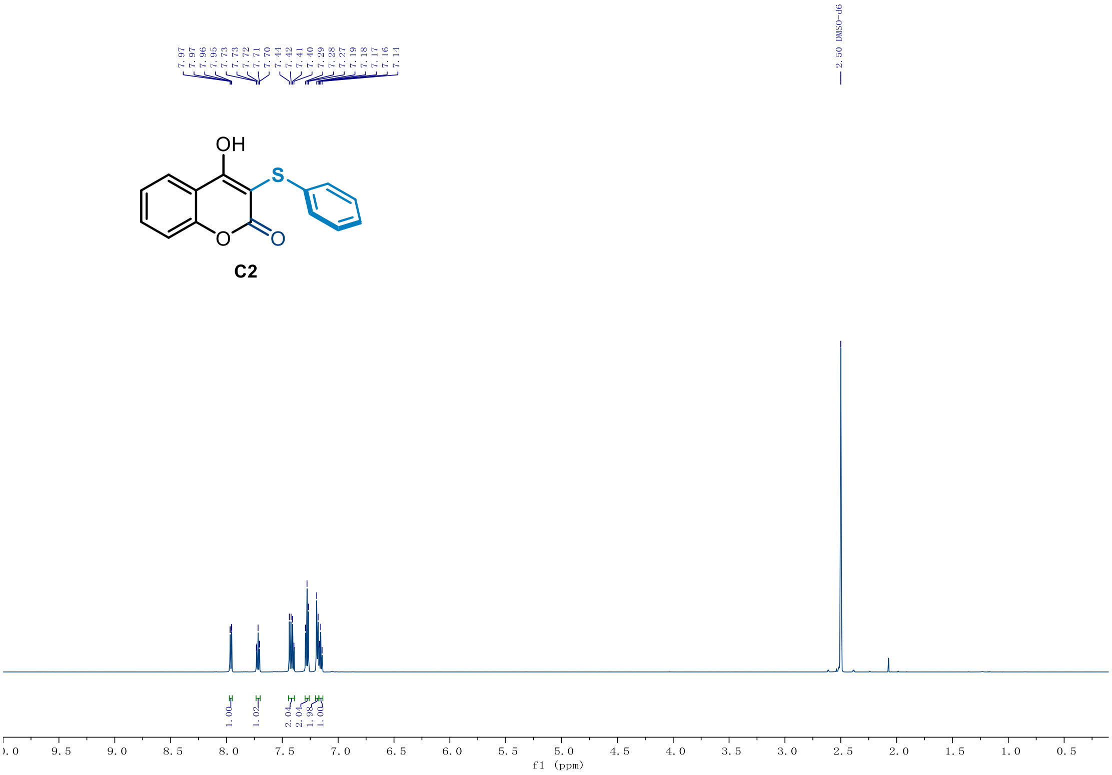

^{13}C NMR (151 MHz, DMSO)

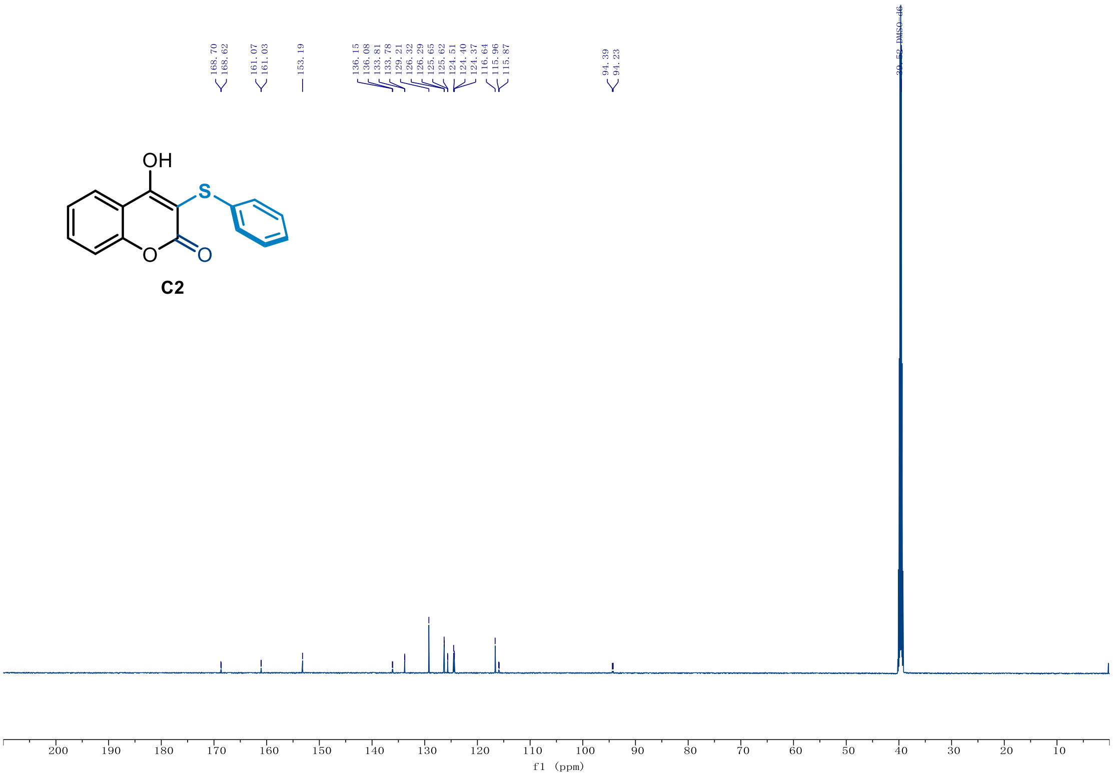

^{1}H NMR (600 MHz, DMSO)

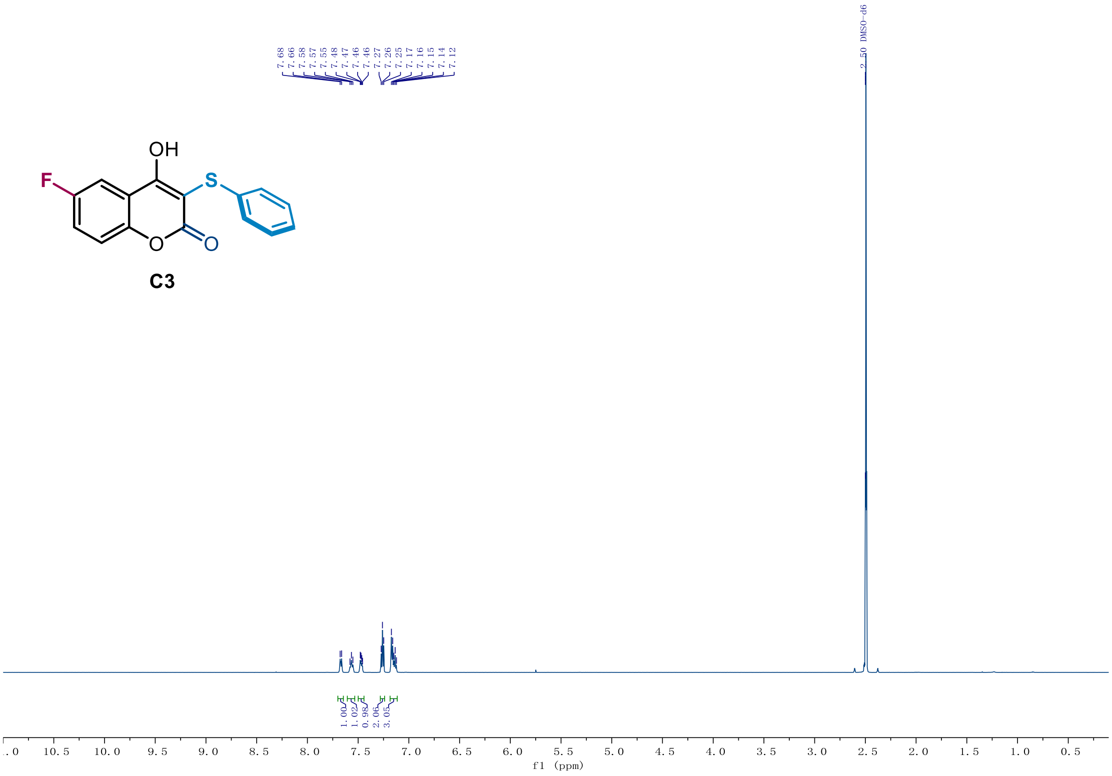

^{13}C NMR (151 MHz, DMSO)

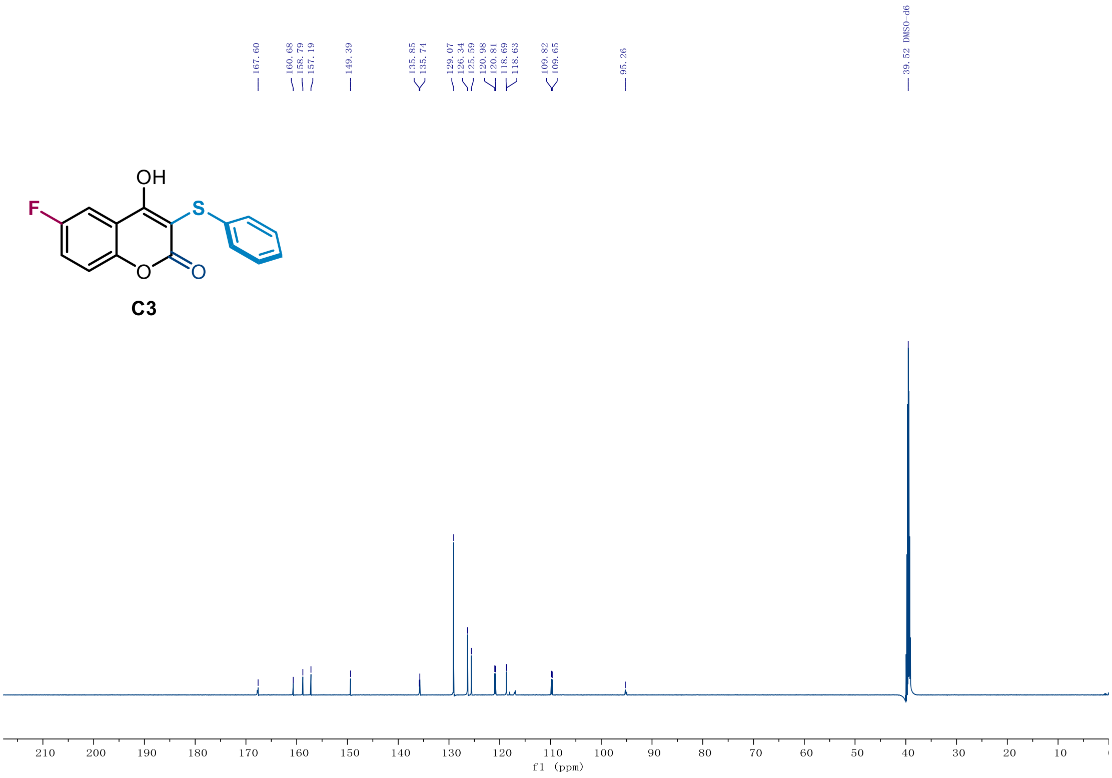

^{19}F NMR (376 MHz, DMSO)

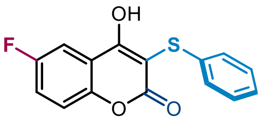

C3

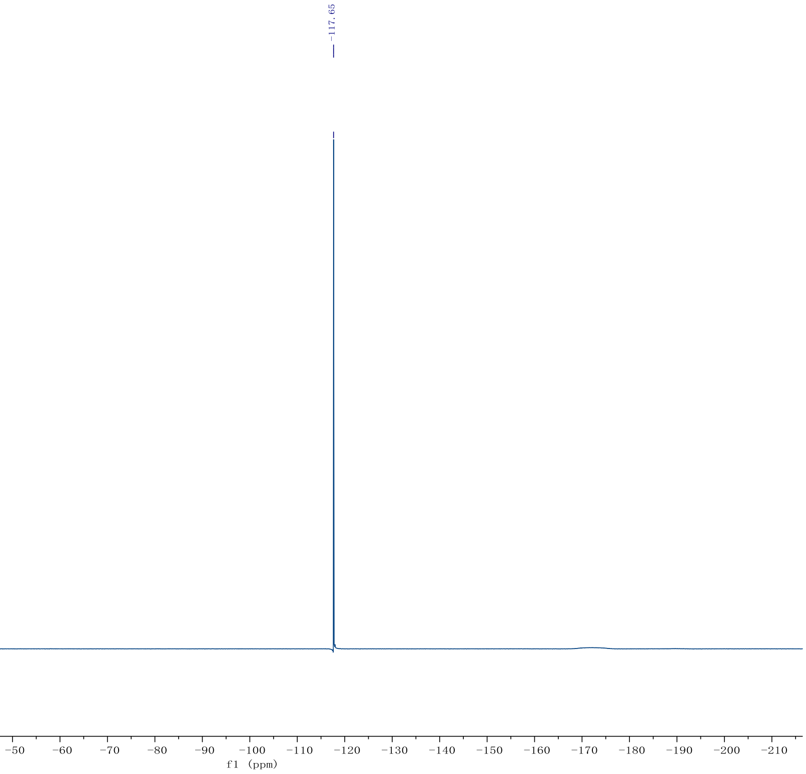

^{1}H NMR (600 MHz, DMSO)

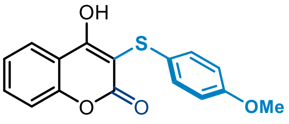

C4

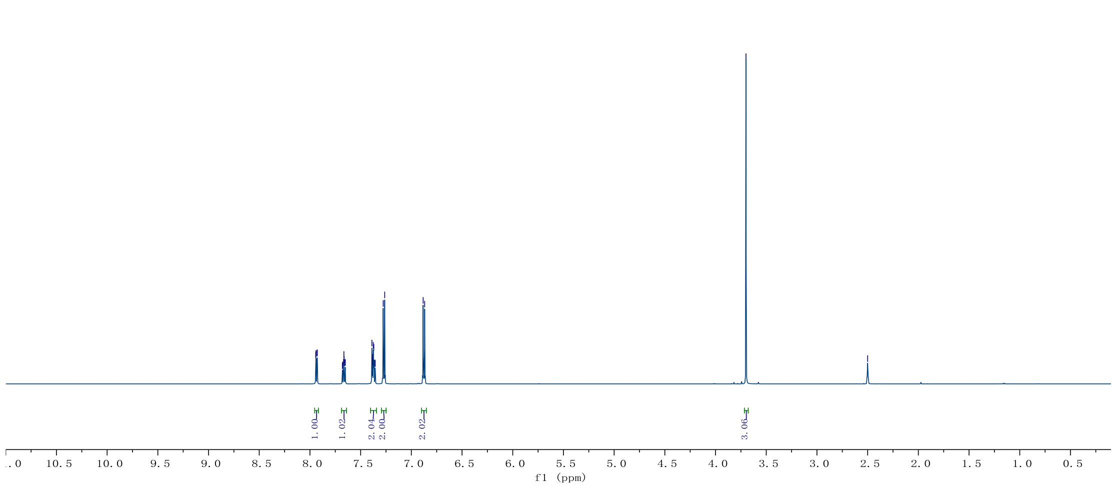

^{13}C NMR (151MHz, DMSO)

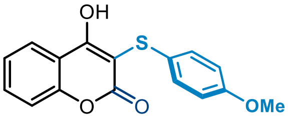

C4

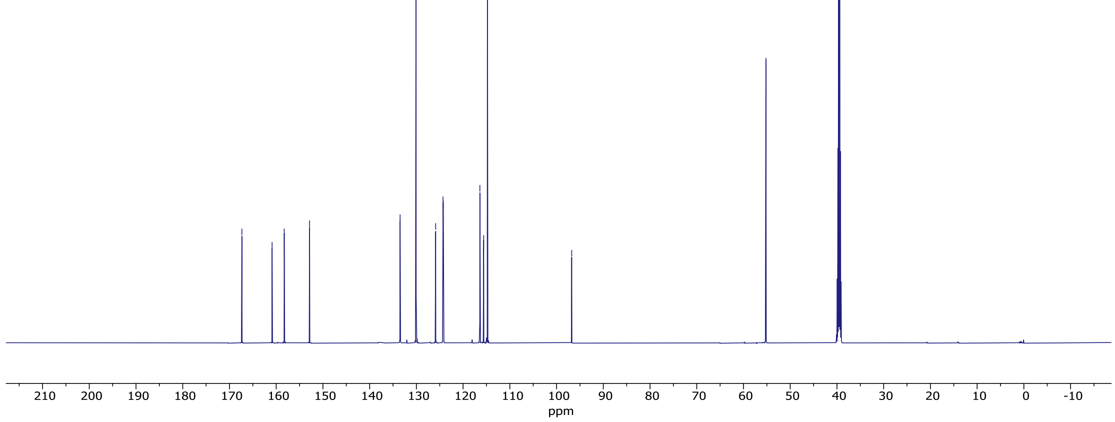

^{1}H NMR (600 MHz, DMSO)

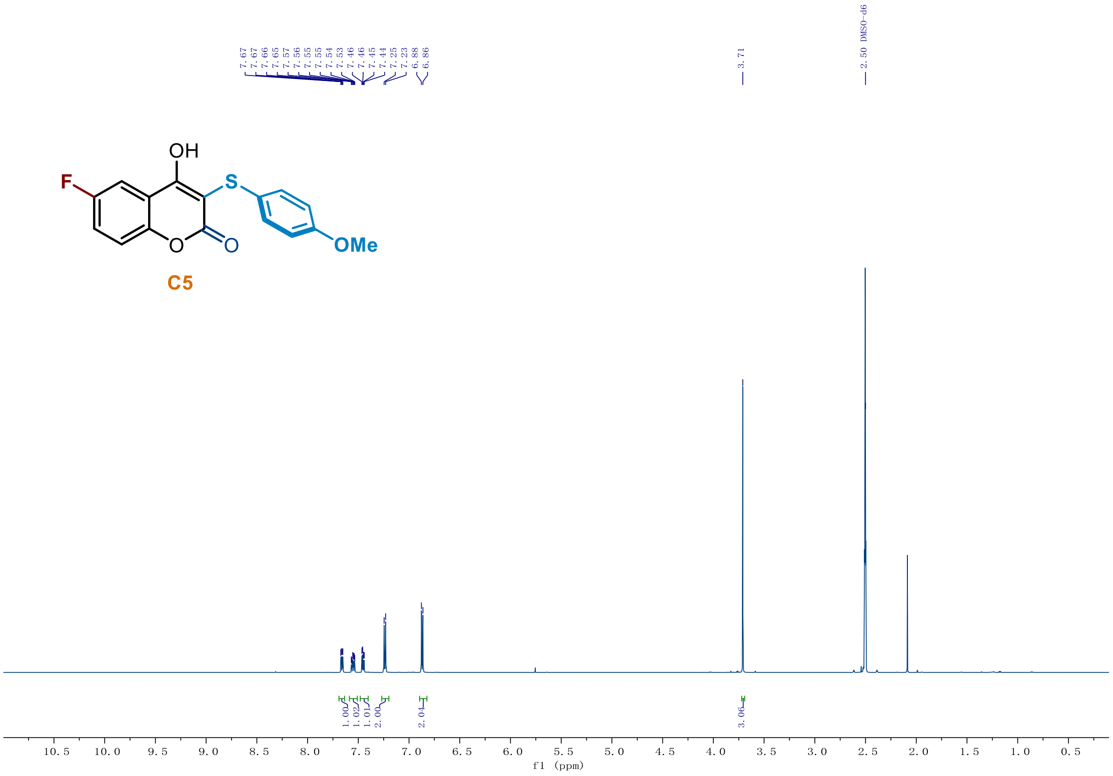

^{19}F NMR (376 MHz, DMSO)

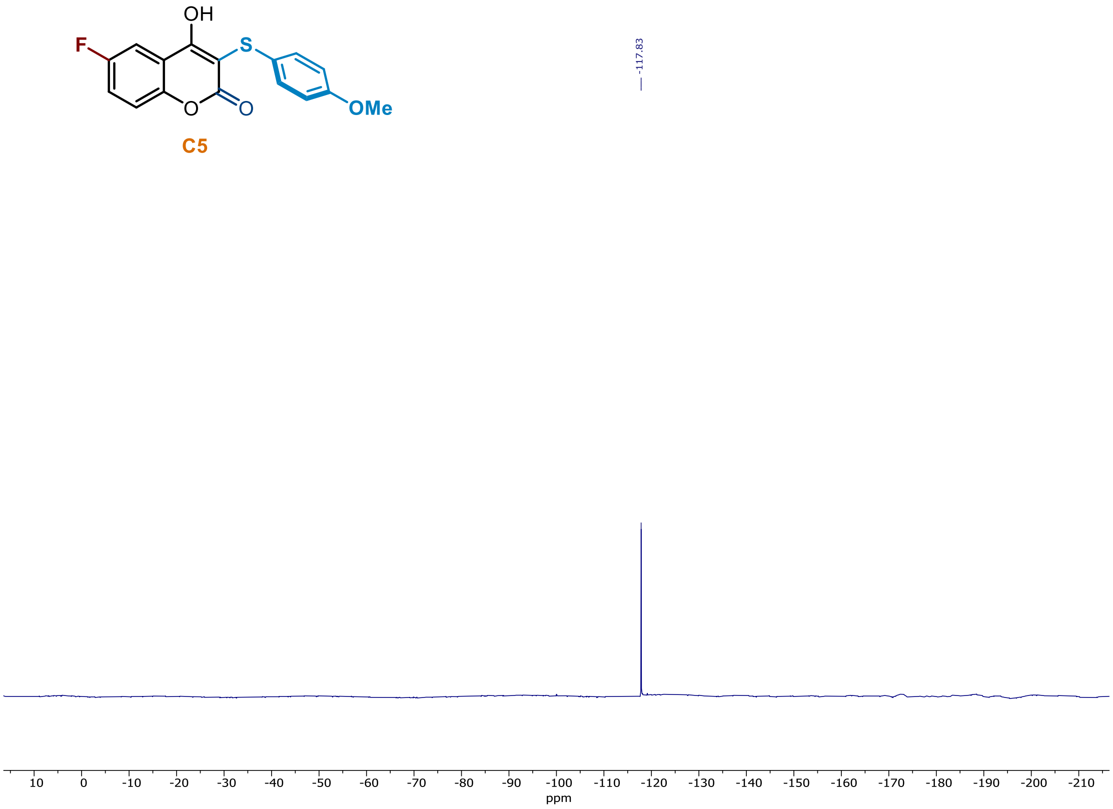

^{13}C NMR (151 MHz, DMSO)

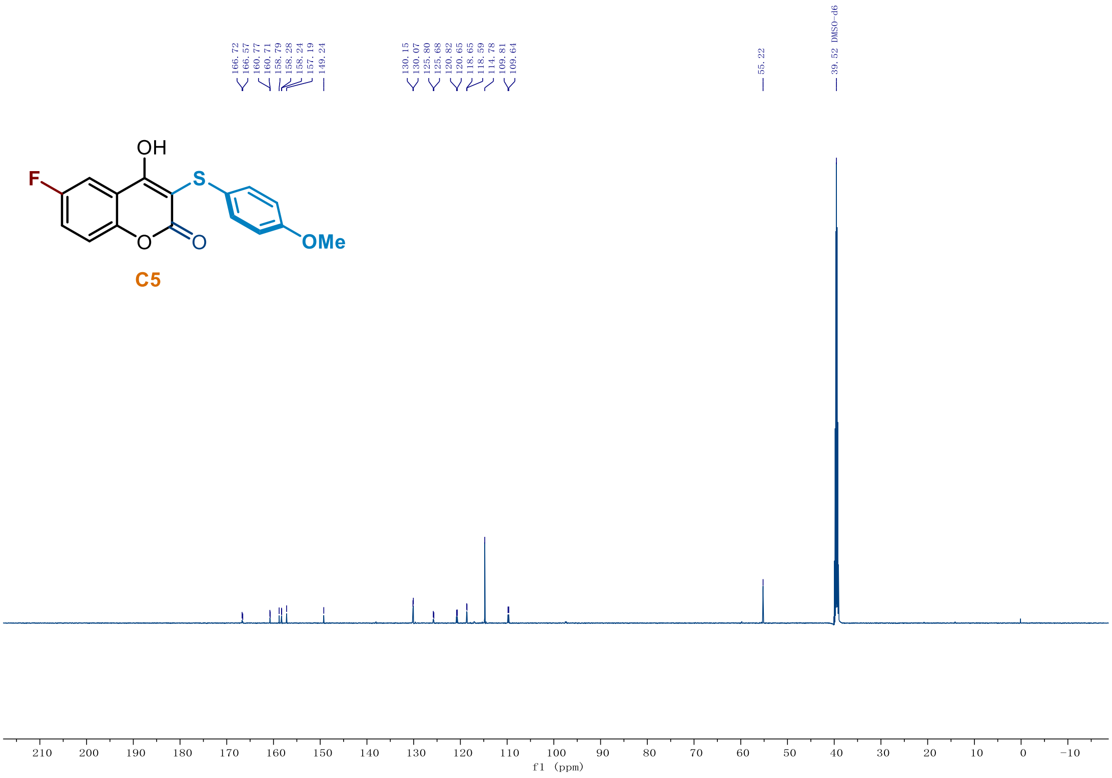

SET-based Processes – Reduction *(Figure 5a in the main manuscript)*

Crude reaction mixture - <u>^{1}H NMR</u> (600 MHz, $CDCl_3$)

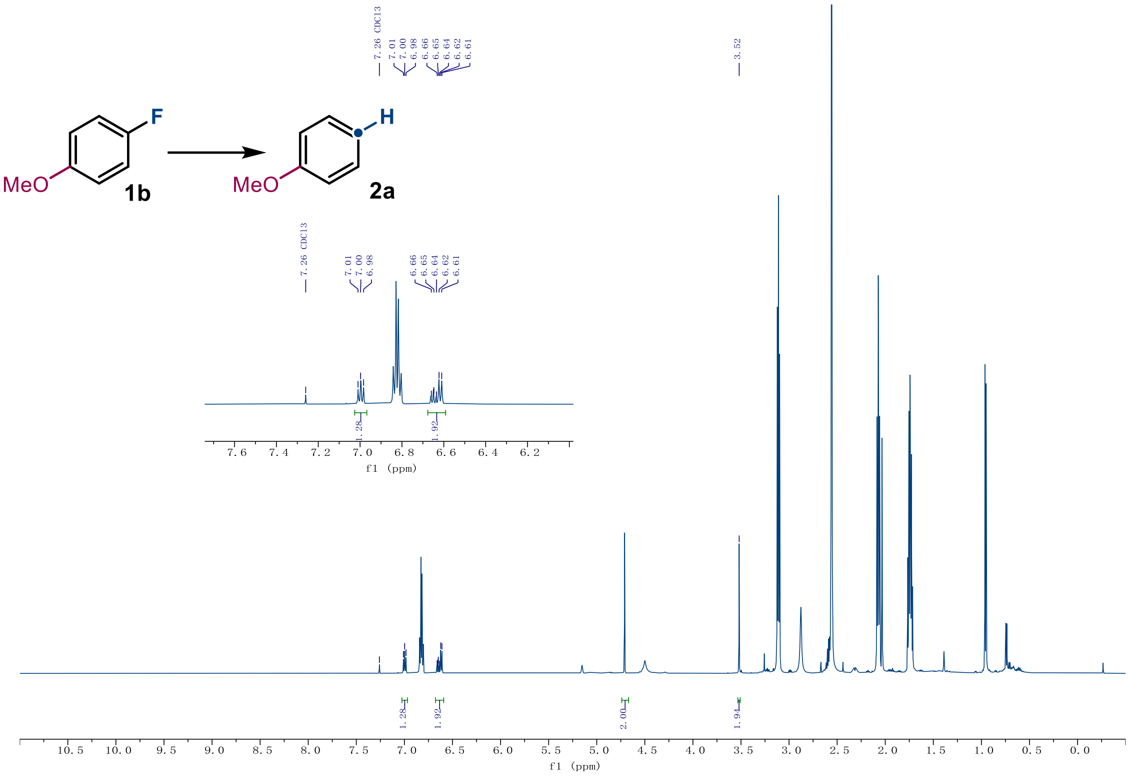

Crude reaction mixture - <u>^{1}H NMR</u> (600 MHz, $CDCl_3$)

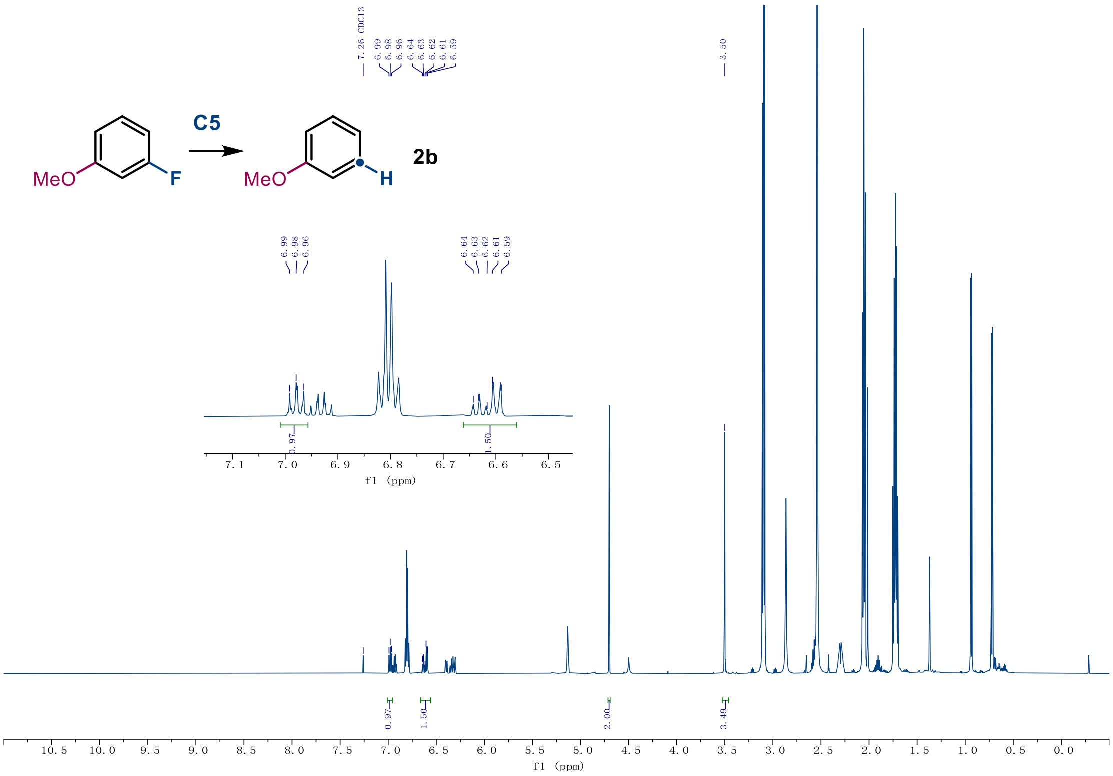

Crude reaction mixture - ^{1}H NMR (600 MHz, $CDCl_3$)

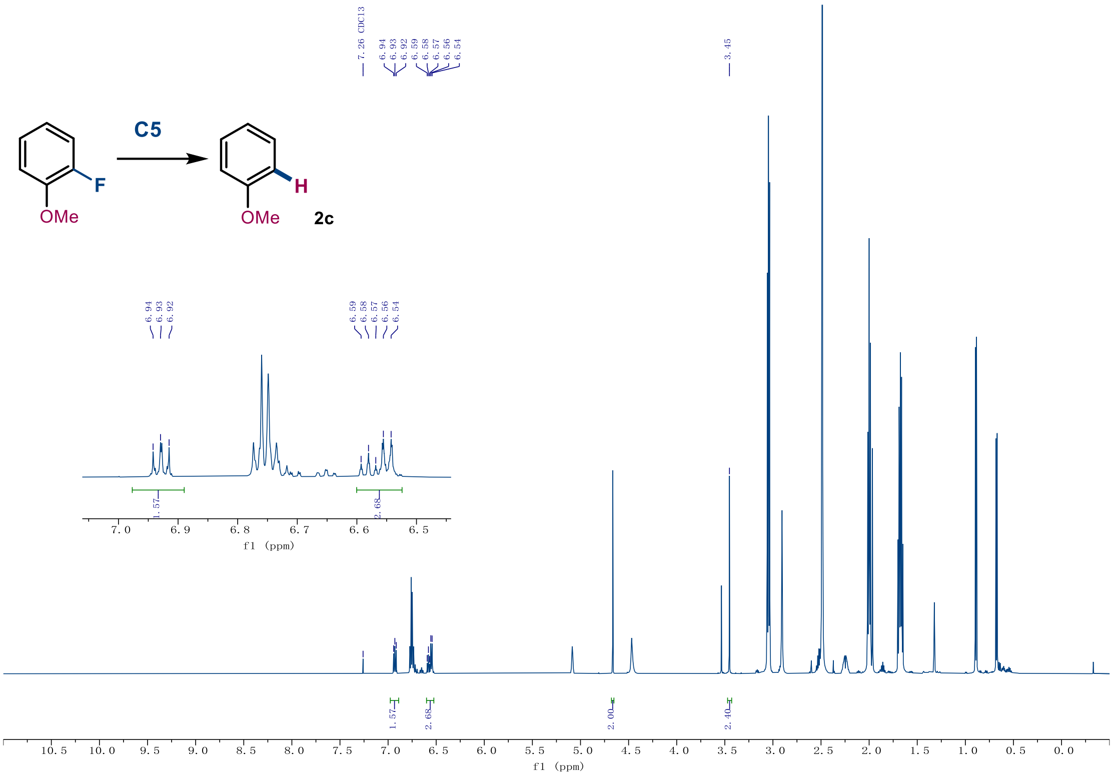

Crude reaction mixture - ^{1}H NMR (600 MHz, $CDCl_3$)

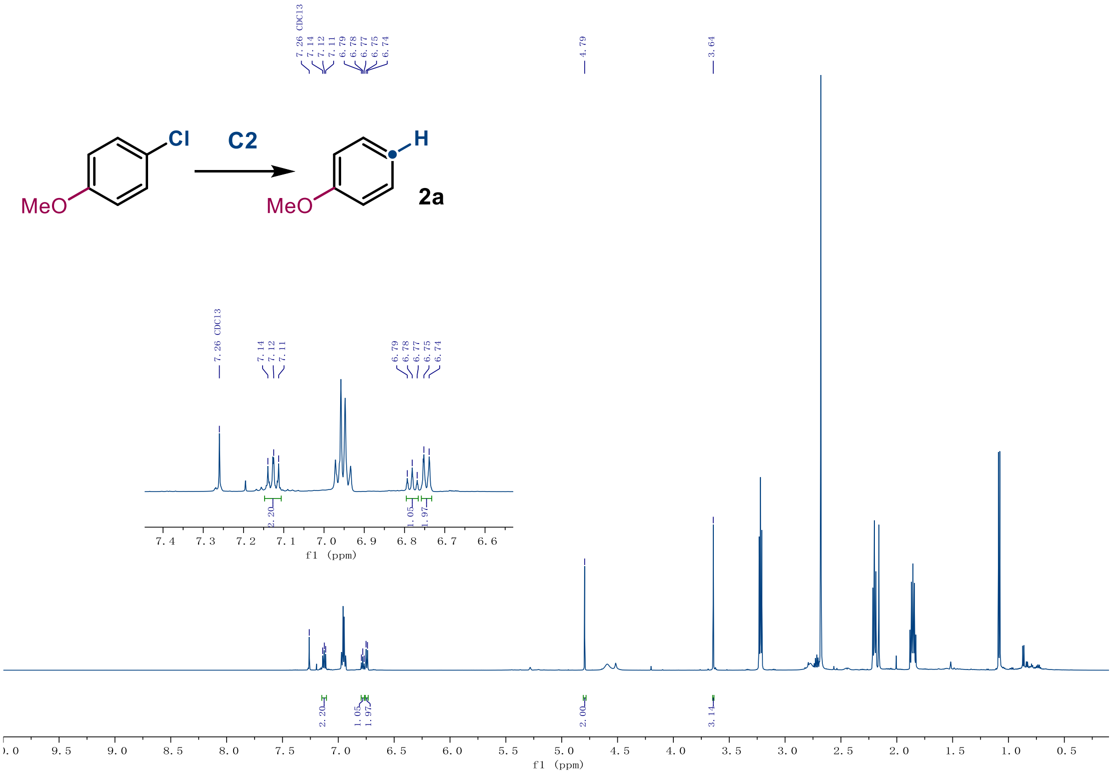

Crude reaction mixture - ^{1}H NMR (500 MHz, $CDCl_3$)

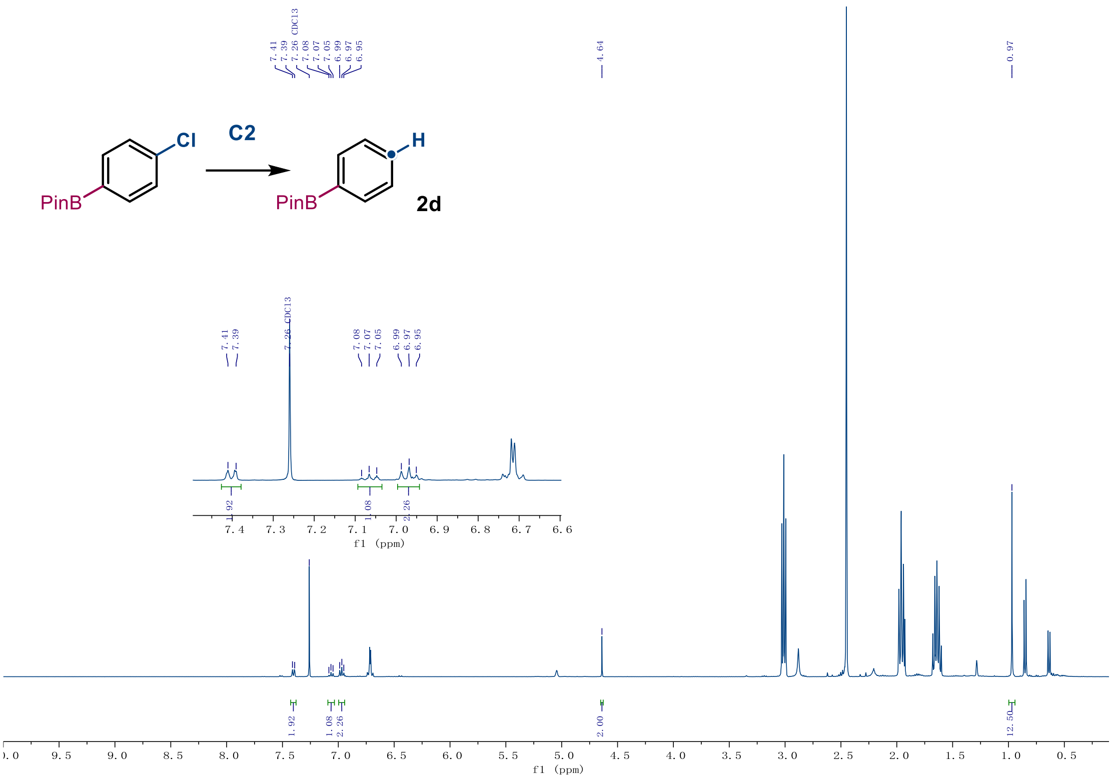

Crude reaction mixture - ^{1}H NMR (600 MHz, $CDCl_3$)

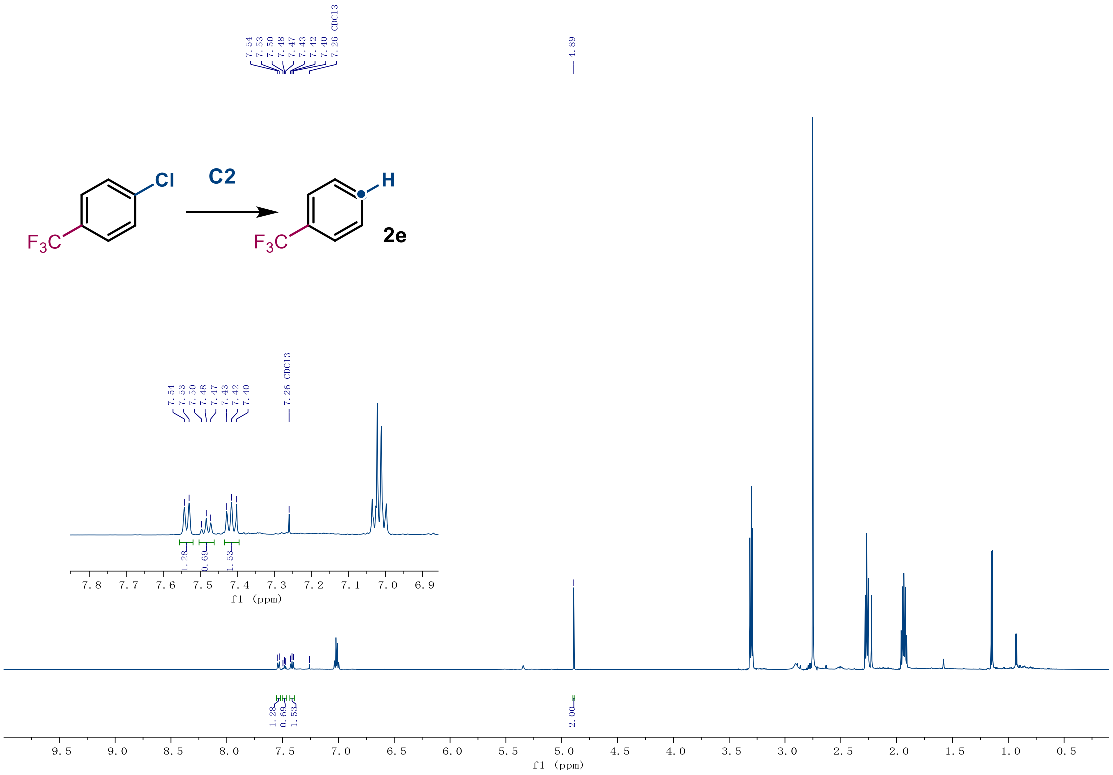

Crude reaction mixture - ^{1}H NMR (600 MHz, $CDCl_3$)

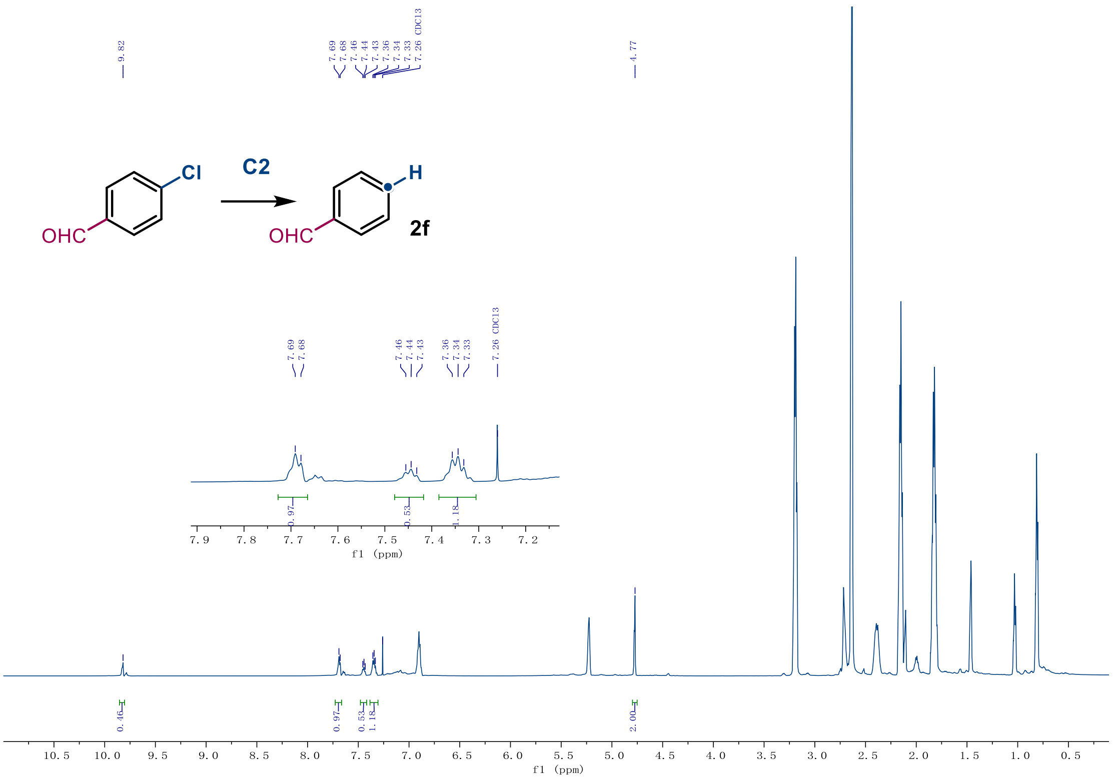

Crude reaction mixture - ^{1}H NMR (600 MHz, $CDCl_3$)

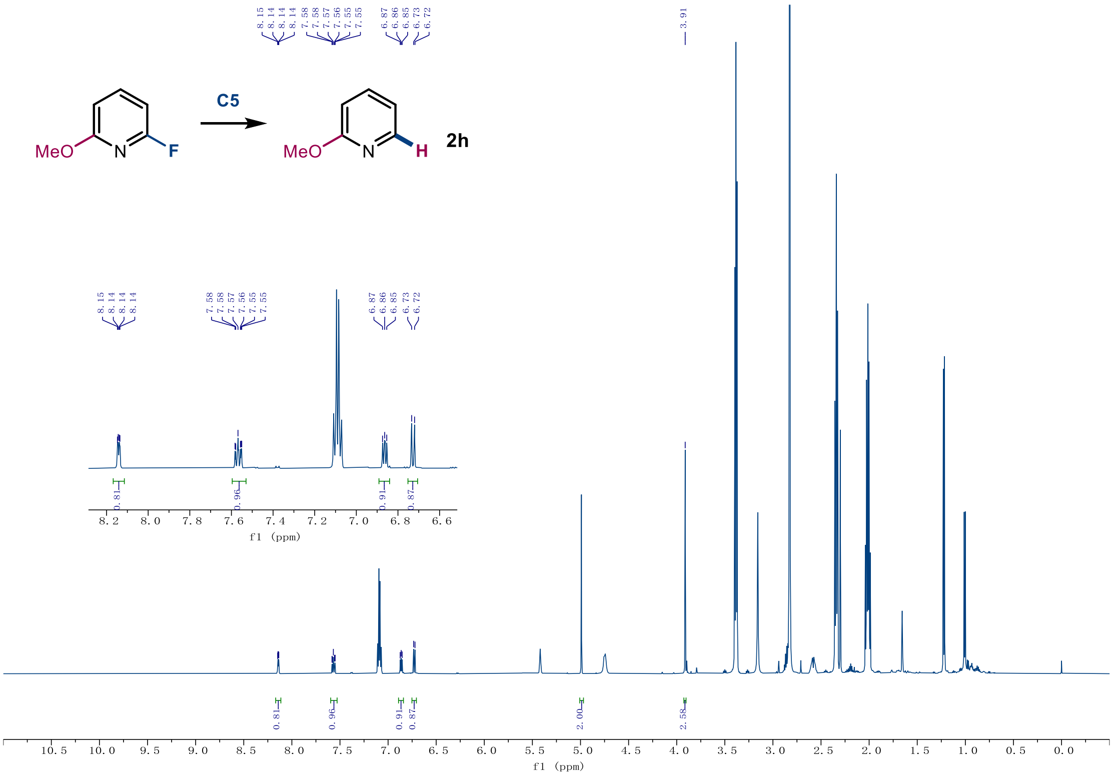

Isolated product - ^{1}H NMR (600 MHz, $CDCl_3$)

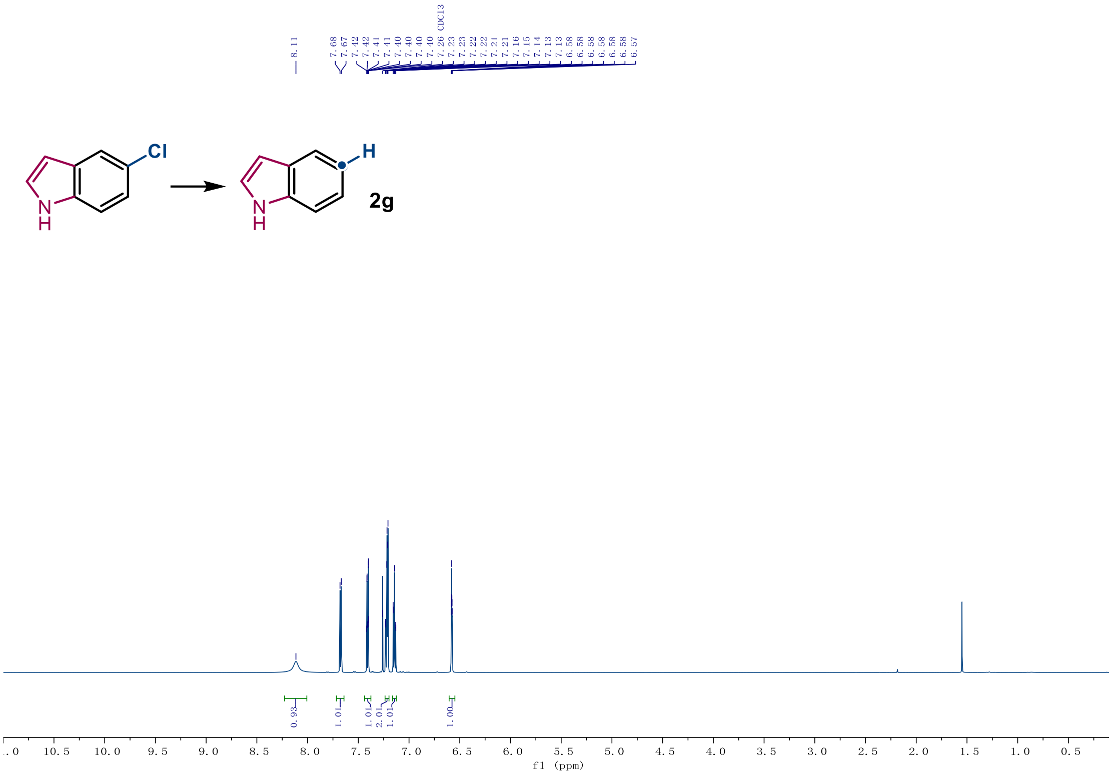

^{13}C NMR (151 MHz, $CDCl_3$)

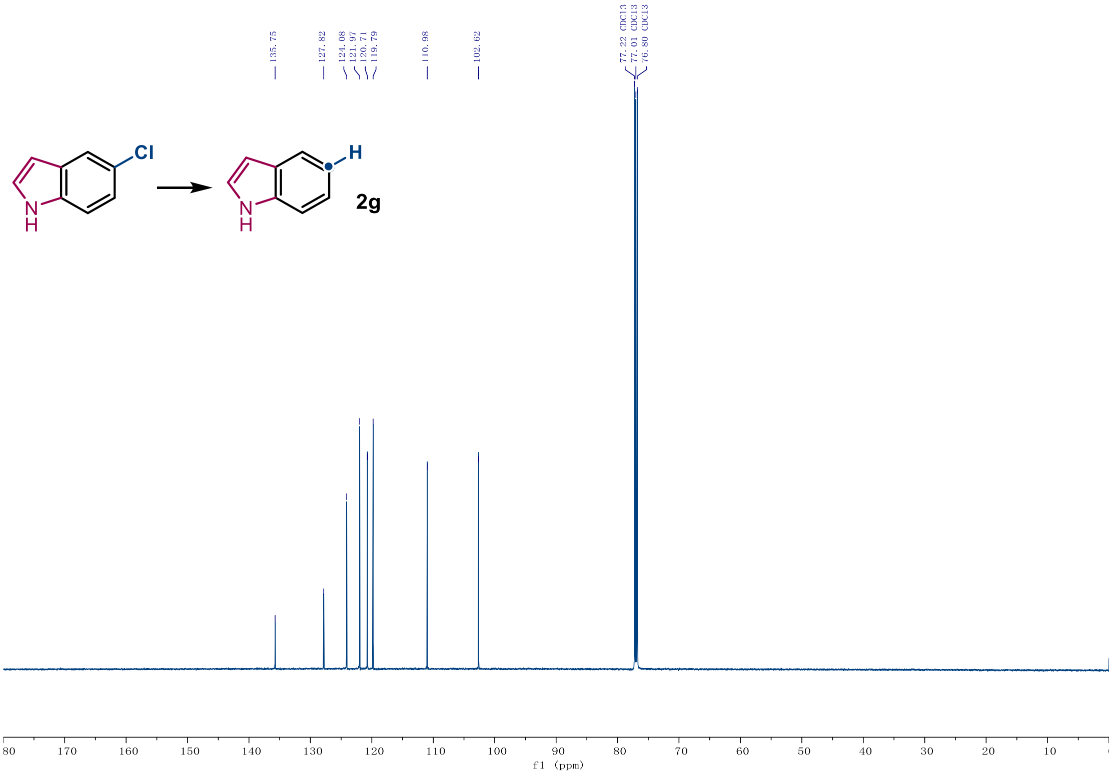

Isolated product - <u>^{1}H NMR</u> (600 MHz, $CDCl_3$)

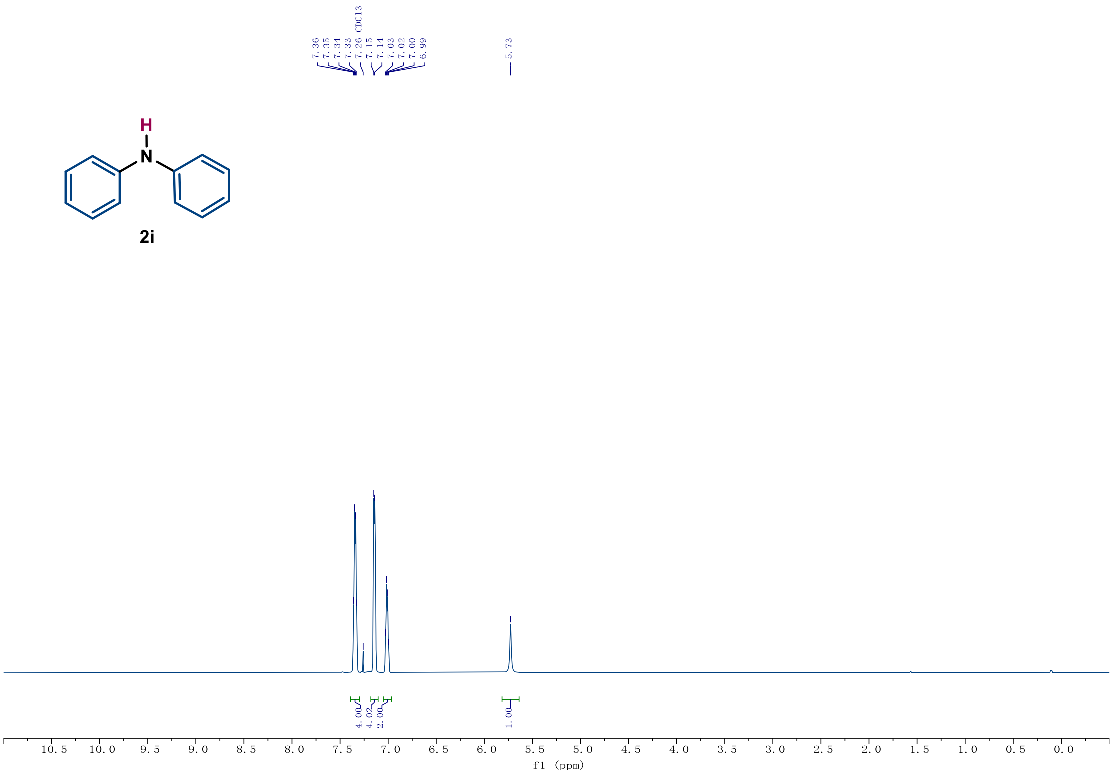

<u>^{13}C NMR</u> (151 MHz, $CDCl_3$)

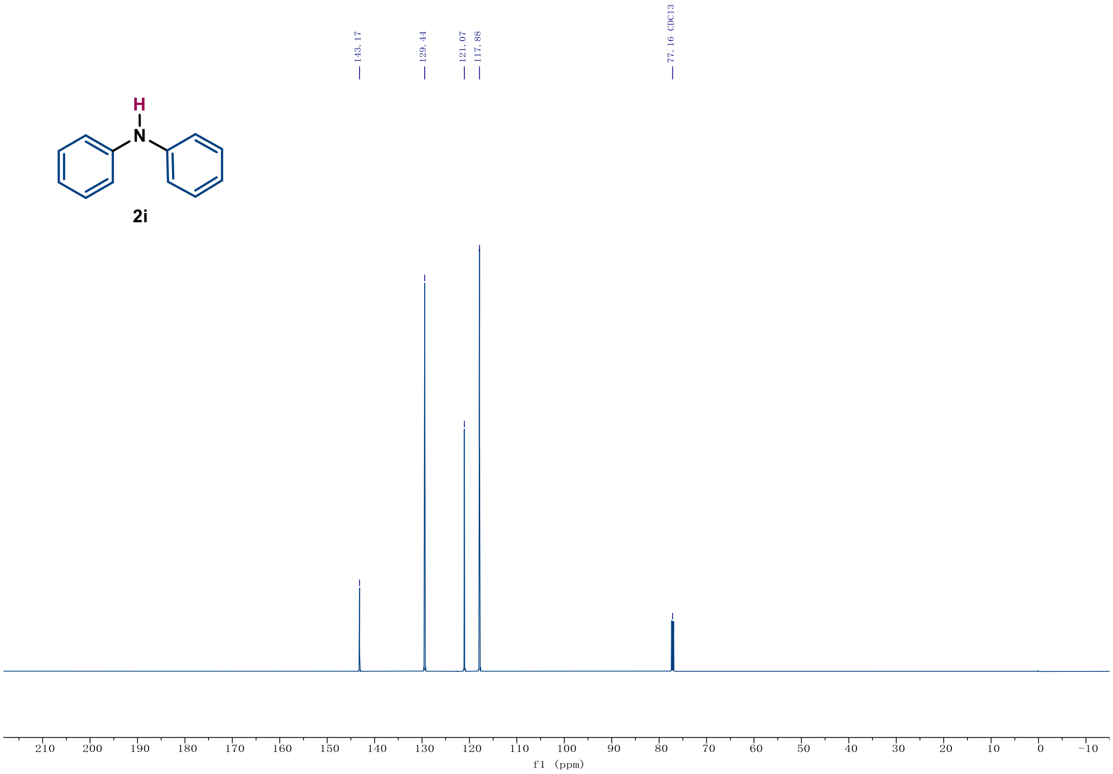

Isolated product - ^{1}H NMR (600 MHz, CDCl$_3$)

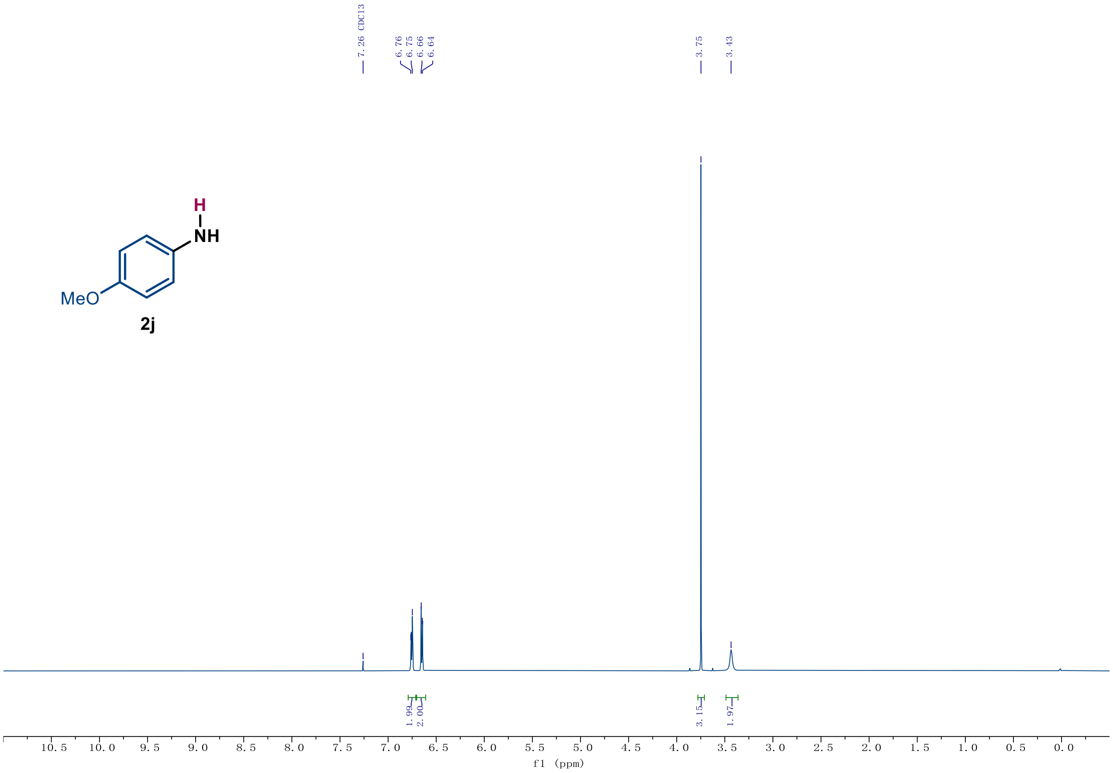

^{13}C NMR (126 MHz, CDCl$_3$)

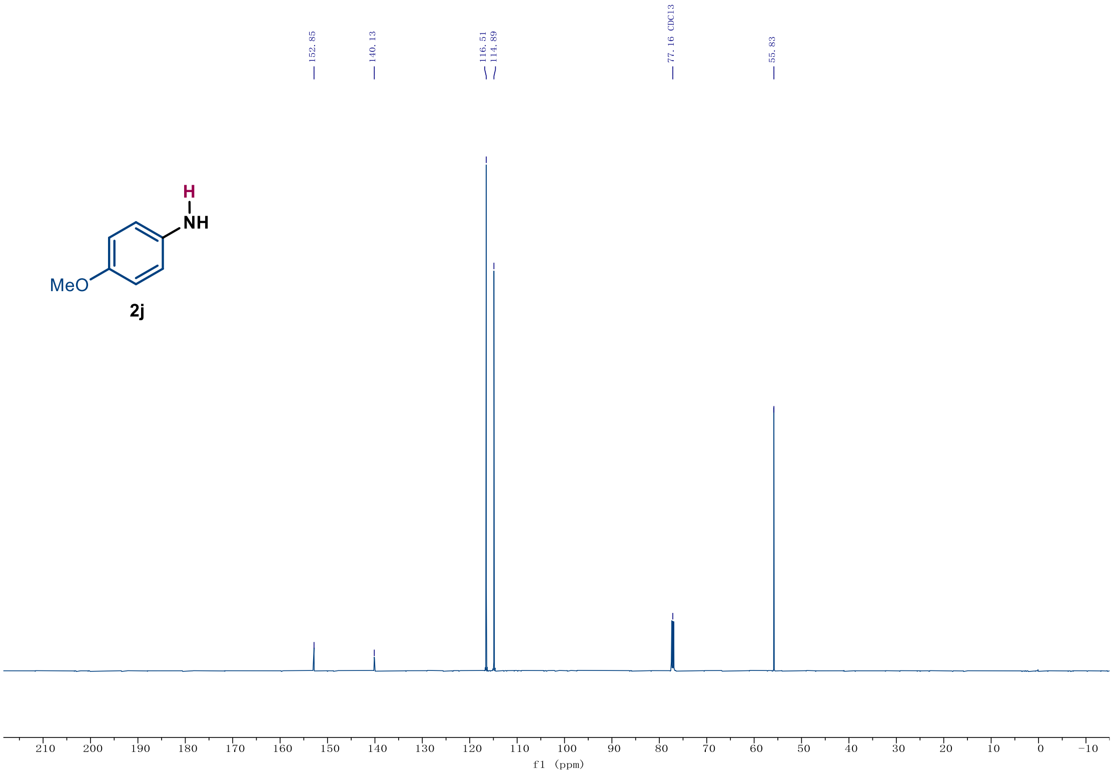

SET-based Processes – borylation, phosphorylation and thiolation *(Figure 5c in the main manuscript)*

Isolated product - ^{1}H NMR (600 MHz, $CDCl_3$)

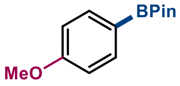

8a

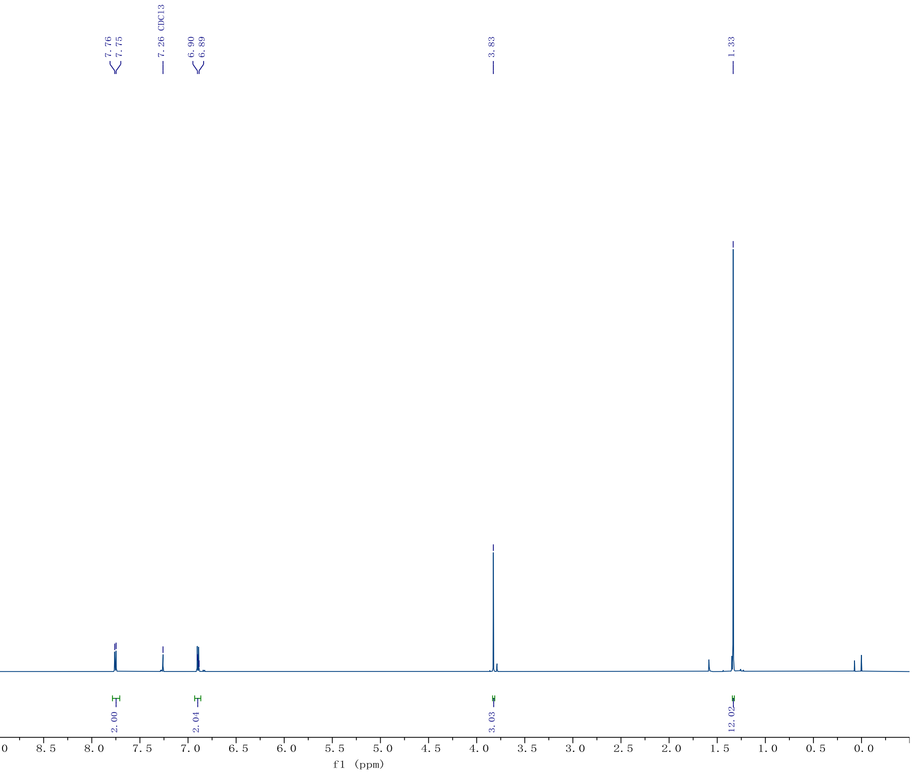

^{13}C NMR (151 MHz, $CDCl_3$)

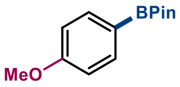

8a

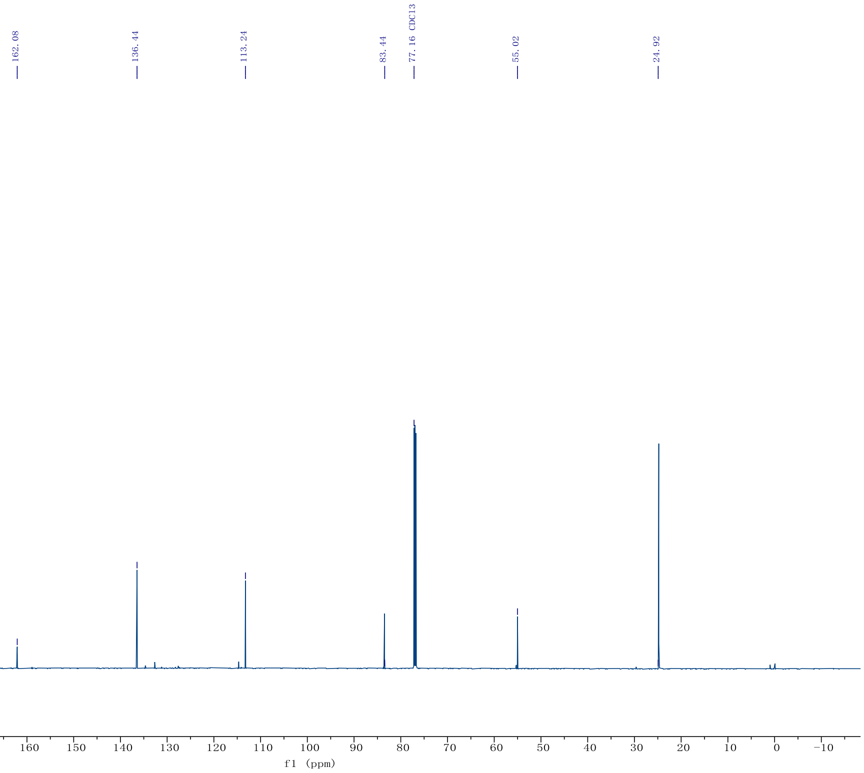

Isolated product - ^{1}H NMR (600 MHz, $CDCl_3$)

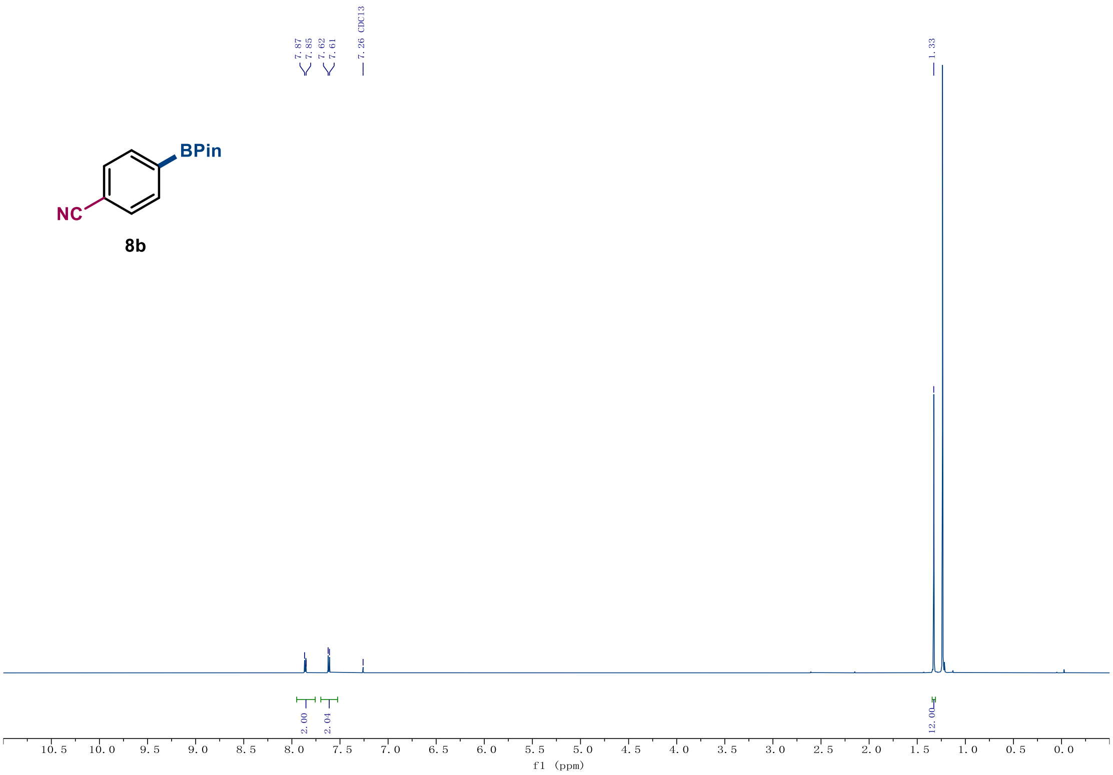

^{13}C NMR (151 MHz, $CDCl_3$)

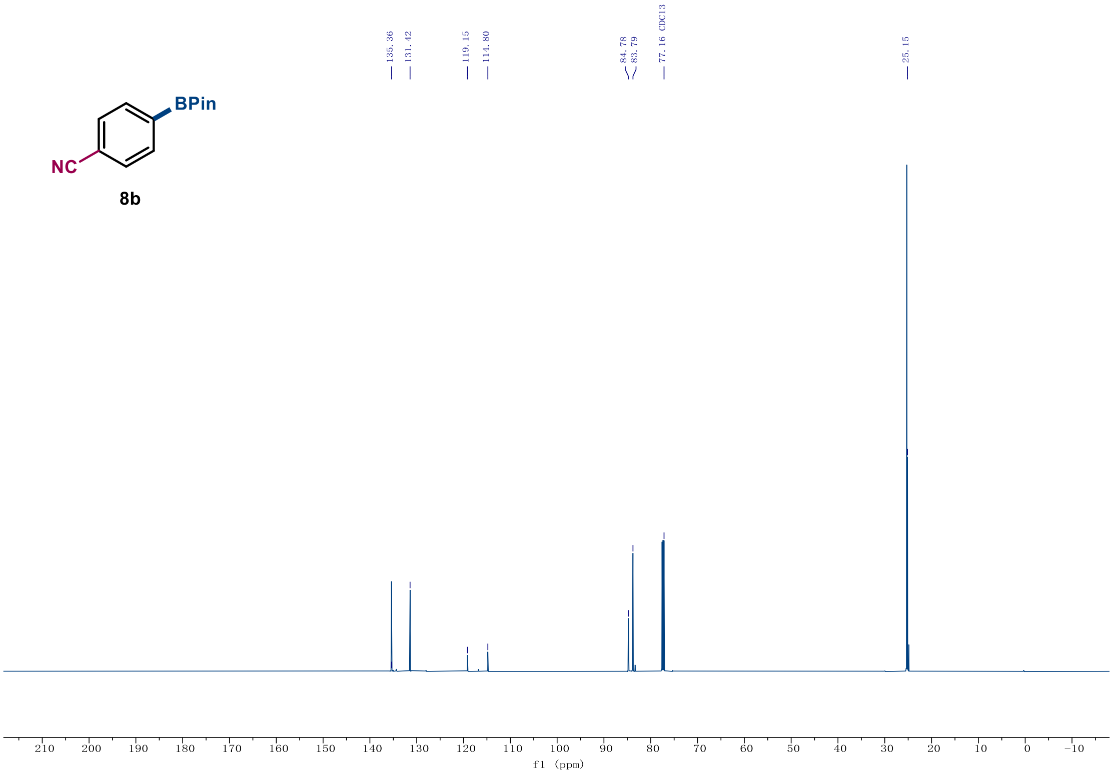

Isolated product - ^{1}H NMR (400 MHz, $CDCl_3$)

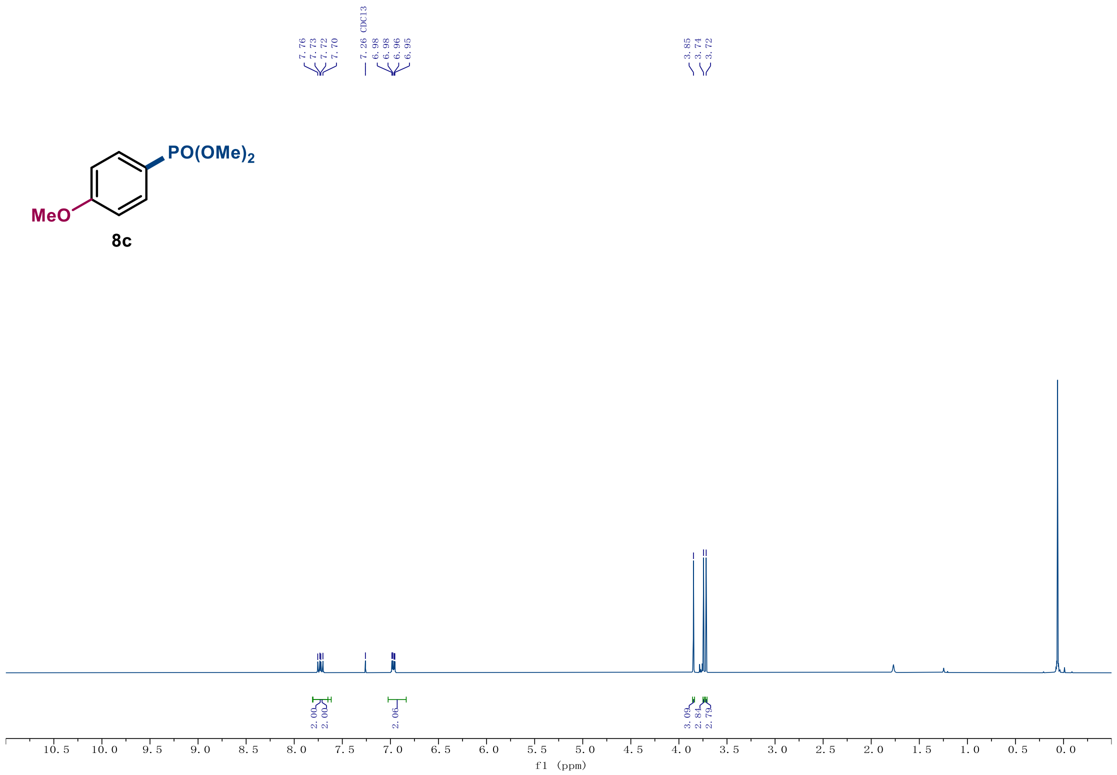

^{13}C NMR (151 MHz, $CDCl_3$)

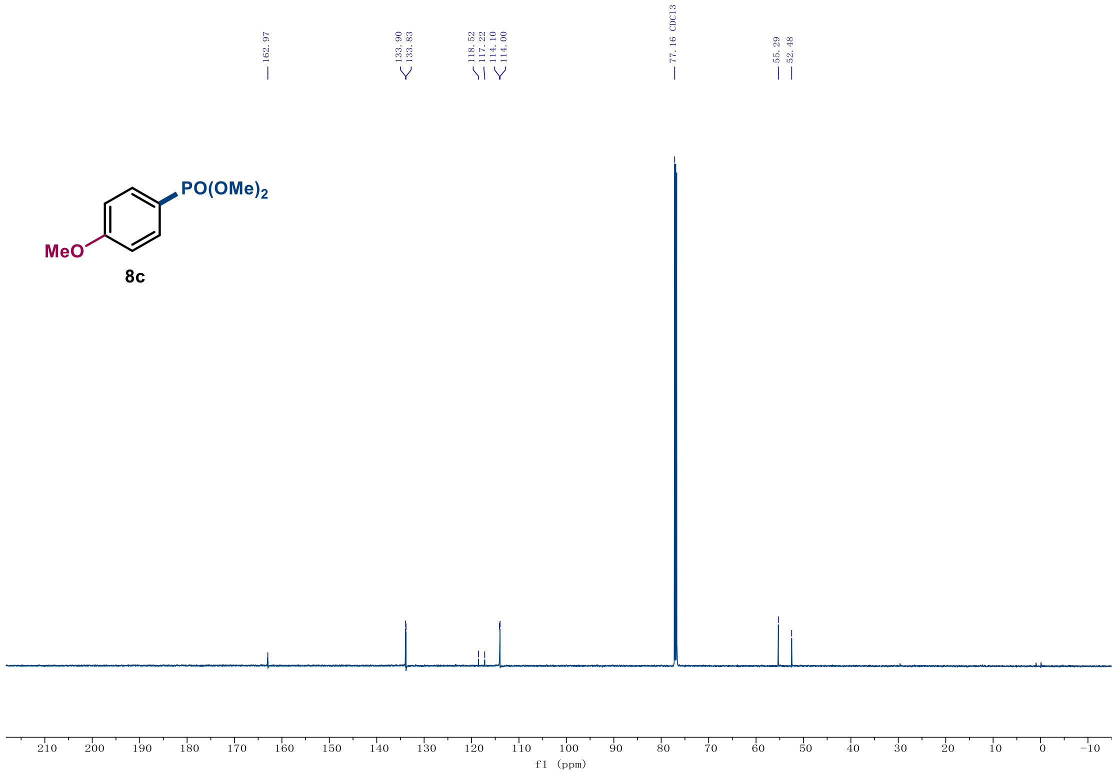

^{31}P NMR (243 MHz, $CDCl_3$)

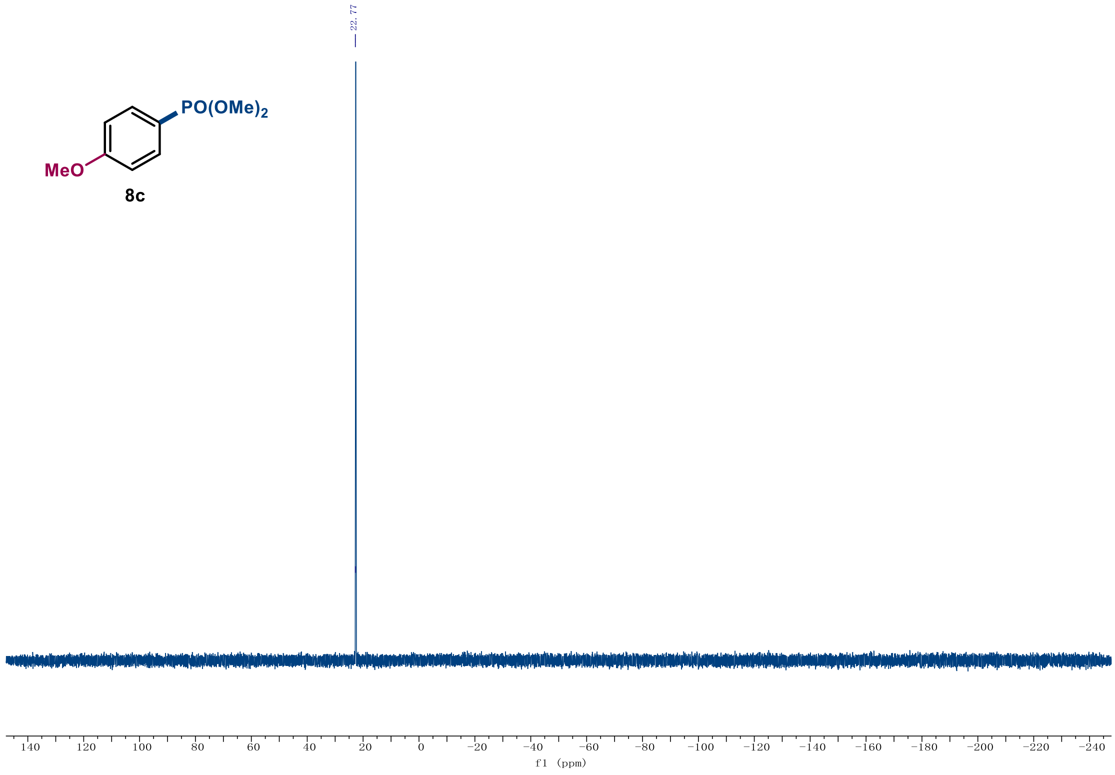

Isolated product - ¹H NMR (400 MHz, $CDCl_3$)

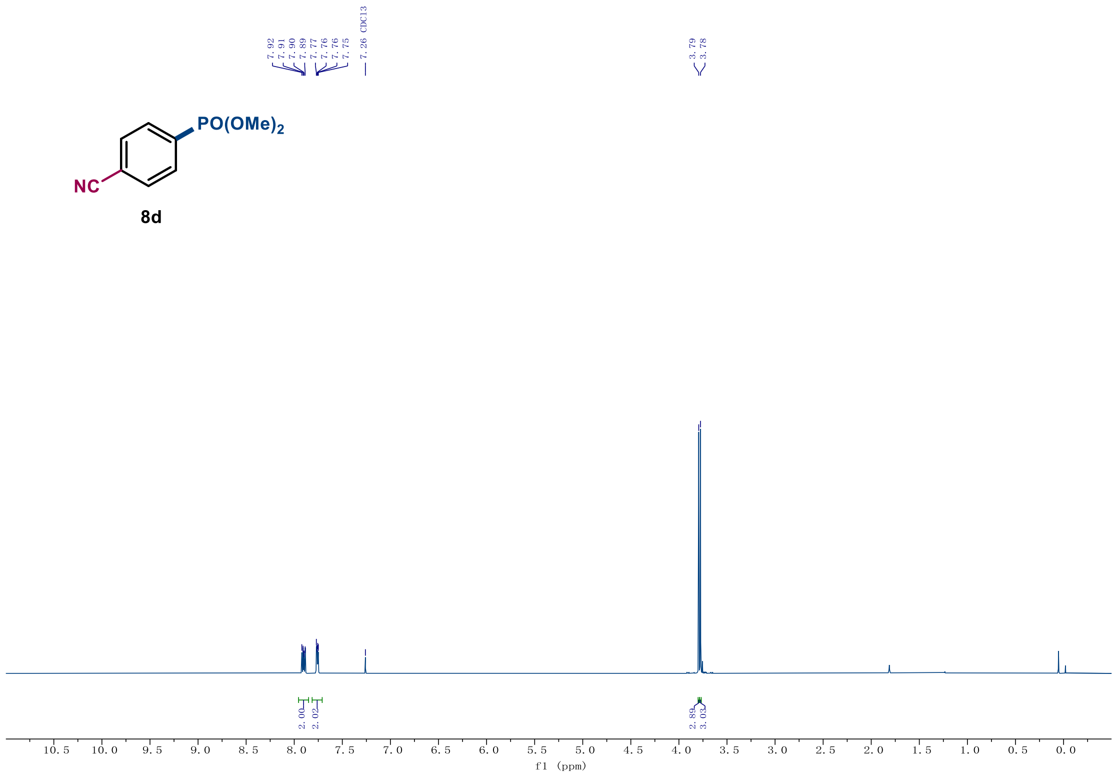

¹³C NMR (151 MHz, $CDCl_3$)

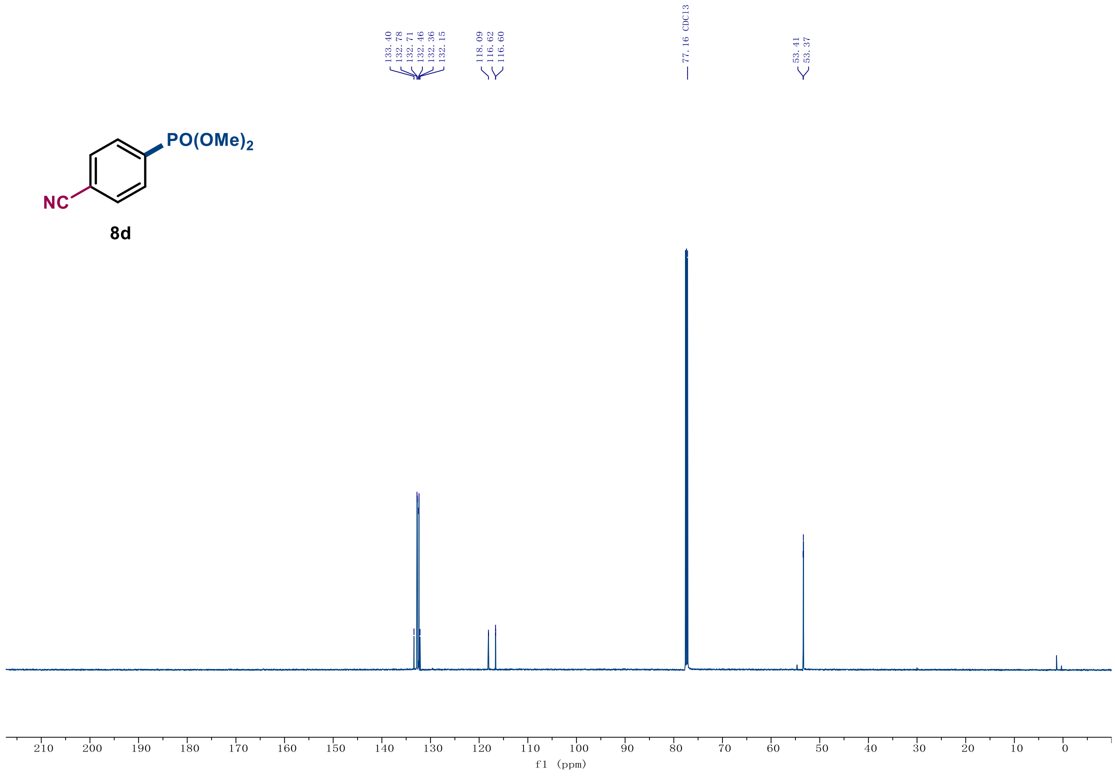

^{19}P NMR (243 MHz, $CDCl_3$)

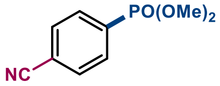

8d

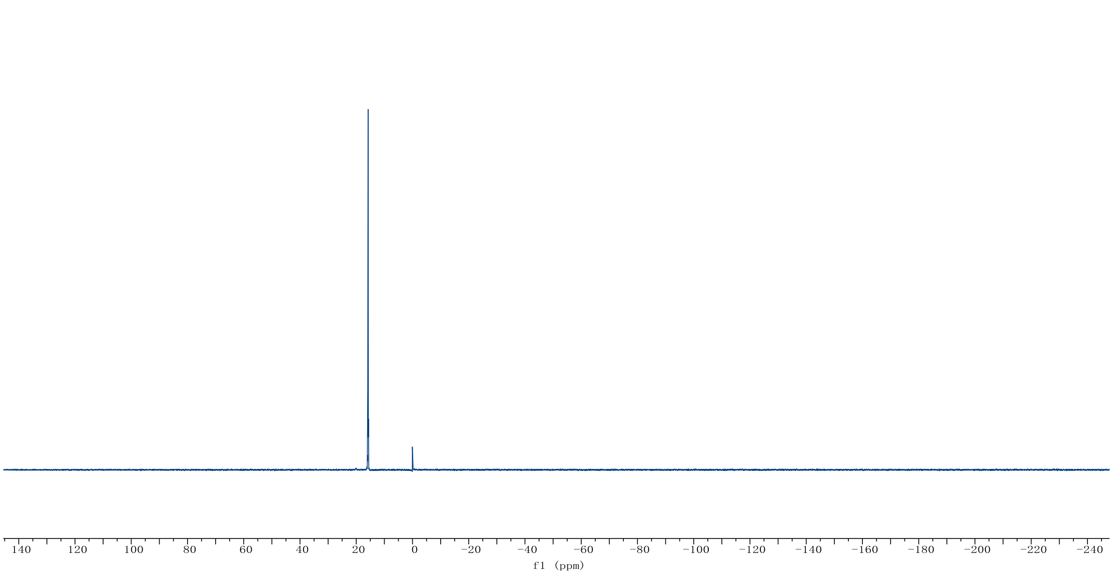

Isolated product - ^{1}H NMR (600 MHz, $CDCl_3$)

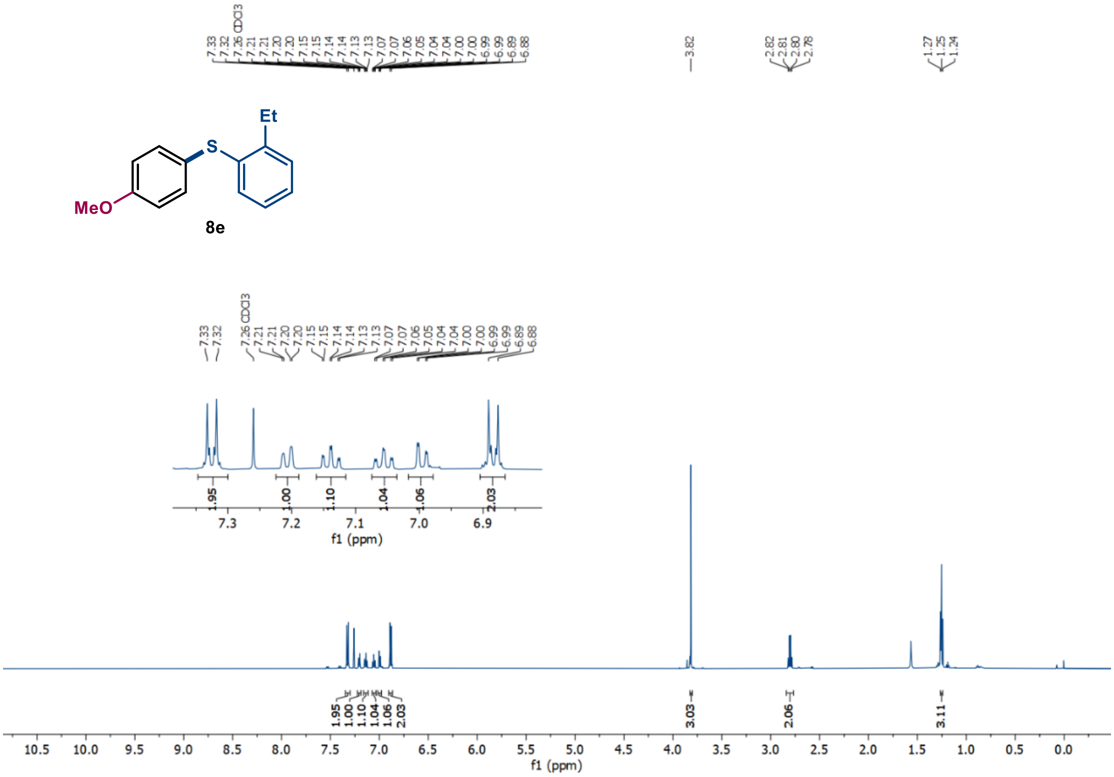

^{13}C NMR (151 MHz, $CDCl_3$)

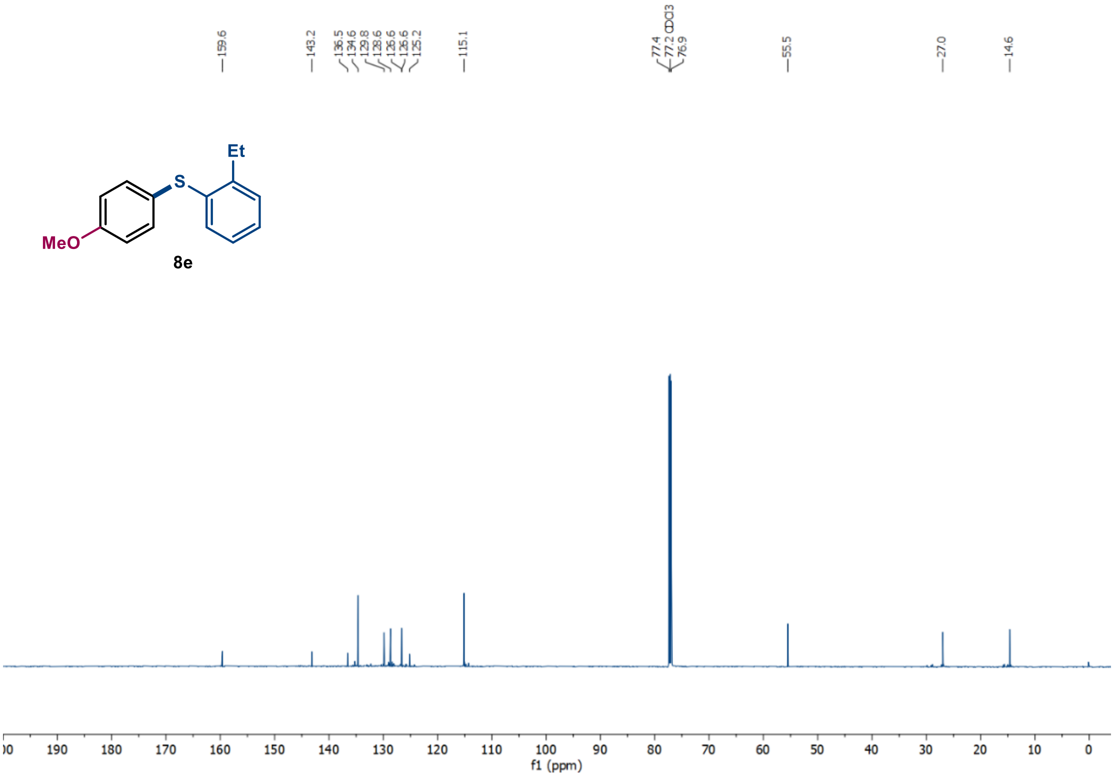

Isolated product - ^{1}H NMR (600 MHz, $CDCl_3$)

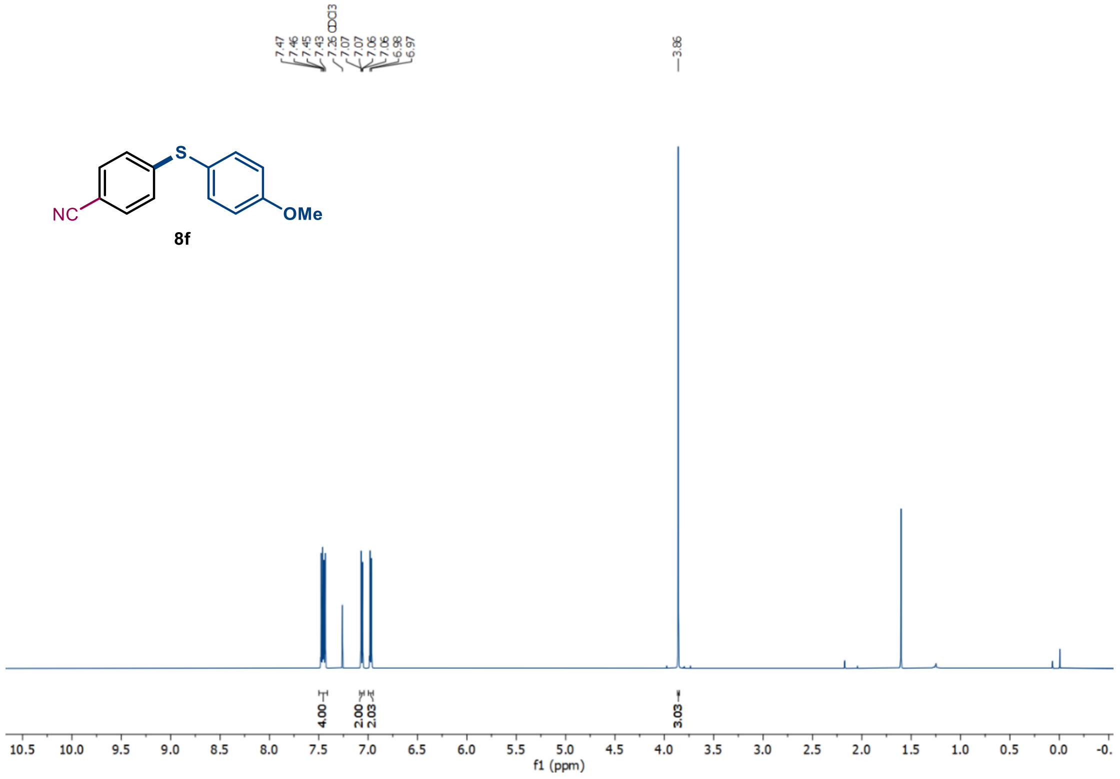

^{13}C NMR (151 MHz, $CDCl_3$)

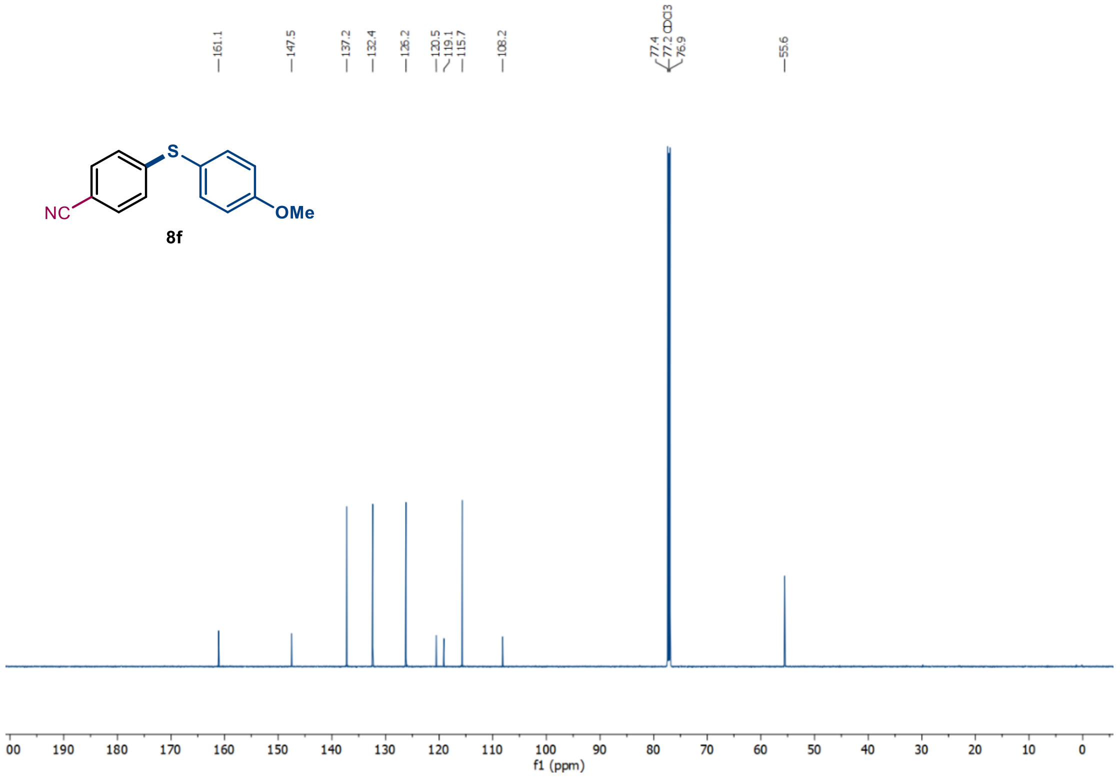

SET-based Processes – Activation of trifluorotoluenes *(Figures 5d&5e in the main manuscript)*

Isolated product - <u>^{1}H NMR</u> (600 MHz, $CDCl_3$)

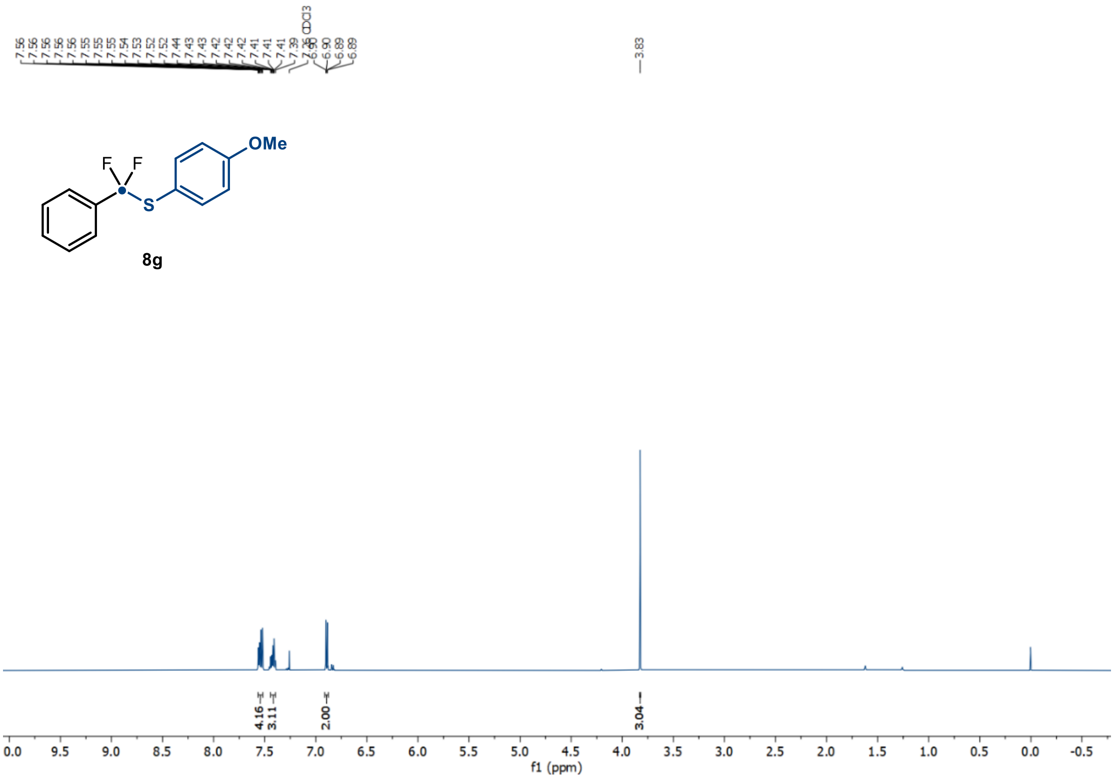

<u>^{19}F NMR</u> (376 MHz, $CDCl_3$)

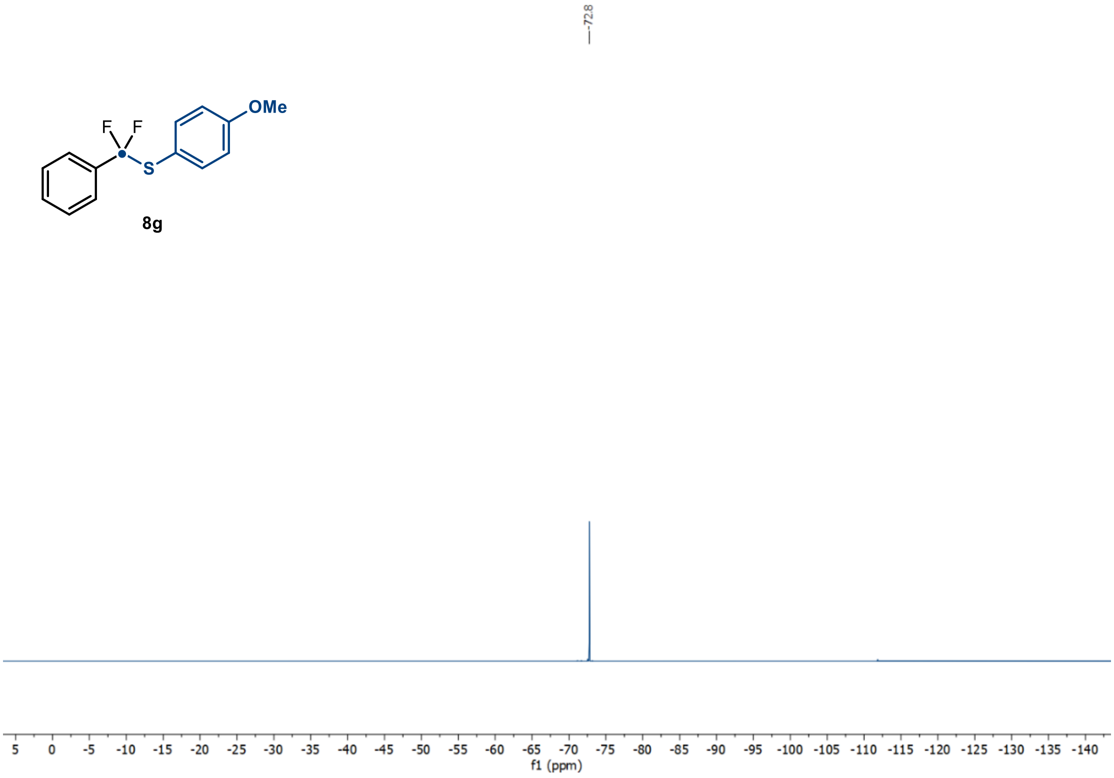

Isolated product - ^{1}H NMR (400 MHz, $CDCl_3$)

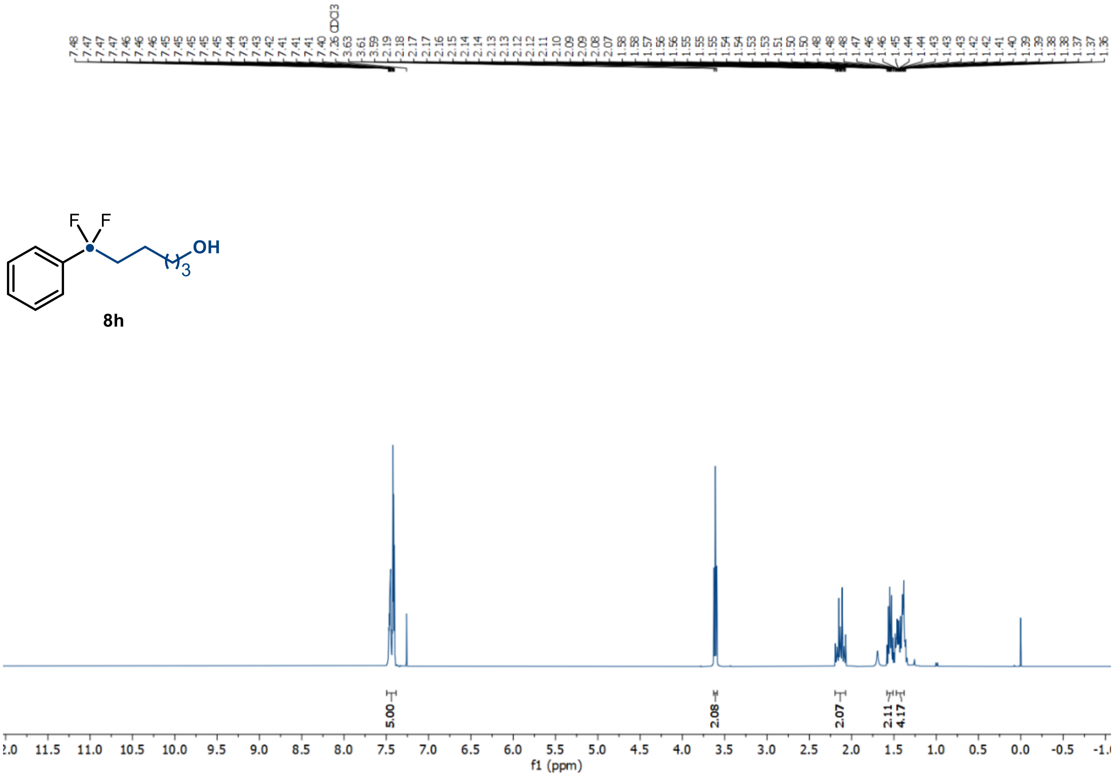

^{19}F NMR (376 MHz, $CDCl_3$)

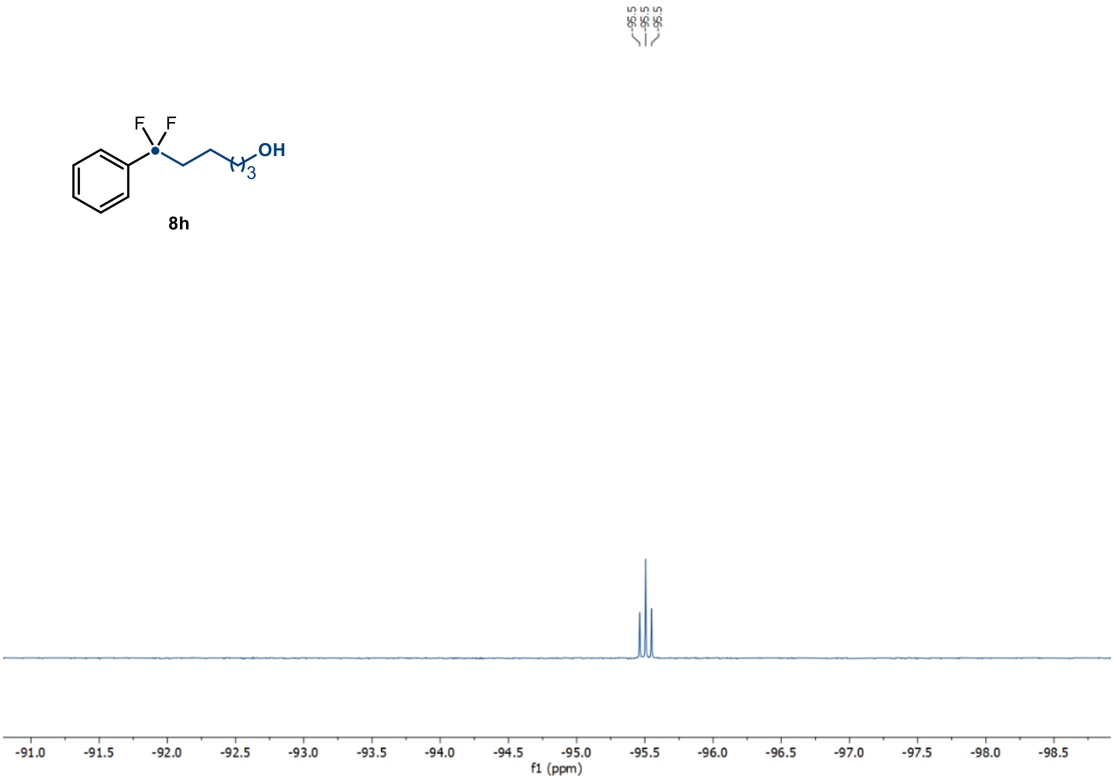

Isolated product - ^{1}H NMR (400 MHz, $CDCl_3$)

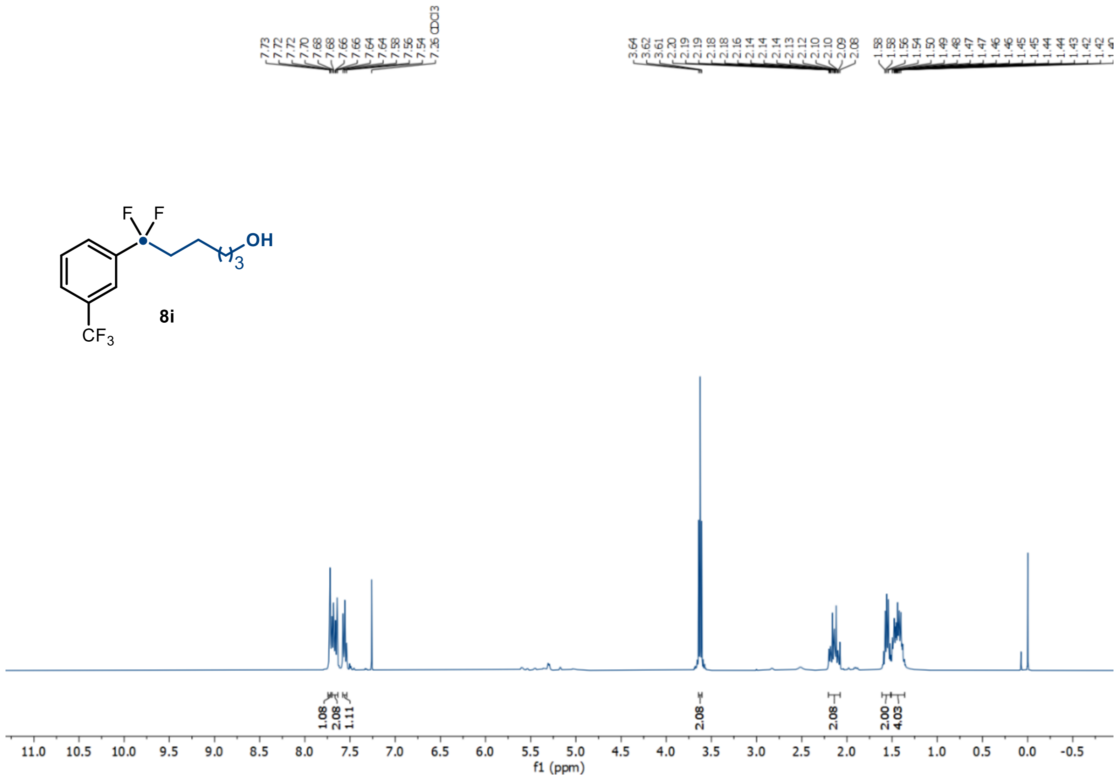

^{19}F NMR (376 MHz, $CDCl_3$)

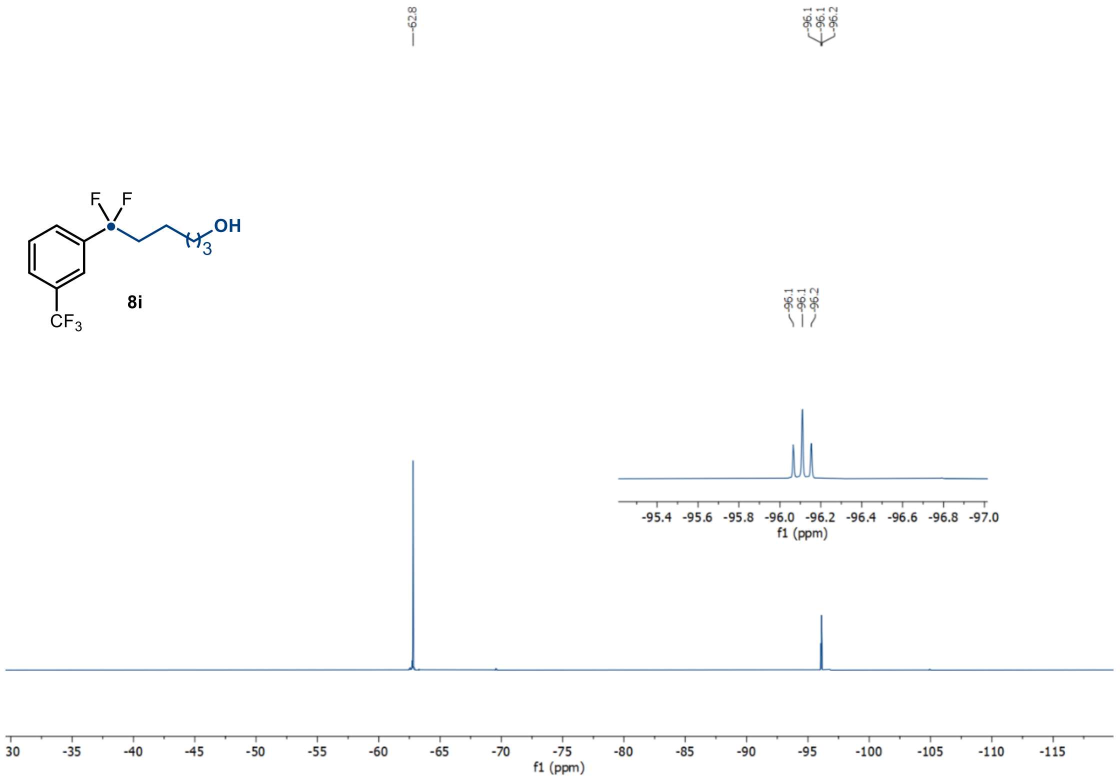

SET-based Processes – Birch-type Reduction (*Figure 5f in the main manuscript)*

Crude reaction mixture - ^{1}H NMR (600 MHz, $CDCl_3$)

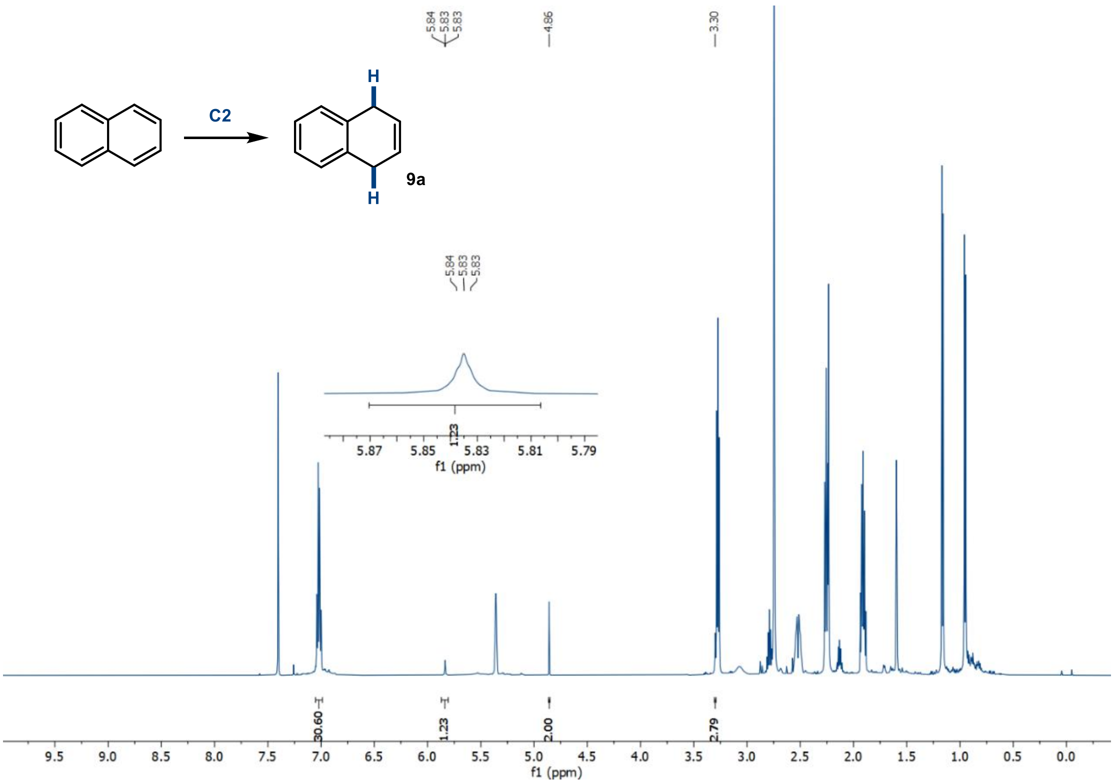

Crude reaction mixture - ^{1}H NMR (600 MHz, $CDCl_3$)

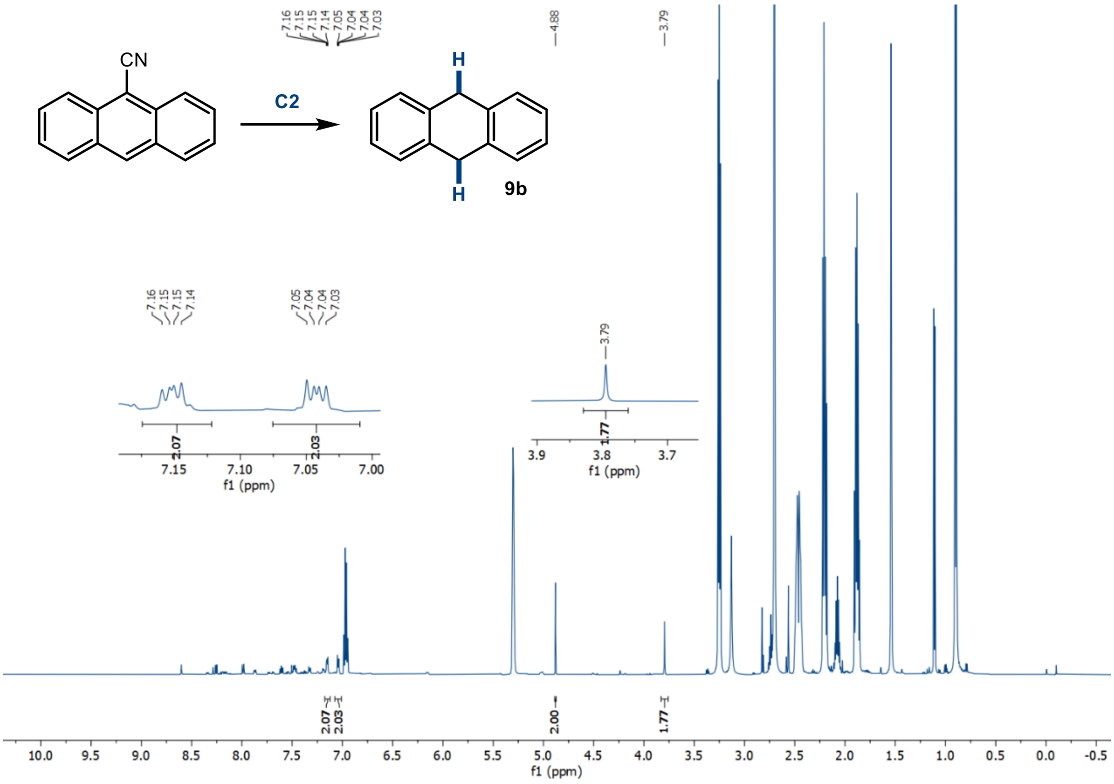

Isolated product - <u>^{1}H NMR</u> (400 MHz, $CDCl_3$)

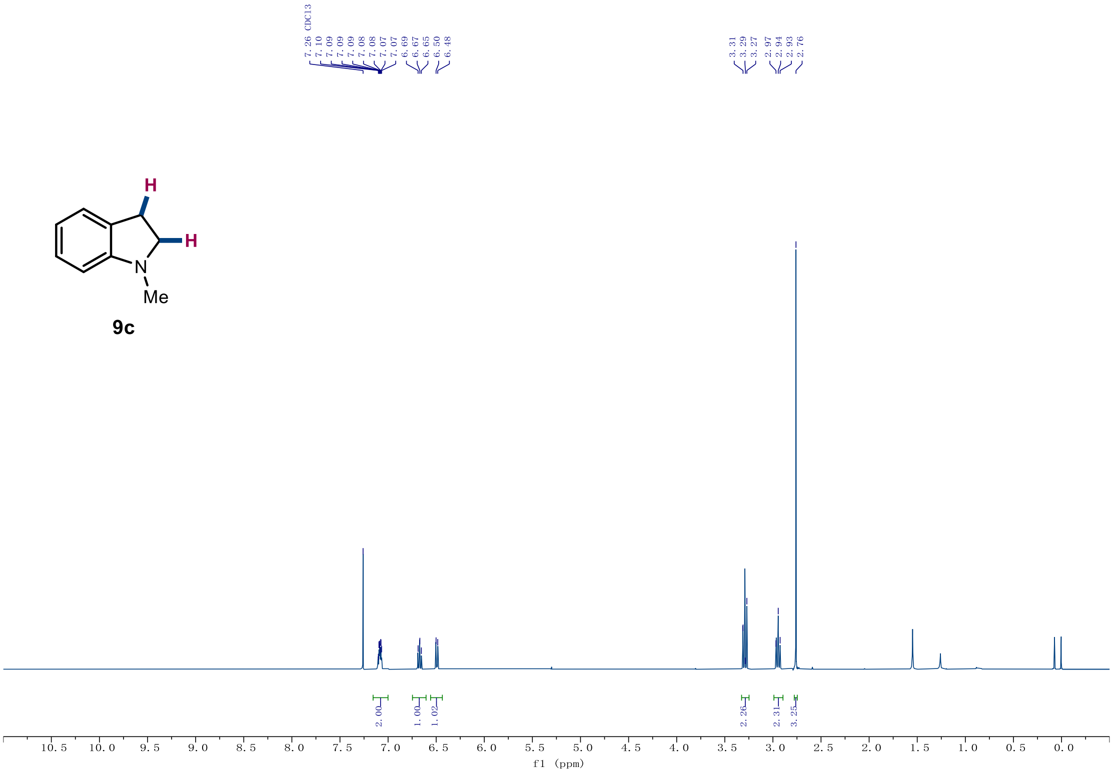

<u>^{13}C NMR</u> (151 MHz, $CDCl_3$)

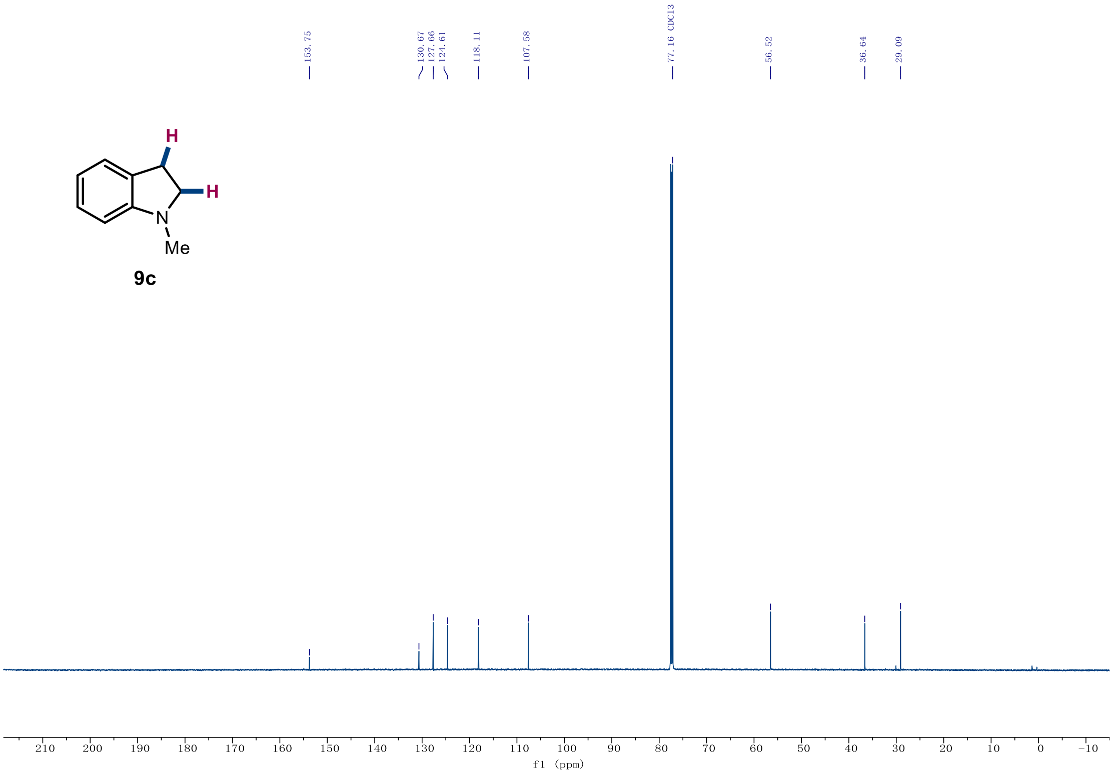

Crude reaction mixture - ^{1}H NMR (600 MHz, $CDCl_3$)

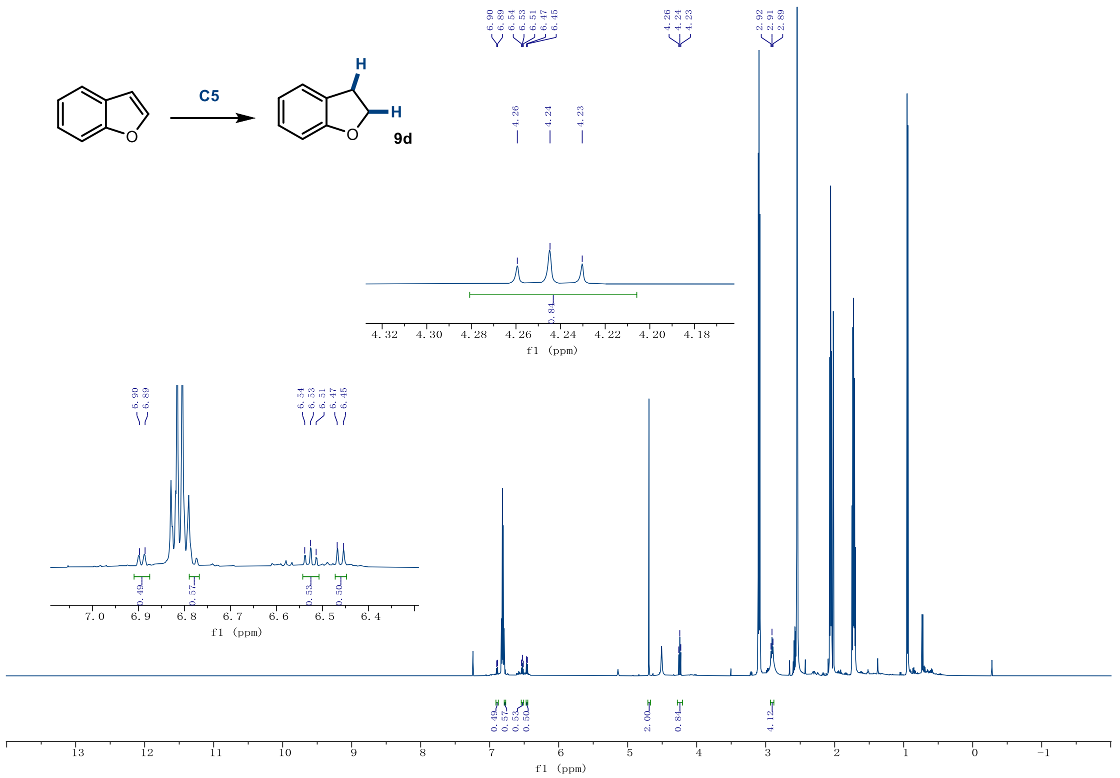

SET-based Processes – Radical Cyclisations *(Figures 5g&5h in the main manuscript)*

Isolated Product - ^{1}H NMR (600 MHz, $CDCl_3$)

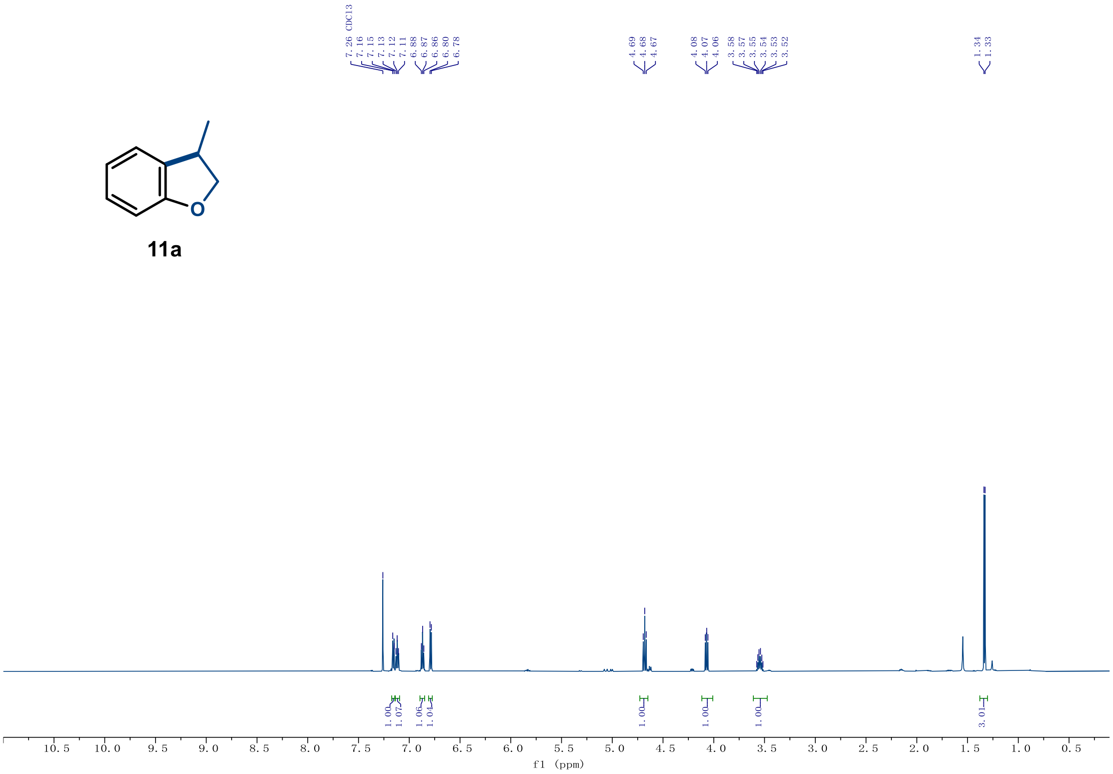

Isolated Product - ^{1}H NMR (600 MHz, $CDCl_3$)

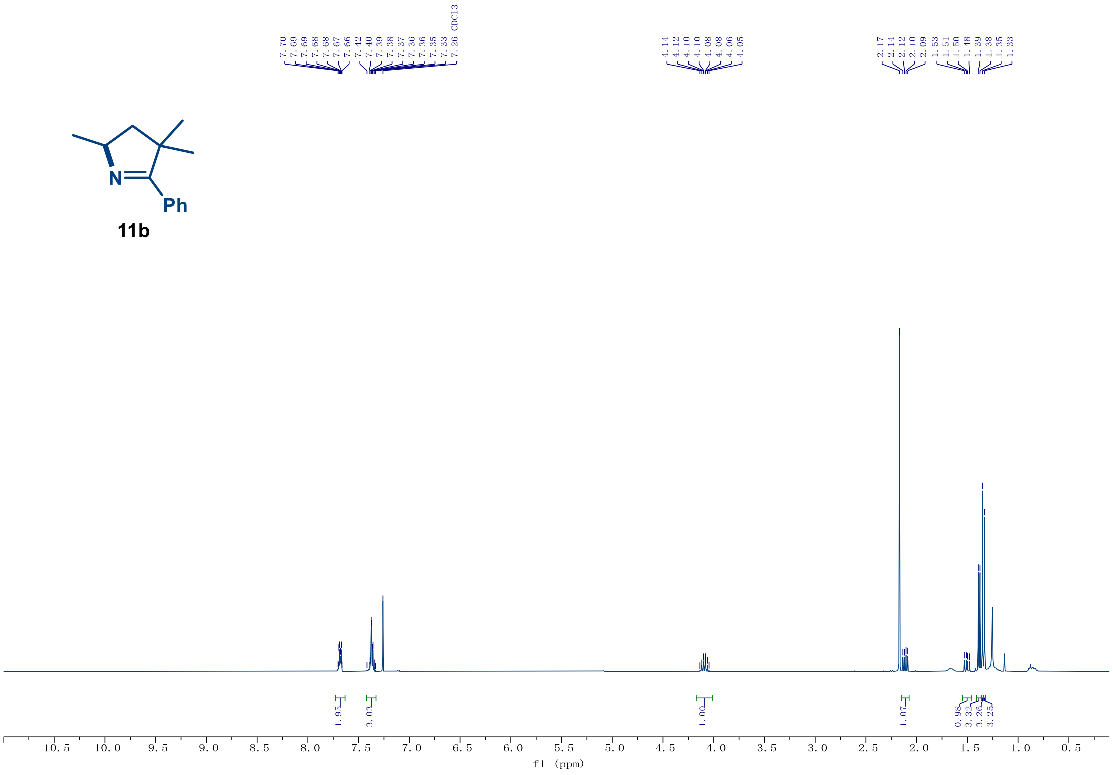

Isolated product - ¹H NMR (600 MHz, $CDCl_3$)

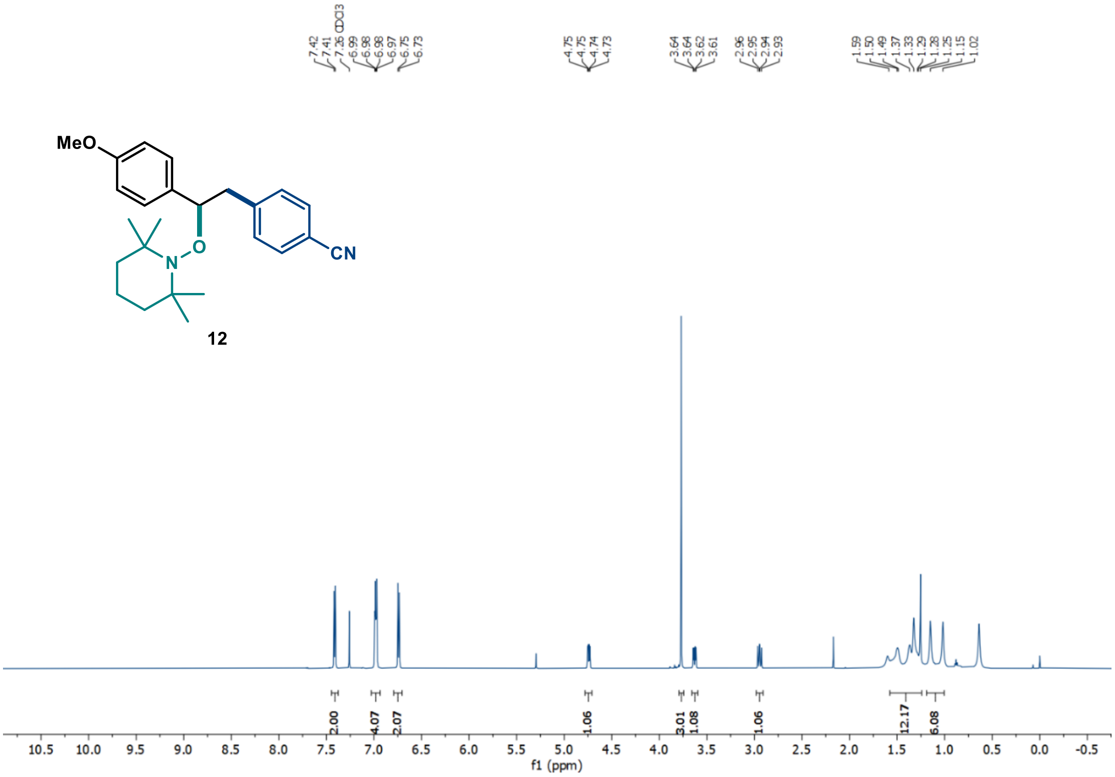

¹³C NMR (151 MHz, $CDCl_3$)

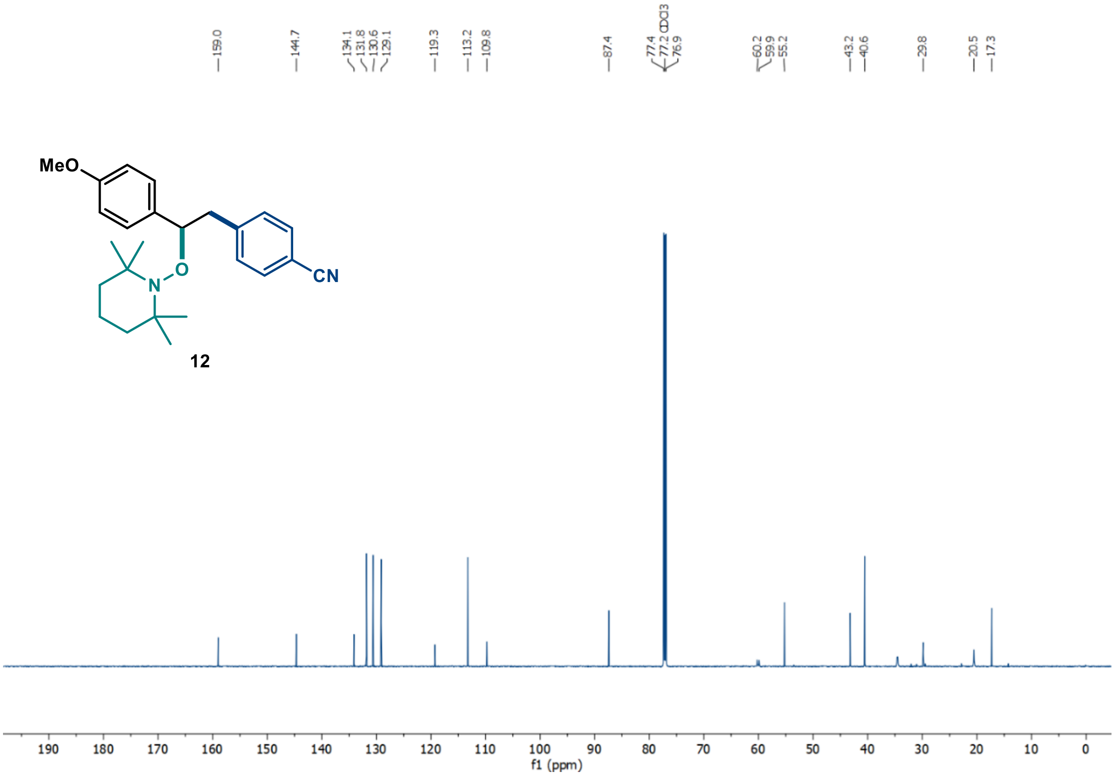

EnT-based Processes – *E/Z* Photoisomerization *(Figure 4d in the main manuscript)*

Substrates - ^{1}H NMR (400 MHz, $CDCl_3$)

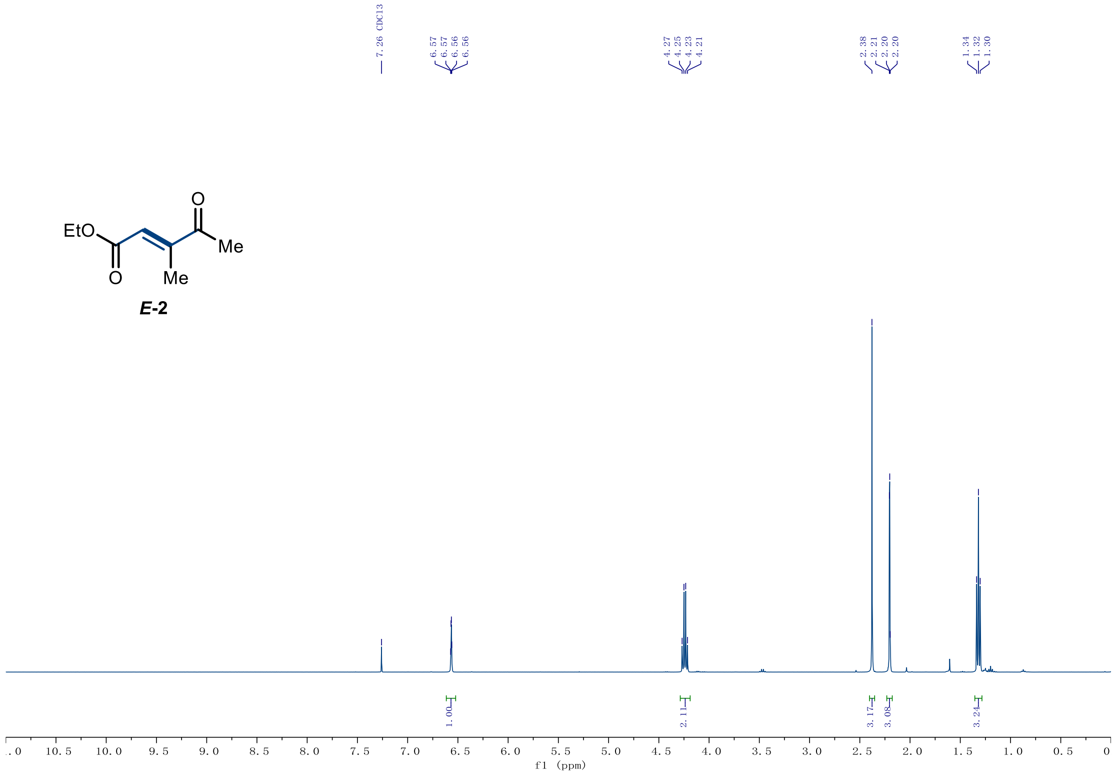

^{1}H NMR (400 MHz, $CDCl_3$)

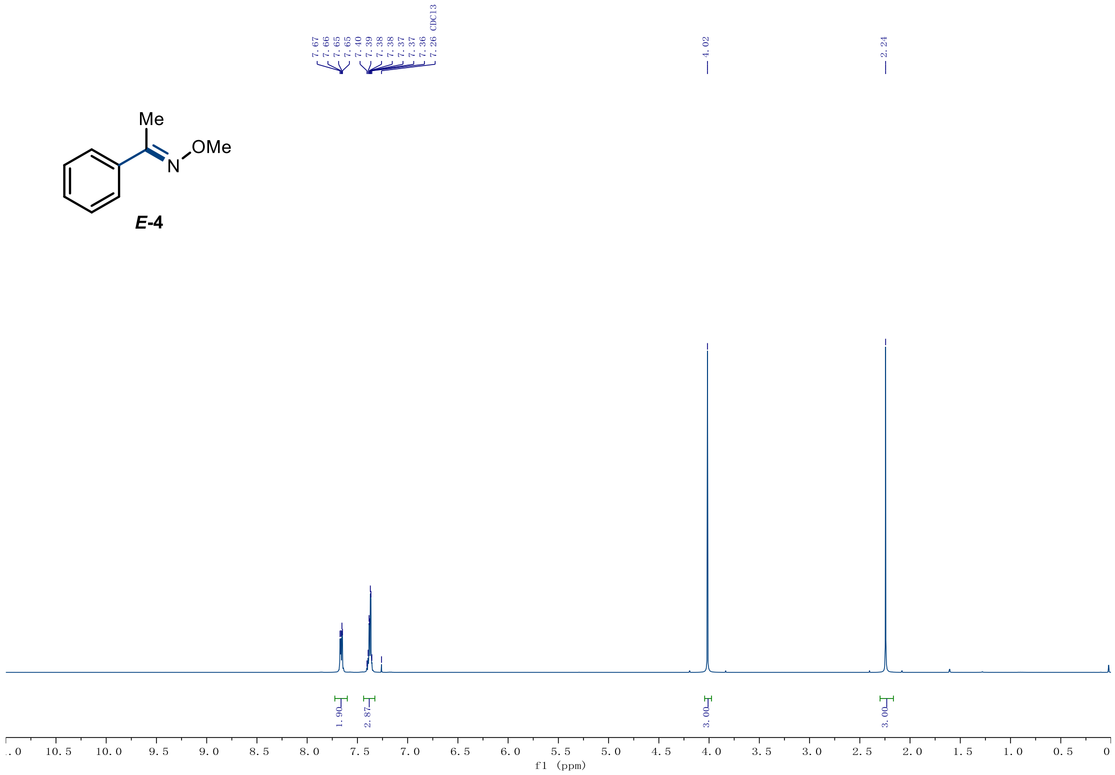

^{1}H NMR (400 MHz, $CDCl_3$)

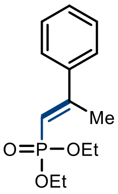

***E*-6**

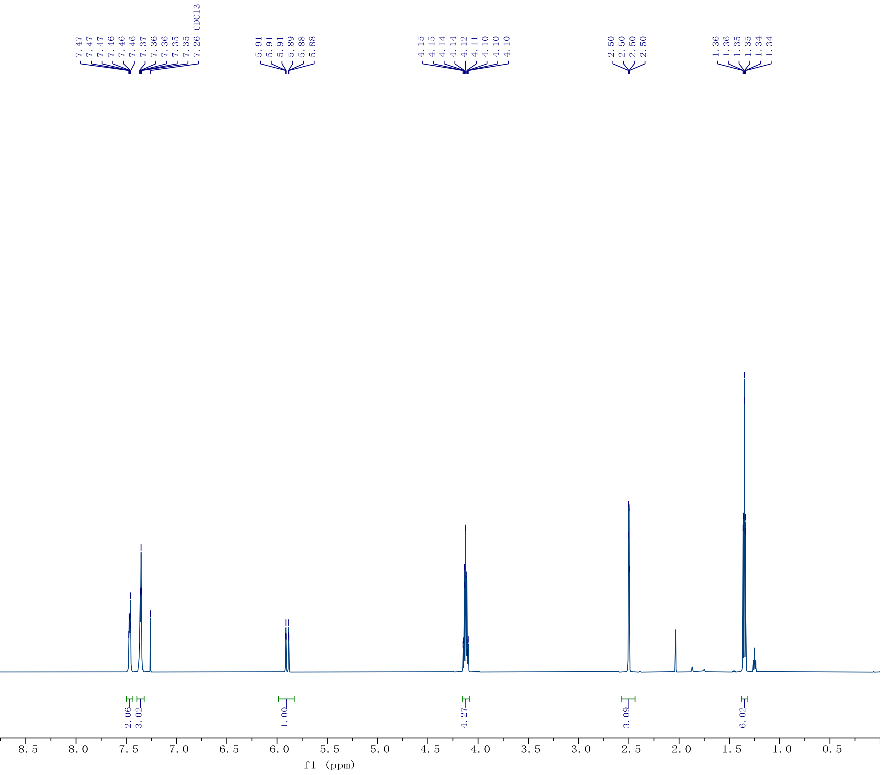

Products

Isolated product - ^{1}H NMR (600 MHz, $CDCl_3$)

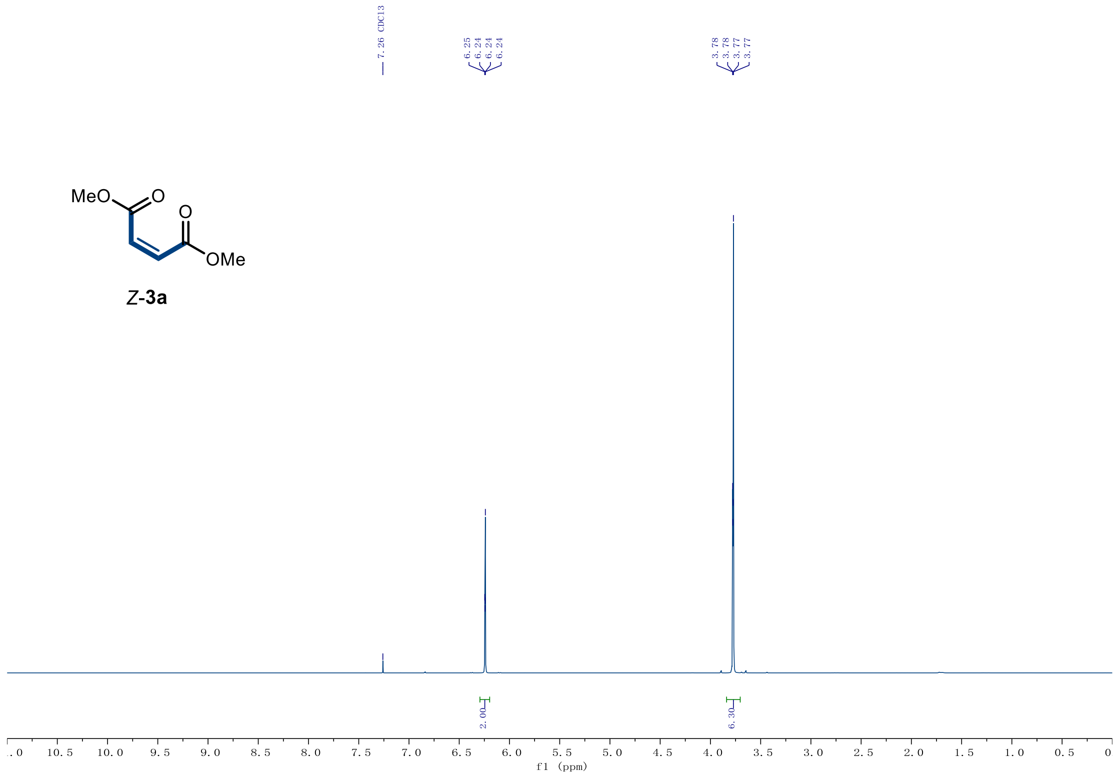

^{13}C NMR (151 MHz, $CDCl_3$)

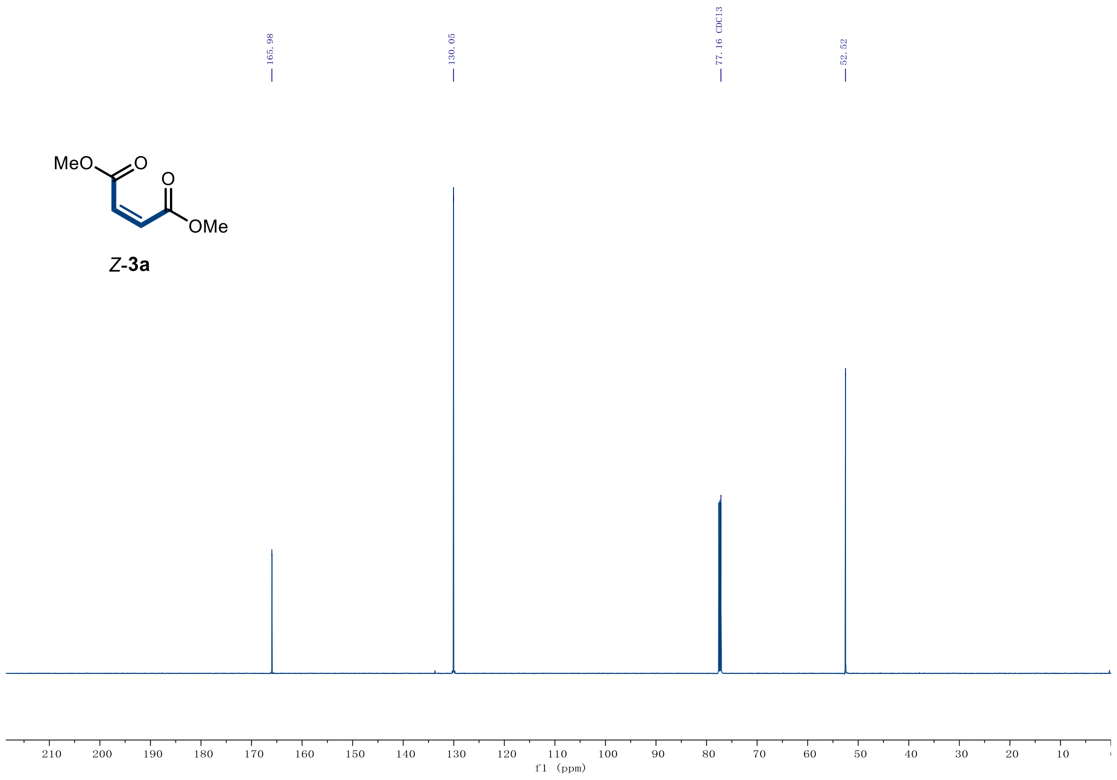

Isolated product - <u>^{1}H NMR</u> (600 MHz, $CDCl_3$)

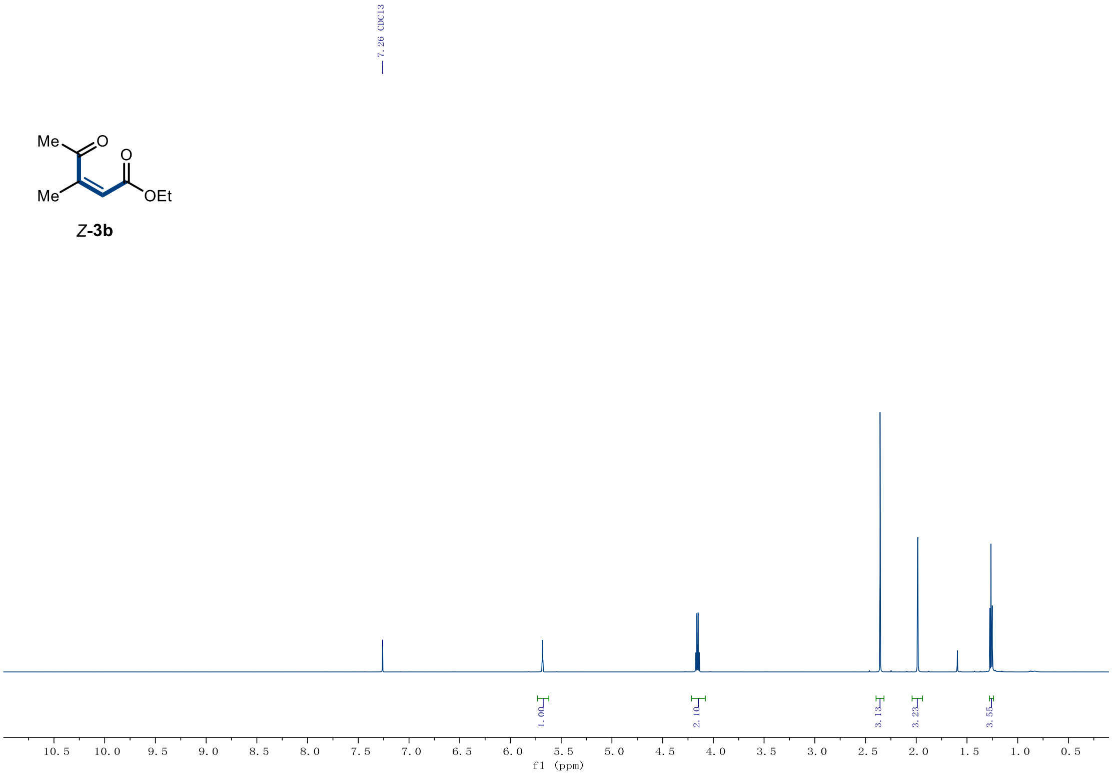

<u>^{13}C NMR</u> (151 MHz, $CDCl_3$)

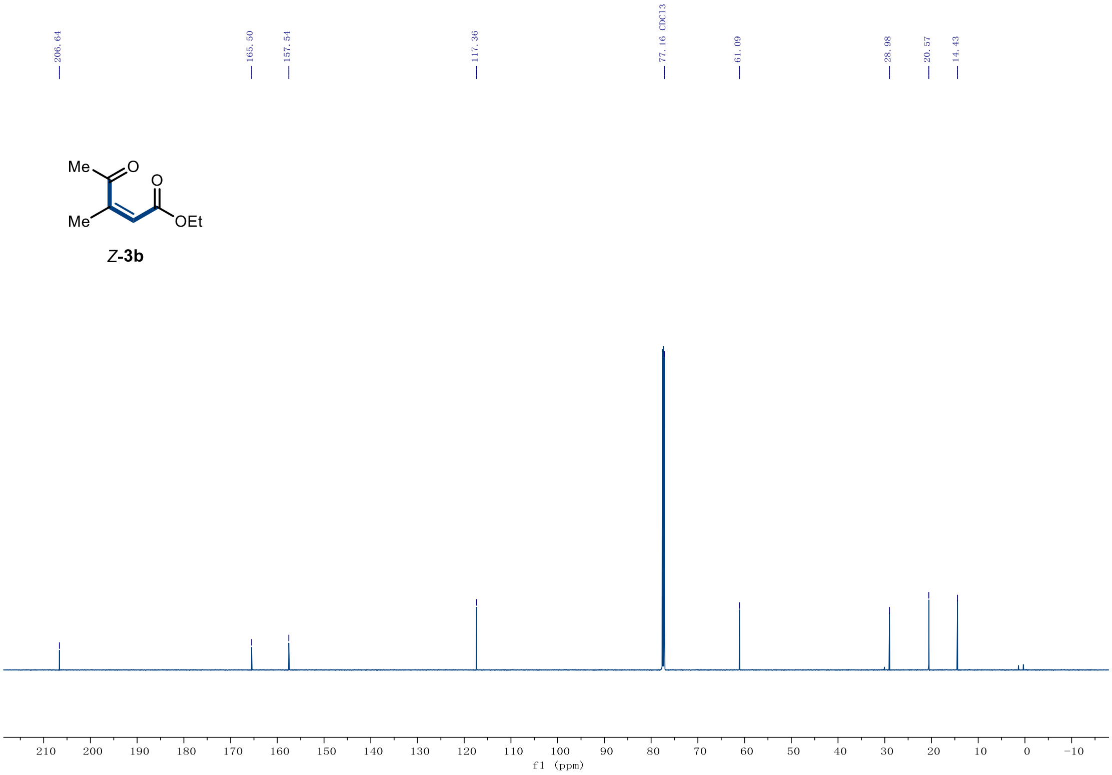

Crude reaction mixture - ¹H NMR (600 MHz, CD_3CN)

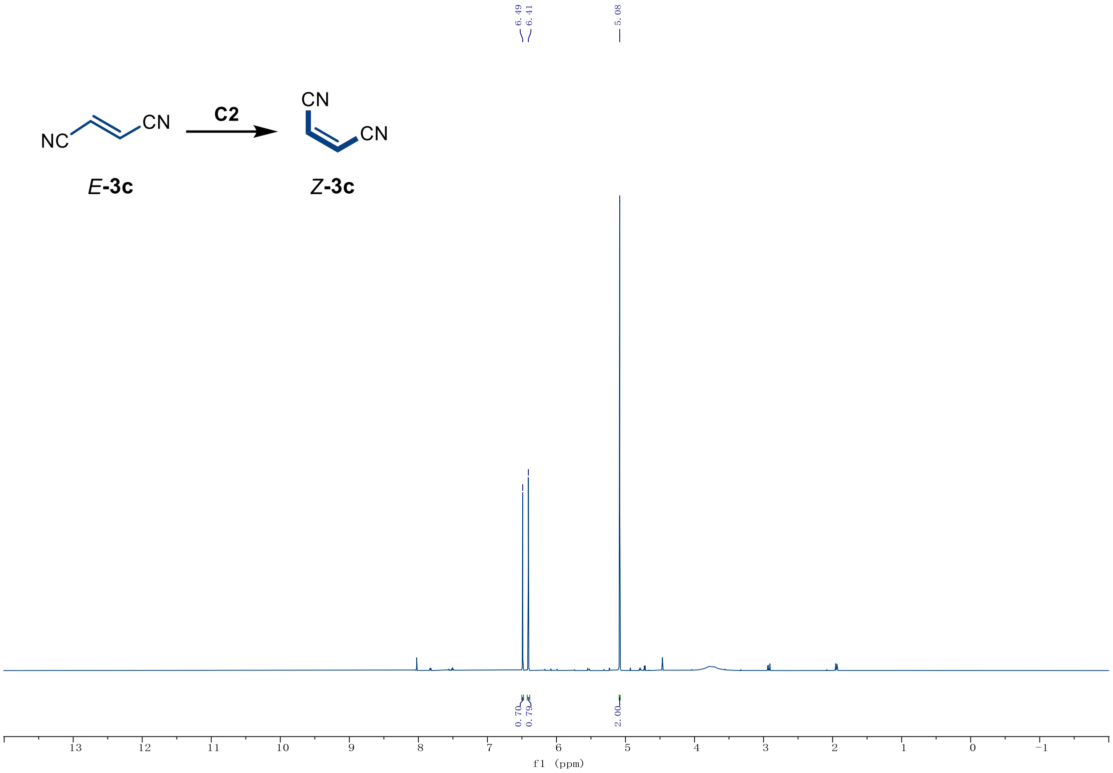

Isolated product - ¹H NMR (600 MHz, $CDCl_3$)

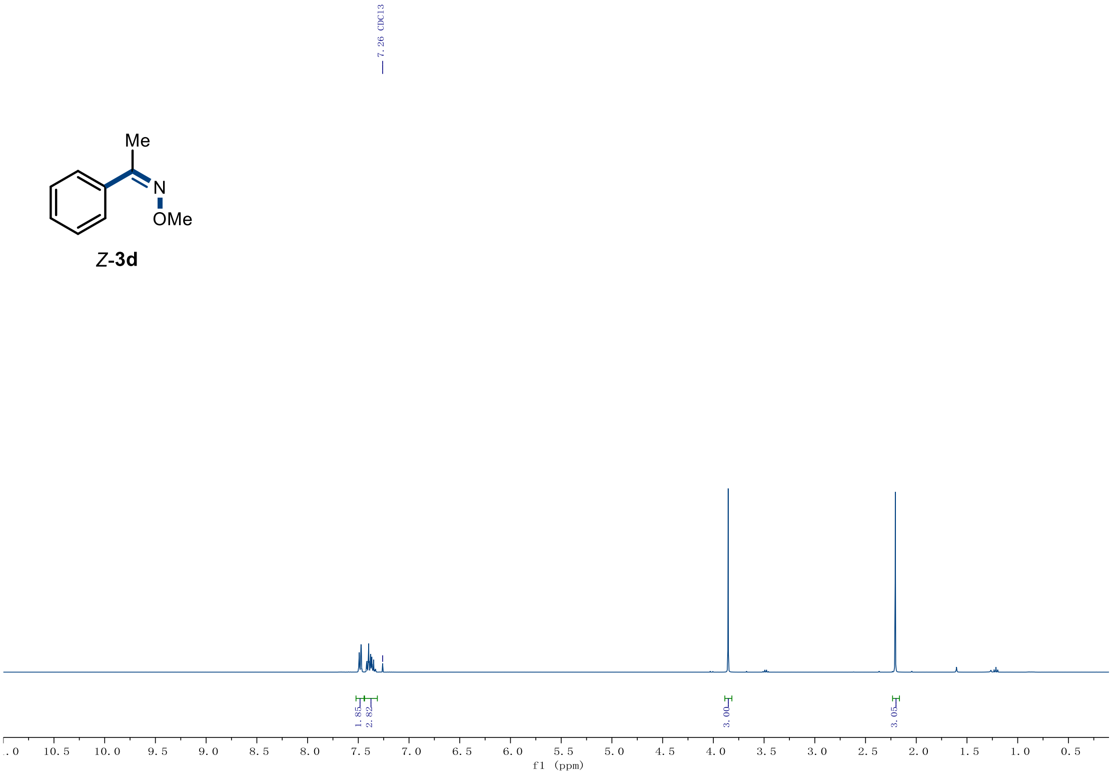

^{13}C NMR (151 MHz, $CDCl_3$)

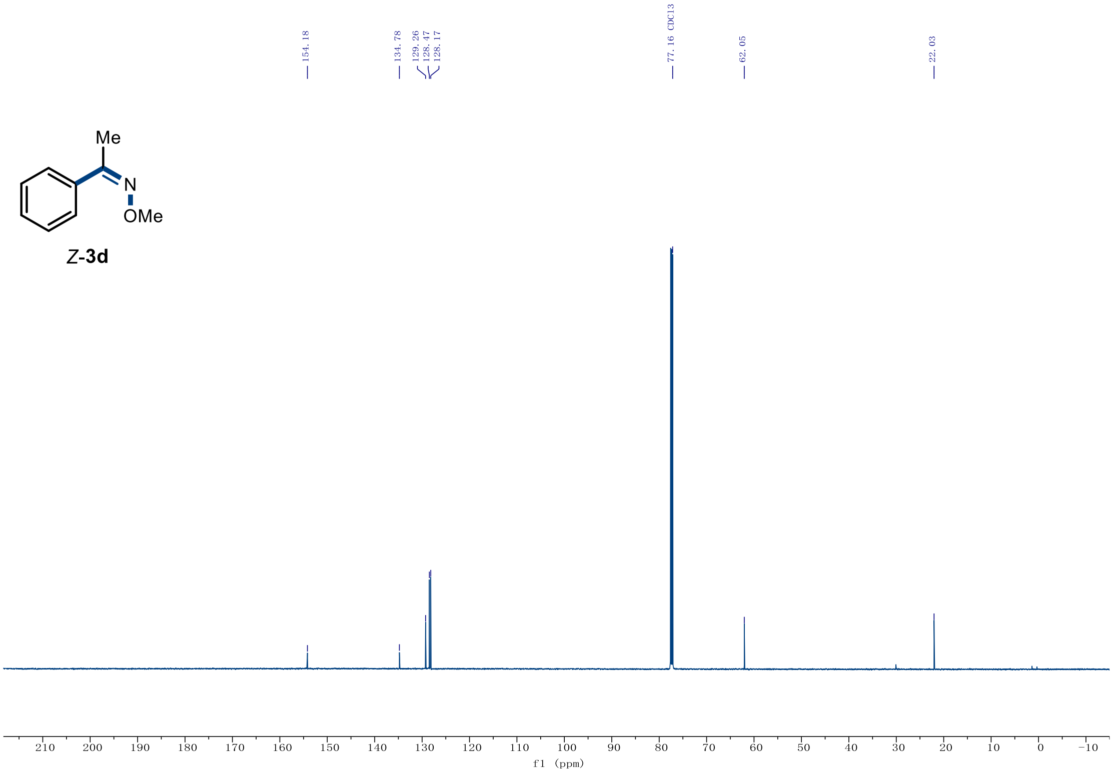

Isolated product - ^{1}H NMR (600 MHz, $CDCl_3$)

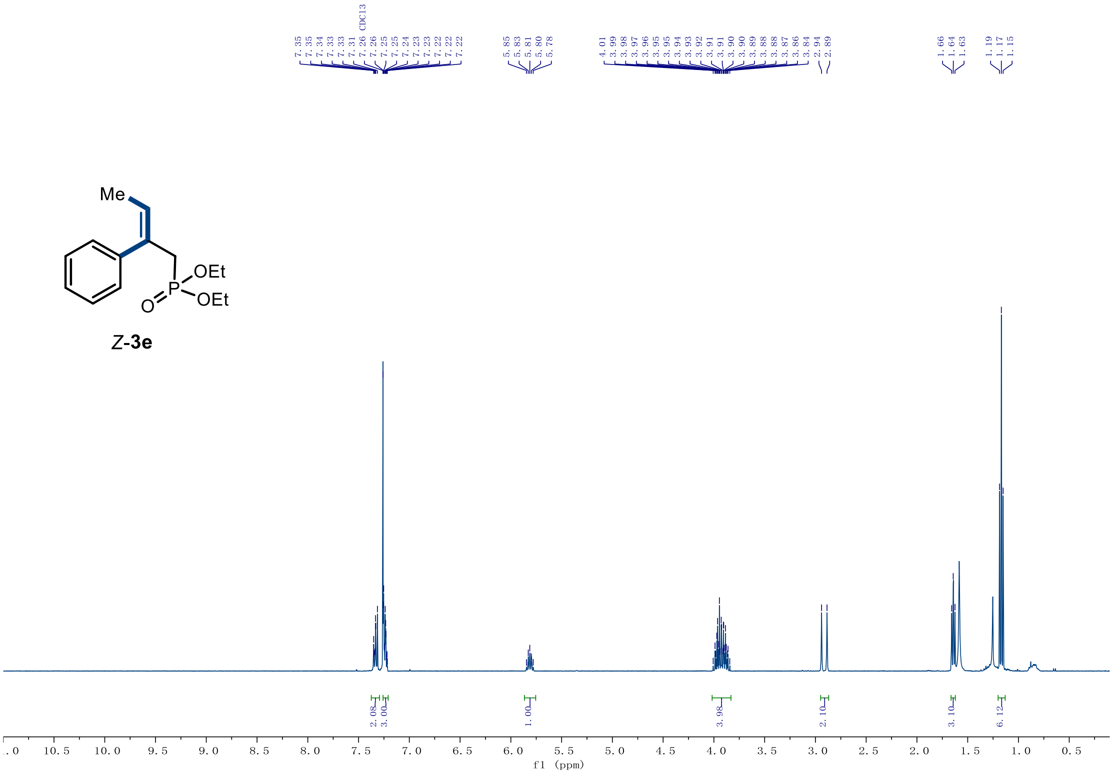

^{13}C NMR (151 MHz, $CDCl_3$)

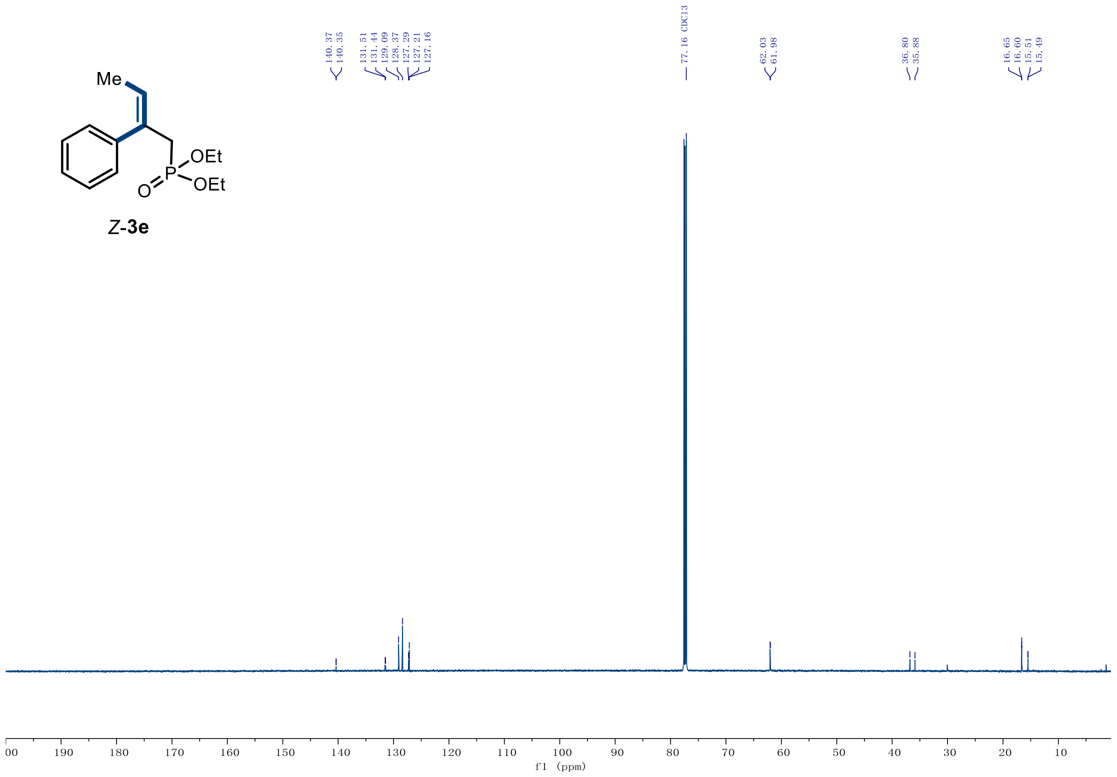

^{31}P NMR (243 MHz, $CDCl_3$)

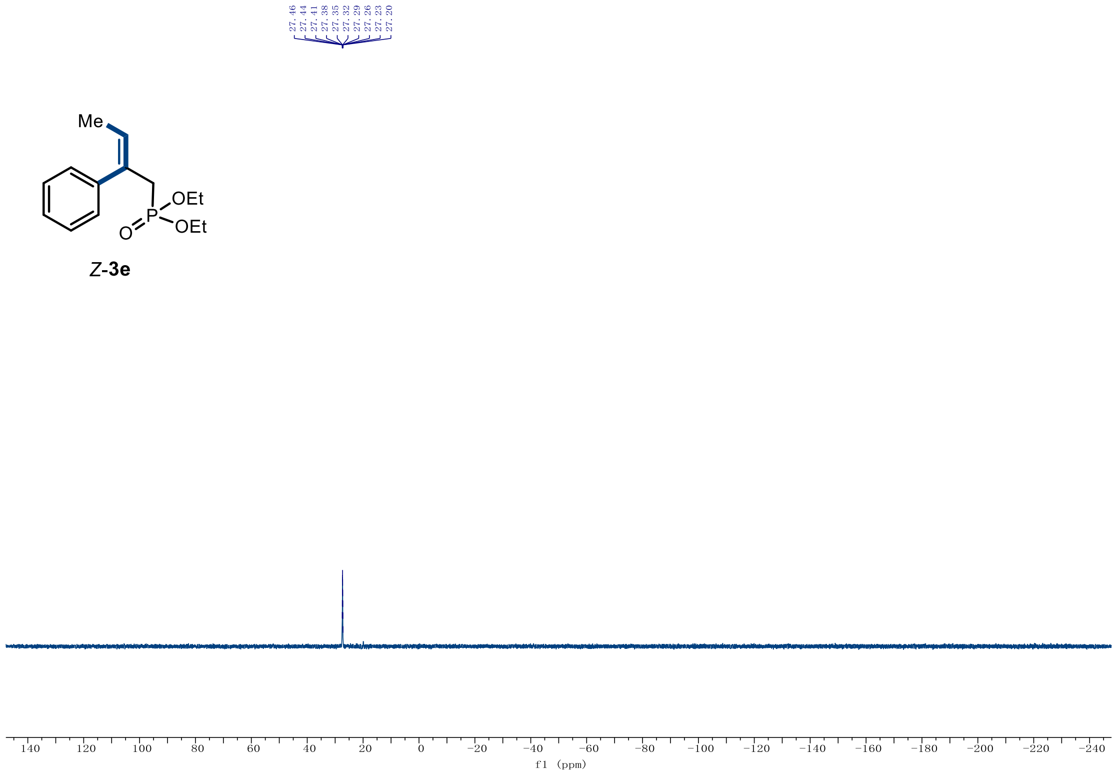

Isolated product - ¹H NMR (600 MHz, $CDCl_3$)

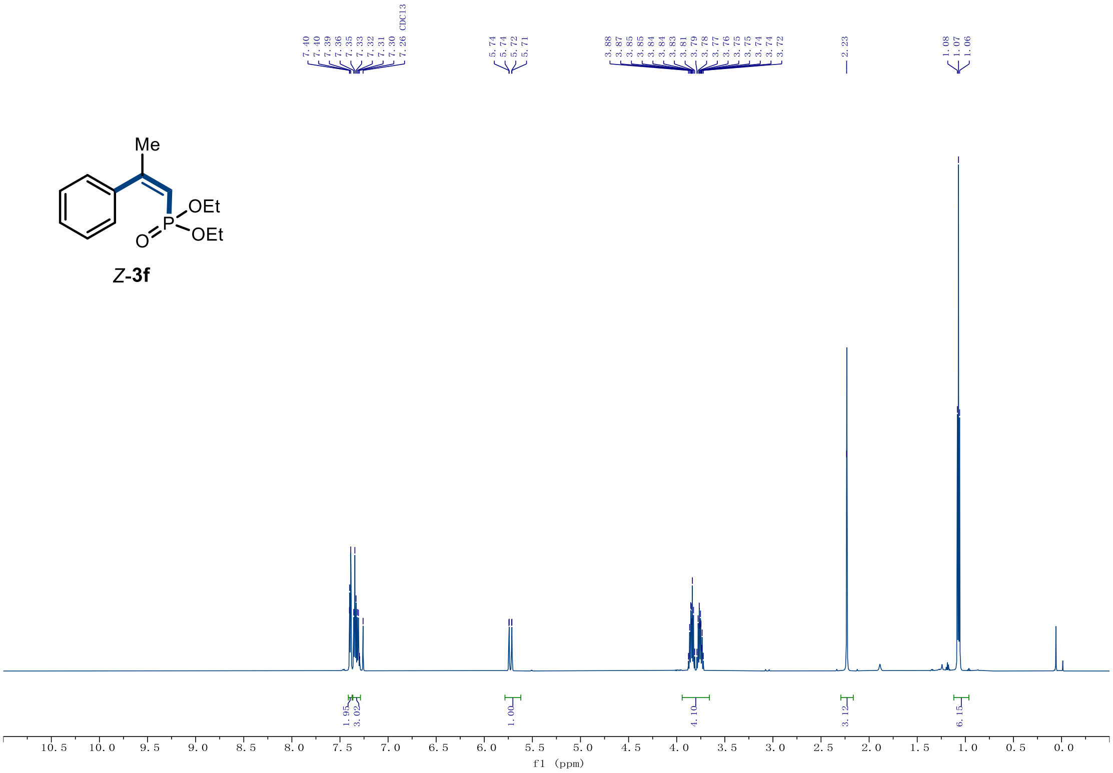

¹³C NMR (151MHz, $CDCl_3$)

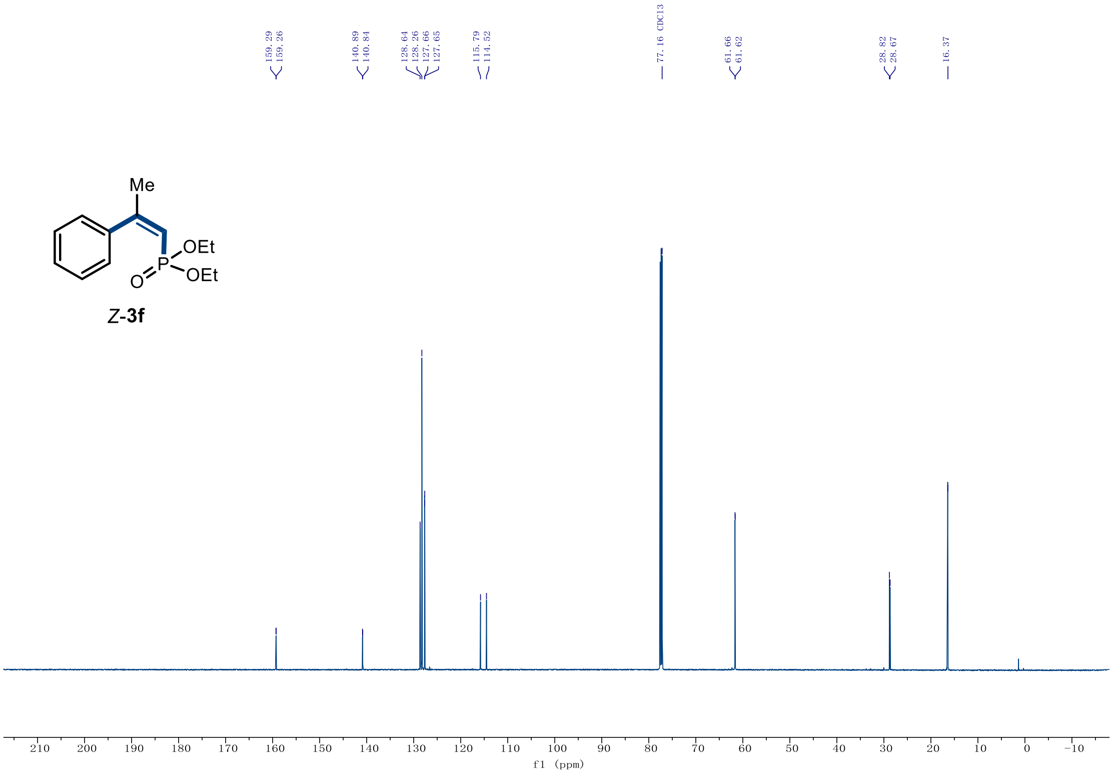

^{31}P NMR (243 MHz, CDCl$_3$)

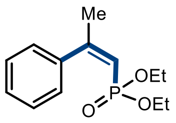

Z-**3f**

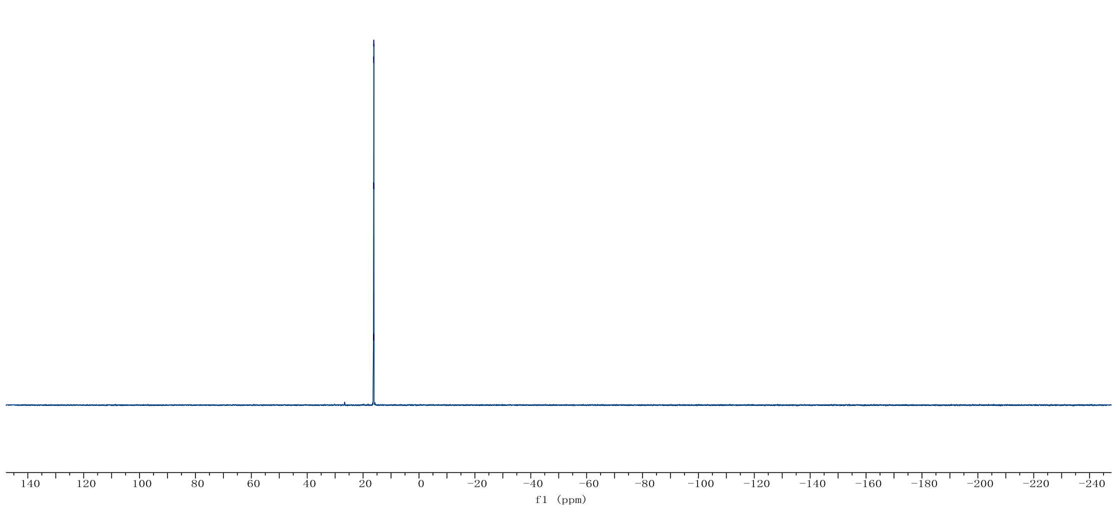

EnT-based Processes – *[2+2]* Photocycloaddition *(Figure 4e&4f in the main manuscript)*

Isolated product - ^{1}H NMR (600 MHz, $CDCl_3$)

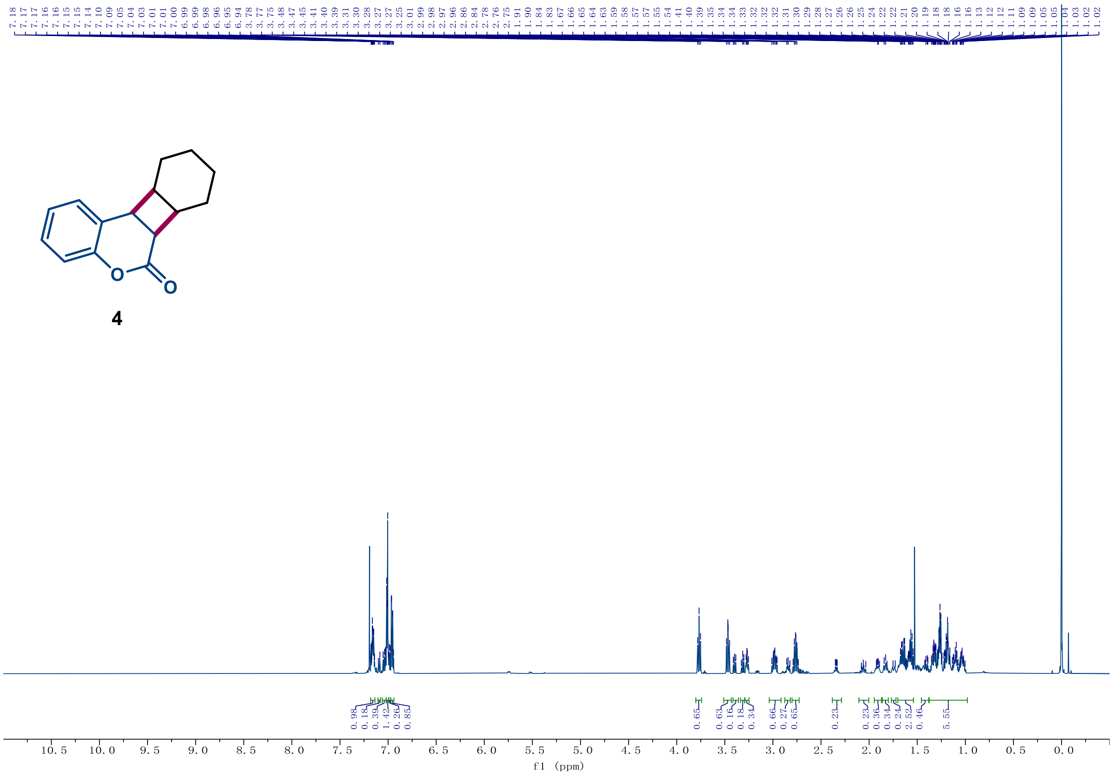

^{13}C NMR (151 MHz, $CDCl_3$)

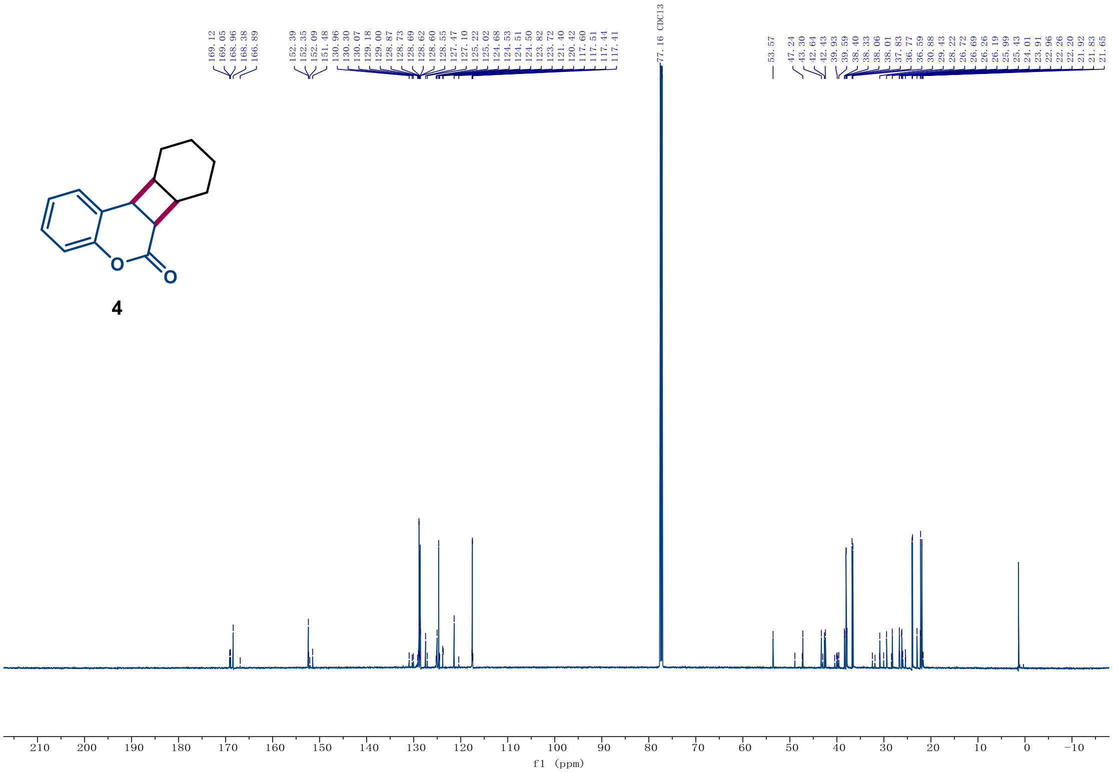

GC-MS analysis – detection of three isomers of **4**

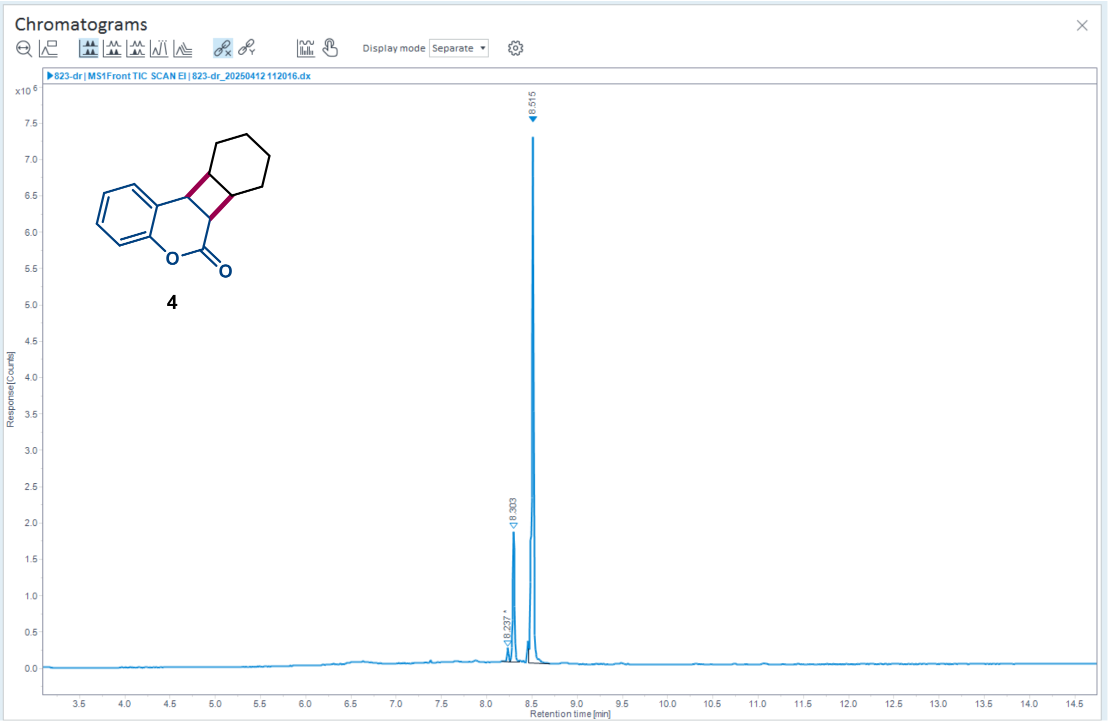

Isolated product - ¹H NMR (600 MHz, $CDCl_3$)

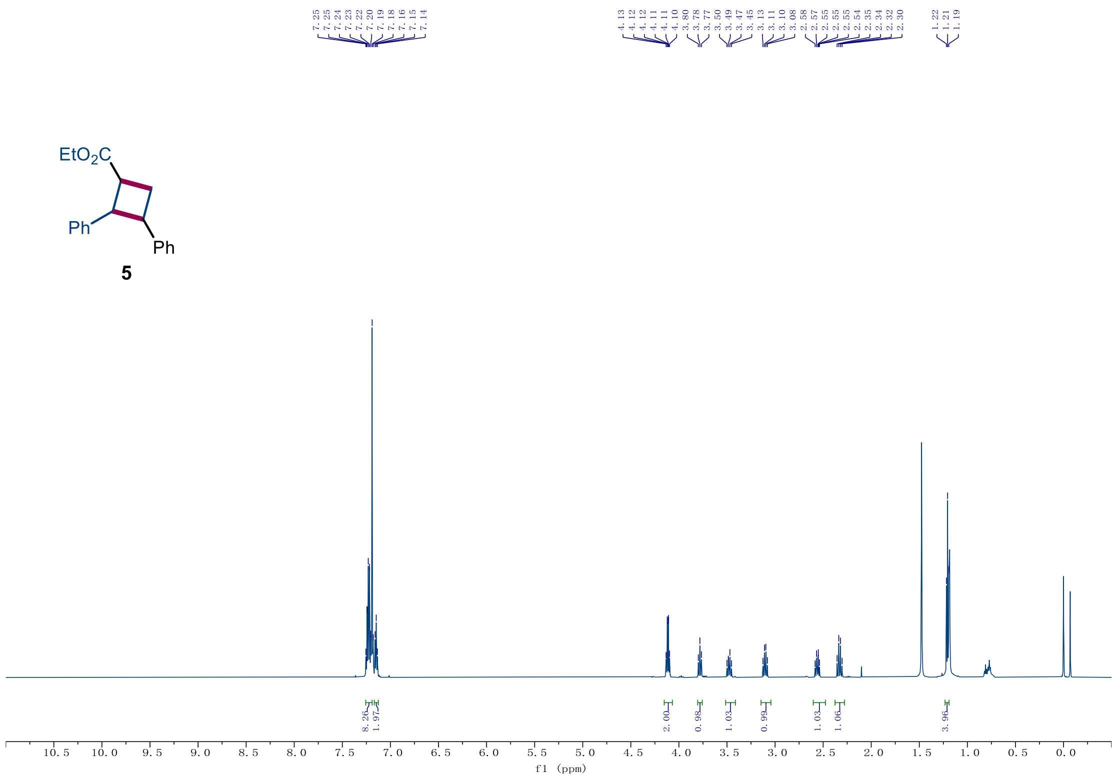

^{13}C NMR (151 MHz, $CDCl_3$)

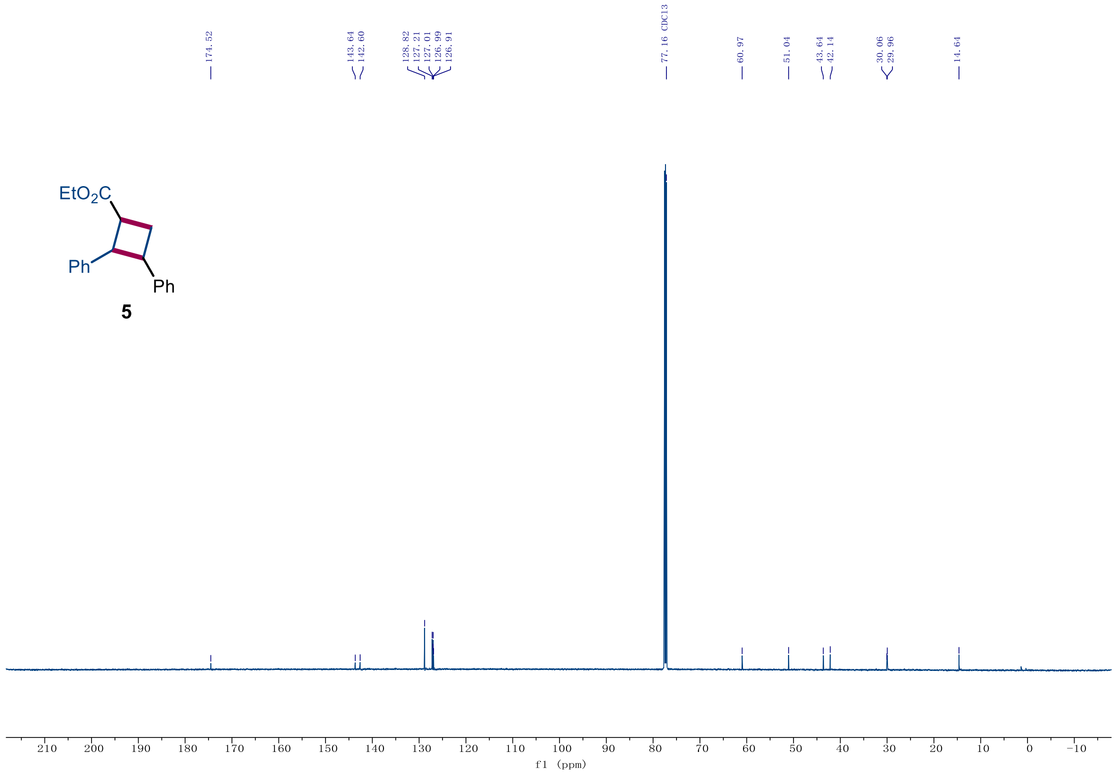

Isolated product - ^{1}H NMR (600 MHz, $CDCl_3$)

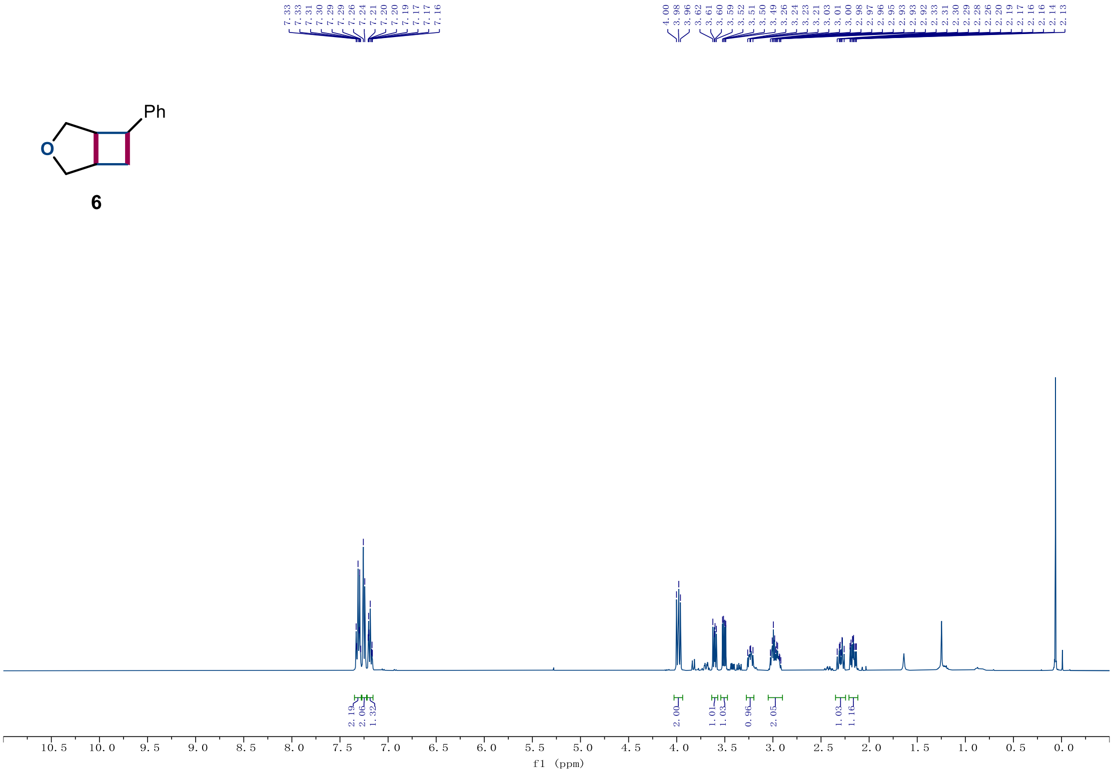

^{13}C NMR (151 MHz, $CDCl_3$)

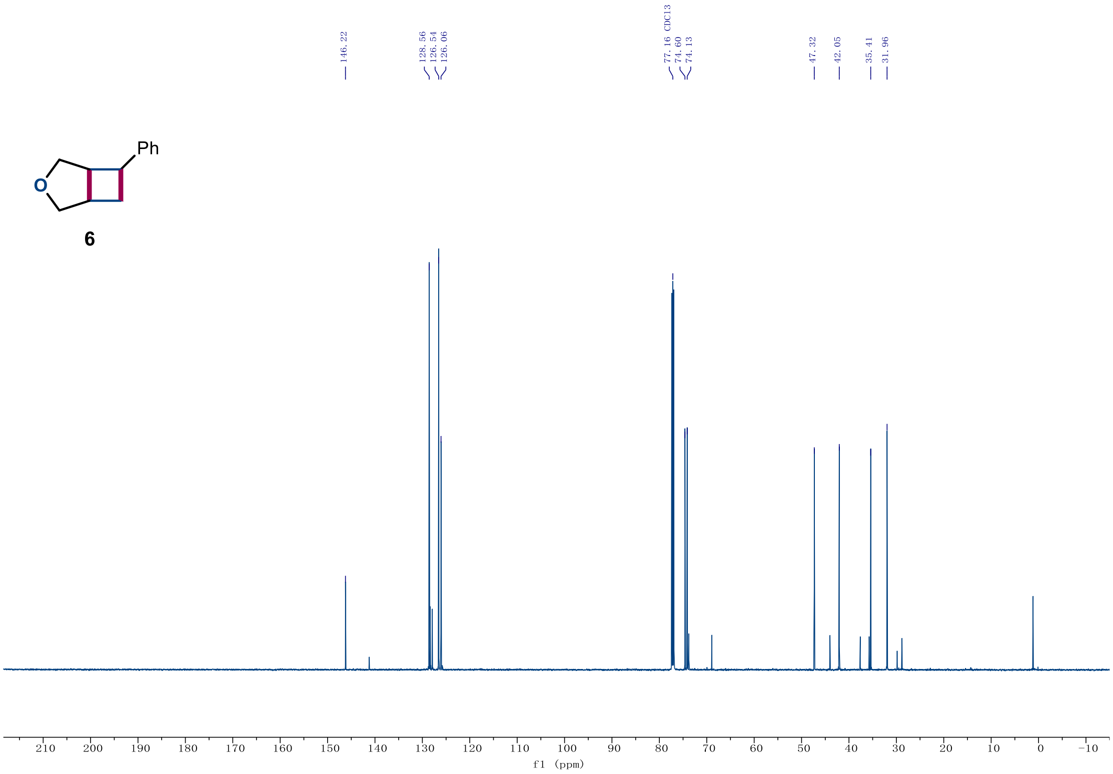

Crude reaction mixture - ^{1}H NMR (600 MHz, CDCl$_3$)

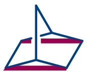

7

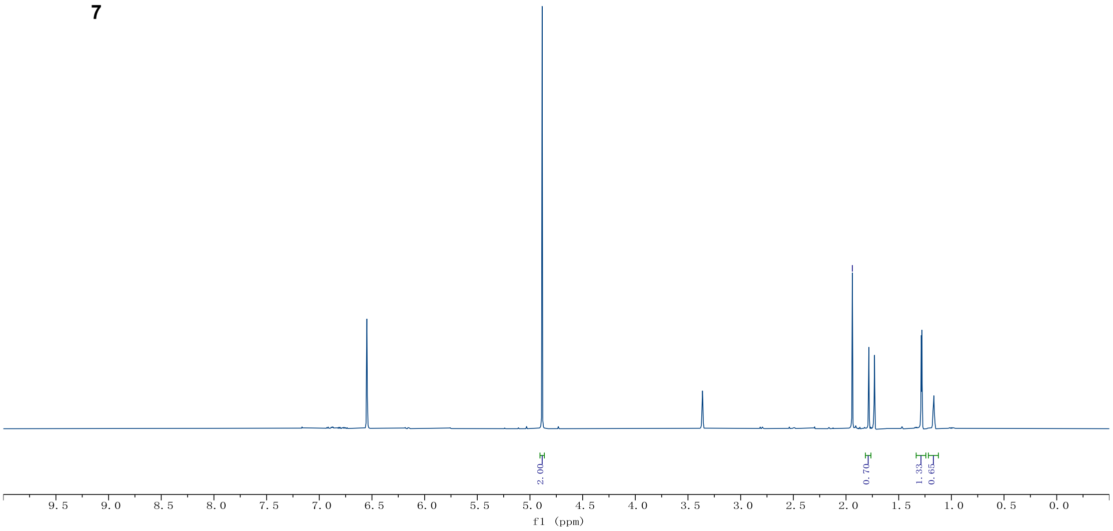

Supplement: Supplementary file 1 — Supporting Information [file ANIE-64-e202509770-s001.pdf]
